# Integrating GWAS meta-analysis with human brain cell mapping implicates the amygdala and the midbrain in the pathogenesis of tinnitus

**DOI:** 10.1101/2025.11.28.25340151

**Published:** 2025-12-01

**Authors:** Nick M.A. Shubert, Shuyang Yao, Maryam Kazemi Naeini, Natalia Trpchevska, Charles Bishop, Argyro Bizaki-Vallaskangas, Linda Broer, Fabio Castellana, Kaare Christensen, Jacob v. B. Hjelmborg, Maria Pina Concas, Aurora Santin, Giorgia Girotto, Giuseppe Giovanni Nardone, Licia Iacoviello, Estonian Biobank Research Team, Jonas Mengel-From, Scott Gallagher, Alexander Gudjonsson, Vilmundur Gudnason, Nancy L. Heard-Costa, Sudha Seshadri, Howard J. Hoffman, Chuan-Ming Li, Jaakko Kaprio, Joel Rämö, Elmo Saarentaus, Johannes Kettunen, Kristi Krebs, Anna K. Kähler, Lenore J. Launer, Hampton Leonard, Mike A. Nalls, Patrik K.E. Magnusson, Joyce van Meurs, Lili Milani, Antti Mäkitie, Berthe C. Oosterloo, Marianne Nygaard, Teemu Palviainen, Sheila Pratt, Nicola Quaranta, Rodolfo Sardone, Claudia L. Satizabal, John M. Schweinfurth, Eric Shiroma, Eleanor Simonsick, Lifelines Cohort Study, Christopher Spankovich, Patrick F. Sullivan, Andre Goedegebure, Andries Paul Nagtegaal, Sonja J. Pyott, Christopher R. Cederroth, Maxim B. Freidin, Frances MK Williams

**Affiliations:** 1. Department of Otorhinolaryngology / Head and Neck Surgery, University of Groningen, University Medical Center Groningen, The Netherlands, 9700 RB Groningen, The Netherlands; 2. Department of Medical Epidemiology and Biostatistics, Karolinska Institutet, 17177 Stockholm, Sweden; 3. Department of Twin Research and Genetic Epidemiology, School of Life Course and Population Sciences, King’s College London, London, UK; 4. Department of Physiology and Pharmacology, Karolinska Institutet, 17177 Stockholm, Sweden; 5. Department of Otolaryngology and Communicative Sciences, The University of Mississippi Medical Center, Jackson, MS 39216, USA; 6. Tampere University, Faculty of Medicine and Health Technology, Department of Otolaryngology, Tampere, Finland; 7. Pirkanmaan hyvinvointialue, Department of Ear and Oral Diseases, Tampere, Finland; 8. Department of Internal Medicine, Erasmus Medical Center, 3015 GD Rotterdam, The Netherlands; 9. Unit of Epidemiology and Statistics, Local Health Authority of Taranto, Taranto, Italy; 10. The Danish Twin Registry, Department of Public Health, University of Southern Denmark, 5000 Odense C, Denmark; 11. Department of Clinical Genetics, Odense University Hospital, 5000 Odense C, Denmark; 12. Department of Clinical Biochemistry and Pharmacology, Odense University Hospital, 5000 Odense C, Denmark; 13. Institute for Maternal and Child Health — IRCCS, Burlo Garofolo, 34127 Trieste, Italy; 14. Department of Medicine Surgery and Health Sciences, University of Trieste, Trieste Italy; 15. Department of Epidemiology and Prevention, I.R.C.C.S. Neuromed, Pozzilli, Italy; 16. Department of Medicine and Surgery, LUM University, Casamassima, Italy; 17. Estonian Genome Centre, Institute of Genomics, University of Tartu, Tartu, Estonia; 18. Decibel Therapeutics Inc.; 19. Icelandic Heart Association, 201 Kopavogur, Iceland; 20. Faculty of Medicine, University of Iceland, 101 Reykjavik, Iceland; 21. Department of Neurology, Boston University Chobanian & Avedisian School of Medicine, Boston, MA 02118, USA; 22. Framingham Heart Study, Framingham, MA 01702, USA; 23. Glenn Biggs Institute for Alzheimer’s & Neurodegenerative Diseases and Department of Population Health Sciences, University of Texas Health Sciences Center, San Antonio, TX 78229, USA; 24. Epidemiology, Statistics, and Population Sciences, National Institute on Deafness and Other Communications Disorders (NIDCD), NIH, Bethesda, MD 20892, USA; 25. Institute for Molecular Medicine Finland (FIMM), University of Helsinki, 00014 Helsinki, Finland; 26. Systems medicine, Research unit of Population Health, University of Oulu, 90220 Oulu, Finland; 27. Biocenter Oulu, University of Oulu, 90220 Oulu, Finland; 28. Finnish Institute for Health and Welfare, 00271 Helsinki, Finland; 29. Laboratory of Epidemiology and Population Sciences, Intramural Research Program National Institute on Aging, Bethesda, MD 20892, USA; 30. Laboratory of Neurogenetics, National Institute on Aging, National Institutes of Health, Bethesda, MD 20892, USA; 31. Center for Alzheimer’s and Related Dementias, Bethesda, MD 20892, USA; 32. Data Tecnica, Washington DC, 20037, USA; 33. Department of Otorhinolaryngology, Head and Neck Surgery, University of Helsinki and Helsinki University Hospital, 00029 HUS, Helsinki, Finland; 34. Department of Otorhinolaryngology, Erasmus Medical Center, 3015 GD Rotterdam, The Netherlands; 35. Department of Communication Science & Disorders, University of Pittsburgh, Pittsburgh, Pa 15260, USA; 36. Otolaryngology Unit, Department of Translational Biomedicine and Neuroscience (DiBrain), University of Bari, Policlinico di Bari, Italy; 37. Laboratory of Epidemiology and Population Sciences, National Institute on Aging, Baltimore, MD 21224, USA; 38. Longitudinal Studies Section, Translational Gerontology Branch, National Institute on Aging, Baltimore, MD 21224, USA; 39. LifeLines Cohort Study and Biobank, Groningen, the Netherlands; 40. Department of Genetics, University of North Carolina, Chapel Hill, NC 27516, USA; 41. Department of Neuroscience, School of Medicine and Health Science, Carl von Ossietzky Universität Oldenburg, 26129 Oldenburg, Germany; 42. Department of Otolaryngology, Head & Neck Surgery, University of Tübingen Medical Center, 72076 Tübingen, Germany

## Abstract

Tinnitus is a distressing condition affecting millions of people worldwide, yet treatment options remain very limited. Molecular evidence on the cellular origins of tinnitus in humans is lacking. Here, we performed a genome-wide association meta-analysis of clinically diagnosed and self-reported tinnitus on 888,214 individuals (75,250 tinnitus cases and 812,964 controls) from 19 independent cohorts. We identified five independent loci, two of which have not been reported previously. Mapping onto a single-cell transcriptomic atlas of the adult human brain, we found enrichment in amygdala excitatory neurons, midbrain-derived inhibitory neurons, and medial ganglionic eminence-derived interneurons, implicating the involvement of these cell types within tinnitus. Moreover, we observed that these cell-types overlapped with those associated with major depressive disorder, insomnia, and neuroticism, pathologies and personality traits which were also genetically correlated with tinnitus. Our findings confirm previously reported genetic associations with tinnitus and identify novel ones, highlighting the amygdala’s excitatory and the midbrain’s inhibitory neurons as crucial cell types involved in tinnitus pathophysiology. Our work, which leverages multiple cohorts, provides a framework for identifying genetic and cellular contributors to tinnitus and defining common mechanisms and treatments.

Tinnitus is the phantom perception of sounds in their physical absence. Its global prevalence is 14.2% of adults, with 1.4% of these individuals experiencing severe tinnitus (either self-reported or medically evaluated/diagnosed)^1^ that is incapacitating and accompanied with insomnia, depression, anxiety, stress, and increased suicide risk^2–4^. Evidence from twin and adoption studies showed that tinnitus is moderately heritable for bilateral tinnitus while unilateral tinnitus is less heritable^5,6^, and familial aggregation studies demonstrated that genetic transmission is stronger the more severe the tinnitus^7^. Subsequent genetic studies identified a first set of replicated common and rare variants^8,9^ and established the first evidence of genetic markers associated with tinnitus. The first large, published genome-wide association study (GWAS), conducted in >200,000 individuals with self-reported tinnitus from the UK Biobank (UKB) and replicated in the US Million Veteran Program (MVP) cohort, found three loci (lead SNPs: rs143424888, rs553448379, rs11249981) with small effect sizes, explaining approximately 6% of the heritability^8^. A recent meta-analysis of these two datasets identified 29 genome-wide significant loci, and a comparison with hearing loss suggested two new loci for tinnitus and suggests tinnitus as a distinct disorder separate from hearing difficulties^10^. A whole exome sequencing study of severe tinnitus reported an enrichment of rare missense variants in several synaptic genes, including *ANK2, AKAP9* and *TSC2,* in a Spanish cohort of Meniere’s Disease (MD) patients with catastrophic tinnitus (N=59). These findings were replicated in an independent Swedish population cohort with bothersome tinnitus (N=97)^9^. Further evaluation of the Swedish dataset with whole genome sequencing identified several large structural variants in the *CACNA1E, NAV2,* and *TMTM132D* genes^11^. Expanding the catalog of genetic associations and linking them to specific cellular mechanisms and disease pathologies is essential to understand the pathophysiology underlying tinnitus and to guide the development of new treatment approaches^12^.

In the present study, we analyzed summary statistics from 19 independent cohorts. These cohorts largely overlap with a recently published GWAS on hearing loss^13^. The current dataset is comprised of 888,214 individuals of European descent, including 75,250 tinnitus cases and 812,964 controls, and corresponding to a prevalence of 8.47% (Figure 1A, Supplementary Table 1). Cases were defined by either a clinical diagnosis of tinnitus (ICD9 (3891) & 10 (H93.1); FinnGen and EstBB, representing 72.3% of all cases with an average tinnitus prevalence of 2.93%) or self-reported tinnitus (all other cohorts, representing 27.6% of all cases and an average prevalence of 22.9%). Such difference in prevalence rates between diagnosed and self-report is consistent with individuals with more severe tinnitus seeking medical help^14^.

We completed a genome-wide meta-analysis of 11,950,440 imputed SNPs that passed quality control and identified five significant independent loci (represented by rs35098301 (Chromosome 2), rs35009431 (Chromosome 8), rs10877884 (Chromosome 12), rs2147632 (Chromosome 13), rs12922637 (Chromosome 16); P < 5×10^−8^) based on conditional and joint association analysis (COJO) (Figure 1B and 1C; Supplementary Table 2, 3). There was no evidence of population stratification. While quantile-quantile plot test statistics (GC) were established at 1.2103, the LD score intercept was 1.0103 (SE = 0.0085), indicating that inflation was likely due to polygenicity (Supplementary Figure 1). SNP-based heritabilities were estimated at 0.0137 (SE = 0.0009) on the observed scale and 0.0535 (SE = 0.0036) on the liability scale. Based on the forest plots, a leave-one-out meta-analysis was performed without the LL_cytosnp cohort, which had no impact on the regression coefficients and I^2^ values (Supplementary Table 4). Consequently, this cohort was retained in all subsequent analyses. Regional plots of independent and significant loci are presented in Supplementary Figure 2. The loci for tinnitus on chromosomes 12 and 13 (rs10877884, rs2147632) were not reported by a previous, recent GWAS meta-analysis on tinnitus^10^.

Gene mapping was performed on the identified loci using VEGAS2 (N_mapped_ = 17), FUMA (N_mapped_ posMap = 30; N_mapped_ eqtlMap = 37) and MAGMA (N_mapped_ = 17) pipelines (Supplementary Table 5-7). In total, 51 unique genes were mapped using a combination of these methods (Supplementary Figure 3). Seven genes were found to be common across all applied mapping methods (*MFHAS1*, *TMEM131*, *MON2*, *C8orf12*, *XKR6*, *SLC35G5*, *C12orf61*), some of which (*TMEM131* and *SLC35G5*) were previously reported^10^. Among the known genes associated with tinnitus^15^, *MON2* appears as a new candidate. *MON2* is expressed in the mouse cortex, hippocampus, thalamus and striatum (Allen Brain Atlas; https://atlas.brain-map.org/) and functions as a membrane traffic regulator between the Golgi and endosomes^16^, potentially regulating neuronal communication. However, its role in neuronal function in mammals remains largely unknown, underscoring the need for further research to clarify its contribution to neuronal homeostasis and tinnitus.

Previous genetic studies using GTEx suggested that tinnitus likely originates in the brain, with cellular enrichment in the cerebellum, the frontal cortex, the anterior cingulate cortex, and the basal ganglia^10^, regions also previously reported in a review by Husain & Khan^17^. To identify the cellular origins of tinnitus more accurately, we used a comprehensive human cell atlas derived from three postmortem donors, containing three million nuclei collected from approximately 100 dissections^18^. We used a recently published analysis pipeline utilizing stratified LD score regression to estimate SNP-heritability enrichment for a specific trait in identified cell clusters^19^. The dataset used by the analysis pipeline comprised 461 cell clusters and 31 superclusters^19^. Adjusting for multiple testing, we identified three superclusters, which were enriched in tinnitus with suggestive significance: the amygdala excitatory neurons (FDR = 0.067), the midbrain-derived inhibitory neurons (FDR = 0.067), and the medial ganglionic eminence interneurons (FDR = 0.067; Figure 2A, Supplementary Table 8). These superclusters largely overlapped with other traits known to be associated with tinnitus in epidemiological studies, including major depressive disorder, insomnia and neuroticism (Supplementary Figure 4)^2,20,21^. The superclusters also overlapped with schizophrenia and bipolar disorder. Correlation analysis confirmed a genetic significant correlation with major depressive disorder (Genetic correlation coefficient (Rg) = 0.32), neuroticism (Rg = 0.26), anxiety disorder (Rg = 0.37), insomnia (Rg = 0.19), post-traumatic stress disorder (Rg = 0.24), chronic pain (Rg = 0.30), hearing loss (Rg = 0.50) and vertigo (Rg = 0.22); Figure 2B, Supplementary Table 9).

To gain insight into the pathophysiology of tinnitus, a common auditory condition with comorbidities, we conducted a large GWAS meta-analysis. We confirmed three previously identified independent risk loci and identified two new ones located on chromosomes 12 and 13 (tagged by rs10877884 and rs2147632, respectively). A single-cell transcriptomic atlas of the adult human brain identified enrichment in amygdala excitatory neurons, midbrain-derived inhibitory neurons, and medial ganglionic eminence-derived interneurons. The amygdala consists of GABAergic interneurons, predominantly in the central and medial amygdala, as well as glutamatergic pyramidal-like neurons, located in the basolateral amygdala, and has been previously associated with tinnitus. Studies have, in general, pointed to the involvement of the limbic system in tinnitus^22^, and a previous tinnitus GWAS from Clifford et al. also showed enrichment of the amygdala using GTEx^10^. Both the ventromedial prefrontal cortex (vmPFC) and the nucleus accumbens (NAc), which together form a so-called “frontostriatal gating system,” receive input from the amygdala. Failure of this gatekeeping mechanism has been observed in individuals with tinnitus^23^ and has been proposed to influence the affective meaning of sensory stimuli, leading to a lack of suppression of irrelevant sensory signals and similarly may be involved in chronic pain^24^. Our findings suggest that the genetic influence of tinnitus is linked to amygdala excitatory neurons that may increase activity in the vmPFC and the NAc and impair proper frontostriatal gating. The resulting corticofugal influence (top-down modulation) may ultimately lead to increased activity in the inferior colliculus, consistent with observations of increased Wave V amplitude and latency of the auditory brainstem responses in humans with tinnitus^25,26^. This hypothesis is further supported by the involvement of midbrain-derived inhibitory neurons and medial ganglionic eminence -derived interneurons, the second and third most enriched supercluster in our GWAS, in tinnitus. Both neuron types are mainly GABAergic cells, present in the inferior colliculus, and involved in the suppression of hyperexcitability. Disrupted GABAergic function in animal models of tinnitus is well established^27^, and GABAergic enhancement in mice abolishes tinnitus^28^ is consistent with the central gain hypothesis whereby peripheral deafferentation (e.g., following noise-induced hearing loss) triggers exacerbated central compensatory activity^29^. In humans, the role of GABA remains debated, largely due to conflicting results in clinical trials repurposing GABA enhancing drugs for tinnitus^30^. Nevertheless, our findings reinforce the involvement of altered neuronal excitability in humans with tinnitus.

Compared to previous tinnitus GWASs, our study highlights the implications of differences in cohort composition. Despite our study being the largest tinnitus GWAS study performed to date, we did not identify as many loci as the recent GWAS meta-analysis, combining UKB with the MVP, which reported 29 loci across 596,925 individuals (cases 203,758)^10^. We suspect that this difference arises from the cohorts examined: specifically, in the MVP cohort, almost 72% of tinnitus cases are ICD diagnosed and, therefore, represent more severe cases of tinnitus (with a prevalence of severe tinnitus more than 15 times greater than that found in the general population). In contrast, only two of our 19 cohorts used ICD codes for classifying tinnitus, with the proportion of ICD-diagnosed tinnitus cases in our study accounting for only 25% of the total cases. Moreover, most of the cohorts using self-reports in our study failed to consider severity. Given the greater heritability of severe tinnitus^6,7^, we suspect that the fewer number of genetic associations found in our study despite the large sample size, may reflect the greater genetic heterogeneity of less severe, predominantly self-reported tinnitus cases. This heterogeneity, in turn, may reduce the number of loci that reached significance compared to studies enriched for severe, ICD-diagnosed cases of tinnitus, which may reflect more homogenous causes (e.g., combat associated noise induced hearing loss). Ultimately, examination of cohorts with diverse case composition is needed to capture the heterogeneity of tinnitus and disentangle its multiple pathophysiological pathways and subtypes^31^.

In addition to the diversity in the phenotyping of tinnitus in our cohorts, there is also a lack of information on hyperacusis status. Hyperacusis is defined as hypersensitivity to sounds and commonly co-occurs with tinnitus^32^. In humans, hyperacusis shows amongst the strongest associations reported with severe tinnitus, with an odds ratio (OR) of 77.4 (95% confidence interval of 35.0 – 171.3), even after adjusting for hearing loss^33^. Studies in animal models of tinnitus also indicate the common co-occurrence of hyperacusis^34^. Like tinnitus, hyperacusis is heterogeneous, ranging in severity and sometimes accompanied with pain-induced sound sensitivity. Hyperacusis was not evaluated in our cohorts. When coded in the medical registries as H93.23 (coded under H93.2 abnormal auditory perceptions), it was certainly underrepresented, with only 589 patients in the whole EstBB, highlighting the poor clinical registration of this condition. Given that nearly 90% of individuals with severe tinnitus experience hyperacusis, and 50% of them present with the severe form of hyperacusis^33^, we expect a strong confounding effect in our GWAS.

Overall, our tinnitus GWAS, including 19 independent cohorts and 75,250 cases, provides further support for a genetic basis of tinnitus and implicates specific neuronal cell types in the pathophysiology of tinnitus. These findings provide novel and critical insight into conserved central mechanisms underlying tinnitus, regardless of severity, and guide future work developing treatments for a broader range of tinnitus forms.

## Materials and Methods

### Population cohorts

A total of 888,214 adult male and female participants were included from the following 19 population-based cohort studies: Age, Genes/Environment Susceptibility - Reykjavik (AGES; n = 3,134), the Danish Twin Registry (DTR; n = 1,314), the Estonian Genome Center at the University of Tartu (EstBB; n = 195,394), FinnGen R12 (n = 447,339), Framingham Heart Study (FHS; n = 3,041), the Lifelines (LL) cohort study (original genotyping cohort, UMCG Genetics Lifelines Initiative (UGLI) release 1, UGLI release 2; n = 54,184), Moli Sani cohort study (n = 1,198), the Rotterdam Study (RS, cohorts 1 – 3; n = 6,146), the Great Age Study (SA; n = 1,780; formerly known as Salus in Apulia study), Health ABC (HABC; n = 1288), three sub-studies of the Swedish Twin Registry: Screening Across the Lifespan Twin (SALT; n = 10,693), SALTY - young (n = 4,436), Screening Twin Adults: Genes and Environment (STAGE; n = 8,310), TwinsUK (n = 2,197), UK Biobank (UKB; n = 147,760). All participants provided written informed consent; ethical approval had been obtained locally, and under an umbrella license in Stockholm (*Regionala etikprövningsnämnden, #*2015/2129–31/1). The Declaration of Helsinki was adhered to in all cohorts. Phenotype definition was based on self-administered questionnaires regarding tinnitus and ICD-9 & −10 diagnoses of tinnitus (EstBB and FinnGen). Mean age overall was 49.0 years. A detailed description of phenotype definition, case control distribution, and age range for each study can be found in Supplementary Table 1 and Supplementary Information.

### Genome-wide association studies and meta-analysis

GWAS was carried out for each cohort locally, and summary statistics were collected for each study. Standardized quality control was performed using EasyQC software^35^, followed by meta-analysis using GWAMA software^36^. Briefly, the quality control steps with EasyQC involved excluding monomorphic SNPs, SNP with missingness < 0.05, duplicate SNPs, and SNPs with an imputation score < 0.5. After harmonizing allele coding and marker names, uniformed summary statistics were produced. LD score regression was applied to estimate the impact of population stratification and other confounders on test statistic inflation in cohorts with sample size n > 5,000 for QC-ed and harmonized GWAS data^37^. The genome build was hg19. A genome-wide significance threshold was defined as P < 5 × 10^−8^. Variants found in less than five studies were excluded. To identify lead SNPs, a conditional & joint association analysis (COJO) was performed^38^.

### SNP-heritability and genetic correlation

To evaluate the extent of shared genetic architecture based on common gene variants and hypothesize about association with potential risk factors, LD score regression was used to estimate SNP heritability and to assess genetic correlation between tinnitus and a range of other disorders and traits^37^. The list comprised 12 phenotypes and levels of significance were set at FDR < 0.05.

### Gene mapping

Mapping of genes was performed using a combination of methodolgical software, including VEGAS2^39^, FUMA (posMap, eqtlMap)^40^ and MAGMA^41^.

### Brain enrichment analysis

This analysis was performed using recently published methodology to identify cell clusters in the brain that are associated with specific traits^19^. This analysis utilized stratified LD score regression to estimate SNP-heritability enrichment for a trait in genes typically expressed by specific cell types. Single-nucleus RNA-sequencing gene expression data from a published atlas^18^, including approximately 100 dissections and three million nuclei from three postmortem donors, was used. Cells were allocated to 461 clusters and 31 superclusters. Top decile of expression proportion (TDEP) genes^19^ were used to evaluate enrichment of the SNP-heritability of tinnitus. An FDR < 0.05 was used for significant enrichment, and an FDR < 0.1 for suggestive enrichment.

## Supplementary Material

Supplement 1

## Figures and Tables

**Figure 1:**
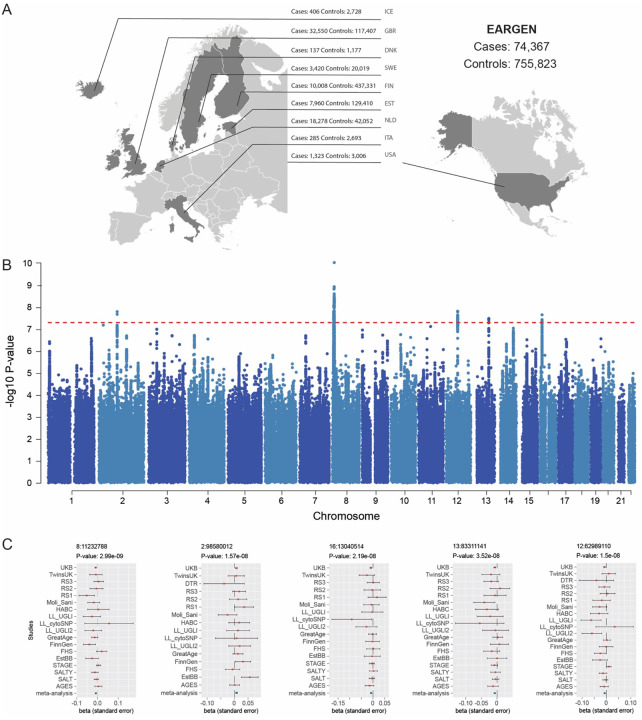
Discovery of risk loci for tinnitus. A) Summary of contributing cohorts. More detailed information on the cohorts can be found in Supplementary Table 1. B) Manhattan plot from the GWAS meta-analysis on tinnitus, showing five genome-wide significant independent loci. The x-axis represents positions across the chromosomes included in the analysis, and the y-axis represents −log_10_ p-values from the meta-analysis. The red-dotted line is p-value 5 × 10^−8^ which represents genome-wide significance. C) Forest plots from leading genome-wide significant SNPs rs35098301, rs35009431, rs10877884, rs2147632, rs12922637. The x-axis represents the beta coefficient, and the y-axis represents the different cohorts included in the analysis. The red and green dots are the beta coefficients, and the error bars represent the standard error.

**Figure 2:**
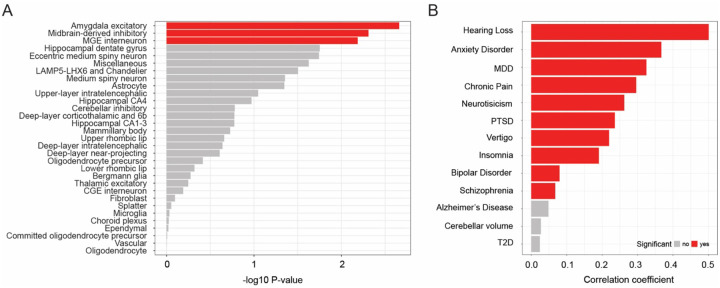
Enrichment of brain cell clusters in tinnitus and genetic correlations between tinnitus and other traits. A) Brain cell supercluster enrichment analysis for tinnitus. On the x-axis −log10 p-values are plotted. An FDR < 0.1 was considered significant (red). B) Bar plot showing results of the LD score regression genetic correlation analysis. FDR < 0.05 was used to identify significant correlations. Abbreviations: MGE = medial ganglionic eminence, CGE = caudal ganglionic eminence, MDD = major depressive disorder, PTSD = post-traumatic stress disorder, T2D = type 2 diabetes mellitus.

## Data Availability

The meta-GWAS summary statistics are deposited and available in Zenodo (https://zenodo.org/records/17639792) The codes used for the present study are available in the following repository (https://github.com/translational-audiology-lab/GWAS_tinnitus).
