# Supplementary material for: Integrating GWAS meta-analysis with human brain cell mapping implicates the amygdala and the midbrain in the pathogenesis of tinnitus
Source: medRxiv. 2025 Dec 1:2025.11.28.25340151. Preprint. [Version 1] doi: 10.1101/2025.11.28.25340151 (PMC12687797; doi:10.1101/2025.11.28.25340151)
Supplement: Supplement 1 [file media-1.pdf]

Supplementary information

Contents:

1. **Cohort descriptions**
2. **Supplementary figures**
3. **Supplementary Tables**
4. **References**
5. **Study specific acknowledgements**

1. Cohort descriptions

Age, Gene/Environment Susceptibility Reykjavik Study: AGES-Reykjavik Study

The Reykjavik Study cohort originally comprised a random sample of 30,795 men and women born in 1907-1935 and living in Reykjavik in 1967[1]. A total of 19,381 people attended, resulting in 71% recruitment rate. The study sample was divided into six groups by birth year and birth date within month. One group was designated for longitudinal follow up and was examined in all stages. One group was designated a control group and was not included in examinations until 1991. Other groups were invited to participate in specific stages of the study. Between 2002 and 2006, the AGES-Reykjavik study re-examined 5764 survivors of the original cohort who had participated before in the Reykjavik Study. The study is approved by the Icelandic National Bioethics Committee, VSN: 00-063.

The Danish Twin Registry

The Danish Twin Registry (DTR) sample included 1,314 individuals (137 cases and 1177 controls) collected as part of the study of Middle Age Danish Twins (MADT, N=1,055) and the Longitudinal Study of Aging Danish Twins (LSADT, N=259)[2]. MADT was initiated in 1998 and includes 4,314 twins randomly chosen from the birth years 1931-1952. Surviving participants were revisited from 2008 to 2011, where the blood samples used in the present study were collected. The survey data used in the present study was obtained from the Omnibus 2 survey undertaken in 2002[2]. LSADT was initiated in 1995 and includes twins aged 70 years and older. Follow-up assessments were conducted every second year through 2005. The individuals included here all participated in the 1997 assessment, where blood samples were collected from same sex twin pairs, and in the 2001 assessment where the survey data used in the present study was collected[2].

Written informed consents were obtained from all participants. Collection and use of biological material, and survey and registry information were approved by the Regional Scientific Ethical Committees for Southern Denmark (MADT: S-VF-19980072, LSADT: S-VF-20040241). The study is registered in SDU’s internal list (notification no. 10.903) and complies with the rules in the General Data Protection Regulation.

EstBB

The Estonian Biobank (EstBB) is a large data-rich population-based biobank, covering approximately 20% of the adult population in Estonia (N=~210,000). All EstBB participants have signed an informed consent form and provided blood samples for genotyping. Electronic health records are regularly retrieved by linking to the national health databases and registries, such as the National Health Insurance Funds (NHIF) database, cause of death register, and hospital records[3].

For the current study, we identified 8,843 tinnitus cases from participants' EHRs, defined as individuals with at least two occurrences of the ICD-10 diagnosis code H93.1. Controls (n= 186,551)

were defined as undiagnosed participants. Individuals with H93.2 (Other abnormal auditory perceptions), Z96.2 (Presence of otological and audiological implants), Z97.4 (Presence of external hearing-aid) ICD10 diagnoses were excluded from all analyses. The analysis was performed using REGENIE version 2.2.4[4], with gender, birth year, and the first 10 principal components included as covariates.

The activities of the EstBB are regulated by the Human Genes Research Act, which was adopted in 2000 specifically for the operations of the EstBB. Individual level data analysis in the EstBB was carried out under ethical approval 1.1-12/624 from the Estonian Committee on Bioethics and Human Research (Estonian Ministry of Social Affairs), using data according to release application 6-1/GI/131 from the Estonian Biobank.

FinnGen

The FinnGen research project (www.finngen.fi) was launched in 2017 with an aim to improve human health through genetic research. The project combines genome information with digital health care data from national registries. The genotype data are linked to national hospital discharge, death, cancer, and medication reimbursement registries using the national personal identification numbers. The FinnGen study aims to combine approximately 200,000 existing samples from Finnish biobanks with approximately 300,000 samples from ongoing collections. Once final, the data resource will cover roughly 10% of the Finnish population. The present study comprised data of 447,339 Finnish adults (10,008 cases and 437,331 controls) from FinnGen Preparatory Phase Data Freeze 10.

Patients and control subjects in FinnGen provided informed consent for biobank research, based on the Finnish Biobank Act. Alternatively, separate research cohorts, collected prior the Finnish Biobank Act came into effect (in September 2013) and start of FinnGen (August 2017), were collected based on study-specific consents and later transferred to the Finnish biobanks after approval by Fimea (Finnish Medicines Agency), the National Supervisory Authority for Welfare and Health. Recruitment protocols followed the biobank protocols approved by Fimea. The Coordinating Ethics Committee of the Hospital District of Helsinki and Uusimaa (HUS) statement number for the FinnGen study is Nr HUS/990/2017.

The FinnGen study is approved by Finnish Institute for Health and Welfare (permit numbers: THL/2031/6.02.00/2017, THL/1101/5.05.00/2017, THL/341/6.02.00/2018, THL/2222/6.02.00/2018, THL/283/6.02.00/2019, THL/1721/5.05.00/2019 and THL/1524/5.05.00/2020), Digital and population data service agency (permit numbers: VRK43431/2017-3, VRK/6909/2018-3, VRK/4415/2019-3), the Social Insurance Institution (permit numbers: KELA 58/522/2017, KELA 131/522/2018, KELA 70/522/2019, KELA 98/522/2019, KELA 134/522/2019, KELA 138/522/2019, KELA 2/522/2020, KELA 16/522/2020), Findata permit numbers THL/2364/14.02/2020, THL/4055/14.06.00/2020, THL/3433/14.06.00/2020, THL/4432/14.06/2020, THL/5189/14.06/2020, THL/5894/14.06.00/2020, THL/6619/14.06.00/2020, THL/209/14.06.00/2021, THL/688/14.06.00/2021, THL/1284/14.06.00/2021,

THL/1965/14.06.00/2021, THL/5546/14.02.00/2020, THL/2658/14.06.00/2021, THL/4235/14.06.00/2021, Statistics Finland (permit numbers: TK-53-1041-17 and TK/143/07.03.00/2020 (earlier TK-53-90-20) TK/1735/07.03.00/2021, TK/3112/07.03.00/2021) and Finnish Registry for Kidney Diseases permission/extract from the meeting minutes on 4th July 2019.

The Biobank Access Decisions for FinnGen samples and data utilized in FinnGen Data Freeze 10 include: THL Biobank BB2017_55, BB2017_111, BB2018_19, BB_2018_34, BB_2018_67, BB2018_71, BB2019_7, BB2019_8, BB2019_26, BB2020_1, BB2021_65, Finnish Red Cross Blood Service Biobank 7.12.2017, Helsinki Biobank HUS/359/2017, HUS/248/2020, HUS/150/2022 § 12, §13, §14, §15, §16, §17, §18, and §23, Auria Biobank AB17-5154 and amendment #1 (August 17 2020) and amendments BB_2021-0140, BB_2021-0156 (August 26 2021, Feb 2 2022), BB_2021-0169, BB_2021-0179, BB_2021-0161, AB20-5926 and amendment #1 (April 23 2020)and it´s modification (Sep 22 2021), Biobank Borealis of Northern Finland_2017_1013, 2021_5010, 2021_5018, 2021_5015, 2021_5023, 2021_5017, 2022_6001, Biobank of Eastern Finland 1186/2018 and amendment 22 § /2020, 53§/2021, 13§/2022, 14§/2022, 15§/2022, Finnish Clinical Biobank Tampere MH0004 and amendments (21.02.2020 & 06.10.2020), §8/2021, §9/2022, §10/2022, §12/2022, §20/2022, §21/2022, §22/2022, §23/2022, Central Finland Biobank 1-2017, and Terveystalo Biobank STB 2018001 and amendment 25th Aug 2020, Finnish Hematological Registry and Clinical Biobank decision 18th June 2021, Arctic biobank P0844: ARC_2021_1001.

Framingham Heart Study

The Framingham Heart Study is a prospective longitudinal investigation of the development of atherosclerosis and its clinical sequelae. Study participants were recruited across three time periods. The study was initiated between 1948 and 1950 with the recruitment of 5,209 individuals ages 28 to 62 (including some spouse pairs, parent-offspring pairs and siblings) for the purpose of investigating the multiple factors involved in the development of cardiovascular disease[5]. This group, known as the "original cohort", has been examined every two years with a total of 32 examinations to date. Between 1971 and 1975, "offspring" of the original cohort and the offspring of spouses of the original cohort were recruited to examine among other goals the familial components of cardiovascular disease and its risk factors[6]. Between 2002 and 2005, the third generation (children of the offspring and grandchildren of the original cohort) was recruited[7]. The offspring cohort totalled 5,124 and the third generation totalled 4,095 at recruitment and have been examined every 4 to 8 years. The offspring cohort now has nice examinations completed and the third generation has two examinations completed.

G-EAR

Within the International consortium called G-EAR, we used individuals coming from the **Salus in Apulia Study** (formerly known as Great Age Study).

The “Salus in Apulia Study” is an ongoing population-based prospective cohort comprising 2,472 individuals aged 65 years and older and residing in Castellana Grotte, a town located near Bari, Puglia, in the Southeast of Italy. It focuses on the analysis of the impact of lifestyle habits on frailty, other age-related impairments and age-related disease outcomes. Salus is a public health initiative funded by the Italian Ministry of Health and Apulia Regional Government and carried on at IRCCS “S. De Bellis” that combines data from two previous populations: the baseline data (MICOL3, M3) were recorded from 2003 to 2005 and the follow-up data collected from 2013 to 2015 (GreatAGE Study - MICOL4, M4). The GreatAGE-M4 study has been described elsewhere[8]. The invitation also included MICOL participants who were in the correct age range (age 64 years and older). In the GreatAge-M4 examination, in addition to the assessment of clinical and lifestyle aspects, sensory-related outcomes were also evaluated together with neuropsychological features and genetic components. The study adhered to the “Standards for Reporting Diagnostic Accuracy Studies” (STARD) guidelines (http://www.stard-statement.org/), the “Strengthening the Reporting of Observational Studies in Epidemiology” (STROBE) guidelines (https://www.strobe-statement.org/).

Health, Aging, and Body Composition (HABC) Study

The HABC Study is a NIA-sponsored cohort study of the factors that contribute to incident disability and the decline in function of healthier older persons, with a particular emphasis on changes in body composition in old age. Between March 1997 and July 1998, 3,075 community-dwelling adults between the ages of 70 and 79 years (41% African American) were recruited to participate. Medicare beneficiary listings were used to recruit in metropolitan areas surrounding Pittsburgh, Pennsylvania, and Memphis, Tennessee. Eligibility criteria included having no difficulty walking one-quarter of a mile, climbing ten steps, or performing activities of daily living (transferring, bathing, dressing, and eating); no history of active treatment for cancer in the prior 3 years; and no plans to move from the area within 3 years.

The Lifelines Cohort Study

Lifelines[9,10] is a multi-disciplinary prospective population-based cohort study examining in a unique three-generation design the health and health-related behaviors of 167,729 persons living in the northern Netherlands. It employs a broad range of investigative procedures in assessing the biomedical, socio-demographic, behavioral, physical and psychological factors which contribute to the health and disease of the general population, with a special focus on multi-morbidity and complex genetics. Genotyping data was obtained in the original GWAS[11] (including approximately 15,000 individuals) and via the UMCG Genetics Lifelines Initiative (UGLI), which genotyped approximately 66,500 individuals. Additional information on phenotyping and genotyping in Lifelines can be found here: https://wiki.lifelines.nl/.

The general Lifelines protocol has been approved by the UMCG Medical ethical committee under number 2007/152.

Moli Sani

“Moli-sani” (www.moli-sani.org) is a cohort study aiming at evaluating the risk factors (environmental, genetics, biomolecular) linked to chronic-degenerative disease, especially cancer, cardiovascular disease and their intermediate phenotypes hypertension, diabetes, dyslipidemia, obesity and metabolic syndrome. Between March 2005 and April 2010, the study recruited participants 35-year-old residents of the Molise region through multistage sampling from city hall registries. First, townships in major areas were selected by cluster sampling. Within each township, participants aged 35 years and older were selected by simple random sampling. For each selected person, all nuclear family members aged 35 years and older were also invited to participate. This approach allowed construction of a large family pedigree. Exclusion criteria were pregnancy at the time of recruitment, disturbances in understanding or willingness, current poly-traumas or coma, or refusal to sign the informed consent. The Moli-sani study was approved by the ethics committee of the Catholic University of Rome and is conducted under the supervision of both the Bioethics Institute of the Catholic University of Romethe and the Istituto Superiore di Sanità, Rome. All participants signed an informed consent before taking part in the study. This study was conducted in accordance with the Declaration of Helsinki.

The Rotterdam Study

The Rotterdam study is a prospective, population-based cohort study among inhabitants of Ommoord, a district of Rotterdam, The Netherlands[12]. As of 2008, the cohort is comprised of 14,926 subjects aged 45 years and older. Since 2016, it has been expanded to include particpatns aged 40 years and older. The Rotterdam study targets cardiovascular, endocrine, hepatic, neurological, ophthalmic, psychiatric, dermatological, otolaryngological, locomotor, and respiratory diseases. The participants were all examined in detail at baseline. They were interviewed at home (2 h) and then had an extensive set of examinations (totaling 5 h) in a specially built research facility in the center of the district. Written informed consent was obtained from all participants and the Medical Ethics Committee of the Erasmus Medical Center, Rotterdam, approved the study.

The Swedish Twin Registry

The Swedish Twin Registry (STR) included in this study was pulled from two national cohorts: 1) the Screening Across the Lifespan Twin Study (SALT), a telephone interview study conducted between 1998 and 2002 and including twins born before 1959 and 2) the Study of Twin Adults: Genes and Environment (STAGE), an online questionnaire study conducted between 2005 and –2006 and including twins born between 1959 and 1985[13]. Phenotypic information about hearing was collected though self-reports in these studies. Additional phenotypic information was available from a paper questionnaire (called SALTY) administered in 2009 to SALT participants born between 1944 and 1985. DNA was collected from blood or saliva and extracted by standard procedures. All genotyping

was performed by SNP&SEQ genotyping facility in Uppsala, in three waves using three different Illumina chip arrays, OmniExpress for the blood DNA, Psychchip and Global screening array for the saliva DNA samples. Written informed consents were obtained from all participants.

TwinsUK

TwinsUK is the only adult twin registry in the UK, comprising of over 12,000 healthy twin volunteers between 16 and 98 years of age[14]. Collection of data and biologic materials commenced in 1992 and is ongoing. Twins have completed detailed health and lifestyle questionnaires and attended clinical evaluations. Pure tone audiometry data was collected in a subset of the cohort (N=1,242) between April 2010 and November 2012. Participants were recruited with an aim to study aging in females. An air-conduction pure-tone audiogram was conducted by trained personnel using a Madsen XETA audiometer including TDH39 headphones. All research was conducted according to the ethical standards as defined by the Helsinki declaration. Ethical approval for this study was obtained from the National Research Ethics service London-Westminster (REC reference number: 07/H0802/84). Written informed consent was obtained from all participants prior to study conduction. Participants were excluded from analysis based on missing data, male, age less than 45 years. There were 819 female participants aged 45 years and older remaining for analysis.

UK Biobank

The UK Biobank[15] is a large prospective cohort study with over 500,000 participants aged 40 to 69 years old. Recruitment occurred between 2006 and 2010. The study collected extensive phenotype and genotype data about the participants. Data includes questionnaires, physical measures, multimodal imaging and genome-wide genotyping. Follow-up data is also available for a wide range of health-related outcomes. The UK Biobank is available for researchers worldwide. This research has been conducted using the UK Biobank Resource under application number #74463.

2. Supplementary figures

Supplementary Figure 1: Quantile-Quantile plot of association test results from the meta-analysis of tinnitus.

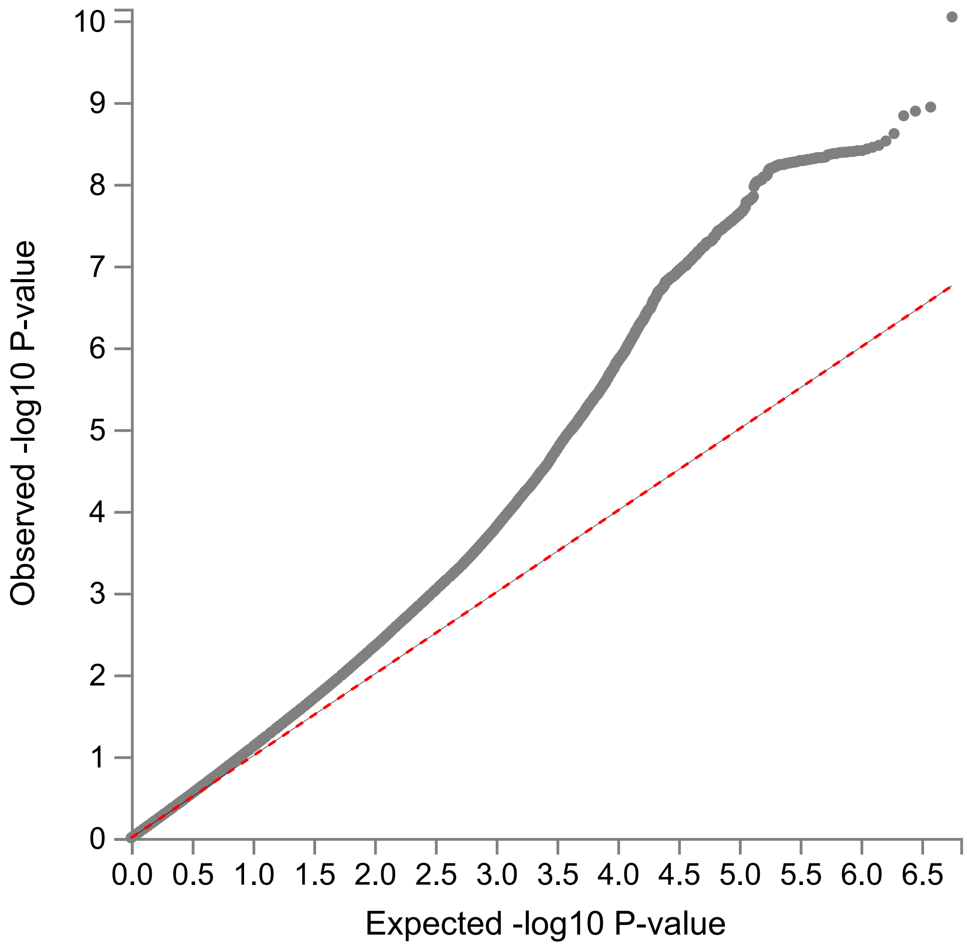

Supplementary Figure 2: Regional plots of significant and independent loci.

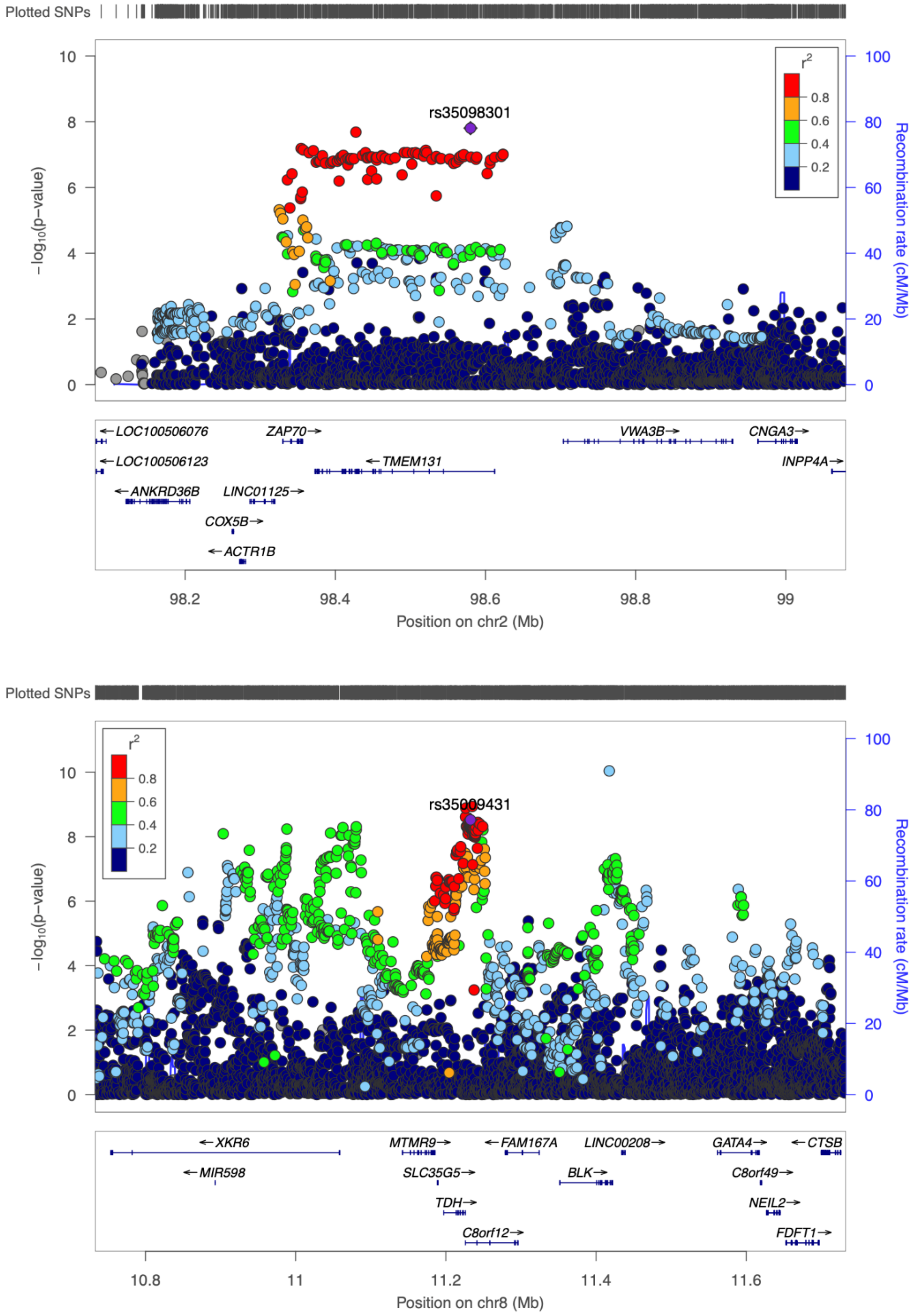

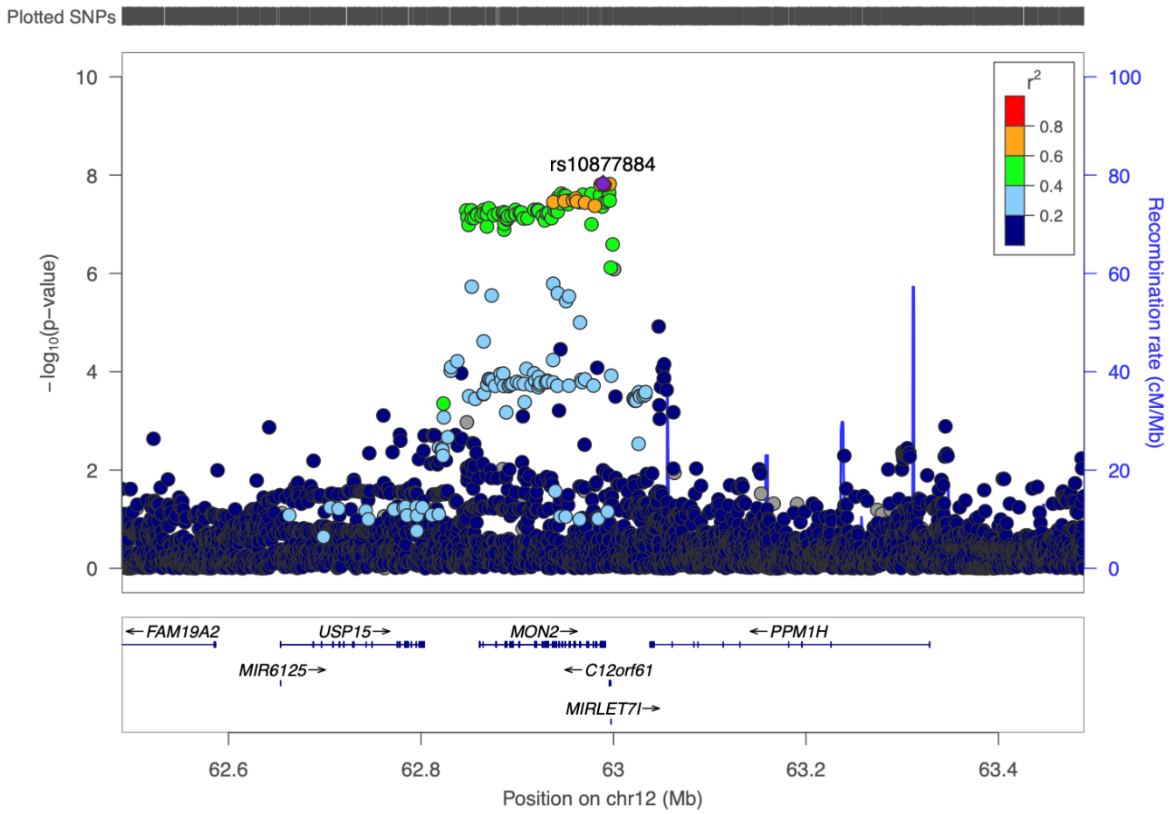
Plotted SNPs
rs10877884
r2
0.8
0.6
0.4
0.2
−log10(p−value)
Recombination rate (cM/Mb)
FAM19A2
USP15
MON2
PPM1H
MIR6125
C12orf61
MIRLET7I
Position on chr12 (Mb)

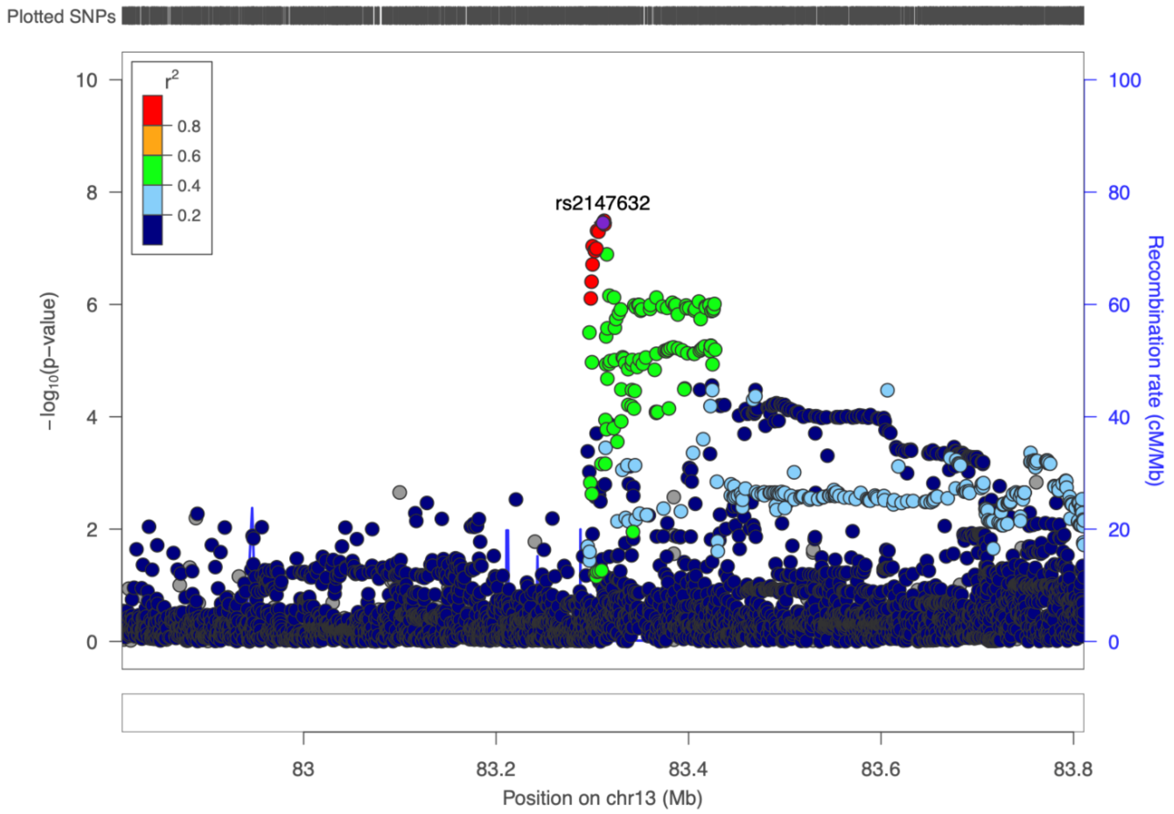
Plotted SNPs
rs2147632
r2
0.8
0.6
0.4
0.2
−log10(p−value)
Recombination rate (cM/Mb)
Position on chr13 (Mb)

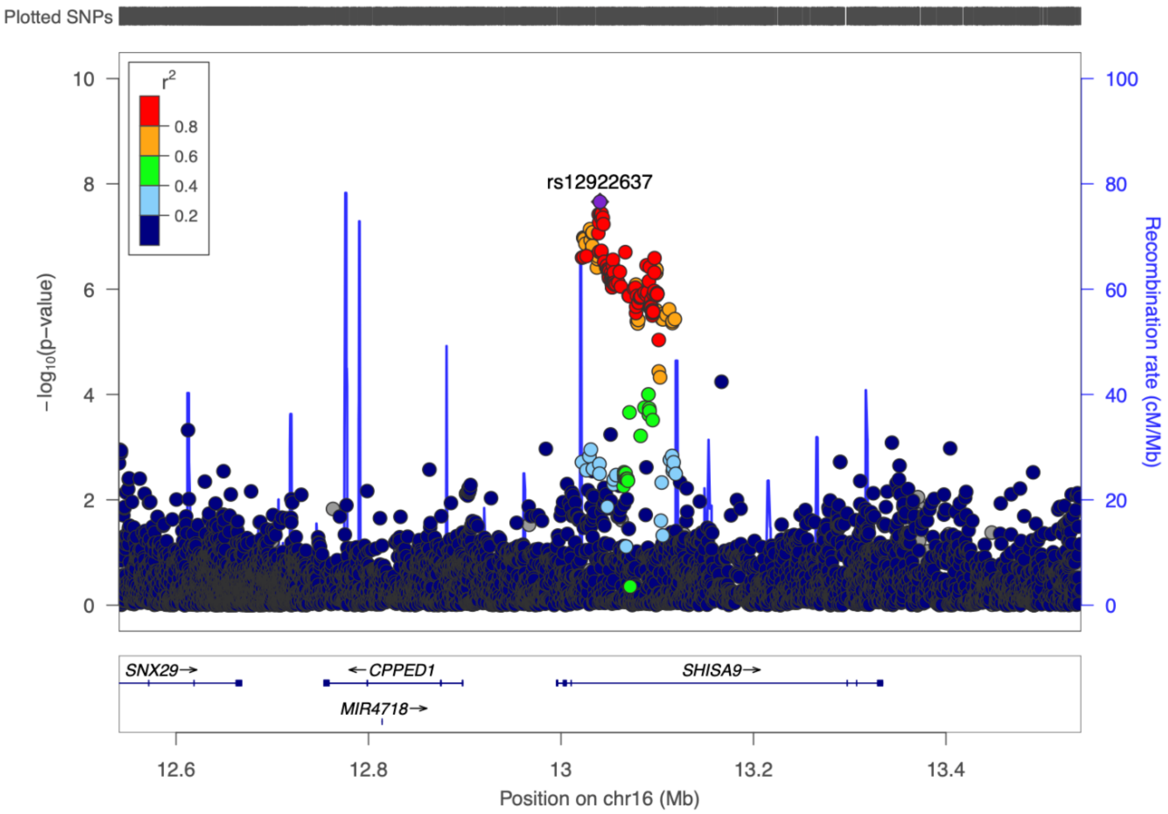

Plotted SNPs
r²
0.8
0.6
0.4
0.2
rs12922637
−log10(p−value)
Recombination rate (cM/Mb)
SNX29→
←CPPED1
MIR4718→
SHISA9→
12.6
12.8
13
13.2
13.4
Position on chr16 (Mb)

Supplementary Figure 3: Venn diagram of the different gene mapping methods. Detailed information about the mapped genes is available in **Supplementary tables S5, S6 and S7**.

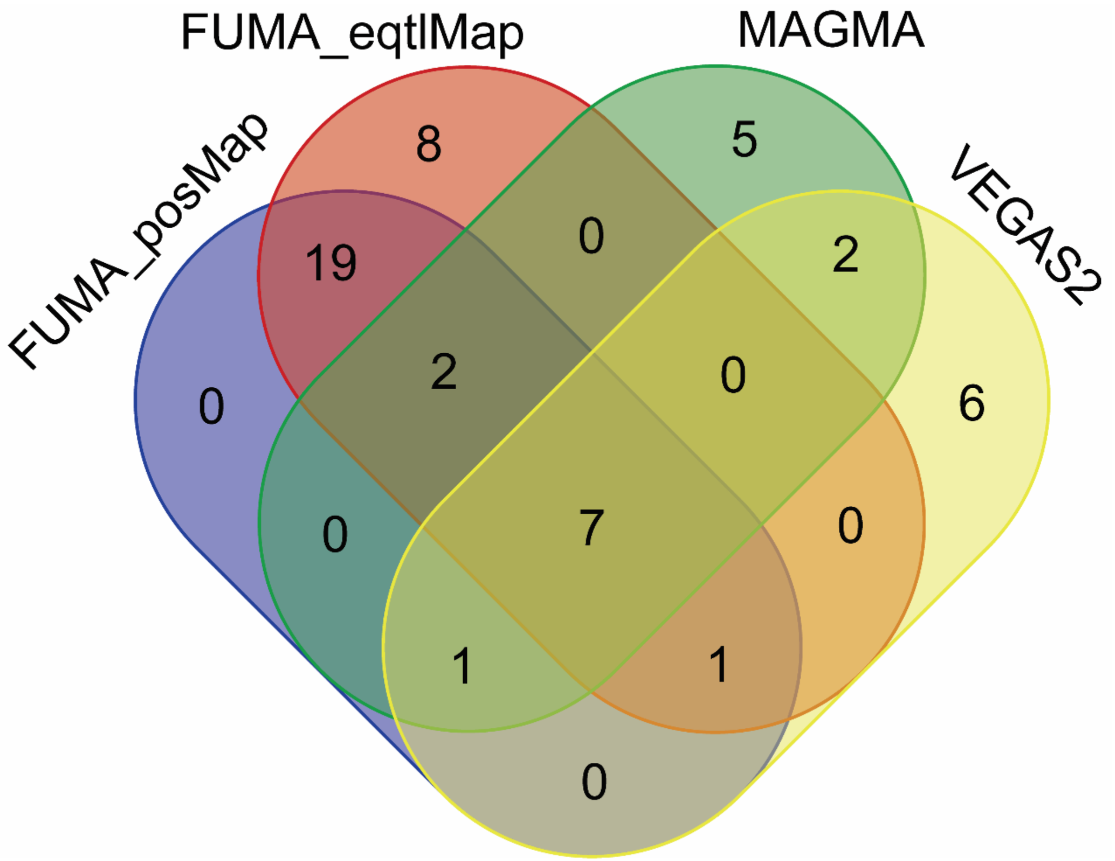

Supplementary Figure 4: Enrichment of brain cell clusters in other traits. Each panel includes results on a single supercluster. On the y-axis are the following traits: hearing loss, major depressive disorder, insomnia, neuroticism, schizophrenia and bipolar disorder (from Yao et al., 2025). On the x-axis are the –log10 p-values. An FDR < 0.1 was considered significant (red). Detailed information in **Supplementary table S10**. Abbreviations: CGE = caudal ganglionic eminence, CO = committed oligodendrocyte, CT = corticothalamic, MSN = medium spiny neuron, LLC = LAMP5-LHX6 and Chandelier, MGE = medial ganglionic eminence, ITC = intratelencephalic.

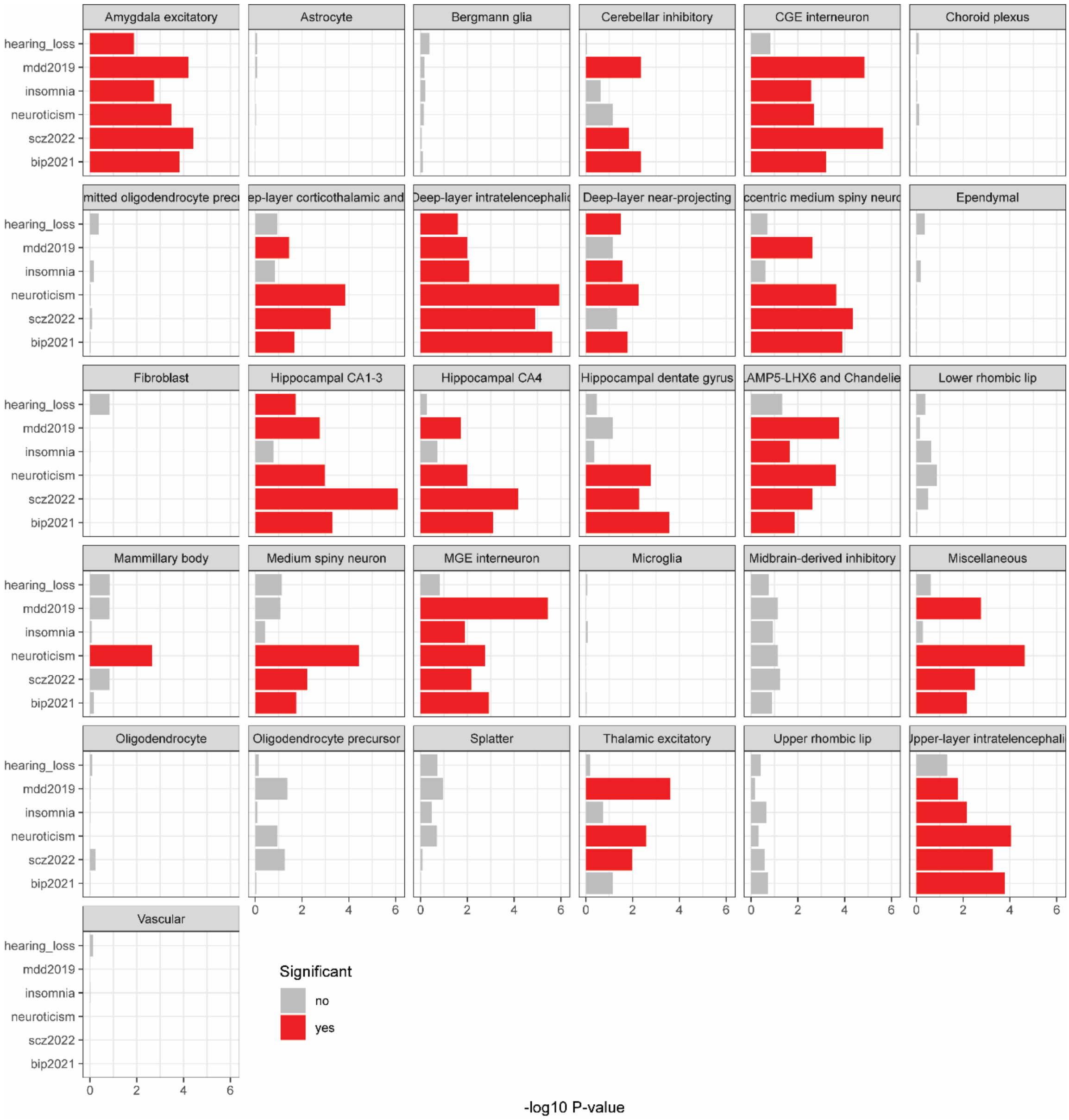

3. Supplementary Tables

Supplementary Tables Outline

Table S1	Summary of samples included in tinnitus meta-analysis
Table S2	Genome-wide significant loci for tinnitus
Table S3	Independent loci based on COJO analysis
Table S4	Leave on out meta-analysis (LL_cytosnp)
Table S5	VEGAS2 gene mapping
Table S6	Gene mapping FUMA
Table S7	MAGMA gene mapping
Table S8	Single-nucleus enrichment analysis for tinnitus.
Table S9	LD score regression genetic correlation analysis
Table S10	Single nucleus enrichment analysis on hearing loss, major depressive disorder, insomnia, neuroticism, schizophrenia and bipolar disorder

Table S1: Summary of samples included in tinnitus meta-analysis

<table>
<tr><th>Cohort study</th><th>Phenotype</th><th>Cases</th><th>Controls</th><th>N cases</th><th>N controls</th><th>Total N participants</th><th>Prevalence (%)</th><th>Array Type</th><th>Genotyping center</th><th>Genotype calling</th><th>Exclusion on SNPs used</th><th>Exclusion on a per sample</th><th>Imputation software</th><th>Imputation backbone (NBCI</th></tr>
<tr><td>AGES</td><td>Are you bothered by tinnitus (ringing in your ears)?</td><td>Yes</td><td>No</td><td>406</td><td>2728</td><td>3134</td><td>12.95</td><td>Illumina hu370CNV</td><td>National Institute of Aging genotyping center</td><td>Illumina BeadStudio</td><td>Callrate < 97%, HWE p-value < 1e-6, MAF < 1%</td><td>Callrate < 95%, sex discrepancy</td><td>MaCH</td><td>1000 Genomes phase 1 v3</td></tr>
<tr><td>Danish Twin Registry</td><td>Do you suffer from a buzzing sound in your ears?'; 'Do you suffer from tinnitus/a buzzing in your ears?'</td><td>Yes</td><td>No</td><td>137</td><td>1177</td><td>1314</td><td>10.43</td><td>Illumina Infinium PsychArray</td><td>SNP&SEQ Technology Platform, Science for Life Laboratory, Uppsala, Sweden (http://snpseq.medsci.uu.se/genotyping/snp-services/)</td><td>Illumina GenomeStudio</td><td>Call rate <98%, HWE P <10^{-6}, and MAF=0</td><td>Call rate <99%, relatedness and gender mismatch</td><td>IMPUTE2</td><td>1000G, Phase 3</td></tr>
<tr><td>Estonian Genome Center (EstBB)</td><td>ICD9 and ICD10 diagnoses of tinnitus</td><td>ICD10: H93.1</td><td>Other</td><td>8843</td><td>186551</td><td>195394</td><td>4.53</td><td>Illumina Global Screening Array</td><td>Genotyping Core Facility of the Institute of Genomics, University of Tartu</td><td>Illumina GenomeStudio</td><td>call-rate < 95%, HWE p-value < 1e-4</td><td>call-rate < 95%, mismatching sex</td><td>Beagle</td><td>Estonian population specific imputation reference</td></tr>
<tr><td>FinnGen</td><td>ICD9 and ICD10 diagnoses oftinnitus</td><td>ICD9: 3891; ICD10: H90.3, H90.4, H90.5</td><td>Other</td><td>10008</td><td>437331</td><td>447339</td><td>2.24</td><td>Several Illumina and Affymetrix FinnGen Axiom arrays</td><td>Thermo Fisher Scientific for FinnGen Axiom array, several other places for Illumina arrays</td><td>GenCall or GenCall+zCall for Illumina and AxiomGT1 for Affymetrix chip genotypes</td><td>Call rate below 98%, minor allele count below 3 and Hardy-Weinberg Equilibrium p-value lower than 1e-06</td><td>Call rate below 95%, heterozygosity exceed ±4SD from the mean, failed sex check and MDS outliers.</td><td>Eagle/Beagle</td><td>SISu v4 all-Finns reference panel</td></tr>
<tr><td>Framingham Heart Study</td><td>Do you have any ringing or buzzing in your ears?</td><td>Yes; Right / left / both</td><td>No</td><td>917</td><td>2124</td><td>3041</td><td>30.15</td><td>Affymetrix GeneChip Human Mapping 500K Array & 50K supplemental Array</td><td>Affymetrix, Inc.</td><td>Affymetrix BRLMM</td><td>MAF <1%, Call rate <97%, HWE < 10-6, Mendelian errors > 1000, not in Hapmap and/or strandedness issues merging with Hapmap</td><td>Call rate <97%, autosomal heterozygosity > ± 5SD, excessive Mendelian errors</td><td>Michigan Server</td><td>HRCr1.1</td></tr>
<tr><td>Great Age Study/Salus in Apulia Study</td><td>Do you ever hear sounds in your in your ears like ringing or other noise without an external source?</td><td>Yes</td><td>No</td><td>97</td><td>1683</td><td>1780</td><td>5.45</td><td>Illumina Infinium Global Screening Array (GSA) v1</td><td>IRCCS S. De Bellis, Castellana Grotte, Bari, Italy</td><td>Illumina GenomeStudio</td><td>Call rate < 0.97; Hardy-Weinberg P < 1*e-06; MAF < 0.01</td><td>Sample call rate < 0.97, gender check</td><td>MINIMAC v4</td><td>HRC (Build 37)</td></tr>
<tr><td>Health ABC - EUR</td><td>Buzzing/ringing in the ear?</td><td>Yes</td><td>No</td><td>406</td><td>882</td><td>1288</td><td>31.52</td><td>Illumina Human1M-Duov3_B</td><td>Johns Hopkins University Center for Inherited Disease Research (CIDR), Baltimore, MD, USA</td><td>Illumina GenomeStudio</td><td>Call rate <95%, no MAF/HWE filter</td><td>Call rate <95%, sex mismatch, excess heterozygosity F cut-off of -0.15 and <- 0.15 for inclusion, ancestry outliers removed, no relatives closer than cousin</td><td>TOPMED Imputation Server</td><td>TOPMED</td></tr>
<tr><td>Lifelines - GWAS</td><td>Do you hear ringing or whistling in your ear/ears?</td><td>Yes, always / Yes, sometimes</td><td>No, never</td><td>2210</td><td>4350</td><td>6560</td><td>33.69</td><td>Illumina HumanCytoSNP-12 v2.1</td><td>Human Genomics facility HuGeF (www.glimdna.org), Erasmus MC / Department of Genetics, UMCG</td><td>Opticall</td><td>Callrate < 99%, HWE p-value < 1e-6, MAF = 0 / Callrate < 95%, HWE p-value < 1e-4 , MAF < 1%</td><td>Sex mismatch, pedigree analysis, non-European ancestry</td><td>IMPUTE2</td><td>1000Genomes & GoNL</td></tr>
<tr><td>Lifelines - UGLI</td><td>Do you hear ringing or whistling in your ear/ears?</td><td>Yes, always / Yes, sometimes</td><td>No, never</td><td>7058</td><td>16094</td><td>23152</td><td>30.49</td><td>Infinium Global Screening Array® (GSA) MultiEthnic Disease Version 1.0</td><td>Human Genomics facility HuGeF (www.glimdna.org), Erasmus MC / Department of Genetics, UMCG</td><td>BeagleCall</td><td>Callrate < 99%, HWE p-value < 1e-6, MAF = 0 / Callrate < 95%, HWE p-value < 1e-4 , MAF < 1%</td><td>Sex mismatch, pedigree analysis, non-European ancestry</td><td>Sanger imputation service</td><td>HRC</td></tr>
<tr><td>Lifelines - UGLI2</td><td>Do you hear ringing or whistling in your ear/ears?</td><td>Yes, always / Yes, sometimes</td><td>No, never</td><td>7675</td><td>16797</td><td>24472</td><td>31.36</td><td>FinnGen Thermo Fisher Axiom® custom array</td><td>Human Genomics facility HuGeF (www.glimdna.org), Erasmus MC / Department of Genetics, UMCG</td><td>Opticall</td><td>Callrate < 99%, HWE p-value < 1e-6, MAF = 0 / Callrate < 95%, HWE p-value < 1e-4 , MAF < 1%</td><td>Sex mismatch, pedigree analysis, non-European ancestry</td><td>Sanger imputation service</td><td>HRCr1.1</td></tr>
<tr><td>Moli Sani</td><td>Have you ever suffered from tinnitus?</td><td>Yes</td><td>No</td><td>188</td><td>1010</td><td>1198</td><td>15.69</td><td>GSAMD-24v1-0_20011747_A1 (642K)/GSAMD- 24v3-0-EA_20034606_A1 (654K)</td><td>IRCSS Burlo Garofolo, Trieste, Italy</td><td>Illumina BeadStudio</td><td>Call rate <95%, HWE p-value < 1e-6, MAF>0.01</td><td>Individuals with sex discrepancy, aged less than 18 years, affected by any forms of inherited hearing loss or other diseases potentially leading to hearing defects, as well as subjects exposed to noise or ototoxic medications, were excluded from the analysis. Moreover, considering that in the study cohort some individuals are related, a as a linear mixed model was applied with GEMMA software (doi:10.1038/ng.2310)</td><td>IMPUTE2</td><td>Italian Genome Reference Panel (https://doi.org/10.1038/s41431- 019-0551-x)</td></tr>
<tr><td>Rotterdam Study - 1</td><td>Do you ever hear sounds in your head or in your ears like ringing/buzzing/peeping without an external source?</td><td>Yes, > 1 day/week | Yes, daily</td><td>Yes, < 1 day/week | No never</td><td>228</td><td>921</td><td>1149</td><td>19.84</td><td>Version 3 Illumina Infinium II HumanHap550 SNP chip array</td><td>Human Genomics facility HuGeF (www.glimdna.org), Erasmus MC</td><td>Illumina BeadStudio</td><td>Call rate <90%, no MAF/HWE filter</td><td>Call rate <97.5%, sex mismatch, excess autosomal heterozygosity > 0.336, outliers identified by the IBS clustering analysis</td><td>MACH/minimac</td><td>HRCr1.1</td></tr>
<tr><td>Rotterdam Study - 2</td><td>Do you ever hear sounds in your head or in your ears like ringing/buzzing/peeping without an external source?</td><td>Yes, > 1 day/week | Yes, daily</td><td>Yes, < 1 day/week | No never</td><td>433</td><td>1449</td><td>1882</td><td>23.01</td><td>Version 3 Illumina Infinium II HumanHap550 SNP chip array</td><td>Human Genomics facility HuGeF (www.glimdna.org), Erasmus MC</td><td>Illumina BeadStudio</td><td>Call rate <90%, no MAF/HWE filter</td><td>Call rate <97.5%, sex mismatch, excess autosomal heterozygosity > 0.336, outliers identified by the IBS clustering analysis</td><td>MACH/minimac</td><td>HRCr1.1</td></tr>
<tr><td>Rotterdam Study - 3</td><td>Do you ever hear sounds in your head or in your ears like ringing/buzzing/peeping without an external source?</td><td>Yes, > 1 day/week | Yes, daily</td><td>Yes, < 1 day/week | No never</td><td>674</td><td>2441</td><td>3115</td><td>21.64</td><td>Version 3 Illumina Infinium II HumanHap550 SNP chip array</td><td>Human Genomics facility HuGeF (www.glimdna.org), Erasmus MC</td><td>Illumina BeadStudio</td><td>Call rate <90%, no MAF/HWE filter</td><td>Call rate <97.5%, sex mismatch, excess autosomal heterozygosity > 0.336, outliers identified by the IBS clustering analysis</td><td>MACH/minimac</td><td>HRCr1.1</td></tr>
<tr><td>SALT</td><td>"Do you have buzzing in your ears?"</td><td>Both ears, "One ear",</td><td>No</td><td>1827</td><td>8866</td><td>10693</td><td>17.09</td><td>Illumina OmniExpress (HumanOmniExpress-12v1_A)</td><td>SNP&SEQ Technology Platform, Uppsala, Sweden</td><td>Illumina GenomeStudio</td><td>Call rate < 0.98, MAF=0, Hardy-Weinberg P < 1.0*10^{-10}</td><td>Call rate < 0.98, excessive heterozygosity (FHET outside +/- 0.2); sex mismatch; non-European ancestry</td><td>Sanger imputation service (EAGLE2, PBWT)</td><td>HRCr1.1</td></tr>
<tr><td>SALTY</td><td>"Do you have buzzing in your ears?"</td><td>Both ears, "One ear",</td><td>No</td><td>634</td><td>3802</td><td>4436</td><td>14.29</td><td>Illumina PsychChip (PsychChip_15048346_A)</td><td>SNP&SEQ Technology Platform, Uppsala, Sweden</td><td>Illumina GenomeStudio</td><td>Call rate < 0.98, MAF<1%, Hardy-Weinberg P < 1.0*10^{-10}</td><td>Call rate < 0.98, excessive heterozygosity (FHET outside +/- 0.2); sex mismatch; non-European ancestry</td><td>Sanger imputation service (EAGLE2, PBWT)</td><td>HRCr1.1</td></tr>
<tr><td>STAGE</td><td>"Do you have buzzing in your ears?"</td><td>Both ears, "One ear",</td><td>No</td><td>959</td><td>7351</td><td>8310</td><td>11.54</td><td>Illumina GSA (GSAMD-24v1-0_20011747_A1)</td><td>SNP&SEQ Technology Platform, Uppsala, Sweden</td><td>Illumina GenomeStudio</td><td>Call rate < 0.98, MAF<1%, Hardy-Weinberg P < 1.0*10^{-10}</td><td>Call rate < 0.98, excessive heterozygosity (FHET outside +/- 0.2); sex mismatch; non-European ancestry</td><td>Sanger imputation service (EAGLE2, PBWT)</td><td>HRCr1.1</td></tr>
<tr><td>TwinsUK</td><td>Do you suffer from Tinnitus (buzzing/ringing in the ears)?</td><td>Yes</td><td>No</td><td>632</td><td>1565</td><td>2197</td><td>28.77</td><td>Several Illumina arrays (HumanHap3001,2, HumanHap610Q, 1M-Duo and 1.2MDuo 1M)</td><td></td><td>Illumina BeadStudio</td><td>Call rate <98%</td><td>Mismatch between MZ twins and self-reported sex; non-European ancestry</td><td>IMPUTE2</td><td>HRCr1.1</td></tr>
<tr><td>UK Biobank</td><td>Do you get or have you had noises (such as ringing or buzzing) in your head or in one or both ears that lasts for more than five minutes at a time?</td><td>Yes, not most or all of the time / Yes, now a lot of the time / Yes, now some of the time/Yes, but not now, but have in the past</td><td>No, never</td><td>31918</td><td>115842</td><td>147760</td><td>21.60</td><td>UK Biobank Affymetrix Axiom and UK BiLEVE Affymetrix Axiom array</td><td>Affymetrix Research Services Laboratory, Santa Clara, California, USA</td><td>Affymetrix BRLMM</td><td>Multiple criteria as outlined here https://biobank.ctsu.ox.ac.uk/crystal/crystal/docs/genotyping_qc.pdf</td><td>Multiple criteria as outlined here https://biobank.ctsu.ox.ac.uk/crystal/crystal/docs/genotyping_qc.pdf</td><td>IMPUTE2</td><td>HRC/1000Genomes</td></tr>
</table>

Abbreviations: ICD = International Classification of Diseases; MAF = minor allele frequency; HWE = Hardy-Weinberg equilibrium

Genome Build	*Data handling and*	*Lambda GC*	*Age (mean)*	*Age (sd)*
GRCh37/Hg19	PLINKv1.9, ProbAbel	1.018316631	76.35	5.4
GRCh37/Hg19	Plink, MLMA	0.9888482	63.7	10.7
GRCh37/Hg19	regenie	1.046705	53.96	16.91
GRCh38/Hg38	REGENIE	1.0522919	59.37	17.80
GRCh37/Hg19	R, LME	0.9953424	63.64	10.96
GRCh37/Hg19	GEMMA	0.9916276	73.65	6.8
GRCh38/Hg38	PLINK, Rvtests	1.0197357	77.59	2.83
GRCh37/Hg19	regenie	1.0359197	45.89	10.87
GRCh37/Hg19	regenie	1.0411959	42.85	14.30
GRCh37/Hg19	regenie	1.0460095	43.62	13.56
GRCh37/Hg19	Gemma 0.98.4	1.0278043	64.32	9.34
GRCh37/Hg19	PLINK, Mach2DAT	1.0464918	83.37	4.42
GRCh37/Hg19	PLINK, Mach2DAT	1.0032698	76.15	5.38
GRCh37/Hg19	PLINK, Mach2DAT	1.0051417	62.08	6.22
GRCh37/Hg19	R, BOLT-LMM	1	58.6	8.01
GRCh37/Hg19	R, BOLT-LMM	1	49	4.2
GRCh37/Hg19	R, BOLT-LMM	1.0474568	37.39	7.7
GRCh37/Hg19	GEMMA	1	60.59	13.51
GRCh37/Hg19	R, BOLT-LMM	1.047456849	59.59	8.03

Table S2: Genome-wide significant loci for tinnitus

chromosome:position	*other_allele*	*reference_allele*	*eaf*	*beta*	*se*	*p-value*	*i2*
8:11417582	T	C	0.625042	0.010087	0.001555	9.09e-11	0.495412
8:11235150	C	A	0.571633	-0.00851	0.001397	1.15e-9	0.371722
8:11226456	C	A	0.572631	-0.007853	0.001293	1.29e-9	0.40946
8:10147398	C	T	0.4776	-0.007824	0.001293	1.47e-9	0.242871
8:11226071	A	G	0.566381	-0.007704	0.00129	2.43e-9	0.417118
8:11232788	G	A	0.571613	-0.007669	0.001292	2.99e-9	0.430868
8:11231249	T	C	0.438687	0.007613	0.001287	3.38e-9	0.402019
8:11242632	G	A	0.570911	-0.007632	0.001292	3.57e-9	0.441026
8:11233318	A	C	0.565947	-0.007603	0.001289	3.74e-9	0.400003
8:11236681	T	G	0.570545	-0.007595	0.001289	3.92e-9	0.388482
8:11236685	C	T	0.57049	-0.007594	0.001289	3.93e-9	0.388926
8:11236419	T	C	0.571055	-0.007605	0.001292	4.02e-9	·2240000000
8:11245303	C	G	0.43391	0.007598	0.001291	4.04e-9	0.397528
8:11236413	C	T	0.571058	-0.007601	0.001292	4.1e-9	0.406257
8:11236572	T	A	0.570387	-0.00758	0.001288	4.12e-9	0.396311
8:11230574	G	A	0.571246	-0.007598	0.001292	4.15e-9	0.415038
8:11237477	A	G	0.570758	-0.007577	0.001289	4.26e-9	0.376905
8:11237480	A	G	0.570752	-0.007577	0.001289	4.26e-9	0.378108
8:11233419	T	G	0.565861	-0.007571	0.001289	4.35e-9	0.400638
8:11231886	A	G	0.565663	-0.007569	0.001289	4.41e-9	0.42393
8:11236392	T	C	0.571097	-0.007572	0.001292	4.7e-9	0.402008
8:11227406	G	A	0.570914	-0.007569	0.001292	4.75e-9	0.411774
8:11239762	A	T	0.565919	-0.007547	0.001288	4.78e-9	0.391425
8:11249010	G	T	0.433776	0.007569	0.001292	4.78e-9	0.416452
8:11232860	G	A	0.57142	-0.007565	0.001291	4.79e-9	0.44514
8:11080675	C	T	0.540333	-0.007576	0.001294	4.9e-9	0.458732
8:11227885	C	T	0.571377	-0.007563	0.001292	4.9e-9	0.424581
8:11228100	G	A	0.570934	-0.007556	0.001291	4.98e-9	0.413246
8:11234500	A	T	0.56559	-0.007536	0.001288	5.04e-9	0.410002
8:11234520	A	G	0.570556	-0.007548	0.00129	5.04e-9	0.425985
8:11234885	C	G	0.570534	-0.007541	0.00129	5.12e-9	0.424282
8:11233582	A	G	0.565869	-0.007535	0.001289	5.16e-9	0.398733
8:11233659	A	G	0.56587	-0.007534	0.001289	5.18e-9	0.398444

chromosome:position	*other_allele*	*reference_allele*	*eaf*	*beta*	*se*	*p-value*	*i2*
8:11234298	C	T	0.57061	-0.007542	0.00129	5.18e-9	0.419525
8:11062882	T	C	0.536661	-0.007525	0.001287	5.19e-9	0.482151
8:11234844	G	A	0.570568	-0.007537	0.00129	5.31e-9	0.423366
8:11233958	A	G	0.570682	-0.007539	0.001291	5.37e-9	0.434524
8:11236975	A	G	0.570495	-0.007528	0.001289	5.4e-9	0.417938
8:11232343	C	G	0.570595	-0.007538	0.001291	5.42e-9	0.442346
8:11234780	C	G	0.570645	-0.007531	0.00129	5.47e-9	0.423597
8:11230206	G	T	0.571167	-0.007537	0.001292	5.51e-9	0.412341
8:11233917	C	T	0.570684	-0.007534	0.001291	5.51e-9	0.434969
8:11234367	T	G	0.570014	-0.007527	0.00129	5.57e-9	0.416627
8:11235360	A	C	0.570631	-0.007524	0.00129	5.59e-9	0.429098
8:11234626	T	C	0.570736	-0.00753	0.001291	5.61e-9	0.42357
8:11235497	G	A	0.570452	-0.007514	0.001289	5.7e-9	0.396491
8:11234613	T	A	0.570732	-0.007523	0.001291	5.78e-9	0.42366
8:10988275	A	G	0.573603	-0.008199	0.001407	5.79e-9	0.506764
8:11230259	A	T	0.571117	-0.007527	0.001292	5.79e-9	0.414148
8:11236850	C	T	0.570474	-0.007503	0.001288	5.81e-9	0.390934
8:11236964	C	T	0.570695	-0.007504	0.001288	5.81e-9	0.387832
8:11235735	T	C	0.570475	-0.007512	0.00129	5.85e-9	0.426266
8:11229319	C	G	0.571402	-0.007531	0.001293	5.9e-9	0.425943
8:11080665	T	A	0.540221	-0.00753	0.001294	6.04e-9	0.461293
8:11235136	C	G	0.570659	-0.00751	0.00129	6.04e-9	0.426922
8:11249261	T	C	0.375683	0.008415	0.001447	6.14e-9	0.266491
8:11243126	C	G	0.575479	-0.007524	0.001294	6.27e-9	0.475733
8:11236809	T	C	0.570332	-0.007484	0.001288	6.35e-9	0.39368
8:11238029	C	T	0.575425	-0.007501	0.001291	6.4e-9	0.469878
8:11239640	C	T	0.575231	-0.00751	0.001293	6.44e-9	0.463109
8:11231354	G	T	0.439329	0.007478	0.001287	6.46e-9	0.406758
8:11235605	T	G	0.570596	-0.007492	0.00129	6.55e-9	0.396929
8:11235393	A	G	0.570522	-0.007485	0.00129	6.65e-9	0.428798
8:11235579	T	G	0.570134	-0.007475	0.00129	7.06e-9	0.391376
8:11046394	C	G	0.600558	-0.008162	0.001412	7.69e-9	0.406279
8:11239510	G	A	0.575036	-0.00746	0.001292	7.93e-9	0.466552
8:11239565	C	T	0.575036	-0.007459	0.001292	7.99e-9	0.46584

chromosome:position	*other_allele*	*reference_allele*	*eaf*	*beta*	*se*	*p-value*	*i2*
8:10903475	T	A	0.514128	-0.007448	0.001291	8.09e-9	0.547415
8:11239352	G	A	0.575032	-0.00745	0.001291	8.18e-9	0.467694
8:11229638	C	T	0.570525	-0.007456	0.001293	8.2e-9	0.444053
8:8700851	C	T	0.514172	0.007429	0.001289	8.41e-9	0.509995
8:11239137	G	A	0.574869	-0.007434	0.001291	8.81e-9	’1680000000
8:11238597	C	T	0.575054	-0.007428	0.001291	8.92e-9	0.462827
8:11061792	C	T	0.537656	-0.007402	0.001287	8.98e-9	0.486205
8:11060311	A	C	0.537547	-0.007402	0.001287	9.05e-9	0.476986
8:11071057	A	G	0.590803	-0.007484	0.001301	9.1e-9	0.537626
8:11237773	T	C	0.574638	-0.007413	0.00129	9.23e-9	0.466364
8:11235910	G	A	0.574394	-0.007423	0.001292	9.37e-9	0.458493
8:8646246	T	C	0.511483	0.007416	0.001291	9.37e-9	0.447765
8:11239078	T	G	0.575111	-0.007419	0.001291	9.43e-9	0.468722
8:11237756	C	A	0.57441	-0.007376	0.001285	9.75e-9	0.468782
8:11239017	T	A	0.575136	-0.007409	0.001291	9.87e-9	0.46476
8:11239054	C	T	0.575157	-0.007403	0.001292	1.03e-8	0.467051
8:11079367	G	A	0.533126	-0.007379	0.00129	1.08e-8	0.498061
8:11078949	G	A	0.533222	-0.007319	0.00129	1.42e-8	0.507733
8:11247814	G	A	0.378427	0.007548	0.001331	1.46e-8	0.357556
12:62989110	A	C	0.496578	-0.00726	0.001281	1.5e-8	0.543745
8:8633548	T	G	0.498942	-0.007318	0.001292	1.51e-8	0.465781
12:62995984	C	T	0.672148	-0.008214	0.00145	1.52e-8	0.4616
12:62986620	G	A	0.668893	-0.008176	0.001445	1.57e-8	0.45937
2:98580012	T	C	0.489779	0.008074	0.001427	1.57e-8	0.344741
12:62990403	A	G	0.496361	-0.007246	0.001281	1.6e-8	0.544762
12:62990415	T	C	0.496356	-0.007244	0.001281	1.61e-8	0.545017
8:8664622	G	A	0.524052	0.007302	0.001292	1.61e-8	0.33436
8:11076635	T	G	0.531667	-0.007286	0.001289	1.63e-8	0.517329
8:8661534	T	C	0.508581	0.007262	0.001286	1.65e-8	0.461778
8:8652889	G	A	0.508531	0.007269	0.001287	1.67e-8	0.461896
8:11078781	A	C	0.531726	-0.007282	0.00129	1.68e-8	0.504546
8:8661681	C	G	0.512504	0.007243	0.001288	1.92e-8	0.441258
8:8685854	A	G	0.510463	0.007265	0.001292	1.92e-8	0.45352
8:8641145	C	T	0.500305	0.007248	0.001291	2.01e-8	0.43898

chromosome:position	***other_allele***	***reference_allele***	***eaf***	***beta***	***se***	***p-value***	***i2***
8:11219781	C	T	0.431441	0.007242	0.00129	2.03e-8	0.400284
2:98427382	T	C	0.490657	0.008	0.001426	2.07e-8	0.413012
8:8637429	G	A	0.499762	-0.007226	0.00129	2.15e-8	0.450121
8:8721473	G	A	0.530325	0.007253	0.001295	2.18e-8	0.52241
16:13040514	C	T	0.680505	-0.008169	0.001459	2.19e-8	0.00
8:10987553	T	C	0.573115	-0.007267	0.001298	2.2e-8	0.477455
12:62995340	G	A	0.672257	-0.007497	0.001339	2.23e-8	0.394087
8:8770512	C	T	0.575732	0.007282	0.001302	2.27e-8	0.34965
8:11242025	T	G	0.537939	-0.007269	0.001299	2.28e-8	0.419451
8:8678530	G	A	0.521683	0.007207	0.00129	2.37e-8	0.398588
12:62945970	C	G	0.672258	-0.007478	0.001339	2.38e-8	0.407387
8:11069960	C	T	0.550512	-0.007195	0.001288	2.4e-8	0.52078
12:62977163	G	A	0.672258	-0.007489	0.001341	2.41e-8	0.38373
12:62995269	A	G	0.67221	-0.007479	0.001339	2.41e-8	0.382106
8:11251175	A	G	0.441543	0.007353	0.001317	2.43e-8	0.400674
12:62968280	T	C	0.672234	-0.007464	0.001338	2.5e-8	0.393524
8:11215617	C	G	0.430443	0.007177	0.001288	2.55e-8	0.386663
12:62948736	G	A	0.672367	-0.007468	0.00134	2.58e-8	0.416193
12:62985871	C	A	0.672227	-0.007461	0.001339	2.59e-8	0.397868
8:10938260	G	A	0.519837	-0.007176	0.001289	2.63e-8	0.528811
8:10987651	T	G	0.573041	-0.007225	0.001298	2.65e-8	0.485532
12:62950556	G	C	0.672318	-0.007452	0.001339	2.66e-8	0.407056
8:11055597	C	A	0.545133	-0.007165	0.001287	2.67e-8	0.416053
8:8685646	T	C	0.510169	0.007186	0.001291	2.69e-8	0.488867
8:8673601	A	C	0.517319	0.007168	0.001289	2.76e-8	0.40252
8:11214972	A	G	0.469705	0.007146	0.001286	2.8e-8	0.338936
12:62942258	C	T	0.672489	-0.007438	0.001339	2.83e-8	0.419595
8:8639740	A	G	0.499759	-0.007166	0.00129	2.85e-8	0.466298
8:11219334	G	A	0.431212	0.007168	0.001291	2.87e-8	0.440499
8:8704330	G	C	0.519017	0.007156	0.001289	2.87e-8	0.507764
8:11214455	A	G	0.469998	0.00714	0.001286	2.89e-8	0.313706
8:8682192	A	T	0.521578	0.007164	0.001291	2.91e-8	0.382837
8:8679614	T	C	0.521505	0.00716	0.00129	2.92e-8	0.382412
12:62960880	A	G	0.668954	-0.007405	0.001335	2.98e-8	0.413649

chromosome:position	***other_allele***	***reference_allele***	***eaf***	***beta***	***se***	***p-value***	***i2***
8:8675325	A	T	0.512892	0.007152	0.00129	3e-8	0.461198
12:62961299	C	T	0.672377	-0.007425	0.00134	3.05e-8	0.401325
8:10987199	T	C	0.573024	-0.007183	0.001296	3.08e-8	0.450852
8:8684953	G	A	0.521055	0.007165	0.001293	3.09e-8	0.388777
8:8660538	C	A	0.518034	0.007136	0.001289	3.14e-8	0.445874
8:8673736	T	C	0.518244	0.007136	0.001289	3.14e-8	0.413675
8:11225168	G	A	0.522452	-0.007119	0.001286	3.18e-8	0.313122
13:83312341	C	T	0.598337	-0.007206	0.001303	3.23e-8	0.00
8:8730488	G	A	0.526933	0.007178	0.001297	3.24e-8	0.521281
12:62957906	G	C	0.668926	-0.007389	0.001336	3.25e-8	0.420553
8:11225910	A	G	0.521744	-0.007125	0.001288	3.27e-8	0.353716
12:62996061	G	A	0.672265	-0.007427	0.001343	3.29e-8	0.4224
12:62993793	G	A	0.669237	-0.007428	0.001344	3.31e-8	0.427323
12:62952696	C	T	0.672222	-0.007403	0.001339	3.33e-8	0.404831
12:62949110	A	C	0.669021	-0.007376	0.001335	3.38e-8	0.421368
12:62961801	G	A	0.669002	-0.007377	0.001336	3.42e-8	0.417933
12:62975307	C	T	0.672225	-0.007408	0.001342	3.48e-8	0.422277
12:62937532	C	T	0.66618	-0.007354	0.001333	3.52e-8	0.516077
13:83311141	G	A	0.598452	-0.007179	0.001301	3.52e-8	0.00
8:8679176	A	G	0.521558	0.007118	0.00129	3.52e-8	0.394142
12:62990871	C	A	0.672166	-0.007389	0.00134	3.56e-8	0.399641
12:62964552	T	C	0.66114	-0.007429	0.001347	3.57e-8	0.441951
12:62970482	A	G	0.668985	-0.007376	0.001338	3.63e-8	0.441969
16:13041027	G	T	0.680643	-0.008021	0.001456	3.66e-8	0.00
16:13042097	G	A	0.680702	-0.00802	0.001456	3.66e-8	0.00
8:11225480	T	C	0.521222	-0.007088	0.001286	3.66e-8	0.311814
8:8680477	G	A	0.521516	0.007109	0.00129	3.66e-8	0.376871
8:8682878	T	C	0.51108	0.007108	0.00129	3.68e-8	0.509731
12:62945245	G	A	0.672347	-0.007386	0.001341	3.73e-8	0.428268
13:83312717	G	C	0.597904	-0.007177	0.001303	3.74e-8	0.00
8:11213589	T	G	0.430584	0.007102	0.00129	3.74e-8	0.430743
16:13039154	C	T	0.679454	-0.008713	0.001583	3.79e-8	0.00
8:9569104	T	C	0.820086	0.009116	0.001657	3.84e-8	0.00
16:13040889	C	G	0.680586	-0.008004	0.001455	3.86e-8	0.00

chromosome:position	***other_allele***	***reference_allele***	***eaf***	***beta***	***se***	***p-value***	***i2***
12:62952910	C	T	0.672283	-0.007379	0.001342	3.9e-8	0.435652
8:11070360	C	G	0.546344	-0.007068	0.001286	3.98e-8	0.511645
8:11045161	G	A	0.531312	-0.007055	0.001285	4.08e-8	0.471785
8:11228672	G	A	0.477972	0.00705	0.001285	4.16e-8	0.34481
8:11054097	C	A	0.548969	-0.007068	0.001288	4.17e-8	0.415959
12:62980580	T	A	0.668903	-0.007328	0.001336	4.22e-8	0.40879
8:11046209	T	C	0.599392	-0.007142	0.001303	4.37e-8	0.460309
12:62988288	G	A	0.672277	-0.007354	0.001342	4.38e-8	0.430955
16:13043452	A	G	0.680533	-0.007971	0.001455	4.41e-8	0.00
8:10986837	T	G	0.572897	-0.007109	0.001298	4.41e-8	0.497053
16:13041924	A	G	0.680633	-0.007968	0.001455	4.44e-8	0.00
8:11041642	A	T	0.529979	-0.007042	0.001286	4.46e-8	0.466136
8:11252425	C	A	0.506025	0.00745	0.001361	4.46e-8	0.389993
8:8644274	G	C	0.512592	0.007074	0.001292	4.53e-8	0.44873
12:62870574	C	G	0.667857	-0.007274	0.001331	4.74e-8	0.433408
8:11425809	T	G	0.536588	-0.007038	0.001288	4.79e-8	0.508878
8:8685190	A	G	0.520781	0.00706	0.001292	4.81e-8	0.385978
13:83304898	T	G	0.5985	-0.007095	0.001299	4.87e-8	0.00
8:8654527	T	C	0.515835	0.007035	0.001289	4.91e-8	0.400242
12:62941837	A	G	0.671432	-0.007297	0.001337	4.93e-8	0.441654
8:11039816	T	G	0.527512	-0.007028	0.001288	4.94e-8	0.478889
12:62941426	T	C	0.671224	-0.007294	0.001337	4.97e-8	0.43931

Sorted by significance. Abbreviations: eaf = effect allele frequency; se = standard error

Table S2: Genome-wide s

chromosome:position	*n_studies*	*n_samples*
8:11417582	6	821622
8:11235150	15	887212
8:11226456	18	911943
8:10147398	18	911943
8:11226071	18	911943
8:11232788	18	911943
8:11231249	18	911943
8:11242632	18	911943
8:11233318	18	911943
8:11236681	19	913247
8:11236685	19	913247
8:11236419	19	913246
8:11245303	18	911943
8:11236413	19	913246
8:11236572	19	913247
8:11230574	18	911943
8:11237477	19	913247
8:11237480	19	913247
8:11233419	18	911943
8:11231886	18	911943
8:11236392	19	913246
8:11227406	18	911943
8:11239762	18	911943
8:11249010	18	911943
8:11232860	18	911943
8:11080675	18	911943
8:11227885	18	911943
8:11228100	18	911943
8:11234500	18	911943
8:11234520	18	911943
8:11234885	18	911943
8:11233582	18	911943
8:11233659	18	911943

chromosome:position	*n_studies*	*n_samples*
8:11234298	18	911943
8:11062882	19	913242
8:11234844	18	911943
8:11233958	18	911943
8:11236975	18	911969
8:11232343	18	911943
8:11234780	18	911943
8:11230206	18	911943
8:11233917	18	911943
8:11234367	18	911943
8:11235360	18	911943
8:11234626	18	911943
8:11235497	19	913238
8:11234613	18	911943
8:10988275	14	885924
8:11230259	18	911943
8:11236850	19	913247
8:11236964	19	913243
8:11235735	18	911943
8:11229319	18	911943
8:11080665	18	911943
8:11235136	18	911943
8:11249261	15	887212
8:11243126	18	911943
8:11236809	19	913246
8:11238029	18	911943
8:11239640	18	911943
8:11231354	18	911943
8:11235605	19	913240
8:11235393	18	911943
8:11235579	19	913239
8:11046394	15	887212
8:11239510	18	911943
8:11239565	18	911943

chromosome:position	***n_studies***	***n_samples***
8:10903475	17	910655
8:11239352	18	911943
8:11229638	17	910655
8:8700851	18	911943
8:11239137	18	911943
8:11238597	18	911943
8:11061792	19	913246
8:11060311	19	913243
8:11071057	17	910655
8:11237773	18	911943
8:11235910	18	911943
8:8646246	17	910655
8:11239078	18	911943
8:11237756	18	911943
8:11239017	18	911943
8:11239054	18	911943
8:11079367	18	911943
8:11078949	18	911943
8:11247814	18	911943
12:62989110	19	913254
8:8633548	18	911943
12:62995984	14	885924
12:62986620	15	887238
2:98580012	16	888525
12:62990403	19	913254
12:62990415	19	913254
8:8664622	17	910655
8:11076635	17	910655
8:8661534	19	913244
8:8652889	19	913236
8:11078781	18	911943
8:8661681	18	911943
8:8685854	17	910655
8:8641145	17	910655

chromosome:position	*n_studies*	*n_samples*
8:11219781	18	911943
2:98427382	15	887234
8:8637429	18	911943
8:8721473	18	911943
16:13040514	17	910655
8:10987553	17	910655
12:62995340	19	913254
8:8770512	18	911943
8:11242025	17	438262
8:8678530	19	913239
12:62945970	19	913256
8:11069960	18	911969
12:62977163	19	913247
12:62995269	19	913255
8:11251175	16	715261
12:62968280	19	913255
8:11215617	18	911943
12:62948736	19	913256
12:62985871	19	913254
8:10938260	17	910655
8:10987651	17	910655
12:62950556	19	913256
8:11055597	18	911943
8:8685646	18	911943
8:8673601	18	911969
8:11214972	18	911943
12:62942258	19	913256
8:8639740	18	911943
8:11219334	17	910655
8:8704330	18	911943
8:11214455	18	911943
8:8682192	19	913236
8:8679614	19	913237
12:62960880	19	913255

chromosome:position	*n_studies*	*n_samples*
8:8675325	18	911943
12:62961299	19	913256
8:10987199	18	911943
8:8684953	18	911943
8:8660538	18	911943
8:8673736	19	913246
8:11225168	18	911943
13:83312341	18	911943
8:8730488	18	911943
12:62957906	19	913255
8:11225910	17	910655
12:62996061	18	911966
12:62993793	18	717861
12:62952696	19	913257
12:62949110	19	913256
12:62961801	19	913256
12:62975307	18	911967
12:62937532	18	911943
13:83311141	18	911943
8:8679176	19	913240
12:62990871	19	913255
12:62964552	17	438286
12:62970482	18	911967
16:13041027	18	911943
16:13042097	18	911943
8:11225480	18	911943
8:8680477	19	913236
8:8682878	18	911943
12:62945245	18	911969
13:83312717	18	911943
8:11213589	17	910655
16:13039154	15	887212
8:9569104	19	913239
16:13040889	18	911943

chromosome:position	*n_studies*	*n_samples*
12:62952910	18	911968
8:11070360	19	913241
8:11045161	19	913257
8:11228672	18	911943
8:11054097	19	913245
12:62980580	19	913255
8:11046209	18	911943
12:62988288	18	911967
16:13043452	18	911943
8:10986837	17	910655
16:13041924	18	911943
8:11041642	18	911943
8:11252425	18	911943
8:8644274	18	911943
12:62870574	19	913254
8:11425809	19	913234
8:8685190	18	911943
13:83304898	18	911943
8:8654527	18	911943
12:62941837	19	913249
8:11039816	17	910655
12:62941426	19	913250

Sorted by significance. Ab

Table S3 - lead SNPs based on COJO analysis

Chr	*SNP*	*bp*	*refA*	*freq*	*b*	*se*	*p*	*n*	*freq_geno*	*bJ*	*bJ_se*	*pJ*	*LD_r*
8	rs35009431	11232788	G	0.571613	-0.007669	0.001292	2.92E-09	499184	0.55183	-0.007669	0.001292	2.93E-09	0
12	rs10877884	62989110	A	0.496578	-0.00726	0.001281	1.45E-08	497403	0.527415	-0.00726	0.001281	1.45E-08	0
2	rs35098301	98580012	T	0.489779	0.008074	0.001427	1.53E-08	400971	0.606143	0.008074	0.0014271	1.53E-08	0
16	rs12922637	13040514	C	0.680505	-0.008169	0.001459	2.16E-08	440877	0.744475	-0.008169	0.0014591	2.16E-08	0
13	rs2147632	83311141	G	0.598452	-0.007179	0.001301	3.43E-08	501657	0.578845	-0.007179	0.001301	3.43E-08	0

Sorted by significance. Abbreviations: Chr = chromosome; SNP = single nucleotide polymorphism; bp = base position;refA = reference allele; freq = frequency effect allele; b = beta; se = standard error; p = p-value; n = number; freq_geno = frequency reference dataset; bJ = effect size joint analysis; bJ_se = standard error joint analysis; pJ = p-value joint analysis; LD_r = LD correlation between SNPs

Table S4: Leave-one-out meta-analysis (LL_cytosnp)

chromosome:position	*beta*	*beta_LL_cytoSNP_out*	*p-value*	*p-value_LL_cytoSNP_out*	*i2*
2:98580012	0.008074	0.008075	1.57E-08	1.60E-08	0.344741
2:98427382	0.008	0.007995	2.07E-08	2.16E-08	0.413012
8:11417582	0.010087	0.010087	9.09E-11	9.09E-11	0.495412
8:11235150	-0.00851	-0.008543	1.15E-09	9.99E-10	0.371722
8:11226456	-0.007853	-0.007882	1.29E-09	1.13E-09	0.40946
8:10147398	-0.007824	-0.007852	1.47E-09	1.30E-09	0.242871
8:11226071	-0.007704	-0.007733	2.43E-09	2.13E-09	0.417118
8:11232788	-0.007669	-0.007699	2.99E-09	2.62E-09	0.430868
8:11231249	0.007613	0.007639	3.38E-09	3.02E-09	0.402019
8:11242632	-0.007632	-0.007653	3.57E-09	3.26E-09	0.441026
8:11233318	-0.007603	-0.007638	3.74E-09	3.20E-09	0.400003
8:11236681	-0.007595	-0.007623	3.92E-09	3.46E-09	0.388482
8:11236685	-0.007594	-0.007622	3.93E-09	3.47E-09	0.388926
8:11236419	-0.007605	-0.007633	4.02E-09	3.55E-09	0.404224
8:11245303	0.007598	0.007624	4.04E-09	3.61E-09	0.397528
8:11236413	-0.007601	-0.007629	4.10E-09	3.63E-09	0.406257
8:11236572	-0.00758	-0.00761	4.12E-09	3.61E-09	0.396311
8:11230574	-0.007598	-0.007628	4.15E-09	3.64E-09	0.415038
8:11237477	-0.007577	-0.007605	4.26E-09	3.76E-09	0.376905
8:11237480	-0.007577	-0.007606	4.26E-09	3.75E-09	0.378108
8:11233419	-0.007571	-0.007606	4.35E-09	3.72E-09	0.400638
8:11231886	-0.007569	-0.007594	4.41E-09	3.96E-09	0.42393
8:11236392	-0.007572	-0.007601	4.70E-09	4.13E-09	0.402008
8:11227406	-0.007569	-0.007591	4.75E-09	4.33E-09	0.411774
8:11239762	-0.007547	-0.007574	4.78E-09	4.25E-09	0.391425
8:11249010	0.007569	0.007602	4.78E-09	4.13E-09	0.416452
8:11232860	-0.007565	-0.00759	4.79E-09	4.30E-09	0.44514
8:11080675	-0.007576	-0.007582	4.90E-09	4.80E-09	0.458732
8:11227885	-0.007563	-0.007593	4.90E-09	4.30E-09	0.424581
8:11228100	-0.007556	-0.007579	4.98E-09	4.51E-09	0.413246

chromosome:position	*beta*	*beta_LL_cytoSNP_out*	*p-value*	*p-value_LL_cytoSNP_out*	*i2*
8:11234500	-0.007536	-0.007564	5.04E-09	4.47E-09	0.410002
8:11234520	-0.007548	-0.007578	5.04E-09	4.43E-09	0.425985
8:11234885	-0.007541	-0.007571	5.12E-09	4.49E-09	0.424282
8:11233582	-0.007535	-0.007569	5.16E-09	4.44E-09	0.398733
8:11233659	-0.007534	-0.007568	5.18E-09	4.45E-09	0.398444
8:11234298	-0.007542	-0.007572	5.18E-09	4.54E-09	0.419525
8:11062882	-0.007525	-0.007545	5.19E-09	4.75E-09	0.482151
8:11234844	-0.007537	-0.007568	5.31E-09	4.65E-09	0.423366
8:11233958	-0.007539	-0.007569	5.37E-09	4.70E-09	0.434524
8:11236975	-0.007528	-0.007556	5.40E-09	4.76E-09	0.417938
8:11232343	-0.007538	-0.007561	5.42E-09	4.91E-09	0.442346
8:11234780	-0.007531	-0.007561	5.47E-09	4.80E-09	0.423597
8:11230206	-0.007537	-0.007565	5.51E-09	4.86E-09	0.412341
8:11233917	-0.007534	-0.007564	5.51E-09	4.81E-09	0.434969
8:11234367	-0.007527	-0.007557	5.57E-09	4.88E-09	0.416627
8:11235360	-0.007524	-0.007553	5.59E-09	4.92E-09	0.429098
8:11234626	-0.00753	-0.007558	5.61E-09	4.96E-09	0.42357
8:11235497	-0.007514	-0.007544	5.70E-09	5.00E-09	0.396491
8:11234613	-0.007523	-0.007551	5.78E-09	5.12E-09	0.42366
8:10988275	-0.008199	-0.008181	5.79E-09	6.32E-09	0.506764
8:11230259	-0.007527	-0.007555	5.79E-09	5.12E-09	0.414148
8:11236850	-0.007503	-0.007532	5.81E-09	5.12E-09	0.390934
8:11236964	-0.007504	-0.007532	5.81E-09	5.12E-09	0.387832
8:11235735	-0.007512	-0.007541	5.85E-09	5.15E-09	0.426266
8:11229319	-0.007531	-0.007555	5.90E-09	5.34E-09	0.425943
8:11080665	-0.00753	-0.007536	6.04E-09	5.93E-09	0.461293
8:11235136	-0.00751	-0.007539	6.04E-09	5,33E+07	0.426922
8:11249261	0.008415	0.008472	6.14E-09	4.89E-09	0.266491
8:11243126	-0.007524	-0.007555	6.27E-09	5.48E-09	0.475733
8:11236809	-0.007484	-0.007513	6.35E-09	5.59E-09	0.39368
8:11238029	-0.007501	-0.007536	6.40E-09	5.49E-09	0.469878

chromosome:position	*beta*	*beta_LL_cytoSNP_out*	*p-value*	*p-value_LL_cytoSNP_out*	*i2*
8:11239640	-0.00751	-0.007538	6.44E-09	5.70E-09	0.463109
8:11231354	0.007478	0.007504	6.46E-09	5.79E-09	0.406758
8:11235605	-0.007492	-0.007522	6.55E-09	5.74E-09	0.396929
8:11235393	-0.007485	-0.007514	6.65E-09	5.85E-09	0.428798
8:11235579	-0.007475	-0.007507	7.06E-09	6.14E-09	0.391376
8:11046394	-0.008162	-0.00818	7.69E-09	7.20E-09	0.406279
8:11239510	-0.00746	-0.007489	7.93E-09	7.02E-09	0.466552
8:11239565	-0.007459	-0.007487	7.99E-09	7.07E-09	0.46584
8:10903475	-0.007448	-0.00743	8.09E-09	8.82E-09	0.547415
8:11239352	-0.00745	-0.007478	8.18E-09	7.23E-09	0.467694
8:11229638	-0.007456	-0.007478	8.20E-09	7.48E-09	0.444053
8:8700851	0.007429	0.007432	8.41E-09	8.31E-09	0.509995
8:11239137	-0.007434	-0.007463	8.81E-09	7.78E-09	0.467168
8:11238597	-0.007428	-0.007457	8.92E-09	7.85E-09	0.462827
8:11061792	-0.007402	-0.007424	8.98E-09	8.20E-09	0.486205
8:11060311	-0.007402	-0.007423	9.05E-09	8.24E-09	0.476986
8:11071057	-0.007484	-0.007493	9.10E-09	8.84E-09	0.537626
8:11237773	-0.007413	-0.007441	9.23E-09	8.17E-09	0.466364
8:11235910	-0.007423	-0.007457	9.37E-09	8.10E-09	0.458493
8:8646246	0.007416	0.007409	9.37E-09	9.68E-09	0.447765
8:11239078	-0.007419	-0.007448	9.43E-09	8.33E-09	0.468722
8:11237756	-0.007376	-0.007404	9.75E-09	8.63E-09	0.468782
8:11239017	-0.007409	-0.007438	9.87E-09	8.70E-09	0.46476
8:11239054	-0.007403	-0.007432	1.03E-08	9.08E-09	0.467051
8:11079367	-0.007379	-0.007394	1.08E-08	1.01E-08	0.498061
8:11078949	-0.007319	-0.007331	1.42E-08	1.36E-08	0.507733
8:11247814	0.007548	0.007582	1.46E-08	1.27E-08	0.357556
8:8633548	-0.007318	-0.007309	1.51E-08	1.58E-08	0.465781
8:8664622	0.007302	0.007302	1.61E-08	1.62E-08	0.33436
8:11076635	-0.007286	-0.007298	1.63E-08	1.55E-08	0.517329
8:8661534	0.007262	0.007256	1.65E-08	1.71E-08	0.461778

chromosome:position	*beta*	*beta_LL_cytoSNP_out*	*p-value*	*p-value_LL_cytoSNP_out*	*i2*
8:8652889	0.007269	0.007263	1.67E-08	1.72E-08	0.461896
8:11078781	-0.007282	-0.007295	1.68E-08	1.58E-08	0.504546
8:8661681	0.007243	0.007233	1.92E-08	2.02E-08	0.441258
8:8685854	0.007265	0.007263	1.92E-08	1.94E-08	0.45352
8:8641145	0.007248	0.007235	2.01E-08	2.15E-08	0.43898
8:11219781	0.007242	0.007252	2.03E-08	1.95E-08	0.400284
8:8637429	-0.007226	-0.007214	2.15E-08	2.28E-08	0.450121
8:8721473	0.007253	0.007263	2.18E-08	2.10E-08	0.52241
8:10987553	-0.007267	-0.007253	2.20E-08	2.37E-08	0.477455
8:8770512	0.007282	0.007223	2.27E-08	2.98E-08	0.34965
8:11242025	-0.007269	-0.007301	2.28E-08	1.99E-08	0.419451
8:8678530	0.007207	0.007208	2.37E-08	2.37E-08	0.398588
8:11069960	-0.007195	-0.007216	2.40E-08	2.20E-08	0.52078
8:11251175	0.007353	0.007399	2.43E-08	2.00E-08	0.400674
8:11215617	0.007177	0.007185	2.55E-08	2.48E-08	0.386663
8:10938260	-0.007176	-0.007188	2.63E-08	2.51E-08	0.528811
8:10987651	-0.007225	-0.007209	2.65E-08	2.86E-08	0.485532
8:11055597	-0.007165	-0.007185	2.67E-08	2.46E-08	0.416053
8:8685646	0.007186	0.00718	2.69E-08	2.78E-08	0.488867
8:8673601	0.007168	0.007169	2.76E-08	2.76E-08	0.40252
8:11214972	0.007146	0.007149	2.80E-08	2.80E-08	0.338936
8:8639740	-0.007166	-0.007154	2.85E-08	3.02E-08	0.466298
8:11219334	0.007168	0.007179	2.87E-08	2.75E-08	0.440499
8:8704330	0.007156	0.007152	2.87E-08	2.95E-08	0.507764
8:11214455	0.00714	0.007145	2.89E-08	2.84E-08	0.313706
8:8682192	0.007164	0.007161	2.91E-08	2.97E-08	0.382837
8:8679614	0.00716	0.007161	2.92E-08	2.93E-08	0.382412
8:8675325	0.007152	0.007149	3.00E-08	3.06E-08	0.461198
8:10987199	-0.007183	-0.007167	3.08E-08	3.35E-08	0.450852
8:8684953	0.007165	0.007168	3.09E-08	3.07E-08	0.388777
8:8660538	0.007136	0.007129	3.14E-08	3.25E-08	0.445874

chromosome:position	***beta***	***beta_LL_cytoSNP_out***	***p-value***	***p-value_LL_cytoSNP_out***	***i2***
8:8673736	0.007136	0.007137	3.14E-08	3.14E-08	0.413675
8:11225168	-0.007119	-0.007145	3.18E-08	2.87E-08	0.313122
8:8730488	0.007178	0.007185	3.24E-08	3.16E-08	0.521281
8:11225910	-0.007125	-0.007151	3.27E-08	2.94E-08	0.353716
8:8679176	0.007118	0.007119	3.52E-08	3.52E-08	0.394142
8:11225480	-0.007088	-0.007115	3.66E-08	3.28E-08	0.311814
8:8680477	0.007109	0.007109	3.66E-08	3.68E-08	0.376871
8:8682878	0.007108	0.007103	3.68E-08	3.79E-08	0.509731
8:11213589	0.007102	0.007111	3.74E-08	3.62E-08	0.430743
8:9569104	0.009116	0.009108	3.84E-08	4.00E-08	0
8:11070360	-0.007068	-0.007087	3.98E-08	3.69E-08	0.511645
8:11045161	-0.007055	-0.00708	4.08E-08	3.67E-08	0.471785
8:11228672	0.00705	0.007086	4.16E-08	3.59E-08	0.34481
8:11054097	-0.007068	-0.007088	4.17E-08	3.83E-08	0.415959
8:11046209	-0.007142	-0.007158	4.37E-08	4.10E-08	0.460309
8:10986837	-0.007109	-0.00709	4.41E-08	4.82E-08	0.497053
8:11041642	-0.007042	-0.00707	4.46E-08	3.97E-08	0.466136
8:11252425	0.00745	0.007451	4.46E-08	4.48E-08	0.389993
8:8644274	0.007074	0.007062	4.53E-08	4.80E-08	0.44873
8:11425809	-0.007038	-0.007063	4.79E-08	4.30E-08	0.508878
8:8685190	0.00706	0.007061	4.81E-08	4.82E-08	0.385978
8:8654527	0.007035	0.007021	4.91E-08	5.24E-08	0.400242
8:11039816	-0.007028	-0.007057	4.94E-08	4.37E-08	0.478889
12:62989110	-0.00726	-0.007303	1.50E-08	1.26E-08	0.543745
12:62995984	-0.008214	-0.008259	1.52E-08	1.29E-08	0.4616
12:62986620	-0.008176	-0.008222	1.57E-08	1.33E-08	0.45937
12:62990403	-0.007246	-0.007289	1.60E-08	1.34E-08	0.544762
12:62990415	-0.007244	-0.007287	1.61E-08	1.35E-08	0.545017
12:62995340	-0.007497	-0.007535	2.23E-08	1.93E-08	0.394087
12:62945970	-0.007478	-0.007514	2.38E-08	2.08E-08	0.407387
12:62977163	-0.007489	-0.007525	2.41E-08	2.10E-08	0.38373

chromosome:position	*beta*	*beta_LL_cytoSNP_out*	*p-value*	*p-value_LL_cytoSNP_out*	*i2*
12:62995269	-0.007479	-0.007515	2.41E-08	2.09E-08	0.382106
12:62968280	-0.007464	-0.0075	2.50E-08	2.18E-08	0.393524
12:62948736	-0.007468	-0.007503	2.58E-08	2.26E-08	0.416193
12:62985871	-0.007461	-0.007499	2.59E-08	2.24E-08	0.397868
12:62950556	-0.007452	-0.007487	2.66E-08	2.33E-08	0.407056
12:62942258	-0.007438	-0.007472	2.83E-08	2.50E-08	0.419595
12:62960880	-0.007405	-0.007442	2.98E-08	2.59E-08	0.413649
12:62961299	-0.007425	-0.00746	3.05E-08	2.67E-08	0.401325
12:62957906	-0.007389	-0.007426	3.25E-08	2.83E-08	0.420553
12:62996061	-0.007427	-0.007465	3.29E-08	2.85E-08	0.4224
12:62993793	-0.007428	-0.007466	3.31E-08	2.87E-08	0.427323
12:62952696	-0.007403	-0.007437	3.33E-08	2.93E-08	0.404831
12:62949110	-0.007376	-0.007411	3.38E-08	2.95E-08	0.421368
12:62961801	-0.007377	-0.007414	3.42E-08	2.98E-08	0.417933
12:62975307	-0.007408	-0.007445	3.48E-08	3.02E-08	0.422277
12:62937532	-0.007354	-0.007397	3.52E-08	2.98E-08	0.516077
12:62990871	-0.007389	-0.007426	3.56E-08	3.09E-08	0.399641
12:62964552	-0.007429	-0.007464	3.57E-08	3.14E-08	0.441951
12:62970482	-0.007376	-0.007414	3.63E-08	3.14E-08	0.441969
12:62945245	-0.007386	-0.00742	3.73E-08	3.28E-08	0.428268
12:62952910	-0.007379	-0.007415	3.90E-08	3.41E-08	0.435652
12:62980580	-0.007328	-0.007365	4.22E-08	3.66E-08	0.40879
12:62988288	-0.007354	-0.007392	4.38E-08	3.80E-08	0.430955
12:62870574	-0.007274	-0.007313	4.74E-08	4.07E-08	0.433408
12:62941837	-0.007297	-0.007331	4.93E-08	4.33E-08	0.441654
12:62941426	-0.007294	-0.007329	4.97E-08	4.37E-08	0.43931
13:83312341	-0.007206	-0.007155	3.23E-08	4.11E-08	0
13:83311141	-0.007179	-0.007128	3.52E-08	4.48E-08	0
13:83312717	-0.007177	-0.007129	3.74E-08	4.69E-08	0
13:83304898	-0.007095	-0.007045	4.87E-08	6.14E-08	0
16:13040514	-0.008169	-0.008077	2.19E-08	3.21E-08	0

chromosome:position	***beta***	***beta_LL_cytoSNP_out***	***p-value***	***p-value_LL_cytoSNP_out***	***i2***
16:13041027	-0.008021	-0.007929	3.66E-08	5.32E-08	0
16:13042097	-0.00802	-0.007927	3.66E-08	5.36E-08	0
16:13039154	-0.008713	-0.008609	3.79E-08	5.59E-08	0
16:13040889	-0.008004	-0.007912	3.86E-08	5.60E-08	0
16:13043452	-0.007971	-0.00788	4.41E-08	6.36E-08	0
16:13041924	-0.007968	-0.007877	4.44E-08	6.42E-08	0

First order sorting by chromosome then by significance.

Table S4: Leave-one-out

chromosome:position	*i2_LL_cytoSNP_out*
2:98580012	0.388423
2:98427382	0.454716
8:11417582	0.495412
8:11235150	0.38782
8:11226456	0.422573
8:10147398	0.269132
8:11226071	0.433021
8:11232788	0.443058
8:11231249	0.422232
8:11242632	0.463711
8:11233318	0.403188
8:11236681	0.401779
8:11236685	0.402229
8:11236419	0.417682
8:11245303	0.415191
8:11236413	0.41966
8:11236572	0.406862
8:11230574	0.426599
8:11237477	0.389609
8:11237480	0.389835
8:11233419	0.402678
8:11231886	0.444055
8:11236392	0.41356
8:11227406	0.434631
8:11239762	0.407622
8:11249010	0.421954
8:11232860	0.464152
8:11080675	0.489721
8:11227885	0.436759
8:11228100	0.434196

chromosome:position	*i2_LL_cytoSNP_out*
8:11234500	0.425
8:11234520	0.438644
8:11234885	0.435865
8:11233582	0.402933
8:11233659	0.402501
8:11234298	0.431152
8:11062882	0.501504
8:11234844	0.434351
8:11233958	0.4456
8:11236975	0.431299
8:11232343	0.462768
8:11234780	0.435157
8:11230206	0.426097
8:11233917	0.445915
8:11234367	0.428141
8:11235360	0.442249
8:11234626	0.438076
8:11235497	0.406929
8:11234613	0.43839
8:10988275	0.540598
8:11230259	0.428318
8:11236850	0.402117
8:11236964	0.399663
8:11235735	0.43916
8:11229319	0.446583
8:11080665	0.492155
8:11235136	0.440555
8:11249261	0.220863
8:11243126	0.487622
8:11236809	0.405048
8:11238029	0.475474

chromosome:position	*i2_LL_cytoSNP_out*
8:11239640	0.478066
8:11231354	0.426665
8:11235605	0.407036
8:11235393	0.441485
8:11235579	0.39831
8:11046394	0.442699
8:11239510	0.481346
8:11239565	0.48058
8:10903475	0.570047
8:11239352	0.482186
8:11229638	0.467344
8:8700851	0.538481
8:11239137	0.481056
8:11238597	0.475978
8:11061792	0.504711
8:11060311	0.494947
8:11071057	0.565545
8:11237773	0.481172
8:11235910	0.466184
8:8646246	0.480947
8:11239078	0.482643
8:11237756	0.483034
8:11239017	0.47815
8:11239054	0.48081
8:11079367	0.522373
8:11078949	0.53382
8:11247814	0.367023
8:8633548	0.494913
8:8664622	0.375963
8:11076635	0.543992
8:8661534	0.49042

chromosome:position	*i2_LL_cytoSNP_out*
8:8652889	0.490761
8:11078781	0.5294
8:8661681	0.471034
8:8685854	0.487551
8:8641145	0.467467
8:11219781	0.433138
8:8637429	0.477485
8:8721473	0.548281
8:10987553	0.506788
8:8770512	0.305538
8:11242025	0.42843
8:8678530	0.431949
8:11069960	0.540735
8:11251175	0.384758
8:11215617	0.421226
8:10938260	0.555619
8:10987651	0.513821
8:11055597	0.439607
8:8685646	0.517872
8:8673601	0.437625
8:11214972	0.377659
8:8639740	0.492938
8:11219334	0.472691
8:8704330	0.536187
8:11214455	0.353219
8:8682192	0.416707
8:8679614	0.416696
8:8675325	0.492614
8:10987199	0.47835
8:8684953	0.42448
8:8660538	0.477101

chromosome:position	*i2_LL_cytoSNP_out*
8:8673736	0.446223
8:11225168	0.337957
8:8730488	0.548258
8:11225910	0.37899
8:8679176	0.427771
8:11225480	0.335335
8:8680477	0.41149
8:8682878	0.53784
8:11213589	0.464471
8:9569104	0
8:11070360	0.532057
8:11045161	0.484872
8:11228672	0.354226
8:11054097	0.436904
8:11046209	0.487547
8:10986837	0.523213
8:11041642	0.477061
8:11252425	0.425862
8:8644274	0.476354
8:11425809	0.522977
8:8685190	0.422092
8:8654527	0.428191
8:11039816	0.48793
12:62989110	0.55691
12:62995984	0.486312
12:62986620	0.481498
12:62990403	0.557832
12:62990415	0.558088
12:62995340	0.412887
12:62945970	0.428078
12:62977163	0.40413

chromosome:position	*i2_LL_cytoSNP_out*
12:62995269	0.40194
12:62968280	0.414283
12:62948736	0.436978
12:62985871	0.416845
12:62950556	0.42774
12:62942258	0.441142
12:62960880	0.433111
12:62961299	0.422364
12:62957906	0.439999
12:62996061	0.442237
12:62993793	0.447006
12:62952696	0.426463
12:62949110	0.441784
12:62961801	0.437638
12:62975307	0.442816
12:62937532	0.531965
12:62990871	0.418782
12:62964552	0.465522
12:62970482	0.461391
12:62945245	0.450589
12:62952910	0.457136
12:62980580	0.427845
12:62988288	0.450895
12:62870574	0.450453
12:62941837	0.462224
12:62941426	0.459923
13:83312341	0
13:83311141	0
13:83312717	0
13:83304898	0
16:13040514	0

chromosome:position	*i2_LL_cytoSNP_out*
16:13041027	0
16:13042097	0
16:13039154	0
16:13040889	0
16:13043452	0
16:13041924	0

First order sorting by chro

Table S5: VEGAS2 gene mapping

Chr	***Gene***	***nSNPs***	***nSims***	***Start***	***Stop***	***Test***	***Pvalue***	***Best.SNP***	***SNP.pvalue***
8	*C8orf12*	322	1000000	11225910	11296166	2.253.150.337	1.00E-06	rs35009431	2.99E-09
8	*XKR6*	1206	1000000	10753656	11058875	4.940.377.089	1.00E-06	rs4841507	7.69E-09
8	*MFHAS1*	586	1000000	8641998	8751131	4.006.720.976	1.00E-06	rs11249893	8.41E-09
12	*MON2*	450	1000000	62860596	62991363	3.277.960.943	2.00E-06	rs10877884	0.000000015
12	*C12orf61*	9	1000000	62995530	62997214	1.018.633.632	1.00E-06	rs10877885	1.52E-08
2	*TMEM131*	717	1000000	98372800	98612354	4.425.086.135	2.00E-06	rs35098301	1.57E-08
16	*SHISA9*	1670	1000000	12995476	13334273	5.249.525.599	1.00E-06	rs12922637	2.19E-08
8	*TDH*	110	1000000	11197145	11225961	1.122.272.376	1.00E-06	rs2249804	2.55E-08
14	*PTPN21*	297	1000000	88932121	89021123	2.730.372.939	1.00E-06	rs1152376	8.93E-08
14	*SPATA7*	117	1000000	88851987	88904804	6.591.039.894	2.00E-06	rs2747081	0.000000178
8	*SLC35G5*	9	1000000	11188494	11189695	1.339.169.005	1.00E-06	rs12681987	0.000000205
15	*WDR76*	107	1000000	44119111	44160617	8.280.972.749	2.00E-06	rs4597285	0.00000184
15	*RNU6-28P_15*	518	1000000	43675287	43894832	3.396.272.122	2.00E-06	rs2444247	0.00000275
2	*TEX41*	1074	1000000	145425533	145834291	3.886.313.145	1.00E-06	rs2381687	0.00000528
3	*ETV5*	159	1000000	185764105	185826901	7.915.419.228	2.00E-06	rs12638263	0.00000953
4	*THAP6*	48	1000000	76439653	76455236	2.212.315.192	2.00E-06	rs13103482	0.000017
6	*HCG14*	4	1000000	28864306	28865097	3.262.777.563	2.00E-06	rs3135316	0.000104

Sorted by SNP.pvalue significance. Abbreviations: Chr = chromosome; nSNPs = number of SNPs; nSims = number of simulations

Table S6: Gene mapping FUMA

uniqID	*rsID*	*chr*	*pos*	*non_effect allele*	*effect_allele*
2:98325330:G:T	2:98325330	2	98325330	T	G
2:98326867:A:G	2:98326867	2	98326867	A	G
2:98330052:A:C	2:98330052	2	98330052	A	C
2:98334679:C:T	2:98334679	2	98334679	T	C
2:98336200:A:C	2:98336200	2	98336200	C	A
2:98339513:C:T	2:98339513	2	98339513	T	C
2:98342323:A:C	2:98342323	2	98342323	A	C
2:98343258:A:G	2:98343258	2	98343258	G	A
2:98345086:G:T	2:98345086	2	98345086	G	T
2:98346461:A:G	2:98346461	2	98346461	A	G
2:98351654:T:TG	rs34532595	2	98351654	TG	T
2:98351986:C:T	2:98351986	2	98351986	C	T
2:98353847:C:T	2:98353847	2	98353847	T	C
2:98354139:G:T	2:98354139	2	98354139	T	G
2:98354511:A:G	2:98354511	2	98354511	A	G
2:98355990:A:G	2:98355990	2	98355990	A	G
2:98356846:A:G	2:98356846	2	98356846	A	G
2:98357163:C:T	2:98357163	2	98357163	C	T
2:98360443:A:G	2:98360443	2	98360443	G	A
2:98361679:C:G	2:98361679	2	98361679	G	C
2:98363313:A:T	2:98363313	2	98363313	A	T
2:98365164:C:G	2:98365164	2	98365164	C	G
2:98368551:A:ACT	rs34388573	2	98368551	ACT	A
2:98370698:A:T	2:98370698	2	98370698	A	T
2:98374567:C:T	2:98374567	2	98374567	T	C
2:98377512:A:T	2:98377512	2	98377512	T	A
2:98379267:C:T	2:98379267	2	98379267	T	C
2:98379813:A:G	2:98379813	2	98379813	A	G
2:98382886:A:G	2:98382886	2	98382886	A	G
2:98384528:A:ATTTTC	rs149697530	2	98384528	ATTTTC	A
2:98386731:C:T	2:98386731	2	98386731	C	T

uniqID	*rsID*	*chr*	*pos*	*non_effect allele*	*effect_allele*
2:98390117:G:GT	rs538393585	2	98390117	G	GT
2:98393231:C:T	2:98393231	2	98393231	T	C
2:98393894:C:T	2:98393894	2	98393894	T	C
2:98395653:A:C	2:98395653	2	98395653	C	A
2:98397301:C:T	2:98397301	2	98397301	T	C
2:98402753:A:T	2:98402753	2	98402753	A	T
2:98402772:C:G	2:98402772	2	98402772	C	G
2:98405034:C:T	2:98405034	2	98405034	C	T
2:98405695:C:T	2:98405695	2	98405695	T	C
2:98405929:A:G	2:98405929	2	98405929	G	A
2:98407350:C:T	2:98407350	2	98407350	T	C
2:98409046:A:G	2:98409046	2	98409046	A	G
2:98409565:C:T	2:98409565	2	98409565	C	T
2:98410769:G:T	2:98410769	2	98410769	T	G
2:98413781:C:T	2:98413781	2	98413781	T	C
2:98416848:A:C	2:98416848	2	98416848	A	C
2:98416850:G:T	2:98416850	2	98416850	G	T
2:98417089:C:T	2:98417089	2	98417089	T	C
2:98419726:A:T	2:98419726	2	98419726	A	T
2:98420142:C:G	2:98420142	2	98420142	C	G
2:98420431:C:T	2:98420431	2	98420431	C	T
2:98421364:A:G	2:98421364	2	98421364	G	A
2:98424802:A:G	2:98424802	2	98424802	G	A
2:98427382:C:T	2:98427382	2	98427382	C	T
2:98434931:G:GGGGGC	rs147071183	2	98434931	GGGGGC	G
2:98435982:C:T	2:98435982	2	98435982	T	C
2:98440234:C:T	2:98440234	2	98440234	T	C
2:98442202:A:G	2:98442202	2	98442202	A	G
2:98443039:C:G	2:98443039	2	98443039	C	G
2:98443081:G:GA	rs35808422	2	98443081	GA	G
2:98443658:C:T	2:98443658	2	98443658	T	C
2:98447004:C:T	2:98447004	2	98447004	C	T

uniqID	*rsID*	*chr*	*pos*	*non_effect allele*	*effect_allele*
2:98448395:G:T	2:98448395	2	98448395	T	G
2:98449024:A:G	2:98449024	2	98449024	A	G
2:98454472:A:G	2:98454472	2	98454472	A	G
2:98454473:C:T	2:98454473	2	98454473	T	C
2:98454572:C:G	2:98454572	2	98454572	C	G
2:98454931:A:G	2:98454931	2	98454931	A	G
2:98455152:C:T	2:98455152	2	98455152	T	C
2:98455510:C:G	2:98455510	2	98455510	C	G
2:98461821:C:T	2:98461821	2	98461821	T	C
2:98461953:C:T	2:98461953	2	98461953	T	C
2:98466647:C:T	2:98466647	2	98466647	T	C
2:98468892:A:AC	rs58300457	2	98468892	AC	A
2:98472610:C:T	2:98472610	2	98472610	C	T
2:98479581:A:C	2:98479581	2	98479581	A	C
2:98488949:C:G	2:98488949	2	98488949	C	G
2:98491750:C:T	2:98491750	2	98491750	C	T
2:98493359:A:G	2:98493359	2	98493359	G	A
2:98495661:G:T	2:98495661	2	98495661	G	T
2:98501884:C:T	2:98501884	2	98501884	C	T
2:98502987:C:T	2:98502987	2	98502987	C	T
2:98506011:T:TATA	rs71386038	2	98506011	TATA	T
2:98506910:C:T	2:98506910	2	98506910	C	T
2:98507972:C:CA	rs35677645	2	98507972	CA	C
2:98510166:G:T	2:98510166	2	98510166	G	T
2:98517626:A:G	2:98517626	2	98517626	A	G
2:98519014:C:T	2:98519014	2	98519014	C	T
2:98520564:C:T	2:98520564	2	98520564	C	T
2:98521823:A:C	2:98521823	2	98521823	A	C
2:98526685:A:AAAAT	rs148038816	2	98526685	AAAAT	A
2:98528689:A:G	2:98528689	2	98528689	A	G
2:98534412:C:T	2:98534412	2	98534412	T	C
2:98534531:C:T	2:98534531	2	98534531	T	C

uniqID	*rsID*	*chr*	*pos*	*non_effect allele*	*effect_allele*
2:98539588:A:C	2:98539588	2	98539588	A	C
2:98541001:C:T	2:98541001	2	98541001	C	T
2:98547069:A:AT	rs35058741	2	98547069	AT	A
2:98548604:T:TA	rs34976723	2	98548604	TA	T
2:98551456:G:GA	rs545443077	2	98551456	G	GA
2:98552271:C:G	2:98552271	2	98552271	C	G
2:98552299:C:G	2:98552299	2	98552299	G	C
2:98554946:A:C	2:98554946	2	98554946	C	A
2:98557575:G:T	2:98557575	2	98557575	G	T
2:98561153:A:C	2:98561153	2	98561153	A	C
2:98565115:C:CT	rs137894541	2	98565115	C	CT
2:98565144:A:G	2:98565144	2	98565144	A	G
2:98565400:A:G	2:98565400	2	98565400	A	G
2:98567820:A:G	2:98567820	2	98567820	A	G
2:98570999:A:G	2:98570999	2	98570999	A	G
2:98571084:A:G	2:98571084	2	98571084	G	A
2:98580012:C:T	2:98580012	2	98580012	C	T
2:98580724:G:T	2:98580724	2	98580724	T	G
2:98587288:A:G	2:98587288	2	98587288	G	A
2:98588372:A:C	2:98588372	2	98588372	A	C
2:98602165:A:G	2:98602165	2	98602165	G	A
2:98604570:G:GACAC	rs140523936	2	98604570	GACAC	G
2:98605982:A:G	2:98605982	2	98605982	A	G
2:98606770:C:T	2:98606770	2	98606770	C	T
2:98612260:C:T	2:98612260	2	98612260	C	T
2:98616519:A:ATT	rs370334808	2	98616519	ATT	A
2:98621058:A:AG	rs147329160	2	98621058	AG	A
2:98621060:A:T	2:98621060	2	98621060	T	A
2:98623227:A:G	2:98623227	2	98623227	A	G
2:98623406:A:G	2:98623406	2	98623406	G	A
8:8524474:C:G	8:8524474	8	8524474	C	G
8:8543324:C:T	8:8543324	8	8543324	C	T

uniqID	*rsID*	*chr*	*pos*	*non_effect allele*	*effect_allele*
8:8544808:C:T	8:8544808	8	8544808	C	T
8:8544872:A:G	8:8544872	8	8544872	A	G
8:8545624:T:TTTG	rs141541089	8	8545624	TTTG	T
8:8546283:A:AAATT	rs3081202	8	8546283	A	AAATT
8:8547110:C:G	8:8547110	8	8547110	C	G
8:8547313:A:C	8:8547313	8	8547313	C	A
8:8547811:A:G	8:8547811	8	8547811	G	A
8:8548117:A:T	8:8548117	8	8548117	A	T
8:8548801:A:G	8:8548801	8	8548801	A	G
8:8549020:A:C	8:8549020	8	8549020	C	A
8:8549432:A:G	8:8549432	8	8549432	A	G
8:8578067:C:T	8:8578067	8	8578067	C	T
8:8578120:A:T	8:8578120	8	8578120	A	T
8:8578229:A:G	8:8578229	8	8578229	G	A
8:8578794:A:T	8:8578794	8	8578794	T	A
8:8578811:C:T	8:8578811	8	8578811	C	T
8:8581408:C:G	8:8581408	8	8581408	G	C
8:8582155:A:AAAAAGAAAAG	rs144010740	8	8582155	AAAAAGAAAAG	A
8:8582155:AAAAAG:AAAAAGAAAAG	rs555499155	8	8582155	AAAAAGAAAAG	AAAAAG
8:8583872:C:T	8:8583872	8	8583872	C	T
8:8584344:A:G	8:8584344	8	8584344	A	G
8:8587571:G:T	8:8587571	8	8587571	G	T
8:8589117:C:T	8:8589117	8	8589117	T	C
8:8592845:C:G	8:8592845	8	8592845	G	C
8:8595104:A:T	8:8595104	8	8595104	T	A
8:8595838:G:T	8:8595838	8	8595838	G	T
8:8596731:G:T	8:8596731	8	8596731	T	G
8:8598388:A:T	8:8598388	8	8598388	T	A
8:8602344:C:T	8:8602344	8	8602344	C	T
8:8603160:A:G	8:8603160	8	8603160	G	A
8:8633548:G:T	8:8633548	8	8633548	G	T
8:8637429:A:G	8:8637429	8	8637429	A	G

uniqID	*rsID*	*chr*	*pos*	*non_effect allele*	*effect_allele*
8:8639740:A:G	8:8639740	8	8639740	G	A
8:8640172:A:C	8:8640172	8	8640172	A	C
8:8641145:C:T	8:8641145	8	8641145	T	C
8:8643938:C:T	8:8643938	8	8643938	T	C
8:8644213:G:GT	8:8644213:G:GT	8	8644213	G	GT
8:8644213:G:GTT	8:8644213:G:GTT	8	8644213	G	GTT
8:8644274:C:G	8:8644274	8	8644274	C	G
8:8644595:A:AT	rs371642880	8	8644595	A	AT
8:8646246:C:T	8:8646246	8	8646246	C	T
8:8649881:C:T	8:8649881	8	8649881	T	C
8:8652889:A:G	8:8652889	8	8652889	A	G
8:8654057:A:G	rs2409091	8	8654057	G	A
8:8654527:C:T	8:8654527	8	8654527	C	T
8:8658540:A:G	8:8658540	8	8658540	G	A
8:8660538:A:C	8:8660538	8	8660538	A	C
8:8661026:C:CA	rs34602481	8	8661026	CA	C
8:8661114:C:CA	rs34140883	8	8661114	C	CA
8:8661534:C:T	8:8661534	8	8661534	C	T
8:8661681:C:G	8:8661681	8	8661681	G	C
8:8663215:C:T	8:8663215	8	8663215	T	C
8:8664622:A:G	8:8664622	8	8664622	A	G
8:8664940:A:G	8:8664940	8	8664940	A	G
8:8665147:A:G	8:8665147	8	8665147	A	G
8:8665802:C:T	rs9644776	8	8665802	T	C
8:8666916:C:T	8:8666916	8	8666916	T	C
8:8667444:C:T	8:8667444	8	8667444	T	C
8:8668486:A:G	8:8668486	8	8668486	G	A
8:8668917:A:C	8:8668917	8	8668917	C	A
8:8669681:C:CGTAA	rs34087173	8	8669681	CGTAA	C
8:8669681:C:T	rs555149467	8	8669681	C	T
8:8670082:C:G	8:8670082	8	8670082	C	G
8:8670177:A:T	8:8670177	8	8670177	A	T

uniqID	*rsID*	*chr*	*pos*	*non_effect allele*	*effect_allele*
8:8670322:C:CT	rs60073172	8	8670322	C	CT
8:8670599:A:G	8:8670599	8	8670599	G	A
8:8670736:A:C	8:8670736	8	8670736	A	C
8:8671962:C:T	8:8671962	8	8671962	T	C
8:8672429:C:G	8:8672429	8	8672429	C	G
8:8672579:A:G	8:8672579	8	8672579	G	A
8:8672952:A:C	8:8672952	8	8672952	C	A
8:8673320:C:T	8:8673320	8	8673320	C	T
8:8673601:A:C	8:8673601	8	8673601	C	A
8:8673736:C:T	8:8673736	8	8673736	C	T
8:8675176:A:G	8:8675176	8	8675176	G	A
8:8675325:A:T	8:8675325	8	8675325	T	A
8:8676626:G:GAATC	rs5889248	8	8676626	GAATC	G
8:8678530:A:G	8:8678530	8	8678530	A	G
8:8679141:C:CTT	rs34362508	8	8679141	CTT	C
8:8679176:A:G	8:8679176	8	8679176	G	A
8:8679614:C:T	8:8679614	8	8679614	C	T
8:8680477:A:G	8:8680477	8	8680477	A	G
8:8682192:A:T	8:8682192	8	8682192	T	A
8:8682878:C:T	8:8682878	8	8682878	C	T
8:8684953:A:G	8:8684953	8	8684953	A	G
8:8685190:A:G	8:8685190	8	8685190	G	A
8:8685646:C:T	8:8685646	8	8685646	C	T
8:8685854:A:G	8:8685854	8	8685854	G	A
8:8687054:C:G	8:8687054	8	8687054	C	G
8:8687325:A:G	8:8687325	8	8687325	G	A
8:8690787:C:T	8:8690787	8	8690787	T	C
8:8691622:A:T	8:8691622	8	8691622	A	T
8:8692477:C:T	8:8692477	8	8692477	C	T
8:8696449:G:T	8:8696449	8	8696449	G	T
8:8698977:C:CA	rs11435426	8	8698977	C	CA
8:8699757:A:T	8:8699757	8	8699757	A	T

uniqID	*rsID*	*chr*	*pos*	*non_effect allele*	*effect_allele*
8:8699761:C:T	8:8699761	8	8699761	T	C
8:8700851:C:T	8:8700851	8	8700851	T	C
8:8702607:C:G	8:8702607	8	8702607	C	G
8:8702827:A:G	8:8702827	8	8702827	G	A
8:8702875:C:T	8:8702875	8	8702875	C	T
8:8703781:G:GGA	rs201075941	8	8703781	GGA	G
8:8704330:C:G	8:8704330	8	8704330	C	G
8:8706130:A:AT	rs35147353	8	8706130	AT	A
8:8706209:A:C	8:8706209	8	8706209	C	A
8:8706332:A:C	8:8706332	8	8706332	C	A
8:8707197:C:G	8:8707197	8	8707197	G	C
8:8708974:C:G	8:8708974	8	8708974	G	C
8:8709756:C:G	8:8709756	8	8709756	C	G
8:8709971:C:T	8:8709971	8	8709971	T	C
8:8713038:C:T	8:8713038	8	8713038	T	C
8:8718850:A:G	8:8718850	8	8718850	A	G
8:8719513:A:G	8:8719513	8	8719513	A	G
8:8721473:A:G	8:8721473	8	8721473	A	G
8:8722675:C:T	8:8722675	8	8722675	T	C
8:8723651:C:G	8:8723651	8	8723651	G	C
8:8724257:C:T	8:8724257	8	8724257	T	C
8:8724276:C:T	8:8724276	8	8724276	T	C
8:8724415:C:T	8:8724415	8	8724415	T	C
8:8725126:G:T	8:8725126	8	8725126	T	G
8:8725229:A:G	8:8725229	8	8725229	A	G
8:8725319:A:G	8:8725319	8	8725319	A	G
8:8726804:G:T	8:8726804	8	8726804	T	G
8:8729761:C:G	8:8729761	8	8729761	C	G
8:8730488:A:G	8:8730488	8	8730488	A	G
8:8768895:A:G	8:8768895	8	8768895	G	A
8:8769708:C:T	8:8769708	8	8769708	T	C
8:8770512:C:T	8:8770512	8	8770512	T	C

uniqID	*rsID*	*chr*	*pos*	*non_effect allele*	*effect_allele*
8:8824858:G:T	8:8824858	8	8824858	G	T
8:9394053:G:T	8:9394053	8	9394053	G	T
8:9489417:A:G	8:9489417	8	9489417	A	G
8:9492426:G:T	8:9492426	8	9492426	G	T
8:9492453:C:T	8:9492453	8	9492453	C	T
8:9494732:G:T	8:9494732	8	9494732	T	G
8:9496118:A:T	8:9496118	8	9496118	A	T
8:9525325:C:T	8:9525325	8	9525325	T	C
8:9527707:A:G	8:9527707	8	9527707	A	G
8:9527863:A:T	8:9527863	8	9527863	T	A
8:9527869:A:G	8:9527869	8	9527869	A	G
8:9568369:A:G	8:9568369	8	9568369	G	A
8:9569104:C:T	8:9569104	8	9569104	C	T
8:9569109:C:T	8:9569109	8	9569109	C	T
8:9572099:A:G	8:9572099	8	9572099	A	G
8:9574830:G:T	8:9574830	8	9574830	G	T
8:9575445:A:G	8:9575445	8	9575445	G	A
8:9579144:C:CT	rs76984329	8	9579144	C	CT
8:9579318:C:T	8:9579318	8	9579318	C	T
8:9579377:C:CA	rs67480155	8	9579377	CA	C
8:9583872:C:T	8:9583872	8	9583872	T	C
8:9584598:C:G	8:9584598	8	9584598	G	C
8:9586062:A:T	8:9586062	8	9586062	A	T
8:9593309:A:C	8:9593309	8	9593309	A	C
8:10121635:A:G	8:10121635	8	10121635	G	A
8:10122423:A:G	8:10122423	8	10122423	G	A
8:10146490:C:CT	rs61082051	8	10146490	C	CT
8:10147398:C:T	8:10147398	8	10147398	T	C
8:10148447:A:G	8:10148447	8	10148447	G	A
8:10149212:A:C	8:10149212	8	10149212	C	A
8:10150070:C:G	8:10150070	8	10150070	C	G
8:10176506:A:T	8:10176506	8	10176506	T	A

uniqID	*rsID*	*chr*	*pos*	*non_effect allele*	*effect_allele*
8:10758213:A:G	8:10758213	8	10758213	G	A
8:10810451:A:G	8:10810451	8	10810451	G	A
8:10811829:C:T	8:10811829	8	10811829	C	T
8:10812333:G:GT	rs34180049	8	10812333	G	GT
8:10813197:C:T	8:10813197	8	10813197	C	T
8:10813904:G:T	8:10813904	8	10813904	T	G
8:10815754:C:G	8:10815754	8	10815754	G	C
8:10816772:A:G	8:10816772	8	10816772	G	A
8:10817197:C:T	8:10817197	8	10817197	T	C
8:10818607:A:G	8:10818607	8	10818607	G	A
8:10818657:A:G	8:10818657	8	10818657	G	A
8:10819854:C:T	8:10819854	8	10819854	T	C
8:10821056:C:CT	rs35470964	8	10821056	C	CT
8:10828909:C:T	8:10828909	8	10828909	C	T
8:10831868:G:T	8:10831868	8	10831868	G	T
8:10835480:C:T	8:10835480	8	10835480	T	C
8:10835917:C:T	8:10835917	8	10835917	T	C
8:10836024:C:T	8:10836024	8	10836024	C	T
8:10836069:C:T	8:10836069	8	10836069	T	C
8:10836359:C:T	8:10836359	8	10836359	T	C
8:10836436:C:T	8:10836436	8	10836436	C	T
8:10836463:C:G	8:10836463	8	10836463	G	C
8:10836508:A:G	8:10836508	8	10836508	A	G
8:10837019:A:G	8:10837019	8	10837019	A	G
8:10837190:A:G	8:10837190	8	10837190	G	A
8:10837414:A:C	8:10837414	8	10837414	C	A
8:10837420:A:G	8:10837420	8	10837420	G	A
8:10837568:C:T	8:10837568	8	10837568	C	T
8:10837569:C:G	8:10837569	8	10837569	C	G
8:10839803:C:T	8:10839803	8	10839803	T	C
8:10841858:A:G	8:10841858	8	10841858	A	G
8:10842659:G:T	8:10842659	8	10842659	G	T

uniqID	*rsID*	*chr*	*pos*	*non_effect allele*	*effect_allele*
8:10903475:A:T	8:10903475	8	10903475	A	T
8:10909193:A:C	8:10909193	8	10909193	A	C
8:10909936:C:T	8:10909936	8	10909936	C	T
8:10910066:C:T	8:10910066	8	10910066	C	T
8:10910343:C:G	8:10910343	8	10910343	C	G
8:10926892:A:C	8:10926892	8	10926892	A	C
8:10927234:C:CAGTA	rs33960758	8	10927234	C	CAGTA
8:10930069:A:C	8:10930069	8	10930069	C	A
8:10932203:C:G	8:10932203	8	10932203	C	G
8:10932695:C:G	8:10932695	8	10932695	C	G
8:10932868:A:G	8:10932868	8	10932868	G	A
8:10933699:A:T	8:10933699	8	10933699	A	T
8:10935082:C:T	8:10935082	8	10935082	C	T
8:10935366:C:T	8:10935366	8	10935366	C	T
8:10935368:A:C	8:10935368	8	10935368	C	A
8:10935898:A:G	8:10935898	8	10935898	G	A
8:10936811:A:G	8:10936811	8	10936811	G	A
8:10936891:C:T	8:10936891	8	10936891	C	T
8:10938260:A:G	8:10938260	8	10938260	A	G
8:10939273:G:T	8:10939273	8	10939273	T	G
8:10939490:G:T	8:10939490	8	10939490	T	G
8:10943276:C:T	8:10943276	8	10943276	T	C
8:10944809:G:T	8:10944809	8	10944809	T	G
8:10945439:A:G	8:10945439	8	10945439	G	A
8:10945767:C:CA	rs529577981	8	10945767	CA	C
8:10948422:C:CA	rs34478855	8	10948422	C	CA
8:10948968:A:G	rs28722721	8	10948968	G	A
8:10950396:C:G	8:10950396	8	10950396	G	C
8:10950757:C:G	8:10950757	8	10950757	G	C
8:10950866:C:T	8:10950866	8	10950866	C	T
8:10951175:G:GT	rs34623605	8	10951175	G	GT
8:10952500:A:T	8:10952500	8	10952500	T	A

uniqID	*rsID*	*chr*	*pos*	*non_effect allele*	*effect_allele*
8:10953092:C:T	8:10953092	8	10953092	C	T
8:10953874:A:G	8:10953874	8	10953874	A	G
8:10955225:C:G	8:10955225	8	10955225	G	C
8:10955383:G:GTT	rs546003990	8	10955383	GTT	G
8:10955383:GT:GTT	rs559667973	8	10955383	GTT	GT
8:10957243:A:G	8:10957243	8	10957243	G	A
8:10958824:C:T	8:10958824	8	10958824	C	T
8:10960572:C:T	8:10960572	8	10960572	T	C
8:10961433:C:T	8:10961433	8	10961433	T	C
8:10962099:A:T	8:10962099	8	10962099	T	A
8:10962800:C:T	8:10962800	8	10962800	C	T
8:10962929:C:G	8:10962929	8	10962929	C	G
8:10963288:C:G	8:10963288	8	10963288	C	G
8:10964906:C:T	8:10964906	8	10964906	T	C
8:10964969:A:T	8:10964969	8	10964969	A	T
8:10968550:C:G	8:10968550	8	10968550	C	G
8:10968926:A:G	8:10968926	8	10968926	A	G
8:10969075:C:T	8:10969075	8	10969075	T	C
8:10970773:A:T	8:10970773	8	10970773	A	T
8:10973149:C:CAA	:10973149:C:CA	8	10973149	C	CAA
8:10973149:C:CA	3:10973149:C:C/	8	10973149	CA	C
8:10974917:C:T	8:10974917	8	10974917	T	C
8:10975081:A:T	8:10975081	8	10975081	A	T
8:10975629:A:C	8:10975629	8	10975629	A	C
8:10975682:C:G	8:10975682	8	10975682	G	C
8:10975733:A:G	8:10975733	8	10975733	G	A
8:10976494:C:G	8:10976494	8	10976494	C	G
8:10976569:A:G	8:10976569	8	10976569	A	G
8:10976571:G:T	rs6995805	8	10976571	T	G
8:10978065:G:T	rs7005680	8	10978065	T	G
8:10979561:C:G	8:10979561	8	10979561	G	C
8:10979821:A:G	8:10979821	8	10979821	G	A

uniqID	*rsID*	*chr*	*pos*	*non_effect allele*	*effect_allele*
8:10981003:A:C	8:10981003	8	10981003	C	A
8:10982051:A:G	8:10982051	8	10982051	G	A
8:10982410:C:G	8:10982410	8	10982410	G	C
8:10983534:A:G	8:10983534	8	10983534	G	A
8:10983579:G:T	rs60383089	8	10983579	G	T
8:10983921:C:G	8:10983921	8	10983921	G	C
8:10985140:A:G	8:10985140	8	10985140	G	A
8:10985432:C:T	8:10985432	8	10985432	T	C
8:10986837:G:T	8:10986837	8	10986837	G	T
8:10986859:C:T	8:10986859	8	10986859	T	C
8:10987199:C:T	8:10987199	8	10987199	C	T
8:10987553:C:T	8:10987553	8	10987553	C	T
8:10987651:G:T	8:10987651	8	10987651	G	T
8:10987967:A:G	8:10987967	8	10987967	A	G
8:10988138:A:C	8:10988138	8	10988138	C	A
8:10988275:A:G	8:10988275	8	10988275	G	A
8:10989057:A:G	8:10989057	8	10989057	A	G
8:10989206:C:T	8:10989206	8	10989206	C	T
8:10989521:A:G	8:10989521	8	10989521	A	G
8:10990164:A:G	8:10990164	8	10990164	A	G
8:10990371:G:GA	rs33917510	8	10990371	G	GA
8:10990672:C:T	8:10990672	8	10990672	T	C
8:10992252:C:G	8:10992252	8	10992252	G	C
8:10992544:A:AT	rs35184172	8	10992544	AT	A
8:10992605:A:C	8:10992605	8	10992605	A	C
8:10992883:C:T	8:10992883	8	10992883	T	C
8:10993904:C:T	8:10993904	8	10993904	T	C
8:10993995:C:T	rs34309263	8	10993995	C	T
8:10994743:C:G	8:10994743	8	10994743	C	G
8:10996089:A:G	8:10996089	8	10996089	G	A
8:11010974:G:T	8:11010974	8	11010974	G	T
8:11012977:C:T	8:11012977	8	11012977	T	C

uniqID	*rsID*	*chr*	*pos*	*non_effect allele*	*effect_allele*
8:11013025:A:C	8:11013025	8	11013025	A	C
8:11014616:G:T	8:11014616	8	11014616	G	T
8:11015338:C:T	8:11015338	8	11015338	T	C
8:11016889:A:G	8:11016889	8	11016889	G	A
8:11019578:C:G	8:11019578	8	11019578	G	C
8:11020313:G:T	8:11020313	8	11020313	T	G
8:11021682:A:G	8:11021682	8	11021682	G	A
8:11022106:C:G	8:11022106	8	11022106	C	G
8:11022185:C:T	8:11022185	8	11022185	C	T
8:11023655:C:T	8:11023655	8	11023655	T	C
8:11023997:C:G	8:11023997	8	11023997	C	G
8:11024275:C:T	8:11024275	8	11024275	C	T
8:11024326:A:C	8:11024326	8	11024326	C	A
8:11024663:A:C	8:11024663	8	11024663	A	C
8:11027491:T:TAA	rs113674355	8	11027491	T	TAA
8:11029029:G:T	8:11029029	8	11029029	G	T
8:11029039:G:T	8:11029039	8	11029039	G	T
8:11030892:A:G	8:11030892	8	11030892	G	A
8:11030935:C:T	8:11030935	8	11030935	C	T
8:11031472:C:T	8:11031472	8	11031472	C	T
8:11032228:G:T	8:11032228	8	11032228	G	T
8:11032240:C:T	8:11032240	8	11032240	T	C
8:11033517:C:G	8:11033517	8	11033517	C	G
8:11033525:C:T	8:11033525	8	11033525	T	C
8:11033737:A:G	8:11033737	8	11033737	A	G
8:11033976:C:T	8:11033976	8	11033976	C	T
8:11034028:A:G	8:11034028	8	11034028	G	A
8:11034859:A:G	8:11034859	8	11034859	G	A
8:11035071:G:GAGGTCATAATGGAAT	rs113410502	8	11035071	GAGGTCATAATGGAAT	G
8:11036052:T:TAA	rs35646464	8	11036052	TAA	T
8:11036799:C:G	8:11036799	8	11036799	C	G
8:11036843:C:T	8:11036843	8	11036843	C	T

uniqID	*rsID*	*chr*	*pos*	*non_effect allele*	*effect_allele*
8:11036919:A:G	8:11036919	8	11036919	A	G
8:11037034:A:G	8:11037034	8	11037034	A	G
8:11037187:C:T	8:11037187	8	11037187	C	T
8:11037903:G:T	8:11037903	8	11037903	G	T
8:11038244:A:T	8:11038244	8	11038244	T	A
8:11038885:A:T	8:11038885	8	11038885	A	T
8:11039159:C:G	8:11039159	8	11039159	C	G
8:11039816:G:T	8:11039816	8	11039816	G	T
8:11040216:C:T	8:11040216	8	11040216	C	T
8:11040647:A:G	8:11040647	8	11040647	G	A
8:11041642:A:T	8:11041642	8	11041642	T	A
8:11041661:C:T	8:11041661	8	11041661	C	T
8:11041897:T:TACACACACACACAC	rs141816060	8	11041897	TACACACACACACAC	T
8:11042974:C:G	8:11042974:C:G	8	11042974	G	C
8:11042974:G:T	8:11042974:G:T	8	11042974	G	T
8:11043138:C:T	8:11043138	8	11043138	C	T
8:11043236:C:T	8:11043236	8	11043236	T	C
8:11043926:C:T	8:11043926	8	11043926	T	C
8:11044689:A:G	8:11044689	8	11044689	G	A
8:11045161:A:G	8:11045161	8	11045161	A	G
8:11046209:C:T	8:11046209	8	11046209	C	T
8:11046394:C:G	8:11046394	8	11046394	G	C
8:11053922:A:G	8:11053922	8	11053922	A	G
8:11054097:A:C	8:11054097	8	11054097	A	C
8:11055597:A:C	8:11055597	8	11055597	A	C
8:11056175:A:G	8:11056175	8	11056175	G	A
8:11056388:C:T	8:11056388	8	11056388	C	T
8:11060217:T:TA	rs36080897	8	11060217	TA	T
8:11060311:A:C	8:11060311	8	11060311	C	A
8:11061792:C:T	8:11061792	8	11061792	T	C
8:11062882:C:T	8:11062882	8	11062882	C	T
8:11065003:A:C	8:11065003	8	11065003	A	C

uniqID	*rsID*	*chr*	*pos*	*non_effect allele*	*effect_allele*
8:11069960:C:T	8:11069960	8	11069960	T	C
8:11070360:C:G	8:11070360	8	11070360	G	C
8:11071057:A:G	8:11071057	8	11071057	G	A
8:11072020:A:G	8:11072020	8	11072020	G	A
8:11073402:A:G	8:11073402	8	11073402	A	G
8:11073578:C:T	8:11073578	8	11073578	T	C
8:11074036:A:G	8:11074036	8	11074036	A	G
8:11074365:C:CTA	rs36095784	8	11074365	CTA	C
8:11074812:C:T	8:11074812	8	11074812	T	C
8:11076635:G:T	8:11076635	8	11076635	G	T
8:11078781:A:C	8:11078781	8	11078781	C	A
8:11078949:A:G	8:11078949	8	11078949	A	G
8:11079367:A:G	8:11079367	8	11079367	A	G
8:11080014:C:T	8:11080014	8	11080014	T	C
8:11080665:A:T	8:11080665	8	11080665	A	T
8:11080675:C:T	8:11080675	8	11080675	T	C
8:11086942:C:T	8:11086942	8	11086942	C	T
8:11087475:A:G	8:11087475	8	11087475	A	G
8:11098992:A:G	rs12334496	8	11098992	G	A
8:11098996:C:T	rs12334549	8	11098996	C	T
8:11109269:C:T	8:11109269	8	11109269	C	T
8:11109303:C:G	8:11109303	8	11109303	G	C
8:11111462:A:T	8:11111462	8	11111462	T	A
8:11113089:A:G	8:11113089	8	11113089	A	G
8:11174484:C:G	8:11174484	8	11174484	C	G
8:11176403:A:G	8:11176403	8	11176403	A	G
8:11177126:A:C	8:11177126	8	11177126	A	C
8:11178093:A:C	8:11178093	8	11178093	C	A
8:11179458:G:T	8:11179458	8	11179458	T	G
8:11182148:C:G	8:11182148	8	11182148	C	G
8:11182455:A:G	8:11182455	8	11182455	G	A
8:11182704:A:G	8:11182704	8	11182704	A	G

uniqID	*rsID*	*chr*	*pos*	*non_effect allele*	*effect_allele*
8:11183505:A:G	8:11183505	8	11183505	A	G
8:11183765:C:CTTA	rs140914421	8	11183765	CTTA	C
8:11184390:A:C	8:11184390	8	11184390	C	A
8:11184396:A:G	8:11184396	8	11184396	A	G
8:11184478:C:CAA	rs34820607	8	11184478	CAA	C
8:11184937:A:G	8:11184937	8	11184937	A	G
8:11185096:C:G	8:11185096	8	11185096	C	G
8:11185671:G:T	8:11185671	8	11185671	T	G
8:11185673:A:AT	rs33915676	8	11185673	A	AT
8:11186215:A:C	8:11186215	8	11186215	C	A
8:11186453:C:T	8:11186453	8	11186453	C	T
8:11186639:C:G	8:11186639	8	11186639	C	G
8:11186674:A:G	8:11186674	8	11186674	G	A
8:11187078:G:T	8:11187078	8	11187078	T	G
8:11187434:C:T	8:11187434	8	11187434	C	T
8:11187651:A:G	8:11187651	8	11187651	A	G
8:11187675:C:T	8:11187675	8	11187675	C	T
8:11187770:C:G	8:11187770	8	11187770	G	C
8:11188532:C:G	8:11188532	8	11188532	G	C
8:11188540:A:G	8:11188540	8	11188540	G	A
8:11188586:C:T	8:11188586	8	11188586	C	T
8:11188752:A:G	8:11188752	8	11188752	A	G
8:11189488:C:T	8:11189488	8	11189488	C	T
8:11189535:C:T	rs12681991	8	11189535	T	C
8:11190647:C:G	8:11190647	8	11190647	C	G
8:11191537:C:T	8:11191537	8	11191537	C	T
8:11192551:C:G	8:11192551	8	11192551	C	G
8:11192593:A:G	8:11192593	8	11192593	A	G
8:11193530:C:T	8:11193530	8	11193530	T	C
8:11193736:G:T	8:11193736	8	11193736	G	T
8:11194457:A:G	8:11194457	8	11194457	A	G
8:11194911:A:G	8:11194911	8	11194911	G	A

uniqID	*rsID*	*chr*	*pos*	*non_effect allele*	*effect_allele*
8:11196295:C:G	8:11196295	8	11196295	G	C
8:11196970:C:T	8:11196970	8	11196970	T	C
8:11197301:C:G	8:11197301	8	11197301	C	G
8:11197323:G:T	8:11197323	8	11197323	G	T
8:11197598:C:T	rs6601581	8	11197598	T	C
8:11198579:C:CTTTT	rs143472078	8	11198579	CTTTT	C
8:11198792:A:G	8:11198792	8	11198792	G	A
8:11199584:A:G	8:11199584	8	11199584	G	A
8:11199938:A:C	8:11199938	8	11199938	A	C
8:11200454:A:AAT	rs34785978	8	11200454	AAT	A
8:11201504:G:T	8:11201504	8	11201504	G	T
8:11201605:A:ATT	rs370296579	8	11201605	ATT	A
8:11202154:A:G	8:11202154	8	11202154	A	G
8:11202960:A:G	8:11202960	8	11202960	A	G
8:11203107:A:T	8:11203107	8	11203107	T	A
8:11204165:C:T	8:11204165	8	11204165	T	C
8:11204184:A:G	8:11204184	8	11204184	A	G
8:11204503:C:G	8:11204503	8	11204503	C	G
8:11204532:A:G	8:11204532	8	11204532	G	A
8:11204755:C:T	8:11204755	8	11204755	C	T
8:11205593:C:T	8:11205593	8	11205593	C	T
8:11205602:C:T	8:11205602	8	11205602	C	T
8:11205654:A:T	8:11205654	8	11205654	A	T
8:11205665:C:G	8:11205665	8	11205665	C	G
8:11205817:T:TA	rs35291885	8	11205817	TA	T
8:11206220:C:G	8:11206220	8	11206220	G	C
8:11206262:C:T	8:11206262	8	11206262	C	T
8:11206363:C:G	8:11206363	8	11206363	G	C
8:11206543:A:G	8:11206543	8	11206543	G	A
8:11206627:C:G	8:11206627	8	11206627	G	C
8:11207326:A:C	8:11207326	8	11207326	A	C
8:11207367:A:C	8:11207367	8	11207367	A	C

uniqID	*rsID*	*chr*	*pos*	*non_effect allele*	*effect_allele*
8:11207431:C:T	8:11207431	8	11207431	T	C
8:11207508:C:T	8:11207508	8	11207508	C	T
8:11207672:C:G	8:11207672	8	11207672	G	C
8:11208903:A:G	8:11208903	8	11208903	A	G
8:11209499:C:T	8:11209499	8	11209499	C	T
8:11210823:G:T	8:11210823	8	11210823	T	G
8:11210824:G:T	8:11210824	8	11210824	T	G
8:11210828:C:T	8:11210828	8	11210828	C	T
8:11210983:A:G	8:11210983	8	11210983	G	A
8:11211068:C:T	8:11211068	8	11211068	C	T
8:11211302:G:T	8:11211302	8	11211302	G	T
8:11212081:A:G	8:11212081	8	11212081	G	A
8:11212599:C:T	8:11212599	8	11212599	T	C
8:11212650:A:AT	rs3021507	8	11212650	A	AT
8:11212778:C:T	8:11212778	8	11212778	T	C
8:11212811:A:C	8:11212811	8	11212811	A	C
8:11212812:C:G	8:11212812	8	11212812	G	C
8:11212875:C:G	8:11212875	8	11212875	C	G
8:11213092:A:G	8:11213092	8	11213092	G	A
8:11213250:C:T	8:11213250	8	11213250	T	C
8:11213363:C:T	8:11213363	8	11213363	T	C
8:11213389:C:T	8:11213389	8	11213389	T	C
8:11213589:G:T	8:11213589	8	11213589	G	T
8:11213881:C:G	8:11213881	8	11213881	C	G
8:11214455:A:G	8:11214455	8	11214455	G	A
8:11214972:A:G	8:11214972	8	11214972	G	A
8:11215617:C:G	8:11215617	8	11215617	G	C
8:11215868:A:AT	rs34369083	8	11215868	AT	A
8:11216761:G:T	8:11216761	8	11216761	G	T
8:11217284:C:T	8:11217284	8	11217284	T	C
8:11217441:A:G	8:11217441	8	11217441	G	A
8:11218893:A:G	8:11218893	8	11218893	G	A

uniqID	*rsID*	*chr*	*pos*	*non_effect allele*	*effect_allele*
8:11219334:A:G	8:11219334	8	11219334	A	G
8:11219386:G:T	8:11219386	8	11219386	T	G
8:11219781:C:T	8:11219781	8	11219781	T	C
8:11220846:C:CAA	rs140232751	8	11220846	C	CAA
8:11221313:C:T	8:11221313	8	11221313	T	C
8:11223022:A:C	8:11223022	8	11223022	A	C
8:11223793:G:T	8:11223793	8	11223793	G	T
8:11224313:C:T	8:11224313	8	11224313	T	C
8:11225168:A:G	8:11225168	8	11225168	A	G
8:11225480:C:T	8:11225480	8	11225480	C	T
8:11225910:A:G	8:11225910	8	11225910	G	A
8:11226071:A:G	8:11226071	8	11226071	G	A
8:11226456:A:C	8:11226456	8	11226456	A	C
8:11227104:A:G	8:11227104	8	11227104	G	A
8:11227406:A:G	8:11227406	8	11227406	A	G
8:11227885:C:T	8:11227885	8	11227885	T	C
8:11228006:A:G	8:11228006	8	11228006	A	G
8:11228100:A:G	8:11228100	8	11228100	A	G
8:11228254:A:G	8:11228254	8	11228254	A	G
8:11228672:A:G	8:11228672	8	11228672	A	G
8:11229319:C:G	8:11229319	8	11229319	G	C
8:11229638:C:T	8:11229638	8	11229638	T	C
8:11229889:A:G	8:11229889	8	11229889	G	A
8:11230206:G:T	8:11230206	8	11230206	T	G
8:11230259:A:T	8:11230259	8	11230259	T	A
8:11230574:A:G	8:11230574	8	11230574	A	G
8:11231249:C:T	8:11231249	8	11231249	C	T
8:11231354:G:T	8:11231354	8	11231354	T	G
8:11231886:A:G	8:11231886	8	11231886	G	A
8:11232343:C:G	8:11232343	8	11232343	G	C
8:11232788:A:G	8:11232788	8	11232788	A	G
8:11232860:A:G	8:11232860	8	11232860	A	G

uniqID	*rsID*	*chr*	*pos*	*non_effect allele*	*effect_allele*
8:11233318:A:C	8:11233318	8	11233318	C	A
8:11233419:G:T	8:11233419	8	11233419	G	T
8:11233582:A:G	8:11233582	8	11233582	G	A
8:11233659:A:G	8:11233659	8	11233659	G	A
8:11233917:C:T	8:11233917	8	11233917	T	C
8:11233958:A:G	8:11233958	8	11233958	G	A
8:11234298:C:T	8:11234298	8	11234298	T	C
8:11234367:G:T	8:11234367	8	11234367	G	T
8:11234500:A:T	8:11234500	8	11234500	T	A
8:11234520:A:G	8:11234520	8	11234520	G	A
8:11234613:A:T	8:11234613	8	11234613	A	T
8:11234626:C:T	8:11234626	8	11234626	C	T
8:11234780:C:G	8:11234780	8	11234780	G	C
8:11234844:A:G	8:11234844	8	11234844	A	G
8:11234885:C:G	8:11234885	8	11234885	G	C
8:11235136:C:G	8:11235136	8	11235136	G	C
8:11235150:A:C	8:11235150	8	11235150	A	C
8:11235360:A:C	8:11235360	8	11235360	C	A
8:11235393:A:G	8:11235393	8	11235393	G	A
8:11235497:A:G	8:11235497	8	11235497	A	G
8:11235579:G:T	8:11235579	8	11235579	G	T
8:11235605:G:T	8:11235605	8	11235605	G	T
8:11235614:A:G	8:11235614	8	11235614	A	G
8:11235735:C:T	8:11235735	8	11235735	C	T
8:11235910:A:G	8:11235910	8	11235910	A	G
8:11236392:C:T	8:11236392	8	11236392	C	T
8:11236413:C:T	8:11236413	8	11236413	T	C
8:11236419:C:T	8:11236419	8	11236419	C	T
8:11236572:A:T	8:11236572	8	11236572	A	T
8:11236681:G:T	8:11236681	8	11236681	G	T
8:11236685:C:T	8:11236685	8	11236685	T	C
8:11236809:C:T	8:11236809	8	11236809	C	T

uniqID	*rsID*	*chr*	*pos*	*non_effect allele*	*effect_allele*
8:11236850:C:T	8:11236850	8	11236850	T	C
8:11236964:C:T	8:11236964	8	11236964	T	C
8:11236975:A:G	8:11236975	8	11236975	G	A
8:11237330:C:G	8:11237330	8	11237330	G	C
8:11237477:A:G	8:11237477	8	11237477	G	A
8:11237480:A:G	8:11237480	8	11237480	G	A
8:11237587:C:CAAAA	rs55683451	8	11237587	CAAAA	C
8:11237591:A:C	8:11237591	8	11237591	C	A
8:11237756:A:C	8:11237756	8	11237756	A	C
8:11237773:C:T	8:11237773	8	11237773	C	T
8:11238029:C:T	8:11238029	8	11238029	T	C
8:11238315:C:T	8:11238315	8	11238315	T	C
8:11238316:G:T	8:11238316	8	11238316	G	T
8:11238332:G:T	8:11238332	8	11238332	G	T
8:11238587:A:G	8:11238587	8	11238587	G	A
8:11238597:C:T	8:11238597	8	11238597	T	C
8:11239017:A:T	8:11239017	8	11239017	A	T
8:11239054:C:T	8:11239054	8	11239054	T	C
8:11239078:G:T	8:11239078	8	11239078	G	T
8:11239137:A:G	8:11239137	8	11239137	A	G
8:11239297:G:GATAG	1239297:G:GAT[illegible]	8	11239297	G	GATAG
8:11239297:G:GATAT	1239297:G:GAT	8	11239297	G	GATAT
8:11239352:A:G	8:11239352	8	11239352	A	G
8:11239510:A:G	8:11239510	8	11239510	A	G
8:11239565:C:T	8:11239565	8	11239565	T	C
8:11239640:C:T	8:11239640	8	11239640	T	C
8:11239762:A:T	8:11239762	8	11239762	T	A
8:11239942:C:CT	rs35106439	8	11239942	C	CT
8:11240571:C:T	8:11240571	8	11240571	T	C
8:11241935:G:GT	rs34637859	8	11241935	GT	G
8:11242025:G:T	8:11242025	8	11242025	G	T
8:11242039:T:TA	rs35786152	8	11242039	T	TA

uniqID	*rsID*	*chr*	*pos*	*non_effect allele*	*effect_allele*
8:11242632:A:G	8:11242632	8	11242632	A	G
8:11243126:C:G	8:11243126	8	11243126	G	C
8:11244841:A:G	8:11244841	8	11244841	A	G
8:11245064:C:T	8:11245064	8	11245064	T	C
8:11245303:C:G	8:11245303	8	11245303	G	C
8:11245562:A:T	rs73534344	8	11245562	A	T
8:11247298:A:G	8:11247298	8	11247298	A	G
8:11247814:A:G	8:11247814	8	11247814	A	G
8:11248500:C:T	8:11248500	8	11248500	T	C
8:11248956:C:T	8:11248956	8	11248956	T	C
8:11249010:G:T	8:11249010	8	11249010	T	G
8:11249261:C:T	8:11249261	8	11249261	C	T
8:11250848:A:C	8:11250848	8	11250848	C	A
8:11251175:A:G	8:11251175	8	11251175	G	A
8:11251705:A:G	8:11251705	8	11251705	A	G
8:11252170:C:T	8:11252170	8	11252170	T	C
8:11252425:A:C	8:11252425	8	11252425	A	C
8:11309192:C:T	8:11309192	8	11309192	C	T
8:11336781:A:G	8:11336781	8	11336781	A	G
8:11338146:A:G	8:11338146	8	11338146	G	A
8:11355602:C:G	8:11355602	8	11355602	G	C
8:11358156:C:T	8:11358156	8	11358156	C	T
8:11361261:C:G	8:11361261	8	11361261	G	C
8:11361850:G:GAGGA	rs200525228	8	11361850	G	GAGGA
8:11362275:A:C	8:11362275	8	11362275	A	C
8:11362277:G:T	8:11362277	8	11362277	T	G
8:11382367:A:G	8:11382367	8	11382367	A	G
8:11384556:C:T	8:11384556	8	11384556	T	C
8:11392093:A:C	8:11392093	8	11392093	C	A
8:11393764:A:G	8:11393764	8	11393764	G	A
8:11395079:A:G	8:11395079	8	11395079	A	G
8:11396856:A:C	8:11396856	8	11396856	C	A

uniqID	*rsID*	*chr*	*pos*	*non_effect allele*	*effect_allele*
8:11396874:A:G	8:11396874	8	11396874	G	A
8:11397073:A:C	8:11397073	8	11397073	C	A
8:11397086:A:T	8:11397086	8	11397086	A	T
8:11397457:C:G	8:11397457	8	11397457	G	C
8:11398865:A:G	8:11398865	8	11398865	G	A
8:11398953:C:T	8:11398953	8	11398953	C	T
8:11399484:A:T	8:11399484	8	11399484	T	A
8:11400628:C:G	8:11400628	8	11400628	G	C
8:11400680:A:G	8:11400680	8	11400680	A	G
8:11400944:G:T	8:11400944	8	11400944	T	G
8:11401116:A:G	8:11401116	8	11401116	A	G
8:11402347:A:G	8:11402347	8	11402347	A	G
8:11410513:A:C	8:11410513	8	11410513	A	C
8:11411005:C:G	8:11411005	8	11411005	C	G
8:11415184:C:G	8:11415184	8	11415184	G	C
8:11415572:A:G	8:11415572	8	11415572	G	A
8:11415597:C:T	8:11415597	8	11415597	T	C
8:11415794:A:T	8:11415794	8	11415794	A	T
8:11415812:C:T	8:11415812	8	11415812	T	C
8:11416171:C:T	8:11416171	8	11416171	C	T
8:11416428:C:T	8:11416428	8	11416428	T	C
8:11416635:A:G	8:11416635	8	11416635	A	G
8:11416885:C:T	8:11416885	8	11416885	T	C
8:11417016:A:ACT	rs35482693	8	11417016	A	ACT
8:11417144:C:T	8:11417144	8	11417144	C	T
8:11417150:G:T	8:11417150	8	11417150	T	G
8:11417257:A:G	8:11417257	8	11417257	G	A
8:11417493:C:T	8:11417493	8	11417493	C	T
8:11417582:C:T	8:11417582	8	11417582	T	C
8:11418385:A:G	8:11418385	8	11418385	A	G
8:11418773:C:T	8:11418773	8	11418773	T	C
8:11419335:A:AT	rs375515340	8	11419335	A	AT

uniqID	*rsID*	*chr*	*pos*	*non_effect allele*	*effect_allele*
8:11419852:C:T	8:11419852	8	11419852	C	T
8:11419861:G:T	8:11419861	8	11419861	G	T
8:11420104:C:G	8:11420104	8	11420104	G	C
8:11420221:A:G	8:11420221	8	11420221	A	G
8:11420295:C:T	8:11420295	8	11420295	C	T
8:11421016:A:G	8:11421016	8	11421016	A	G
8:11421358:A:C	8:11421358	8	11421358	A	C
8:11421384:C:T	8:11421384	8	11421384	T	C
8:11421793:C:T	8:11421793	8	11421793	T	C
8:11422045:A:G	8:11422045	8	11422045	A	G
8:11422130:C:T	8:11422130	8	11422130	C	T
8:11422170:A:G	8:11422170	8	11422170	G	A
8:11422289:C:G	8:11422289	8	11422289	C	G
8:11422442:A:C	8:11422442	8	11422442	A	C
8:11422491:A:C	rs7823100	8	11422491	A	C
8:11422492:C:G	rs7823101	8	11422492	G	C
8:11422494:A:AT	rs34765125	8	11422494	AT	A
8:11422521:G:GA	rs34883095	8	11422521	GA	G
8:11422861:C:G	8:11422861	8	11422861	G	C
8:11422936:C:T	8:11422936	8	11422936	T	C
8:11423072:A:G	8:11423072	8	11423072	G	A
8:11423083:A:C	8:11423083	8	11423083	C	A
8:11423142:A:G	8:11423142	8	11423142	G	A
8:11423434:A:G	8:11423434	8	11423434	G	A
8:11423537:A:G	8:11423537	8	11423537	A	G
8:11423781:C:G	8:11423781	8	11423781	G	C
8:11425077:C:T	8:11425077	8	11425077	T	C
8:11425081:G:T	8:11425081	8	11425081	T	G
8:11425105:G:T	8:11425105	8	11425105	T	G
8:11425809:G:T	8:11425809	8	11425809	G	T
8:11426400:G:T	8:11426400	8	11426400	G	T
8:11426790:C:G	8:11426790	8	11426790	C	G

uniqID	*rsID*	*chr*	*pos*	*non_effect allele*	*effect_allele*
8:11427133:G:T	8:11427133	8	11427133	T	G
8:11427341:G:T	8:11427341	8	11427341	G	T
8:11427637:A:T	8:11427637	8	11427637	T	A
8:11428395:C:T	8:11428395	8	11428395	T	C
8:11430485:A:G	8:11430485	8	11430485	A	G
8:11430990:C:T	8:11430990	8	11430990	T	C
8:11431558:T:TAC	rs35580513	8	11431558	T	TAC
8:11431943:T:TAA	rs560993129	8	11431943	T	TAA
8:11432085:A:ACG	:11432085:A:AC	8	11432085	ACG	A
8:11432085:A:ACACG	1432085:A:ACA	8	11432085	A	ACACG
8:11432438:C:G	8:11432438	8	11432438	G	C
8:11432453:C:T	8:11432453	8	11432453	T	C
8:11432946:A:C	8:11432946	8	11432946	A	C
8:11433780:C:T	8:11433780	8	11433780	C	T
8:11433909:C:T	8:11433909	8	11433909	C	T
8:11434176:A:T	8:11434176	8	11434176	T	A
8:11434232:C:T	8:11434232	8	11434232	T	C
8:11434415:C:CTCGGTTT	rs111613945	8	11434415	CTCGGTTT	C
8:11434792:C:G	8:11434792	8	11434792	G	C
8:11434929:C:T	8:11434929	8	11434929	T	C
8:11435049:A:G	8:11435049	8	11435049	A	G
8:11435291:G:GTGGC	rs140471203	8	11435291	GTGGC	G
8:11435516:C:T	8:11435516	8	11435516	C	T
8:11435564:C:T	8:11435564	8	11435564	T	C
8:11435927:C:G	8:11435927	8	11435927	C	G
8:11438064:G:T	8:11438064	8	11438064	T	G
8:11439225:A:G	8:11439225	8	11439225	A	G
8:11440019:A:G	8:11440019	8	11440019	A	G
8:11444516:A:G	8:11444516	8	11444516	A	G
8:11444837:C:T	8:11444837	8	11444837	T	C
8:11446421:A:G	8:11446421	8	11446421	A	G
8:11446637:A:G	8:11446637	8	11446637	A	G

uniqID	*rsID*	*chr*	*pos*	*non_effect allele*	*effect_allele*
8:11446652:A:G	rs13255662	8	11446652	G	A
8:11446680:C:T	8:11446680	8	11446680	C	T
8:11446800:C:T	8:11446800	8	11446800	T	C
8:11446868:C:T	8:11446868	8	11446868	C	T
8:11446955:A:G	8:11446955	8	11446955	A	G
8:11447093:A:G	8:11447093	8	11447093	A	G
8:11447119:C:T	8:11447119	8	11447119	T	C
8:11447679:C:G	8:11447679	8	11447679	C	G
8:11448659:C:G	8:11448659	8	11448659	C	G
8:11450133:A:G	8:11450133	8	11450133	A	G
8:11450422:A:G	8:11450422	8	11450422	G	A
8:11450587:G:T	8:11450587	8	11450587	G	T
8:11460909:T:TA	rs34394602	8	11460909	T	TA
8:11461111:A:G	8:11461111	8	11461111	G	A
8:11466745:A:T	8:11466745	8	11466745	T	A
8:11467557:C:G	8:11467557	8	11467557	G	C
12:62830952:C:T	12:62830952	12	62830952	T	C
12:62831343:C:T	12:62831343	12	62831343	C	T
12:62837767:A:G	12:62837767	12	62837767	A	G
12:62847085:C:G	12:62847085	12	62847085	G	C
12:62848152:A:G	12:62848152	12	62848152	A	G
12:62849418:A:G	12:62849418	12	62849418	A	G
12:62851080:A:AC	rs36039527	12	62851080	AC	A
12:62852271:A:G	12:62852271	12	62852271	A	G
12:62852916:A:G	12:62852916	12	62852916	A	G
12:62855388:C:T	12:62855388	12	62855388	T	C
12:62858342:A:G	12:62858342	12	62858342	A	G
12:62858561:C:T	12:62858561	12	62858561	T	C
12:62859241:A:AC	rs35579550	12	62859241	AC	A
12:62861935:T:TA	rs35738119	12	62861935	TA	T
12:62862739:G:T	12:62862739	12	62862739	G	T
12:62865152:C:T	12:62865152	12	62865152	T	C

uniqID	*rsID*	*chr*	*pos*	*non_effect allele*	*effect_allele*
12:62865291:A:G	12:62865291	12	62865291	A	G
12:62868455:C:T	12:62868455	12	62868455	T	C
12:62868499:C:T	12:62868499	12	62868499	C	T
12:62868500:A:C	12:62868500	12	62868500	C	A
12:62870574:C:G	12:62870574	12	62870574	G	C
12:62875839:C:T	12:62875839	12	62875839	T	C
12:62880276:C:G	12:62880276	12	62880276	G	C
12:62884714:A:ATTT	rs34591945	12	62884714	ATTT	A
12:62884972:A:G	12:62884972	12	62884972	A	G
12:62886001:A:T	12:62886001	12	62886001	A	T
12:62886372:C:G	12:62886372	12	62886372	C	G
12:62886649:C:T	12:62886649	12	62886649	T	C
12:62888219:A:C	12:62888219	12	62888219	C	A
12:62889402:A:G	12:62889402	12	62889402	G	A
12:62889409:A:G	12:62889409	12	62889409	A	G
12:62890491:A:C	12:62890491	12	62890491	A	C
12:62891828:C:CT	rs34160326	12	62891828	CT	C
12:62894058:A:G	12:62894058	12	62894058	G	A
12:62897930:A:G	12:62897930	12	62897930	A	G
12:62898111:A:G	12:62898111	12	62898111	A	G
12:62898463:C:CT	rs75092735	12	62898463	C	CT
12:62898490:C:T	12:62898490	12	62898490	T	C
12:62902420:C:T	12:62902420	12	62902420	T	C
12:62903639:A:G	12:62903639	12	62903639	G	A
12:62903655:G:GT	rs574068633	12	62903655	G	GT
12:62903793:A:G	12:62903793	12	62903793	G	A
12:62904252:C:T	12:62904252	12	62904252	T	C
12:62905380:C:CA	rs35153665	12	62905380	CA	C
12:62905772:A:AC	rs35759179	12	62905772	A	AC
12:62906488:A:G	12:62906488	12	62906488	A	G
12:62910714:A:T	12:62910714	12	62910714	T	A
12:62914776:T:TA	rs71086606	12	62914776	TA	T

uniqID	*rsID*	*chr*	*pos*	*non_effect allele*	*effect_allele*
12:62917704:A:G	12:62917704	12	62917704	G	A
12:62920860:C:G	12:62920860	12	62920860	G	C
12:62921257:C:T	12:62921257	12	62921257	T	C
12:62922143:A:G	12:62922143	12	62922143	A	G
12:62926398:A:G	12:62926398	12	62926398	A	G
12:62928006:T:TA	rs545076659	12	62928006	T	TA
12:62928633:A:G	12:62928633	12	62928633	A	G
12:62930621:A:G	12:62930621	12	62930621	A	G
12:62930798:C:T	12:62930798	12	62930798	T	C
12:62931846:A:ATTTGT	rs147032464	12	62931846	ATTTGT	A
12:62932816:A:G	12:62932816	12	62932816	G	A
12:62935154:C:CA	rs531553553	12	62935154	CA	C
12:62935705:C:T	12:62935705	12	62935705	T	C
12:62937348:G:T	12:62937348	12	62937348	G	T
12:62937532:C:T	12:62937532	12	62937532	T	C
12:62939055:C:T	12:62939055	12	62939055	C	T
12:62941426:C:T	12:62941426	12	62941426	C	T
12:62941444:A:G	12:62941444	12	62941444	G	A
12:62941837:A:G	12:62941837	12	62941837	G	A
12:62941929:A:T	12:62941929	12	62941929	T	A
12:62942258:C:T	12:62942258	12	62942258	T	C
12:62945158:G:GGAAACAACCTGACGAT	rs113510588	12	62945158	GGAAACAACCTGACGAT	G
12:62945245:A:G	12:62945245	12	62945245	A	G
12:62945970:C:G	12:62945970	12	62945970	G	C
12:62948736:A:G	12:62948736	12	62948736	A	G
12:62949110:A:C	12:62949110	12	62949110	C	A
12:62950556:C:G	12:62950556	12	62950556	C	G
12:62952696:C:T	12:62952696	12	62952696	T	C
12:62952910:C:T	12:62952910	12	62952910	T	C
12:62957906:C:G	12:62957906	12	62957906	C	G
12:62960880:A:G	12:62960880	12	62960880	G	A
12:62961299:C:T	12:62961299	12	62961299	T	C

uniqID	*rsID*	*chr*	*pos*	*non_effect allele*	*effect_allele*
12:62961801:A:G	12:62961801	12	62961801	A	G
12:62964552:C:T	12:62964552	12	62964552	C	T
12:62968280:C:T	12:62968280	12	62968280	C	T
12:62968740:C:CA	rs11301095	12	62968740	CA	C
12:62970482:A:G	12:62970482	12	62970482	G	A
12:62972210:G:GT	rs555030366	12	62972210	G	GT
12:62975307:C:T	12:62975307	12	62975307	T	C
12:62977162:A:G	12:62977162	12	62977162	A	G
12:62977163:A:G	12:62977163	12	62977163	A	G
12:62977175:A:AG	rs34394452	12	62977175	AG	A
12:62980580:A:T	12:62980580	12	62980580	A	T
12:62982152:C:CT	rs566612223	12	62982152	C	CT
12:62985871:A:C	12:62985871	12	62985871	A	C
12:62986620:A:G	12:62986620	12	62986620	A	G
12:62988288:A:G	12:62988288	12	62988288	A	G
12:62989110:A:C	12:62989110	12	62989110	C	A
12:62990403:A:G	12:62990403	12	62990403	G	A
12:62990415:C:T	12:62990415	12	62990415	C	T
12:62990871:A:C	12:62990871	12	62990871	A	C
12:62992896:G:GA	rs34135257	12	62992896	GA	G
12:62993793:A:G	12:62993793	12	62993793	A	G
12:62995269:A:G	12:62995269	12	62995269	G	A
12:62995340:A:G	12:62995340	12	62995340	A	G
12:62995984:C:T	12:62995984	12	62995984	T	C
12:62996061:A:G	12:62996061	12	62996061	A	G
12:62997180:C:T	12:62997180	12	62997180	C	T
12:62999154:C:G	12:62999154	12	62999154	G	C
12:63001068:A:AT	rs35168986	12	63001068	AT	A
13:83298475:A:G	13:83298475	13	83298475	A	G
13:83299269:C:T	13:83299269	13	83299269	C	T
13:83300084:A:G	rs9575156	13	83300084	G	A
13:83300138:A:AGAGAGC	rs563056680	13	83300138	AGAGAGC	A

uniqID	*rsID*	*chr*	*pos*	*non_effect allele*	*effect_allele*
13:83300269:C:T	13:83300269	13	83300269	T	C
13:83300359:A:G	13:83300359	13	83300359	G	A
13:83302594:C:T	13:83302594	13	83302594	T	C
13:83302795:A:T	13:83302795	13	83302795	T	A
13:83304274:C:T	13:83304274	13	83304274	C	T
13:83304898:G:T	13:83304898	13	83304898	G	T
13:83306586:A:G	13:83306586	13	83306586	A	G
13:83311141:A:G	13:83311141	13	83311141	A	G
13:83312341:C:T	13:83312341	13	83312341	T	C
13:83312717:C:G	13:83312717	13	83312717	C	G
16:13021889:C:T	16:13021889	16	13021889	C	T
16:13022033:A:AT	rs11361092	16	13022033	A	AT
16:13023207:C:T	16:13023207	16	13023207	T	C
16:13023388:A:G	16:13023388	16	13023388	G	A
16:13023394:A:G	16:13023394	16	13023394	G	A
16:13024150:A:G	16:13024150	16	13024150	G	A
16:13025315:G:T	16:13025315	16	13025315	G	T
16:13026502:A:G	16:13026502	16	13026502	A	G
16:13029711:G:GA	rs11428944	16	13029711	GA	G
16:13030222:A:G	16:13030222	16	13030222	G	A
16:13030875:A:G	16:13030875	16	13030875	G	A
16:13031195:A:C	16:13031195	16	13031195	C	A
16:13032351:C:G	16:13032351	16	13032351	G	C
16:13032547:A:G	16:13032547	16	13032547	G	A
16:13032863:A:T	16:13032863	16	13032863	A	T
16:13035206:T:TA	rs34236666	16	13035206	TA	T
16:13036811:C:T	16:13036811	16	13036811	T	C
16:13037305:C:T	16:13037305	16	13037305	C	T
16:13038054:G:T	16:13038054	16	13038054	T	G
16:13038196:A:G	16:13038196	16	13038196	G	A
16:13038723:G:T	16:13038723	16	13038723	G	T
16:13039154:C:T	16:13039154	16	13039154	T	C

uniqID	*rsID*	*chr*	*pos*	*non_effect allele*	*effect_allele*
16:13039642:A:T	16:13039642	16	13039642	A	T
16:13039646:C:T	16:13039646	16	13039646	T	C
16:13040514:C:T	16:13040514	16	13040514	T	C
16:13040889:C:G	16:13040889	16	13040889	G	C
16:13041027:G:T	16:13041027	16	13041027	T	G
16:13041921:C:T	16:13041921	16	13041921	C	T
16:13041924:A:G	16:13041924	16	13041924	G	A
16:13041940:A:C	16:13041940	16	13041940	A	C
16:13042097:A:G	16:13042097	16	13042097	A	G
16:13043452:A:G	16:13043452	16	13043452	G	A
16:13044033:C:T	16:13044033	16	13044033	C	T
16:13044827:C:T	16:13044827	16	13044827	C	T
16:13045117:C:CA	rs113668400	16	13045117	CA	C
16:13047022:C:G	16:13047022	16	13047022	G	C
16:13047598:C:G	16:13047598	16	13047598	C	G
16:13047734:C:T	16:13047734	16	13047734	C	T
16:13048395:C:T	16:13048395	16	13048395	C	T
16:13048841:C:T	16:13048841	16	13048841	T	C
16:13048916:G:GT	rs527441820	16	13048916	G	GT
16:13049490:T:TA	rs571415029	16	13049490	T	TA
16:13049557:C:T	16:13049557	16	13049557	T	C
16:13049749:A:C	16:13049749	16	13049749	C	A
16:13049906:A:T	16:13049906	16	13049906	T	A
16:13050340:A:C	16:13050340	16	13050340	A	C
16:13051686:A:C	16:13051686	16	13051686	A	C
16:13052050:A:G	16:13052050	16	13052050	A	G
16:13052714:C:G	16:13052714	16	13052714	G	C
16:13052846:A:G	16:13052846	16	13052846	G	A
16:13053072:A:G	16:13053072	16	13053072	G	A
16:13053136:G:T	16:13053136	16	13053136	T	G
16:13053140:C:G	16:13053140	16	13053140	G	C
16:13053187:C:T	16:13053187	16	13053187	C	T

uniqID	*rsID*	*chr*	*pos*	*non_effect allele*	*effect_allele*
16:13053270:A:G	16:13053270	16	13053270	G	A
16:13053457:A:AC	rs11430580	16	13053457	A	AC
16:13053576:A:T	16:13053576	16	13053576	T	A
16:13053882:A:G	16:13053882	16	13053882	A	G
16:13053924:C:G	16:13053924	16	13053924	G	C
16:13053951:C:T	16:13053951	16	13053951	T	C
16:13054037:C:G	16:13054037	16	13054037	G	C
16:13054265:A:G	16:13054265	16	13054265	A	G
16:13054645:C:T	16:13054645	16	13054645	C	T
16:13054883:C:G	16:13054883	16	13054883	C	G
16:13054897:A:C	16:13054897	16	13054897	C	A
16:13055220:C:T	16:13055220	16	13055220	T	C
16:13055621:T:TCC	rs35005963	16	13055621	T	TCC
16:13055766:C:T	16:13055766	16	13055766	T	C
16:13057200:A:G	16:13057200	16	13057200	A	G
16:13058241:G:GT	rs566468808	16	13058241	G	GT
16:13059539:C:T	16:13059539	16	13059539	C	T
16:13061109:A:G	16:13061109	16	13061109	G	A
16:13062232:A:G	16:13062232	16	13062232	A	G
16:13066833:C:T	16:13066833	16	13066833	T	C
16:13070238:A:G	16:13070238	16	13070238	G	A
16:13070671:C:T	16:13070671	16	13070671	T	C
16:13070809:C:G	16:13070809	16	13070809	G	C
16:13075745:A:G	16:13075745	16	13075745	G	A
16:13077351:C:T	16:13077351	16	13077351	C	T
16:13077901:A:G	16:13077901	16	13077901	A	G
16:13077915:A:G	16:13077915	16	13077915	A	G
16:13078044:A:T	16:13078044	16	13078044	A	T
16:13078807:C:T	16:13078807	16	13078807	T	C
16:13079063:C:CTT	rs3075030	16	13079063	C	CTT
16:13079082:A:C	16:13079082	16	13079082	C	A
16:13079214:A:G	16:13079214	16	13079214	G	A

uniqID	*rsID*	*chr*	*pos*	*non_effect allele*	*effect_allele*
16:13079389:G:T	16:13079389	16	13079389	T	G
16:13079463:C:T	16:13079463	16	13079463	C	T
16:13079698:A:T	16:13079698	16	13079698	T	A
16:13080252:A:G	16:13080252	16	13080252	A	G
16:13080515:C:T	16:13080515	16	13080515	T	C
16:13081923:C:G	16:13081923	16	13081923	G	C
16:13082445:A:G	16:13082445	16	13082445	A	G
16:13083055:G:T	16:13083055	16	13083055	G	T
16:13083068:G:GT	rs71147777	16	13083068	G	GT
16:13086810:C:T	16:13086810	16	13086810	T	C
16:13088905:C:T	16:13088905	16	13088905	C	T
16:13089059:A:G	16:13089059	16	13089059	A	G
16:13089854:G:T	16:13089854	16	13089854	G	T
16:13091332:C:T	16:13091332	16	13091332	T	C
16:13092100:A:G	16:13092100	16	13092100	G	A
16:13092220:C:T	16:13092220	16	13092220	T	C
16:13092663:C:T	16:13092663	16	13092663	C	T
16:13093774:G:T	16:13093774	16	13093774	T	G
16:13093778:C:T	16:13093778	16	13093778	T	C
16:13093858:C:T	16:13093858	16	13093858	T	C
16:13094769:G:T	16:13094769	16	13094769	G	T
16:13094897:C:G	16:13094897	16	13094897	C	G
16:13095142:C:G	16:13095142	16	13095142	G	C
16:13095171:C:T	16:13095171	16	13095171	T	C
16:13095296:A:T	16:13095296	16	13095296	T	A
16:13095739:A:C	16:13095739	16	13095739	C	A
16:13096300:C:T	16:13096300	16	13096300	T	C
16:13097084:G:T	16:13097084	16	13097084	T	G
16:13097125:A:G	16:13097125	16	13097125	G	A
16:13097206:C:G	16:13097206	16	13097206	G	C
16:13097746:G:T	rs60560408	16	13097746	G	T
16:13097749:G:T	rs8050866	16	13097749	G	T

uniqID	*rsID*	*chr*	*pos*	*non_effect allele*	*effect_allele*
16:13098440:C:G	16:13098440	16	13098440	C	G
16:13098508:A:G	16:13098508	16	13098508	A	G
16:13098762:A:C	16:13098762	16	13098762	A	C
16:13098934:A:G	16:13098934	16	13098934	A	G
16:13099114:C:G	16:13099114	16	13099114	C	G
16:13099177:G:T	16:13099177	16	13099177	T	G
16:13099919:A:G	16:13099919	16	13099919	A	G
16:13099953:G:T	16:13099953	16	13099953	G	T
16:13100021:C:T	16:13100021	16	13100021	C	T
16:13101555:C:T	16:13101555	16	13101555	C	T
16:13101618:A:G	16:13101618	16	13101618	A	G
16:13102532:C:T	rs4781381	16	13102532	C	T
16:13102906:A:T	16:13102906	16	13102906	T	A
16:13104924:C:T	16:13104924	16	13104924	T	C
16:13105091:A:C	16:13105091	16	13105091	A	C
16:13105863:C:T	16:13105863	16	13105863	T	C
16:13105998:C:T	16:13105998	16	13105998	C	T
16:13109594:G:T	16:13109594	16	13109594	T	G
16:13112335:C:T	16:13112335	16	13112335	T	C
16:13115702:C:G	16:13115702	16	13115702	C	G
16:13115755:A:G	16:13115755	16	13115755	G	A
16:13116871:T:TTTTA	rs144610339	16	13116871	TTTTA	T
16:13118299:A:T	16:13118299	16	13118299	A	T

Abbreviations: chr = chromosome; pos = position; MAF = minor allele frequency; gwasP = p-value GWAS; se = standard error; r2 = across 127 tissue/cell type; commonChrState = most common 15-core chromatin state across 127 tissue/cell types; Filt = filter

Table S6: Gene mapping FUMA

uniqID	*MAF*	*gwasP*	*beta*	*se*	*r2*	*IndSigSNP*
2:98325330:G:T	0.4245	4.78E-06	-0.00668	0.001459	0.710014	2:98580012
2:98326867:A:G	0.4245	6.08E-06	0.005863	0.001295	0.710014	2:98580012
2:98330052:A:C	0.4235	9.06E-06	0.005754	0.001295	0.713051	2:98580012
2:98334679:C:T	0.3638	4.54E-05	0.005509	0.00135	0.799332	2:98580012
2:98336200:A:C	0.4066	5.87E-07	0.006567	0.001314	0.886064	2:98580012
2:98339513:C:T	0.3608	4.20E-06	0.006157	0.001337	0.811148	2:98580012
2:98342323:A:C	0.3926	3.83E-07	0.006673	0.001313	0.927428	2:98580012
2:98343258:A:G	0.493	8.91E-05	0.005048	0.001288	0.626927	2:98580012
2:98345086:G:T	0.4891	0.000109	0.005415	0.001399	0.620639	2:98580012
2:98346461:A:G	0.498	0.000895	-0.004907	0.001477	0.64041	2:98580012
2:98351654:T:TG	0.3608	NA	NA	NA	0.845975	2:98580012
2:98351986:C:T	0.498	8.75E-05	0.005052	0.001287	0.645262	2:98580012
2:98353847:C:T	0.3628	2.19E-06	0.006332	0.001336	0.845363	2:98580012
2:98354139:G:T	0.3618	1.93E-06	0.006353	0.001333	0.842339	2:98580012
2:98354511:A:G	0.3618	6.50E-08	0.007867	0.001454	0.849355	2:98580012
2:98355990:A:G	0.3628	1.38E-06	0.00644	0.001333	0.845363	2:98580012
2:98356846:A:G	0.4871	9.82E-06	0.005687	0.001285	0.610479	2:98580012
2:98357163:C:T	0.3748	7.20E-08	0.007207	0.001337	0.90016	2:98580012
2:98360443:A:G	0.4831	1.82E-05	0.005517	0.001286	0.601985	2:98580012
2:98361679:C:G	0.4851	1.58E-05	0.005552	0.001285	0.604987	2:98580012
2:98363313:A:T	0.4841	3.39E-05	0.00533	0.001285	0.604094	2:98580012
2:98365164:C:G	0.3926	8.77E-08	0.007025	0.001312	0.977964	2:98580012
2:98368551:A:ACT	0.4831	NA	NA	NA	0.60076	2:98580012
2:98370698:A:T	0.3907	7.60E-08	0.007116	0.001323	0.970489	2:98580012
2:98374567:C:T	0.3907	1.70E-07	0.006859	0.001311	0.985305	2:98580012
2:98377512:A:T	0.3926	1.56E-07	0.006878	0.00131	0.992648	2:98580012
2:98379267:C:T	0.3926	1.57E-07	0.006874	0.00131	0.992648	2:98580012
2:98379813:A:G	0.3926	1.56E-07	0.006875	0.00131	0.992648	2:98580012
2:98382886:A:G	0.3986	1.08E-07	0.006991	0.001315	0.956453	2:98580012
2:98384528:A:ATTTTC	0.3996	NA	NA	NA	0.967881	2:98580012
2:98386731:C:T	0.3917	1.82E-07	0.006839	0.00131	0.988961	2:98580012

uniqID	MAF	gwasP	beta	se	r2	IndSigSNP
2:98390117:G:GT	0.3579	NA	NA	NA	0.841361	2:98580012
2:98393231:C:T	0.3111	0.000698	0.004691	0.001383	0.697549	2:98580012
2:98393894:C:T	0.3917	1.84E-07	0.006851	0.001313	0.981726	2:98580012
2:98395653:A:C	0.3926	1.65E-07	0.006855	0.001309	0.992648	2:98580012
2:98397301:C:T	0.3917	1.58E-07	0.006866	0.001309	0.988961	2:98580012
2:98402753:A:T	0.3926	1.58E-07	0.006866	0.001309	0.992648	2:98580012
2:98402772:C:G	0.3907	1.59E-07	0.006887	0.001313	0.985305	2:98580012
2:98405034:C:T	0.3917	6.38E-07	0.006544	0.001313	0.988961	2:98580012
2:98405695:C:T	0.3917	1.37E-07	0.006902	0.001309	0.988961	2:98580012
2:98405929:A:G	0.3926	1.42E-07	0.006891	0.001309	0.992648	2:98580012
2:98407350:C:T	0.3926	1.19E-07	0.006934	0.001309	0.992648	2:98580012
2:98409046:A:G	0.3917	1.38E-07	0.00692	0.001313	0.988961	2:98580012
2:98409565:C:T	0.3936	1.12E-07	0.006948	0.001309	0.989048	2:98580012
2:98410769:G:T	0.3917	1.05E-07	0.006968	0.001309	0.988961	2:98580012
2:98413781:C:T	0.3926	1.47E-07	0.006899	0.001312	0.992648	2:98580012
2:98416848:A:C	0.3907	2.06E-07	0.006835	0.001315	0.985305	2:98580012
2:98416850:G:T	0.3907	2.06E-07	0.006834	0.001315	0.985305	2:98580012
2:98417089:C:T	0.3926	1.32E-07	0.006909	0.001309	0.992648	2:98580012
2:98419726:A:T	0.3926	1.40E-07	0.006895	0.001309	0.992648	2:98580012
2:98420142:C:G	0.3926	1.40E-07	0.006894	0.001309	0.992648	2:98580012
2:98420431:C:T	0.3926	1.39E-07	0.006896	0.001309	0.992648	2:98580012
2:98421364:A:G	0.3917	1.32E-07	0.00691	0.001309	0.988961	2:98580012
2:98424802:A:G	0.3926	1.34E-07	0.006906	0.001309	0.992648	2:98580012
2:98427382:C:T	0.3926	2.07E-08	0.008	0.001426	0.992648	2:98580012
2:98434931:G:GGGGGC	0.3897	NA	NA	NA	0.981591	2:98580012
2:98435982:C:T	0.3926	1.06E-07	0.006964	0.001309	0.992648	2:98580012
2:98440234:C:T	0.3926	8.69E-08	0.007015	0.00131	0.985292	2:98580012
2:98442202:A:G	0.3926	1.26E-07	0.00693	0.00131	0.985292	2:98580012
2:98443039:C:G	0.3926	5.71E-07	0.006573	0.001313	0.992648	2:98580012
2:98443081:G:GA	0.3917	NA	NA	NA	0.988961	2:98580012
2:98443658:C:T	0.3926	1.25E-07	0.006924	0.001309	0.992648	2:98580012
2:98447004:C:T	0.3917	1.18E-07	0.006939	0.001309	0.988961	2:98580012

uniqID	*MAF*	*gwasP*	*beta*	*se*	*r2*	*IndSigSNP*
2:98448395:G:T	0.3936	3.14E-07	0.006727	0.001314	0.98899	2:98580012
2:98449024:A:G	0.3917	1.18E-07	0.006939	0.001309	0.988961	2:98580012
2:98454472:A:G	0.3926	1.38E-07	0.006926	0.001314	0.985371	2:98580012
2:98454473:C:T	0.3926	1.30E-07	0.006941	0.001314	0.992648	2:98580012
2:98454572:C:G	0.3926	1.23E-07	0.006928	0.001309	0.992648	2:98580012
2:98454931:A:G	0.3917	5.52E-07	0.007512	0.001499	0.988961	2:98580012
2:98455152:C:T	0.3936	1.47E-07	0.006895	0.001311	0.98899	2:98580012
2:98455510:C:G	0.3926	1.22E-07	0.006929	0.001309	0.992648	2:98580012
2:98461821:C:T	0.3907	1.08E-07	0.006959	0.001309	1	2:98580012
2:98461953:C:T	0.3917	1.13E-07	0.006949	0.001309	0.996304	2:98580012
2:98466647:C:T	0.3907	1.28E-07	0.006918	0.001309	1	2:98580012
2:98468892:A:AC	0.3907	NA	NA	NA	1	2:98580012
2:98472610:C:T	0.3887	1.39E-07	0.00692	0.001313	0.992654	2:98580012
2:98479581:A:C	0.3887	1.25E-07	0.006942	0.001312	0.992625	2:98580012
2:98488949:C:G	0.3907	4.15E-07	0.006657	0.001314	1	2:98580012
2:98491750:C:T	0.3907	8.69E-08	0.007011	0.001309	1	2:98580012
2:98493359:A:G	0.3897	7.70E-08	0.007042	0.00131	0.996311	2:98580012
2:98495661:G:T	0.3907	8.68E-08	0.007012	0.001309	1	2:98580012
2:98501884:C:T	0.3897	1.95E-07	-0.007752	0.001489	0.996311	2:98580012
2:98502987:C:T	0.3907	8.89E-08	0.007007	0.001309	1	2:98580012
2:98506011:T:TATA	0.3887	NA	NA	NA	0.992654	2:98580012
2:98506910:C:T	0.3907	8.70E-08	0.007012	0.001309	1	2:98580012
2:98507972:C:CA	0.3907	NA	NA	NA	1	2:98580012
2:98510166:G:T	0.3907	9.47E-08	0.006992	0.001309	1	2:98580012
2:98517626:A:G	0.3897	1.12E-07	0.00697	0.001313	0.996311	2:98580012
2:98519014:C:T	0.3907	8.13E-08	0.007028	0.001309	1	2:98580012
2:98520564:C:T	0.3897	7.39E-08	0.007052	0.00131	0.996311	2:98580012
2:98521823:A:C	0.3907	8.75E-08	0.00701	0.001309	1	2:98580012
2:98526685:A:AAAAT	0.3897	NA	NA	NA	0.996311	2:98580012
2:98528689:A:G	0.3897	1.40E-07	0.006915	0.001312	0.996311	2:98580012
2:98534412:C:T	0.3956	1.80E-06	-0.007266	0.001521	0.981788	2:98580012
2:98534531:C:T	0.3897	1.14E-07	0.006956	0.001311	0.996311	2:98580012

uniqID	*MAF*	*gwasP*	*beta*	*se*	*r2*	*IndSigSNP*
2:98539588:A:C	0.3907	1.33E-07	0.006915	0.00131	1	2:98580012
2:98541001:C:T	0.3907	1.32E-07	0.006918	0.00131	1	2:98580012
2:98547069:A:AT	0.3907	NA	NA	NA	1	2:98580012
2:98548604:T:TA	0.4453	NA	NA	NA	0.715678	2:98580012
2:98551456:G:GA	0.3857	NA	NA	NA	0.966844	2:98580012
2:98552271:C:G	0.3887	1.40E-07	0.00693	0.001315	0.992654	2:98580012
2:98552299:C:G	0.3897	1.36E-07	0.006915	0.001311	0.996311	2:98580012
2:98554946:A:C	0.3897	2.11E-07	-0.007741	0.001491	0.996311	2:98580012
2:98557575:G:T	0.3897	1.85E-07	-0.007775	0.00149	0.996311	2:98580012
2:98561153:A:C	0.3907	1.71E-07	0.006872	0.001313	1	2:98580012
2:98565115:C:CT	0.3877	NA	NA	NA	0.988954	2:98580012
2:98565144:A:G	0.3907	1.38E-07	0.00691	0.001311	1	2:98580012
2:98565400:A:G	0.3907	1.20E-07	0.006943	0.001311	1	2:98580012
2:98567820:A:G	0.3907	1.09E-07	0.006969	0.001311	0.992646	2:98580012
2:98570999:A:G	0.3907	1.15E-07	0.006958	0.001311	1	2:98580012
2:98571084:A:G	0.3897	1,00E-07	0.006996	0.001313	0.996311	2:98580012
2:98580012:C:T	0.3907	1.57E-08	0.008074	0.001427	1	2:98580012
2:98580724:G:T	0.3907	1.19E-07	0.006953	0.001312	1	2:98580012
2:98587288:A:G	0.3887	1.38E-07	0.006955	0.001319	0.992654	2:98580012
2:98588372:A:C	0.3897	1.19E-07	0.006967	0.001315	0.996304	2:98580012
2:98602165:A:G	0.3897	3.77E-07	0.006718	0.001321	0.988902	2:98580012
2:98604570:G:GACAC	0.3887	NA	NA	NA	0.992625	2:98580012
2:98605982:A:G	0.3897	1.88E-07	-0.007884	0.001512	0.996304	2:98580012
2:98606770:C:T	0.3877	1.46E-07	0.00695	0.001321	0.988977	2:98580012
2:98612260:C:T	0.3887	1.21E-07	0.006988	0.001319	0.992625	2:98580012
2:98616519:A:ATT	0.4702	NA	NA	NA	0.728945	2:98580012
2:98621058:A:AG	0.3907	NA	NA	NA	1	2:98580012
2:98621060:A:T	0.3907	1.14E-07	-0.008091	0.001525	1	2:98580012
2:98623227:A:G	0.3887	9.79E-08	0.007081	0.001327	0.992625	2:98580012
2:98623406:A:G	0.3897	9.93E-08	0.007077	0.001327	0.988902	2:98580012
8:8524474:C:G	0.497	1.11E-06	-0.006404	0.001314	0.658999	8:8700851
8:8543324:C:T	0.496	1.37E-07	-0.006794	0.001288	0.666193	8:8700851

uniqID	***MAF***	***gwasP***	***beta***	***se***	***r2***	***IndSigSNP***
8:8544808:C:T	0.4891	5.18E-07	-0.006469	0.001288	0.650959	8:8700851
8:8544872:A:G	0.4891	5.31E-07	-0.006463	0.001288	0.650959	8:8700851
8:8545624:T:TTTG	0.4891	NA	NA	NA	0.644568	8:8700851
8:8546283:A:AAATT	0.4851	NA	NA	NA	0.647886	8:8700851
8:8547110:C:G	0.4871	7.04E-07	-0.006391	0.001288	0.644901	8:8700851
8:8547313:A:C	0.4811	1.89E-06	-0.006146	0.001289	0.647816	8:8700851
8:8547811:A:G	0.5	9.03E-08	-0.006885	0.001287	0.673959	8:8700851
8:8548117:A:T	0.4702	1.10E-07	-0.006841	0.001288	0.761793	8:8700851
8:8548801:A:G	0.495	5.01E-07	-0.006471	0.001287	0.65658	8:8700851
8:8549020:A:C	0.5	1.47E-07	-0.006773	0.001287	0.673959	8:8700851
8:8549432:A:G	0.495	6.47E-07	-0.00641	0.001287	0.663079	8:8700851
8:8578067:C:T	0.4394	2.04E-07	-0.006715	0.001291	0.718619	8:8700851
8:8578120:A:T	0.4453	1.20E-07	-0.006842	0.001292	0.704506	8:8700851
8:8578229:A:G	0.4414	2.02E-07	-0.006717	0.001291	0.718733	8:8700851
8:8578794:A:T	0.4384	9.96E-08	-0.006875	0.00129	0.721473	8:8700851
8:8578811:C:T	0.4394	1.04E-07	-0.006864	0.00129	0.718619	8:8700851
8:8581408:C:G	0.4453	3.67E-07	-0.006555	0.001288	0.712333	8:8700851
8:8582155:A:AAAAAGAAAAG	0.4463	NA	NA	NA	0.708543	8:8700851
8:8582155:AAAAAG:AAAAAGAAAAG	0.03579	NA	NA	NA	0.708543	8:8700851
8:8583872:C:T	0.4453	6.86E-07	-0.006392	0.001286	0.712333	8:8700851
8:8584344:A:G	0.4453	5.59E-07	-0.006442	0.001286	0.712333	8:8700851
8:8587571:G:T	0.4761	6.23E-07	-0.006395	0.001282	0.611144	8:8700851
8:8589117:C:T	0.4404	4.54E-07	-0.007033	0.001393	0.714749	8:8700851
8:8592845:C:G	0.4404	8.02E-08	-0.006916	0.001288	0.70905	8:8700851
8:8595104:A:T	0.4414	1.43E-07	-0.00678	0.001288	0.718733	8:8700851
8:8595838:G:T	0.4771	6.96E-07	-0.006376	0.001284	0.615422	8:8700851
8:8596731:G:T	0.4771	6.88E-07	-0.006382	0.001285	0.615422	8:8700851
8:8598388:A:T	0.4463	1.63E-07	-0.006755	0.001289	0.742669	8:8700851
8:8602344:C:T	0.4801	2.36E-06	-0.006579	0.001393	0.652447	8:8700851
8:8603160:A:G	0.4801	2.34E-06	-0.006093	0.00129	0.652447	8:8700851
8:8633548:G:T	0.4553	1.51E-08	-0.007318	0.001292	0.862496	8:8700851
8:8637429:A:G	0.4543	2.15E-08	-0.007226	0.00129	0.858312	8:8700851

uniqID	*MAF*	*gwasP*	*beta*	*se*	*r2*	*IndSigSNP*
8:8639740:A:G	0.4553	2.85E-08	-0.007166	0.00129	0.863089	8:8700851
8:8640172:A:C	0.4414	1.12E-07	-0.006871	0.001294	0.824271	8:8700851
8:8641145:C:T	0.4553	2.01E-08	0.007248	0.001291	0.869369	8:8700851
8:8643938:C:T	0.4483	5.01E-08	0.007051	0.001293	0.879353	8:8700851
8:8644213:G:GT	0.4056	NA	NA	NA	0.7681	8:8700851
8:8644213:G:GTT	0.2932	NA	NA	NA	0.7681	8:8700851
8:8644274:C:G	0.4483	4.53E-08	0.007074	0.001292	0.879353	8:8700851
8:8644595:A:AT	0.4761	NA	NA	NA	0.71305	8:8700851
8:8646246:C:T	0.4483	9.37E-09	0.007416	0.001291	0.894914	8:8700851
8:8649881:C:T	0.4414	5.67E-08	0.007025	0.001293	0.86851	8:8700851
8:8652889:A:G	0.4463	1.67E-08	0.007269	0.001287	0.894348	8:8700851
8:8654057:A:G	0.4463	NA	NA	NA	0.894348	8:8700851
8:8654527:C:T	0.4304	4.91E-08	0.007035	0.001289	0.835563	8:8700851
8:8658540:A:G	0.4324	7.71E-08	0.00693	0.001289	0.835317	8:8700851
8:8660538:A:C	0.4245	3.14E-08	0.007136	0.001289	0.815212	8:8700851
8:8661026:C:CA	0.4533	NA	NA	NA	0.85866	8:8700851
8:8661114:C:CA	0.4463	NA	NA	NA	0.893959	8:8700851
8:8661534:C:T	0.4473	1.65E-08	0.007262	0.001286	0.898029	8:8700851
8:8661681:C:G	0.4304	1.92E-08	0.007243	0.001288	0.843543	8:8700851
8:8663215:C:T	0.4583	8.66E-05	0.005086	0.001295	0.643232	8:8700851
8:8664622:A:G	0.4245	1.61E-08	0.007302	0.001292	0.814548	8:8700851
8:8664940:A:G	0.4573	4.18E-05	0.00529	0.00129	0.64675	8:8700851
8:8665147:A:G	0.4573	2.24E-05	0.005924	0.001396	0.64675	8:8700851
8:8665802:C:T	0.4443	NA	NA	NA	0.869838	8:8700851
8:8666916:C:T	0.4543	5.56E-05	0.00521	0.001292	0.646281	8:8700851
8:8667444:C:T	0.4513	2.72E-05	0.005432	0.001294	0.653566	8:8700851
8:8668486:A:G	0.4513	4.07E-05	0.005308	0.001293	0.654927	8:8700851
8:8668917:A:C	0.4513	3.71E-05	0.005335	0.001293	0.654927	8:8700851
8:8669681:C:CGTAA	0.4304	NA	NA	NA	0.812894	8:8700851
8:8669681:C:T	0.000994	NA	NA	NA	0.812894	8:8700851
8:8670082:C:G	0.4294	6.67E-08	0.006963	0.001289	0.817012	8:8700851
8:8670177:A:T	0.4294	8.34E-08	0.006915	0.001289	0.817012	8:8700851

uniqID	*MAF*	*gwasP*	*beta*	*se*	*r2*	*IndSigSNP*
8:8670322:C:CT	0.4523	NA	NA	NA	0.65623	8:8700851
8:8670599:A:G	0.4294	9.99E-08	0.006877	0.00129	0.817012	8:8700851
8:8670736:A:C	0.4264	1.10E-07	0.006858	0.001291	0.806197	8:8700851
8:8671962:C:T	0.4284	6.86E-08	0.006958	0.001289	0.821145	8:8700851
8:8672429:C:G	0.4304	6.63E-08	0.00697	0.00129	0.81356	8:8700851
8:8672579:A:G	0.4294	8.14E-08	0.006921	0.001289	0.817012	8:8700851
8:8672952:A:C	0.4553	2.99E-05	0.005399	0.001293	0.646246	8:8700851
8:8673320:C:T	0.4553	3.08E-05	0.005389	0.001292	0.646246	8:8700851
8:8673601:A:C	0.4433	2.76E-08	0.007168	0.001289	0.875531	8:8700851
8:8673736:C:T	0.4404	3.14E-08	0.007136	0.001289	0.864008	8:8700851
8:8675176:A:G	0.4543	3.86E-05	0.005323	0.001292	0.644912	8:8700851
8:8675325:A:T	0.4463	3,00E-08	0.007152	0.00129	0.886756	8:8700851
8:8676626:G:GAATC	0.4394	NA	NA	NA	0.867548	8:8700851
8:8678530:A:G	0.4384	2.37E-08	0.007207	0.00129	0.879145	8:8700851
8:8679141:C:CTT	0.4404	NA	NA	NA	0.88665	8:8700851
8:8679176:A:G	0.4404	3.52E-08	0.007118	0.00129	0.88665	8:8700851
8:8679614:C:T	0.4414	2.92E-08	0.00716	0.00129	0.882627	8:8700851
8:8680477:A:G	0.4414	3.66E-08	0.007109	0.00129	0.882627	8:8700851
8:8682192:A:T	0.4374	2.91E-08	0.007164	0.001291	0.8752	8:8700851
8:8682878:C:T	0.4493	3.68E-08	0.007108	0.00129	0.905506	8:8700851
8:8684953:A:G	0.4443	3.09E-08	0.007165	0.001293	0.87023	8:8700851
8:8685190:A:G	0.4453	4.81E-08	0.00706	0.001292	0.874272	8:8700851
8:8685646:C:T	0.4533	2.69E-08	0.007186	0.001291	0.89766	8:8700851
8:8685854:A:G	0.4543	1.92E-08	0.007265	0.001292	0.89381	8:8700851
8:8687054:C:G	0.4175	7.49E-07	0.006463	0.001305	0.804384	8:8700851
8:8687325:A:G	0.4732	3.52E-07	-0.006599	0.001295	0.682135	8:8700851
8:8690787:C:T	0.4225	5,00E-07	0.006535	0.001299	0.837119	8:8700851
8:8691622:A:T	0.4175	3.98E-07	0.006598	0.0013	0.82771	8:8700851
8:8692477:C:T	0.4205	1.08E-07	0.006913	0.0013	0.838216	8:8700851
8:8696449:G:T	0.4225	4.86E-07	0.006543	0.001299	0.837692	8:8700851
8:8698977:C:CA	0.3926	NA	NA	NA	0.691379	8:8700851
8:8699757:A:T	0.4195	3.83E-07	0.006605	0.0013	0.864894	8:8700851

uniqID	*MAF*	*gwasP*	*beta*	*se*	*r2*	*IndSigSNP*
8:8699761:C:T	0.4195	4.06E-07	0.00659	0.0013	0.864894	8:8700851
8:8700851:C:T	0.4453	8.41E-09	0.007429	0.001289	1	8:8700851
8:8702607:C:G	0.4274	2.30E-06	0.006129	0.001296	0.7906	8:8700851
8:8702827:A:G	0.4165	8.60E-08	0.006942	0.001296	0.819293	8:8700851
8:8702875:C:T	0.4165	5.84E-08	0.007028	0.001295	0.819293	8:8700851
8:8703781:G:GGA	0.4056	NA	NA	NA	0.797657	8:8700851
8:8704330:C:G	0.4394	2.87E-08	0.007156	0.001289	0.96021	8:8700851
8:8706130:A:AT	0.3688	NA	NA	NA	0.679623	8:8700851
8:8706209:A:C	0.3728	3.22E-05	0.00552	0.001327	0.689251	8:8700851
8:8706332:A:C	0.4145	1.04E-07	0.006928	0.001302	0.824166	8:8700851
8:8707197:C:G	0.3867	1.86E-06	0.006245	0.001309	0.760986	8:8700851
8:8708974:C:G	0.3549	0.000256	0.004875	0.001333	0.638323	8:8700851
8:8709756:C:G	0.3569	0.00042	0.004733	0.001341	0.635456	8:8700851
8:8709971:C:T	0.3569	0.000227	0.004943	0.00134	0.634339	8:8700851
8:8713038:C:T	0.3569	4.06E-07	0.006726	0.001327	0.672386	8:8700851
8:8718850:A:G	0.3489	5.27E-06	0.006073	0.001333	0.672234	8:8700851
8:8719513:A:G	0.3698	4.65E-06	0.006028	0.001315	0.722472	8:8700851
8:8721473:A:G	0.4095	2.18E-08	0.007253	0.001295	0.853201	8:8700851
8:8722675:C:T	0.3946	3.44E-06	0.006534	0.001406	0.776181	8:8700851
8:8723651:C:G	0.3976	9.29E-07	0.006376	0.001299	0.771217	8:8700851
8:8724257:C:T	0.3787	1.85E-06	0.006232	0.001306	0.730922	8:8700851
8:8724276:C:T	0.3777	1.52E-06	0.00629	0.001307	0.728537	8:8700851
8:8724415:C:T	0.3509	1.97E-06	0.006297	0.001323	0.662353	8:8700851
8:8725126:G:T	0.3787	3.34E-06	0.00609	0.001309	0.630584	8:8700851
8:8725229:A:G	0.3797	3.70E-06	0.006064	0.001309	0.632684	8:8700851
8:8725319:A:G	0.3857	2.62E-05	0.00549	0.001305	0.624255	8:8700851
8:8726804:G:T	0.3986	1.64E-06	0.006243	0.001301	0.658076	8:8700851
8:8729761:C:G	0.4235	5.09E-08	0.007064	0.001296	0.839283	8:8700851
8:8730488:A:G	0.4175	3.24E-08	0.007178	0.001297	0.849079	8:8700851
8:8768895:A:G	0.3976	2.01E-07	0.006778	0.001303	0.975257	8:8770512
8:8769708:C:T	0.3996	8.08E-08	0.007008	0.001305	0.97504	8:8770512
8:8770512:C:T	0.4036	2.27E-08	0.007282	0.001302	1	8:8770512

uniqID	*MAF*	*gwasP*	*beta*	*se*	*r2*	*IndSigSNP*
8:8824858:G:T	0.4602	3.11E-05	0.005376	0.00129	0.60318	8:8700851
8:9394053:G:T	0.161	7.78E-06	-0.007565	0.001691	0.672355	8:9569104
8:9489417:A:G	0.165	3.82E-07	0.008442	0.001661	0.872055	8:9569104
8:9492426:G:T	0.164	6.36E-07	0.008278	0.001661	0.853467	8:9569104
8:9492453:C:T	0.164	6.04E-07	0.008294	0.001661	0.853467	8:9569104
8:9494732:G:T	0.171	1.28E-06	-0.007973	0.001645	0.848521	8:9569104
8:9496118:A:T	0.173	5.45E-06	-0.007439	0.001635	0.836838	8:9569104
8:9525325:C:T	0.16	6.26E-07	0.008354	0.001675	0.904352	8:9569104
8:9527707:A:G	0.166	1.18E-06	0.008085	0.001663	0.904136	8:9569104
8:9527863:A:T	0.16	8.67E-07	0.008237	0.001673	0.904352	8:9569104
8:9527869:A:G	0.16	8.91E-07	0.008229	0.001673	0.904352	8:9569104
8:9568369:A:G	0.1849	3.11E-06	0.00738	0.001581	0.8689	8:9569104
8:9569104:C:T	0.169	3.84E-08	0.009116	0.001657	1	8:9569104
8:9569109:C:T	0.1083	8.97E-06	0.00886	0.001994	0.614656	8:9569104
8:9572099:A:G	0.1044	9,00E-06	0.008905	0.002004	0.6079	8:9569104
8:9574830:G:T	0.1044	8.21E-06	0.008945	0.002004	0.6079	8:9569104
8:9575445:A:G	0.1044	8.65E-06	0.008917	0.002003	0.6079	8:9569104
8:9579144:C:CT	0.1034	NA	NA	NA	0.603121	8:9569104
8:9579318:C:T	0.1034	0.000199	-0.008522	0.00229	0.603121	8:9569104
8:9579377:C:CA	0.1044	NA	NA	NA	0.6079	8:9569104
8:9583872:C:T	0.1044	9.71E-06	0.008893	0.002009	0.6079	8:9569104
8:9584598:C:G	0.1054	8.61E-06	0.008939	0.002008	0.612705	8:9569104
8:9586062:A:T	0.1054	1.06E-05	0.008869	0.002012	0.612705	8:9569104
8:9593309:A:C	0.1054	1.06E-05	0.008907	0.002021	0.612705	8:9569104
8:10121635:A:G	0.4414	6.97E-08	0.007013	0.0013	0.608898	8:10147398
8:10122423:A:G	0.4423	1.24E-07	0.006867	0.001298	0.611745	8:10147398
8:10146490:C:CT	0.4304	NA	NA	NA	0.966987	8:10147398
8:10147398:C:T	0.4344	1.47E-09	-0.007824	0.001293	1	8:10147398
8:10148447:A:G	0.3171	6.47E-07	0.006846	0.001375	0.616603	8:10147398
8:10149212:A:C	0.3191	1.07E-06	0.006713	0.001375	0.621124	8:10147398
8:10150070:C:G	0.3191	8.40E-07	0.006772	0.001374	0.621124	8:10147398
8:10176506:A:T	0.3738	2.39E-07	0.006827	0.001321	0.719169	8:10147398

uniqID	*MAF*	*gwasP*	*beta*	*se*	*r2*	*IndSigSNP*
8:10758213:A:G	0.4692	7.16E-05	-0.005127	0.00129	0.602947	8:11080675
8:10810451:A:G	0.4712	1.14E-05	-0.005678	0.001293	0.689151	8:11080675
8:10811829:C:T	0.4712	2.06E-05	-0.005503	0.001291	0.700569	8:11080675
8:10812333:G:GT	0.4911	NA	NA	NA	0.632362	8:11080675
8:10813197:C:T	0.4801	1.28E-05	-0.005638	0.001291	0.682847	8:11080675
8:10813904:G:T	0.4831	1.08E-05	-0.00568	0.00129	0.666365	8:11080675
8:10815754:C:G	0.4821	1.08E-05	-0.005678	0.00129	0.678995	8:11080675
8:10816772:A:G	0.4771	8.29E-06	-0.005751	0.001289	0.683304	8:11080675
8:10817197:C:T	0.4791	1.05E-05	-0.005685	0.001289	0.683056	8:11080675
8:10818607:A:G	0.4851	1.36E-05	-0.005608	0.001288	0.684993	8:11080675
8:10818657:A:G	0.4771	1.34E-05	-0.005612	0.001288	0.684509	8:11080675
8:10819854:C:T	0.4831	8.02E-06	-0.005751	0.001287	0.691423	8:11080675
8:10821056:C:CT	0.4751	NA	NA	NA	0.628464	8:11080675
8:10828909:C:T	0.4702	8.81E-06	-0.005752	0.001293	0.704543	8:11080675
8:10831868:G:T	0.4732	1.12E-05	-0.005688	0.001294	0.688789	8:11080675
8:10835480:C:T	0.4871	2.82E-05	-0.005433	0.001297	0.662377	8:11080675
8:10835917:C:T	0.4861	1.80E-05	-0.005559	0.001295	0.666165	8:11080675
8:10836024:C:T	0.4861	1.78E-05	-0.005567	0.001296	0.666165	8:11080675
8:10836069:C:T	0.4861	1.95E-05	-0.005535	0.001295	0.666165	8:11080675
8:10836359:C:T	0.495	3.17E-05	-0.005395	0.001296	0.666257	8:11080675
8:10836436:C:T	0.4861	2.28E-05	-0.005492	0.001296	0.666165	8:11080675
8:10836463:C:G	0.4861	2.49E-05	-0.005467	0.001296	0.666165	8:11080675
8:10836508:A:G	0.496	2.14E-05	-0.005507	0.001295	0.66878	8:11080675
8:10837019:A:G	0.4871	1.48E-05	-0.005618	0.001296	0.668647	8:11080675
8:10837190:A:G	0.495	3.92E-05	-0.005329	0.001295	0.666257	8:11080675
8:10837414:A:C	0.4861	1.74E-05	-0.00557	0.001296	0.688707	8:11080675
8:10837420:A:G	0.4851	2.71E-05	-0.005442	0.001296	0.66371	8:11080675
8:10837568:C:T	0.4801	1.42E-05	-0.005633	0.001297	0.680392	8:11080675
8:10837569:C:G	0.4801	1.53E-05	-0.00561	0.001297	0.680392	8:11080675
8:10839803:C:T	0.4891	5.48E-06	-0.005895	0.001296	0.663593	8:11080675
8:10841858:A:G	0.495	4.49E-06	-0.005928	0.001291	0.671212	8:11080675
8:10842659:G:T	0.4781	4.54E-06	-0.005937	0.001294	0.680617	8:11080675

uniqID	*MAF*	*gwasP*	*beta*	*se*	*r2*	*IndSigSNP*
8:10903475:A:T	0.4881	8.09E-09	-0.007448	0.001291	0.705225	8:11080675
8:10909193:A:C	0.4374	7.91E-08	-0.006971	0.001297	0.658836	8:11080675
8:10909936:C:T	0.4364	1,00E-07	0.006917	0.001298	0.655615	8:11080675
8:10910066:C:T	0.4364	9.88E-08	0.006924	0.001298	0.655615	8:11080675
8:10910343:C:G	0.4344	2.59E-07	-0.006751	0.00131	0.649205	8:11080675
8:10926892:A:C	0.498	1.13E-07	-0.00684	0.001288	0.711057	8:11080675
8:10927234:C:CAGTA	0.498	NA	NA	NA	0.711057	8:11080675
8:10930069:A:C	0.4771	1.45E-07	-0.006794	0.001291	0.750978	8:11080675
8:10932203:C:G	0.492	8.82E-08	-0.006897	0.001288	0.724052	8:11080675
8:10932695:C:G	0.4801	1.83E-07	-0.006733	0.00129	0.739053	8:11080675
8:10932868:A:G	0.4781	3.88E-07	-0.006548	0.001289	0.74607	8:11080675
8:10933699:A:T	0.4821	2.29E-07	-0.006674	0.001289	0.73883	8:11080675
8:10935082:C:T	0.4712	1.56E-07	-0.006768	0.001289	0.770099	8:11080675
8:10935366:C:T	0.4712	2.81E-07	-0.006652	0.001294	0.770099	8:11080675
8:10935368:A:C	0.4712	1.28E-07	-0.006821	0.001291	0.770099	8:11080675
8:10935898:A:G	0.4712	1.93E-07	-0.006718	0.001289	0.770099	8:11080675
8:10936811:A:G	0.4722	1.25E-07	-0.006817	0.001289	0.766812	8:11080675
8:10936891:C:T	0.4722	1.30E-07	-0.006808	0.001289	0.766812	8:11080675
8:10938260:A:G	0.4831	2.63E-08	-0.007176	0.001289	0.755201	8:11080675
8:10939273:G:T	0.4891	7.22E-07	-0.00639	0.001289	0.673246	8:11080675
8:10939490:G:T	0.4901	5.13E-07	-0.006475	0.001289	0.669227	8:11080675
8:10943276:C:T	0.4722	4.38E-05	-0.005265	0.001287	0.673798	8:11080675
8:10944809:G:T	0.499	7.55E-06	-0.00577	0.001288	0.751444	8:11080675
8:10945439:A:G	0.493	1.53E-05	-0.005573	0.001288	0.736523	8:11080675
8:10945767:C:CA	0.4871	NA	NA	NA	0.757637	8:11080675
8:10948422:C:CA	0.499	NA	NA	NA	0.735175	8:11080675
8:10948968:A:G	0.4861	NA	NA	NA	0.635195	8:11080675
8:10950396:C:G	0.499	6,00E-06	-0.005833	0.001288	0.734961	8:11080675
8:10950757:C:G	0.499	5.79E-06	-0.005844	0.001288	0.734961	8:11080675
8:10950866:C:T	0.499	5.96E-06	-0.005836	0.001288	0.734235	8:11080675
8:10951175:G:GT	0.496	NA	NA	NA	0.732707	8:11080675
8:10952500:A:T	0.4771	2.08E-05	-0.005494	0.00129	0.673469	8:11080675

uniqID	*MAF*	*gwasP*	*beta*	*se*	*r2*	*IndSigSNP*
8:10953092:C:T	0.499	4.04E-06	-0.005946	0.001289	0.73613	8:11080675
8:10953874:A:G	0.493	3.89E-06	-0.005956	0.001289	0.743754	8:11080675
8:10955225:C:G	0.4901	6.96E-06	-0.005799	0.001289	0.750096	8:11080675
8:10955383:G:GTT	0.03479	NA	NA	NA	0.651528	8:11080675
8:10955383:GT:GTT	0.4592	NA	NA	NA	0.651528	8:11080675
8:10957243:A:G	0.4891	6.39E-06	-0.005824	0.00129	0.78233	8:11080675
8:10958824:C:T	0.4881	7.38E-06	-0.005781	0.001289	0.785444	8:11080675
8:10960572:C:T	0.4841	1.68E-07	-0.006739	0.001287	0.77954	8:11080675
8:10961433:C:T	0.4662	1.49E-05	-0.005593	0.001291	0.674611	8:11080675
8:10962099:A:T	0.4702	1.35E-05	-0.005623	0.001291	0.685626	8:11080675
8:10962800:C:T	0.4831	1.82E-07	-0.006723	0.001288	0.76289	8:11080675
8:10962929:C:G	0.4801	1.74E-07	-0.006734	0.001288	0.779975	8:11080675
8:10963288:C:G	0.4831	1.45E-07	-0.006776	0.001288	0.77659	8:11080675
8:10964906:C:T	0.4483	2.72E-06	-0.006043	0.001287	0.600213	8:11080675
8:10964969:A:T	0.4483	2.07E-06	-0.006114	0.001287	0.600213	8:11080675
8:10968550:C:G	0.4463	2.95E-05	-0.006163	0.001474	0.616425	8:11080675
8:10968926:A:G	0.4443	1.79E-05	-0.005543	0.001291	0.611485	8:11080675
8:10969075:C:T	0.4463	3.89E-06	-0.005952	0.001288	0.608933	8:11080675
8:10970773:A:T	0.4761	3.59E-07	-0.006554	0.001287	0.781408	8:11080675
8:10973149:C:CAA	0.003976	NA	NA	NA	0.777747	8:11080675
8:10973149:C:CA	0.4662	NA	NA	NA	0.777747	8:11080675
8:10974917:C:T	0.4543	1.85E-06	-0.006135	0.001285	0.603421	8:11080675
8:10975081:A:T	0.4553	7.41E-07	-0.006367	0.001285	0.605263	8:11080675
8:10975629:A:C	0.4553	1.39E-06	-0.006215	0.001287	0.606612	8:11080675
8:10975682:C:G	0.4722	1.16E-07	-0.006814	0.001285	0.797653	8:11080675
8:10975733:A:G	0.4563	3.73E-06	-0.005973	0.00129	0.622026	8:11080675
8:10976494:C:G	0.4732	5.06E-07	0.007356	0.001463	0.8005	8:11080675
8:10976569:A:G	0.492	1.89E-07	0.007776	0.001492	0.748587	8:11080675
8:10976571:G:T	0.492	NA	NA	NA	0.748587	8:11080675
8:10978065:G:T	0.4742	NA	NA	NA	0.795656	8:11080675
8:10979561:C:G	0.4632	1.55E-07	-0.006755	0.001287	0.781738	8:11080675
8:10979821:A:G	0.4622	2.73E-07	-0.007193	0.001398	0.785148	8:11080675

uniqID	*MAF*	*gwasP*	*beta*	*se*	*r2*	*IndSigSNP*
8:10981003:A:C	0.4722	2.23E-05	-0.005488	0.001294	0.707395	8:11080675
8:10982051:A:G	0.4672	8.22E-08	-0.006908	0.001287	0.800562	8:11080675
8:10982410:C:G	0.3917	1.47E-05	-0.005725	0.00132	0.742891	8:10988275
8:10983534:A:G	0.4563	1.49E-07	-0.006766	0.001287	0.795996	8:11080675
8:10983579:G:T	0.4324	NA	NA	NA	0.689915	8:11080675
8:10983921:C:G	0.3847	3.96E-06	-0.006093	0.00132	0.715547	8:10988275
8:10985140:A:G	0.3767	7.79E-06	-0.00592	0.001323	0.810136	8:10988275
8:10985432:C:T	0.4771	6.77E-08	-0.006943	0.001285	0.783503	8:11080675
8:10986837:G:T	0.4274	4.41E-08	-0.007109	0.001298	0.995983	8:10988275
8:10986859:C:T	0.4771	1.01E-07	-0.006851	0.001286	0.783503	8:11080675
8:10987199:C:T	0.4274	3.08E-08	-0.007183	0.001296	0.995983	8:10988275
8:10987553:C:T	0.4264	2.20E-08	-0.007267	0.001298	1	8:10988275
8:10987651:G:T	0.4264	2.65E-08	-0.007225	0.001298	1	8:10988275
8:10987967:A:G	0.3708	1.78E-06	-0.00635	0.001328	0.784459	8:10988275
8:10988138:A:C	0.3748	1.48E-05	-0.005764	0.00133	0.804653	8:10988275
8:10988275:A:G	0.4264	5.79E-09	-0.008199	0.001407	1	8:10988275
8:10989057:A:G	0.3688	1.82E-06	-0.006335	0.001326	0.778454	8:10988275
8:10989206:C:T	0.3708	2.15E-06	-0.006289	0.001326	0.784459	8:10988275
8:10989521:A:G	0.3688	1.96E-06	-0.006323	0.001328	0.779216	8:10988275
8:10990164:A:G	0.3688	2.55E-06	-0.006243	0.001326	0.779216	8:10988275
8:10990371:G:GA	0.4463	NA	NA	NA	0.653135	8:11080675
8:10990672:C:T	0.3678	3.51E-06	-0.006156	0.001326	0.769437	8:10988275
8:10992252:C:G	0.3509	8.02E-06	-0.006052	0.001355	0.714415	8:10988275
8:10992544:A:AT	0.4781	NA	NA	NA	0.665209	8:11080675
8:10992605:A:C	0.3678	2.74E-06	-0.006224	0.001326	0.761448	8:10988275
8:10992883:C:T	0.3688	2.87E-06	-0.00621	0.001326	0.764825	8:10988275
8:10993904:C:T	0.3877	5.72E-06	-0.005979	0.001317	0.690481	8:10988275
8:10993995:C:T	0.3688	NA	NA	NA	0.764825	8:10988275
8:10994743:C:G	0.3688	3.73E-06	-0.006137	0.001326	0.764825	8:10988275
8:10996089:A:G	0.3787	2.23E-06	-0.006274	0.001325	0.723591	8:10988275
8:11010974:G:T	0.4463	2.68E-05	-0.005445	0.001296	0.607867	8:11080675
8:11012977:C:T	0.4801	8.51E-07	-0.006351	0.001289	0.858958	8:11080675

uniqID	*MAF*	*gwasP*	*beta*	*se*	*r2*	*IndSigSNP*
8:11013025:A:C	0.3628	2.80E-06	-0.006212	0.001325	0.719967	8:10988275
8:11014616:G:T	0.4911	1.52E-06	-0.006219	0.001292	0.852586	8:11080675
8:11015338:C:T	0.4781	8.80E-07	-0.006338	0.001288	0.859283	8:11080675
8:11016889:A:G	0.3738	3.90E-06	-0.00612	0.001325	0.751798	8:10988275
8:11019578:C:G	0.4821	2.35E-06	-0.006092	0.00129	0.828846	8:11080675
8:11020313:G:T	0.3658	1.59E-06	-0.006382	0.001329	0.706287	8:10988275
8:11021682:A:G	0.3777	1.33E-05	-0.005787	0.001328	0.733031	8:10988275
8:11022106:C:G	0.3777	4.40E-06	-0.00608	0.001323	0.733031	8:10988275
8:11022185:C:T	0.3777	4.61E-06	-0.006067	0.001323	0.733031	8:10988275
8:11023655:C:T	0.3777	4.26E-06	-0.006093	0.001324	0.740066	8:10988275
8:11023997:C:G	0.4751	1.25E-06	-0.006779	0.001397	0.826753	8:11080675
8:11024275:C:T	0.3757	3.69E-06	-0.006136	0.001325	0.748991	8:10988275
8:11024326:A:C	0.4742	3.76E-06	-0.005976	0.001291	0.830216	8:11080675
8:11024663:A:C	0.5	1.72E-06	-0.00617	0.001289	0.856468	8:11080675
8:11027491:T:TAA	0.4901	NA	NA	NA	0.705092	8:11080675
8:11029029:G:T	0.492	8.99E-06	-0.005745	0.001293	0.712278	8:11080675
8:11029039:G:T	0.4602	1.06E-06	-0.006332	0.001297	0.806852	8:11080675
8:11030892:A:G	0.3728	2.56E-06	-0.006261	0.00133	0.749253	8:10988275
8:11030935:C:T	0.4622	4.33E-06	-0.005962	0.001297	0.704456	8:11080675
8:11031472:C:T	0.4612	2.48E-07	-0.006714	0.001301	0.76702	8:11080675
8:11032228:G:T	0.4751	3.19E-07	-0.006618	0.001294	0.818364	8:11080675
8:11032240:C:T	0.4751	5.02E-07	-0.006518	0.001296	0.818364	8:11080675
8:11033517:C:G	0.4871	1,00E-07	-0.006875	0.00129	0.85324	8:11080675
8:11033525:C:T	0.4871	1.04E-07	-0.006866	0.00129	0.85324	8:11080675
8:11033737:A:G	0.3499	5.62E-06	-0.006072	0.001337	0.631583	8:10988275
8:11033976:C:T	0.4851	6.52E-08	-0.006967	0.001288	0.860141	8:11080675
8:11034028:A:G	0.4523	1.06E-05	-0.00569	0.001291	0.69918	8:11080675
8:11034859:A:G	0.3499	7.07E-06	-0.005992	0.001333	0.631583	8:10988275
8:11035071:G:GAGGTCATAATGGAAT	0.3509	NA	NA	NA	0.633632	8:10988275
8:11036052:T:TAA	0.3976	NA	NA	NA	0.668109	8:10988275
8:11036799:C:G	0.3489	8.17E-06	-0.005946	0.001332	0.621748	8:10988275
8:11036843:C:T	0.3489	8.84E-06	-0.005923	0.001332	0.621748	8:10988275

uniqID	*MAF*	*gwasP*	*beta*	*se*	*r2*	*IndSigSNP*
8:11036919:A:G	0.3489	9.57E-06	-0.0059	0.001332	0.621748	8:10988275
8:11037034:A:G	0.4543	7.22E-06	-0.005788	0.001289	0.696883	8:11080675
8:11037187:C:T	0.4861	5.20E-08	-0.007005	0.001286	0.85617	8:11080675
8:11037903:G:T	0.3489	1.40E-05	-0.005779	0.00133	0.621748	8:10988275
8:11038244:A:T	0.4404	6.62E-06	-0.005826	0.001292	0.711278	8:11080675
8:11038885:A:T	0.3499	9.50E-06	-0.005901	0.001332	0.618479	8:10988275
8:11039159:C:G	0.3489	1.17E-05	-0.00584	0.001332	0.621748	8:10988275
8:11039816:G:T	0.4851	4.94E-08	-0.007028	0.001288	0.852859	8:11080675
8:11040216:C:T	0.4105	1.16E-07	-0.006892	0.0013	0.785271	8:10988275
8:11040647:A:G	0.4821	1.88E-07	-0.006713	0.001287	0.843154	8:11080675
8:11041642:A:T	0.4871	4.46E-08	-0.007042	0.001286	0.859994	8:11080675
8:11041661:C:T	0.3499	8.09E-06	-0.005951	0.001333	0.623772	8:10988275
8:11041897:T:TACACACACACACAC	0.3519	NA	NA	NA	0.615997	8:10988275
8:11042974:C:G	0.1014	NA	NA	NA	0.777089	8:10988275
8:11042974:G:T	0.4125	NA	NA	NA	0.777089	8:10988275
8:11043138:C:T	0.3489	9.03E-06	-0.005921	0.001333	0.621748	8:10988275
8:11043236:C:T	0.4394	6.85E-06	-0.005819	0.001293	0.710174	8:11080675
8:11043926:C:T	0.4394	8.55E-06	-0.005758	0.001293	0.710174	8:11080675
8:11044689:A:G	0.3489	8.46E-06	-0.005939	0.001333	0.621748	8:10988275
8:11045161:A:G	0.4881	4.08E-08	-0.007055	0.001285	0.849852	8:11080675
8:11046209:C:T	0.4066	4.37E-08	-0.007142	0.001303	0.794521	8:10988275
8:11046394:C:G	0.4076	7.69E-09	-0.008162	0.001412	0.789643	8:10988275
8:11053922:A:G	0.4841	1.36E-07	-0.006812	0.001292	0.851814	8:11080675
8:11054097:A:C	0.4831	4.17E-08	-0.007068	0.001288	0.848726	8:11080675
8:11055597:A:C	0.4871	2.67E-08	-0.007165	0.001287	0.869899	8:11080675
8:11056175:A:G	0.3449	2.15E-05	-0.005675	0.001335	0.603496	8:10988275
8:11056388:C:T	0.3439	1.59E-05	-0.005767	0.001336	0.601547	8:10988275
8:11060217:T:TA	0.5	NA	NA	NA	0.858045	8:11080675
8:11060311:A:C	0.498	9.05E-09	-0.007402	0.001287	0.865612	8:11080675
8:11061792:C:T	0.497	8.98E-09	-0.007402	0.001287	0.869415	8:11080675
8:11062882:C:T	0.496	5.19E-09	-0.007525	0.001287	0.873232	8:11080675
8:11065003:A:C	0.4284	7.50E-06	-0.005829	0.0013	0.70543	8:11080675

uniqID	*MAF*	*gwasP*	*beta*	*se*	*r2*	*IndSigSNP*
8:11069960:C:T	0.4861	2.40E-08	-0.007195	0.001288	0.817445	8:11080675
8:11070360:C:G	0.497	3.98E-08	-0.007068	0.001286	0.861267	8:11080675
8:11071057:A:G	0.4165	9.10E-09	-0.007484	0.001301	0.736923	8:10988275
8:11072020:A:G	0.3449	4.01E-06	-0.006161	0.001335	0.607352	8:10988275
8:11073402:A:G	0.498	8.03E-08	-0.006912	0.001287	0.864508	8:11080675
8:11073578:C:T	0.4622	5.13E-07	-0.006516	0.001297	0.849973	8:11080675
8:11074036:A:G	0.499	8.95E-08	-0.00689	0.001288	0.868263	8:11080675
8:11074365:C:CTA	0.4841	NA	NA	NA	0.931889	8:11080675
8:11074812:C:T	0.3439	1.01E-05	-0.005909	0.001338	0.605431	8:10988275
8:11076635:G:T	0.4781	1.63E-08	-0.007286	0.001289	0.925	8:11080675
8:11078781:A:C	0.4821	1.68E-08	-0.007282	0.00129	0.924333	8:11080675
8:11078949:A:G	0.4861	1.42E-08	-0.007319	0.00129	0.938932	8:11080675
8:11079367:A:G	0.4841	1.08E-08	-0.007379	0.00129	0.946693	8:11080675
8:11080014:C:T	0.4553	5.47E-07	-0.006535	0.001304	0.850294	8:11080675
8:11080665:A:T	0.492	6.04E-09	-0.00753	0.001294	1	8:11080675
8:11080675:C:T	0.492	4.90E-09	-0.007576	0.001294	1	8:11080675
8:11086942:C:T	0.495	5.29E-08	-0.007097	0.001303	0.890168	8:11080675
8:11087475:A:G	0.4911	1.30E-07	-0.006911	0.001308	0.834855	8:11080675
8:11098992:A:G	0.4821	NA	NA	NA	0.619995	8:11080675
8:11098996:C:T	0.4801	NA	NA	NA	0.61276	8:11080675
8:11109269:C:T	0.4712	2.13E-06	-0.006122	0.00129	0.71771	8:11080675
8:11109303:C:G	0.4612	1.56E-05	-0.005598	0.001295	0.697352	8:11080675
8:11111462:A:T	0.4284	4.95E-05	-0.005259	0.001295	0.621485	8:11080675
8:11113089:A:G	0.4245	9.37E-05	-0.005078	0.001299	0.612932	8:11080675
8:11174484:C:G	0.4016	5.20E-05	0.005358	0.001323	0.662531	8:11235150
8:11176403:A:G	0.4523	3.97E-06	0.005965	0.001292	0.69792	8:11080675
8:11177126:A:C	0.4483	1.47E-06	0.006232	0.001293	0.686706	8:11080675
8:11178093:A:C	0.4523	2.21E-06	0.006122	0.001292	0.69792	8:11080675
8:11179458:G:T	0.4304	1.59E-06	0.006241	0.001299	0.629832	8:11235150
8:11182148:C:G	0.4652	2.18E-05	0.005462	0.001286	0.662583	8:11235150
8:11182455:A:G	0.4612	1.13E-06	0.006786	0.001393	0.653055	8:11235150
8:11182704:A:G	0.4563	2.66E-05	0.005406	0.001286	0.640867	8:11235150

uniqID	*MAF*	*gwasP*	*beta*	*se*	*r2*	*IndSigSNP*
8:11183505:A:G	0.4553	3.26E-05	0.005351	0.001287	0.638003	8:11235150
8:11183765:C:CTTA	0.4553	NA	NA	NA	0.638003	8:11235150
8:11184390:A:C	0.4543	2.57E-05	0.005415	0.001286	0.641485	8:11235150
8:11184396:A:G	0.4543	2.68E-05	0.005403	0.001286	0.641485	8:11235150
8:11184478:C:CAA	0.4632	NA	NA	NA	0.669531	8:11235150
8:11184937:A:G	0.4553	2.64E-05	0.005404	0.001285	0.644337	8:11235150
8:11185096:C:G	0.4771	9.81E-07	0.006302	0.001286	0.8519	8:11235150
8:11185671:G:T	0.4563	1.45E-05	0.005635	0.001299	0.639721	8:11235150
8:11185673:A:AT	0.4901	NA	NA	NA	0.802063	8:11235150
8:11186215:A:C	0.4602	3.62E-05	0.005311	0.001285	0.649019	8:11235150
8:11186453:C:T	0.4801	3.44E-07	0.006561	0.001286	0.834396	8:11235150
8:11186639:C:G	0.4801	3.40E-07	0.006564	0.001286	0.834396	8:11235150
8:11186674:A:G	0.4881	1.86E-07	0.006708	0.001286	0.801425	8:11235150
8:11187078:G:T	0.4563	2.17E-05	0.00546	0.001285	0.639721	8:11235150
8:11187434:C:T	0.4891	1.79E-07	0.006721	0.001287	0.80486	8:11235150
8:11187651:A:G	0.4553	2.69E-05	0.005427	0.001292	0.643212	8:11235150
8:11187675:C:T	0.4553	1.30E-05	-0.006404	0.001468	0.643212	8:11235150
8:11187770:C:G	0.4573	4.47E-05	0.005282	0.001293	0.64146	8:11235150
8:11188532:C:G	0.4811	2.25E-07	-0.007598	0.001467	0.837866	8:11235150
8:11188540:A:G	0.4553	2.99E-06	0.006518	0.001394	0.643212	8:11235150
8:11188586:C:T	0.4543	1.04E-05	-0.006441	0.00146	0.640361	8:11235150
8:11188752:A:G	0.4553	1.60E-05	0.005555	0.001287	0.643212	8:11235150
8:11189488:C:T	0.4861	2.05E-07	0.007409	0.001425	0.814104	8:11235150
8:11189535:C:T	0.4602	NA	NA	NA	0.64791	8:11235150
8:11190647:C:G	0.4592	2.99E-05	0.005363	0.001284	0.652506	8:11235150
8:11191537:C:T	0.4592	2.53E-05	0.00542	0.001286	0.652506	8:11235150
8:11192551:C:G	0.4583	3.02E-05	0.005363	0.001285	0.649614	8:11235150
8:11192593:A:G	0.4791	5.70E-07	0.006436	0.001286	0.844044	8:11235150
8:11193530:C:T	0.4592	4.35E-05	0.005252	0.001284	0.652506	8:11235150
8:11193736:G:T	0.4602	2.98E-05	0.005364	0.001284	0.64791	8:11235150
8:11194457:A:G	0.4592	4.68E-05	0.005248	0.001288	0.652506	8:11235150
8:11194911:A:G	0.4602	3.36E-05	0.005329	0.001284	0.64791	8:11235150

uniqID	*MAF*	*gwasP*	*beta*	*se*	*r2*	*IndSigSNP*
8:11196295:C:G	0.4702	3.43E-05	0.00533	0.001286	0.622412	8:11235150
8:11196970:C:T	0.4771	6.34E-07	0.006417	0.001287	0.850866	8:11235150
8:11197301:C:G	0.4602	2.97E-05	0.005366	0.001284	0.64791	8:11235150
8:11197323:G:T	0.4602	3.12E-05	0.005351	0.001284	0.64791	8:11235150
8:11197598:C:T	0.4602	NA	NA	NA	0.64791	8:11235150
8:11198579:C:CTTTT	0.4602	NA	NA	NA	0.655409	8:11235150
8:11198792:A:G	0.4642	1.17E-06	0.006274	0.00129	0.825073	8:11235150
8:11199584:A:G	0.4652	8.34E-07	0.006362	0.00129	0.827796	8:11235150
8:11199938:A:C	0.4801	3.66E-07	0.00654	0.001285	0.840936	8:11235150
8:11200454:A:AAT	0.4573	NA	NA	NA	0.64146	8:11235150
8:11201504:G:T	0.4732	3.71E-05	0.005313	0.001287	0.634219	8:11235150
8:11201605:A:ATT	0.4901	NA	NA	NA	0.740296	8:11235150
8:11202154:A:G	0.4573	2.01E-05	0.005482	0.001285	0.653158	8:11235150
8:11202960:A:G	0.4573	1.84E-05	0.005506	0.001285	0.653158	8:11235150
8:11203107:A:T	0.4583	1.92E-05	0.005498	0.001285	0.648527	8:11235150
8:11204165:C:T	0.4871	2.13E-07	0.006677	0.001286	0.809925	8:11235150
8:11204184:A:G	0.4592	1.54E-05	0.005561	0.001285	0.650345	8:11235150
8:11204503:C:G	0.4592	2,00E-05	0.005486	0.001285	0.650345	8:11235150
8:11204532:A:G	0.4592	1.66E-05	0.005537	0.001285	0.650345	8:11235150
8:11204755:C:T	0.4891	2.55E-07	0.006629	0.001285	0.817007	8:11235150
8:11205593:C:T	0.4592	1.59E-05	0.005566	0.001289	0.650345	8:11235150
8:11205602:C:T	0.4881	2.50E-07	0.006647	0.001288	0.820528	8:11235150
8:11205654:A:T	0.4592	1.37E-05	0.005607	0.001289	0.650345	8:11235150
8:11205665:C:G	0.4592	1.42E-05	0.005597	0.001289	0.650345	8:11235150
8:11205817:T:TA	0.4632	NA	NA	NA	0.631986	8:11235150
8:11206220:C:G	0.4592	1.38E-05	0.005593	0.001286	0.650345	8:11235150
8:11206262:C:T	0.4592	1.42E-05	0.005586	0.001286	0.650345	8:11235150
8:11206363:C:G	0.4602	3.01E-06	0.006502	0.001391	0.645733	8:11235150
8:11206543:A:G	0.4602	1.36E-05	0.005597	0.001286	0.646814	8:11235150
8:11206627:C:G	0.4592	1.39E-05	0.005591	0.001286	0.650345	8:11235150
8:11207326:A:C	0.4592	1.35E-05	0.005596	0.001285	0.650345	8:11235150
8:11207367:A:C	0.4881	2.45E-07	0.006635	0.001285	0.820528	8:11235150

uniqID	***MAF***	***gwasP***	***beta***	***se***	***r2***	***IndSigSNP***
8:11207431:C:T	0.4592	1.34E-05	0.005597	0.001285	0.650345	8:11235150
8:11207508:C:T	0.4592	1.34E-05	0.005598	0.001285	0.650345	8:11235150
8:11207672:C:G	0.4583	1.33E-05	0.005619	0.001289	0.648527	8:11235150
8:11208903:A:G	0.4781	2.41E-07	0.006655	0.001288	0.847193	8:11235150
8:11209499:C:T	0.4612	1.58E-05	0.005555	0.001286	0.656159	8:11235150
8:11210823:G:T	0.4781	1.95E-06	-0.007059	0.001482	0.854402	8:11235150
8:11210824:G:T	0.4781	1.66E-06	-0.007106	0.001482	0.854402	8:11235150
8:11210828:C:T	0.4592	3.24E-05	-0.006162	0.001482	0.657862	8:11235150
8:11210983:A:G	0.4592	2.05E-05	0.005482	0.001286	0.657862	8:11235150
8:11211068:C:T	0.4592	2.10E-05	0.005475	0.001286	0.657862	8:11235150
8:11211302:G:T	0.4742	3.52E-07	0.006559	0.001287	0.839403	8:11235150
8:11212081:A:G	0.4612	1.13E-05	0.005654	0.001287	0.670148	8:11235150
8:11212599:C:T	0.4662	1.35E-06	0.006219	0.001286	0.69035	8:11235150
8:11212650:A:AT	0.4662	NA	NA	NA	0.69035	8:11235150
8:11212778:C:T	0.4682	9.23E-07	0.00634	0.001291	0.683301	8:11235150
8:11212811:A:C	0.4682	9.36E-07	0.006331	0.00129	0.683301	8:11235150
8:11212812:C:G	0.4682	9.88E-07	0.006318	0.00129	0.683301	8:11235150
8:11212875:C:G	0.4662	1.18E-06	0.006242	0.001284	0.69035	8:11235150
8:11213092:A:G	0.4662	5.88E-07	0.006428	0.001286	0.69035	8:11235150
8:11213250:C:T	0.4672	3.69E-07	0.006543	0.001286	0.685782	8:11235150
8:11213363:C:T	0.4662	4.12E-07	0.006513	0.001285	0.69035	8:11235150
8:11213389:C:T	0.4662	4.44E-07	0.006497	0.001286	0.69035	8:11235150
8:11213589:G:T	0.4652	3.74E-08	0.007102	0.00129	0.886279	8:11235150
8:11213881:C:G	0.4662	6.51E-07	0.006392	0.001284	0.69035	8:11235150
8:11214455:A:G	0.4831	2.89E-08	0.00714	0.001286	0.837235	8:11235150
8:11214972:A:G	0.4831	2.80E-08	0.007146	0.001286	0.837235	8:11235150
8:11215617:C:G	0.4662	2.55E-08	0.007177	0.001288	0.882787	8:11235150
8:11215868:A:AT	0.4751	NA	NA	NA	0.626147	8:11235150
8:11216761:G:T	0.4692	3.29E-07	0.006563	0.001284	0.696349	8:11235150
8:11217284:C:T	0.4662	2,00E-07	-0.007657	0.001472	0.896929	8:11235150
8:11217441:A:G	0.4722	2.63E-07	0.006613	0.001284	0.684727	8:11235150
8:11218893:A:G	0.4702	3.80E-07	0.006532	0.001285	0.681785	8:11235150

uniqID	*MAF*	*gwasP*	*beta*	*se*	*r2*	*IndSigSNP*
8:11219334:A:G	0.4662	2.87E-08	0.007168	0.001291	0.882426	8:11235150
8:11219386:G:T	0.4682	2.30E-07	0.006663	0.001287	0.688768	8:11235150
8:11219781:C:T	0.4652	2.03E-08	0.007242	0.00129	0.886279	8:11235150
8:11220846:C:CAA	0.4364	NA	NA	NA	0.632273	8:11235150
8:11221313:C:T	0.4702	3.41E-07	0.006576	0.001289	0.731532	8:11235150
8:11223022:A:C	0.4851	6.75E-08	0.006942	0.001285	0.791126	8:11235150
8:11223793:G:T	0.4801	9.29E-08	-0.006872	0.001286	0.79062	8:11235150
8:11224313:C:T	0.4811	9.68E-08	0.006866	0.001287	0.780078	8:11235150
8:11225168:A:G	0.4801	3.18E-08	-0.007119	0.001286	0.733889	8:11235150
8:11225480:C:T	0.4771	3.66E-08	-0.007088	0.001286	0.747343	8:11235150
8:11225910:A:G	0.4761	3.27E-08	-0.007125	0.001288	0.751855	8:11235150
8:11226071:A:G	0.4692	2.43E-09	-0.007704	0.00129	0.923761	8:11235150
8:11226456:A:C	0.4602	1.29E-09	-0.007853	0.001293	0.95791	8:11235150
8:11227104:A:G	0.4751	9.09E-08	-0.00687	0.001285	0.769909	8:11235150
8:11227406:A:G	0.4622	4.75E-09	-0.007569	0.001292	0.965461	8:11235150
8:11227885:C:T	0.4592	4.90E-09	-0.007563	0.001292	0.961941	8:11235150
8:11228006:A:G	0.4761	1.74E-07	-0.006708	0.001283	0.772167	8:11235150
8:11228100:A:G	0.4622	4.98E-09	-0.007556	0.001291	0.965461	8:11235150
8:11228254:A:G	0.4791	5.05E-08	-0.007003	0.001284	0.773028	8:11235150
8:11228672:A:G	0.4771	4.16E-08	0.00705	0.001285	0.768449	8:11235150
8:11229319:C:G	0.4592	5.90E-09	-0.007531	0.001293	0.969752	8:11235150
8:11229638:C:T	0.4652	8.20E-09	-0.007456	0.001293	0.969176	8:11235150
8:11229889:A:G	0.4841	1.82E-07	0.006827	0.001308	0.655714	8:11235150
8:11230206:G:T	0.4622	5.51E-09	-0.007537	0.001292	0.980914	8:11235150
8:11230259:A:T	0.4622	5.79E-09	-0.007527	0.001292	0.980914	8:11235150
8:11230574:A:G	0.4632	4.15E-09	-0.007598	0.001292	0.977052	8:11235150
8:11231249:C:T	0.4891	3.38E-09	0.007613	0.001287	0.883242	8:11235150
8:11231354:G:T	0.4881	6.46E-09	0.007478	0.001287	0.893708	8:11235150
8:11231886:A:G	0.4722	4.41E-09	-0.007569	0.001289	0.950556	8:11235150
8:11232343:C:G	0.4662	5.42E-09	-0.007538	0.001291	0.980818	8:11235150
8:11232788:A:G	0.4642	2.99E-09	-0.007669	0.001292	0.988526	8:11235150
8:11232860:A:G	0.4672	4.79E-09	-0.007565	0.001291	0.984679	8:11235150

uniqID	*MAF*	*gwasP*	*beta*	*se*	*r2*	*IndSigSNP*
8:11233318:A:C	0.4781	3.74E-09	-0.007603	0.001289	0.943812	8:11235150
8:11233419:G:T	0.4781	4.35E-09	-0.007571	0.001289	0.943812	8:11235150
8:11233582:A:G	0.4781	5.16E-09	-0.007535	0.001289	0.943812	8:11235150
8:11233659:A:G	0.4781	5.18E-09	-0.007534	0.001289	0.943812	8:11235150
8:11233917:C:T	0.4642	5.51E-09	-0.007534	0.001291	0.988526	8:11235150
8:11233958:A:G	0.4642	5.37E-09	-0.007539	0.001291	0.988526	8:11235150
8:11234298:C:T	0.4672	5.18E-09	-0.007542	0.00129	1	8:11235150
8:11234367:G:T	0.4672	5.57E-09	-0.007527	0.00129	1	8:11235150
8:11234500:A:T	0.4801	5.04E-09	-0.007536	0.001288	0.9367	8:11235150
8:11234520:A:G	0.4672	5.04E-09	-0.007548	0.00129	1	8:11235150
8:11234613:A:T	0.4672	5.78E-09	-0.007523	0.001291	1	8:11235150
8:11234626:C:T	0.4672	5.61E-09	-0.00753	0.001291	1	8:11235150
8:11234780:C:G	0.4672	5.47E-09	-0.007531	0.00129	1	8:11235150
8:11234844:A:G	0.4672	5.31E-09	-0.007537	0.00129	1	8:11235150
8:11234885:C:G	0.4672	5.12E-09	-0.007541	0.00129	1	8:11235150
8:11235136:C:G	0.4672	6.04E-09	-0.00751	0.00129	1	8:11235150
8:11235150:A:C	0.4672	1.15E-09	-0.00851	0.001397	1	8:11235150
8:11235360:A:C	0.4682	5.59E-09	-0.007524	0.00129	0.996144	8:11235150
8:11235393:A:G	0.4662	6.65E-09	-0.007485	0.00129	0.996157	8:11235150
8:11235497:A:G	0.4672	5.70E-09	-0.007514	0.001289	1	8:11235150
8:11235579:G:T	0.4672	7.06E-09	-0.007475	0.00129	1	8:11235150
8:11235605:G:T	0.4672	6.55E-09	-0.007492	0.00129	1	8:11235150
8:11235614:A:G	0.4841	7.33E-08	-0.006929	0.001286	0.808633	8:11235150
8:11235735:C:T	0.4672	5.85E-09	-0.007512	0.00129	1	8:11235150
8:11235910:A:G	0.4592	9.37E-09	-0.007423	0.001292	0.970174	8:11235150
8:11236392:C:T	0.4672	4.70E-09	-0.007572	0.001292	1	8:11235150
8:11236413:C:T	0.4672	4.10E-09	-0.007601	0.001292	1	8:11235150
8:11236419:C:T	0.4672	4.02E-09	-0.007605	0.001292	1	8:11235150
8:11236572:A:T	0.4672	4.12E-09	-0.00758	0.001288	1	8:11235150
8:11236681:G:T	0.4672	3.92E-09	-0.007595	0.001289	1	8:11235150
8:11236685:C:T	0.4672	3.93E-09	-0.007594	0.001289	1	8:11235150
8:11236809:C:T	0.4672	6.35E-09	-0.007484	0.001288	1	8:11235150

uniqID	*MAF*	*gwasP*	*beta*	*se*	*r2*	*IndSigSNP*
8:11236850:C:T	0.4672	5.81E-09	-0.007503	0.001288	1	8:11235150
8:11236964:C:T	0.4672	5.81E-09	-0.007504	0.001288	1	8:11235150
8:11236975:A:G	0.4672	5.40E-09	-0.007528	0.001289	1	8:11235150
8:11237330:C:G	0.4046	4.35E-07	0.007593	0.001501	0.884308	8:11249261
8:11237477:A:G	0.4672	4.26E-09	-0.007577	0.001289	1	8:11235150
8:11237480:A:G	0.4672	4.26E-09	-0.007577	0.001289	1	8:11235150
8:11237587:C:CAAAA	0.4592	NA	NA	NA	0.970174	8:11235150
8:11237591:A:C	0.4592	0.000568	-0.009538	0.002766	0.970174	8:11235150
8:11237756:A:C	0.4612	9.75E-09	-0.007376	0.001285	0.962473	8:11235150
8:11237773:C:T	0.4622	9.23E-09	-0.007413	0.00129	0.958547	8:11235150
8:11238029:C:T	0.4553	6.40E-09	-0.007501	0.001291	0.955667	8:11235150
8:11238315:C:T	0.4751	2.07E-07	0.007624	0.001467	0.752395	8:11235150
8:11238316:G:T	0.494	1.04E-07	0.007931	0.00149	0.778765	8:11235150
8:11238332:G:T	0.4911	1.07E-07	-0.006844	0.001287	0.79002	8:11235150
8:11238587:A:G	0.492	8.36E-08	-0.006905	0.001287	0.798452	8:11235150
8:11238597:C:T	0.4602	8.92E-09	-0.007428	0.001291	0.958918	8:11235150
8:11239017:A:T	0.4602	9.87E-09	-0.007409	0.001291	0.958918	8:11235150
8:11239054:C:T	0.4602	1.03E-08	-0.007403	0.001292	0.958918	8:11235150
8:11239078:G:T	0.4602	9.43E-09	-0.007419	0.001291	0.958918	8:11235150
8:11239137:A:G	0.4602	8.81E-09	-0.007434	0.001291	0.958918	8:11235150
8:11239297:G:GATAG	0.001988	NA	NA	NA	0.962882	8:11235150
8:11239297:G:GATAT	0.4573	NA	NA	NA	0.962882	8:11235150
8:11239352:A:G	0.4602	8.18E-09	-0.00745	0.001291	0.958918	8:11235150
8:11239510:A:G	0.4612	7.93E-09	-0.00746	0.001292	0.954981	8:11235150
8:11239565:C:T	0.4612	7.99E-09	-0.007459	0.001292	0.954981	8:11235150
8:11239640:C:T	0.4612	6.44E-09	-0.00751	0.001293	0.954981	8:11235150
8:11239762:A:T	0.4801	4.78E-09	-0.007547	0.001288	0.929242	8:11235150
8:11239942:C:CT	0.3628	NA	NA	NA	0.649271	8:11235150
8:11240571:C:T	0.33	1.57E-06	-0.006579	0.001369	0.705372	8:11249261
8:11241935:G:GT	0.4602	NA	NA	NA	0.951452	8:11235150
8:11242025:G:T	0.4553	2.28E-08	-0.007269	0.001299	0.948454	8:11235150
8:11242039:T:TA	0.4583	NA	NA	NA	0.959118	8:11235150

uniqID	*MAF*	*gwasP*	*beta*	*se*	*r2*	*IndSigSNP*
8:11242632:A:G	0.4662	3.57E-09	-0.007632	0.001292	0.973323	8:11235150
8:11243126:C:G	0.4563	6.27E-09	-0.007524	0.001294	0.952167	8:11235150
8:11244841:A:G	0.3678	9.37E-08	-0.007179	0.001344	0.832392	8:11249261
8:11245064:C:T	0.3897	7.04E-08	-0.007185	0.001332	0.905741	8:11249261
8:11245303:C:G	0.4781	4.04E-09	0.007598	0.001291	0.913721	8:11235150
8:11245562:A:T	0.3887	NA	NA	NA	0.932951	8:11249261
8:11247298:A:G	0.3887	5.38E-08	0.007251	0.001332	0.932951	8:11249261
8:11247814:A:G	0.3887	1.46E-08	0.007548	0.001331	0.956162	8:11249261
8:11248500:C:T	0.337	8.99E-07	0.006669	0.001357	0.828557	8:11249261
8:11248956:C:T	0.338	6.14E-07	0.006768	0.001356	0.839745	8:11249261
8:11249010:G:T	0.4722	4.78E-09	0.007569	0.001292	0.847937	8:11235150
8:11249261:C:T	0.3797	6.14E-09	0.008415	0.001447	1	8:11249261
8:11250848:A:C	0.337	4.88E-07	0.00692	0.001374	0.822332	8:11249261
8:11251175:A:G	0.4881	2.43E-08	0.007353	0.001317	0.728006	8:11235150
8:11251705:A:G	0.493	1.18E-07	-0.00695	0.001311	0.743345	8:11235150
8:11252170:C:T	0.4632	2.87E-07	-0.00685	0.001334	0.671683	8:11235150
8:11252425:A:C	0.4423	4.46E-08	0.00745	0.001361	0.659454	8:11235150
8:11309192:C:T	0.4433	6.59E-06	-0.005835	0.001294	0.621606	8:11425809
8:11336781:A:G	0.4185	4.83E-06	-0.006471	0.001414	0.637784	8:11425809
8:11338146:A:G	0.4175	5.23E-05	-0.005278	0.001304	0.635948	8:11425809
8:11355602:C:G	0.4632	3.34E-05	-0.005364	0.001292	0.606363	8:11425809
8:11358156:C:T	0.4692	3.61E-05	-0.005349	0.001294	0.713524	8:11425809
8:11361261:C:G	0.4563	4.75E-05	-0.005248	0.001289	0.6958	8:11425809
8:11361850:G:GAGGA	0.4751	NA	NA	NA	0.713843	8:11425809
8:11362275:A:C	0.4672	3.98E-05	-0.005321	0.001294	0.712836	8:11425809
8:11362277:G:T	0.4672	4.16E-05	-0.005308	0.001294	0.712836	8:11425809
8:11382367:A:G	0.4165	1.19E-05	0.00572	0.001305	0.656046	8:11425809
8:11384556:C:T	0.4404	0.000201	0.004812	0.001294	0.625148	8:11425809
8:11392093:A:C	0.4652	6.50E-06	-0.005808	0.001287	0.707857	8:11425809
8:11393764:A:G	0.4801	4.43E-05	-0.005257	0.001287	0.695105	8:11425809
8:11395079:A:G	0.4831	6.06E-05	-0.005153	0.001284	0.697682	8:11425809
8:11396856:A:C	0.4612	1.03E-05	0.005674	0.001286	0.698214	8:11425809

uniqID	*MAF*	*gwasP*	*beta*	*se*	*r2*	*IndSigSNP*
8:11396874:A:G	0.4811	7.14E-05	-0.005095	0.001282	0.698763	8:11425809
8:11397073:A:C	0.4811	5.52E-05	-0.005176	0.001283	0.698763	8:11425809
8:11397086:A:T	0.4811	5.79E-05	-0.005162	0.001283	0.698763	8:11425809
8:11397457:C:G	0.4732	9.84E-05	0.005004	0.001284	0.691905	8:11425809
8:11398865:A:G	0.4791	3.94E-05	-0.005281	0.001284	0.706203	8:11425809
8:11398953:C:T	0.4851	0.000125	-0.004939	0.001287	0.686199	8:11425809
8:11399484:A:T	0.4791	4.13E-05	-0.005267	0.001284	0.706203	8:11425809
8:11400628:C:G	0.4712	1.94E-06	0.006636	0.001393	0.699514	8:11425809
8:11400680:A:G	0.4791	3.78E-05	-0.0053	0.001285	0.706203	8:11425809
8:11400944:G:T	0.4791	3.87E-05	-0.005302	0.001288	0.706203	8:11425809
8:11401116:A:G	0.4801	3.37E-05	-0.005338	0.001286	0.703541	8:11425809
8:11402347:A:G	0.4712	9.45E-05	0.005033	0.001288	0.694281	8:11425809
8:11410513:A:C	0.2982	1.85E-06	0.006624	0.001388	0.746004	8:11417582
8:11411005:C:G	0.4553	1.01E-07	-0.006904	0.001296	0.748224	8:11425809
8:11415184:C:G	0.3549	1.95E-07	-0.006937	0.001332	0.838325	8:11417582
8:11415572:A:G	0.3539	1.50E-07	-0.006987	0.001329	0.841966	8:11417582
8:11415597:C:T	0.3539	1.44E-07	-0.006997	0.001329	0.841966	8:11417582
8:11415794:A:T	0.3539	1.36E-07	-0.00701	0.001329	0.841966	8:11417582
8:11415812:C:T	0.4911	7.39E-08	-0.006968	0.001294	0.958834	8:11425809
8:11416171:C:T	0.3539	1.27E-07	-0.007027	0.001329	0.841966	8:11417582
8:11416428:C:T	0.3529	1.33E-07	-0.00702	0.00133	0.837485	8:11417582
8:11416635:A:G	0.3529	1.35E-07	-0.007011	0.001329	0.837485	8:11417582
8:11416885:C:T	0.4781	4.30E-07	-0.006565	0.001298	0.925956	8:11425809
8:11417016:A:ACT	0.4791	NA	NA	NA	0.929647	8:11425809
8:11417144:C:T	0.4771	1.89E-07	-0.00675	0.001295	0.922559	8:11425809
8:11417150:G:T	0.4771	1.39E-07	-0.006817	0.001293	0.922559	8:11425809
8:11417257:A:G	0.4781	2.11E-07	-0.006716	0.001293	0.925956	8:11425809
8:11417493:C:T	0.4513	1.37E-07	0.00681	0.001292	0.818259	8:11425809
8:11417582:C:T	0.339	9.09E-11	-0.010087	0.001555	1	8:11417582
8:11418385:A:G	0.3211	3.01E-07	0.006938	0.001353	0.929555	8:11417582
8:11418773:C:T	0.3231	3.87E-07	0.006869	0.001353	0.921244	8:11417582
8:11419335:A:AT	0.4652	NA	NA	NA	0.755512	8:11425809

uniqID	*MAF*	*gwasP*	*beta*	*se*	*r2*	*IndSigSNP*
8:11419852:C:T	0.332	1.84E-07	0.007031	0.001347	0.964019	8:11417582
8:11419861:G:T	0.332	2,00E-07	0.007011	0.001347	0.964019	8:11417582
8:11420104:C:G	0.332	9.13E-08	0.007198	0.001346	0.964019	8:11417582
8:11420221:A:G	0.33	9.01E-08	0.007202	0.001346	0.964158	8:11417582
8:11420295:C:T	0.332	1.02E-07	0.00718	0.001348	0.964019	8:11417582
8:11421016:A:G	0.332	1.02E-07	0.007167	0.001345	0.964019	8:11417582
8:11421358:A:C	0.332	9.89E-08	0.007175	0.001345	0.964019	8:11417582
8:11421384:C:T	0.333	1.35E-07	0.007098	0.001345	0.96009	8:11417582
8:11421793:C:T	0.333	1.10E-07	0.00715	0.001345	0.951683	8:11417582
8:11422045:A:G	0.4901	1.33E-07	-0.006825	0.001293	0.96991	8:11425809
8:11422130:C:T	0.4901	1.20E-07	-0.006846	0.001292	0.96991	8:11425809
8:11422170:A:G	0.332	2.44E-07	-0.006958	0.001347	0.947825	8:11417582
8:11422289:C:G	0.334	5.21E-08	0.007315	0.001343	0.947564	8:11417582
8:11422442:A:C	0.4901	1.51E-07	-0.006787	0.001292	0.96991	8:11425809
8:11422491:A:C	0.4901	NA	NA	NA	0.96991	8:11425809
8:11422492:C:G	0.4901	NA	NA	NA	0.96991	8:11425809
8:11422494:A:AT	0.4463	NA	NA	NA	0.810571	8:11425809
8:11422521:G:GA	0.3718	NA	NA	NA	0.808269	8:11417582
8:11422861:C:G	0.4473	1.55E-07	0.006765	0.001289	0.813165	8:11425809
8:11422936:C:T	0.4473	1.44E-07	0.006787	0.001289	0.807083	8:11425809
8:11423072:A:G	0.4483	2.05E-07	0.006704	0.00129	0.81647	8:11425809
8:11423083:A:C	0.492	1.34E-07	-0.006813	0.001291	0.977574	8:11425809
8:11423142:A:G	0.3579	5.51E-07	0.006659	0.001329	0.787159	8:11417582
8:11423434:A:G	0.3231	3.55E-07	0.006881	0.001351	0.897287	8:11417582
8:11423537:A:G	0.325	2.68E-07	0.00694	0.001348	0.905252	8:11417582
8:11423781:C:G	0.3708	2.64E-07	-0.006814	0.001323	0.818804	8:11417582
8:11425077:C:T	0.3549	3.03E-07	0.006804	0.001328	0.775418	8:11417582
8:11425081:G:T	0.3221	1.91E-07	0.007043	0.001351	0.863135	8:11417582
8:11425105:G:T	0.492	9.06E-08	-0.00689	0.001288	0.977574	8:11425809
8:11425809:G:T	0.4861	4.79E-08	-0.007038	0.001288	1	8:11425809
8:11426400:G:T	0.333	2.13E-07	0.006984	0.001345	0.919948	8:11417582
8:11426790:C:G	0.4891	1.45E-07	-0.006786	0.001289	0.981162	8:11425809

uniqID	*MAF*	*gwasP*	*beta*	*se*	*r2*	*IndSigSNP*
8:11427133:G:T	0.4901	1.97E-07	-0.006736	0.001294	0.984964	8:11425809
8:11427341:G:T	0.327	5.87E-07	0.006715	0.001343	0.889138	8:11417582
8:11427637:A:T	0.4901	7.66E-08	-0.00694	0.00129	0.985009	8:11425809
8:11428395:C:T	0.4911	1.30E-07	-0.006843	0.001295	0.981284	8:11425809
8:11430485:A:G	0.2962	5.23E-07	0.006922	0.001378	0.748053	8:11417582
8:11430990:C:T	0.2913	1.19E-06	0.00673	0.001385	0.723619	8:11417582
8:11431558:T:TAC	0.4523	NA	NA	NA	0.854002	8:11425809
8:11431943:T:TAA	0.3141	NA	NA	NA	0.67551	8:11417582
8:11432085:A:ACG	0.325	NA	NA	NA	0.667433	8:11417582
8:11432085:A:ACACG	0.000994	NA	NA	NA	0.667433	8:11417582
8:11432438:C:G	0.3042	9.39E-07	-0.006752	0.001376	0.752428	8:11417582
8:11432453:C:T	0.2893	9.86E-07	0.006789	0.001386	0.732607	8:11417582
8:11432946:A:C	0.4483	5.74E-07	-0.006463	0.001292	0.83584	8:11425809
8:11433780:C:T	0.4384	8.69E-07	-0.006359	0.001292	0.807192	8:11425809
8:11433909:C:T	0.4473	5.25E-07	-0.006483	0.001291	0.833136	8:11425809
8:11434176:A:T	0.4473	4.54E-07	-0.00652	0.001291	0.833136	8:11425809
8:11434232:C:T	0.4473	4.74E-07	-0.00651	0.001292	0.833136	8:11425809
8:11434415:C:CTCGGTTT	0.4423	NA	NA	NA	0.790012	8:11425809
8:11434792:C:G	0.4513	9.03E-07	-0.006345	0.001291	0.844127	8:11425809
8:11434929:C:T	0.2942	5.19E-06	-0.006317	0.001385	0.714427	8:11417582
8:11435049:A:G	0.4473	4.78E-07	-0.006507	0.001291	0.832544	8:11425809
8:11435291:G:GTGGC	0.4543	NA	NA	NA	0.817048	8:11425809
8:11435516:C:T	0.4384	6.63E-07	-0.006428	0.001292	0.813392	8:11425809
8:11435564:C:T	0.4473	5.09E-07	-0.006492	0.001292	0.83951	8:11425809
8:11435927:C:G	0.4473	4.73E-07	-0.006511	0.001292	0.83951	8:11425809
8:11438064:G:T	0.2903	6.71E-07	0.00689	0.001385	0.723808	8:11417582
8:11439225:A:G	0.2793	3.56E-05	0.005831	0.00141	0.610589	8:11417582
8:11440019:A:G	0.4423	1.03E-06	-0.006315	0.001292	0.804337	8:11425809
8:11444516:A:G	0.4354	1.64E-06	0.006207	0.001294	0.771327	8:11425809
8:11444837:C:T	0.4354	1.94E-06	0.006164	0.001294	0.778812	8:11425809
8:11446421:A:G	0.4036	3.65E-05	-0.005401	0.001307	0.661948	8:11425809
8:11446637:A:G	0.4016	1.24E-05	-0.006552	0.001498	0.657364	8:11425809

uniqID	*MAF*	*gwasP*	*beta*	*se*	*r2*	*IndSigSNP*
8:11446652:A:G	0.3946	NA	NA	NA	0.637267	8:11425809
8:11446680:C:T	0.4016	2.80E-05	-0.005481	0.001307	0.657364	8:11425809
8:11446800:C:T	0.4016	2.59E-05	-0.005499	0.001306	0.651155	8:11425809
8:11446868:C:T	0.4016	2.12E-05	-0.005558	0.001306	0.657364	8:11425809
8:11446955:A:G	0.4016	2.29E-05	-0.005534	0.001306	0.657364	8:11425809
8:11447093:A:G	0.4433	1.70E-06	-0.006203	0.001295	0.800069	8:11425809
8:11447119:C:T	0.4016	1.76E-05	-0.005611	0.001306	0.657364	8:11425809
8:11447679:C:G	0.4036	3.17E-05	-0.00544	0.001307	0.654603	8:11425809
8:11448659:C:G	0.3847	6.46E-05	-0.005279	0.001321	0.611065	8:11425809
8:11450133:A:G	0.3966	2.48E-05	0.00551	0.001306	0.639348	8:11425809
8:11450422:A:G	0.4453	2.79E-06	0.006067	0.001294	0.791575	8:11425809
8:11450587:G:T	0.3976	3.29E-05	0.005428	0.001306	0.641002	8:11425809
8:11460909:T:TA	0.3926	NA	NA	NA	0.61754	8:11425809
8:11461111:A:G	0.4652	1.47E-06	-0.006266	0.0013	0.644552	8:11425809
8:11466745:A:T	0.4592	4.59E-07	-0.006621	0.001312	0.635504	8:11425809
8:11467557:C:G	0.4612	7.39E-07	-0.006544	0.001321	0.628224	8:11425809
12:62830952:C:T	0.3022	9.57E-05	-0.005377	0.001378	0.673865	12:62995340
12:62831343:C:T	0.3022	8.01E-05	-0.005448	0.00138	0.673865	12:62995340
12:62837767:A:G	0.3002	6.11E-05	-0.005533	0.001379	0.675634	12:62995340
12:62847085:C:G	0.3559	5.24E-08	-0.007287	0.001338	0.973564	12:62995340
12:62848152:A:G	0.3559	7.19E-08	-0.007229	0.001341	0.973564	12:62995340
12:62849418:A:G	0.3579	1.03E-07	-0.007113	0.001336	0.964868	12:62995340
12:62851080:A:AC	0.3608	NA	NA	NA	0.952209	12:62995340
12:62852271:A:G	0.3608	5.21E-08	-0.007248	0.00133	0.952209	12:62995340
12:62852916:A:G	0.3588	7.48E-08	-0.007171	0.001332	0.96055	12:62995340
12:62855388:C:T	0.3588	7.46E-08	-0.007177	0.001333	0.96055	12:62995340
12:62858342:A:G	0.3539	6.11E-08	-0.00725	0.001338	0.982221	12:62995340
12:62858561:C:T	0.3549	6.44E-08	-0.007237	0.001338	0.986783	12:62995340
12:62859241:A:AC	0.3549	NA	NA	NA	0.977943	12:62995340
12:62861935:T:TA	0.4165	NA	NA	NA	0.757895	12:62995340
12:62862739:G:T	0.3608	6.77E-08	-0.007213	0.001335	0.960943	12:62995340
12:62865152:C:T	0.3549	5.11E-08	-0.007293	0.001338	0.986783	12:62995340

uniqID	*MAF*	*gwasP*	*beta*	*se*	*r2*	*IndSigSNP*
12:62865291:A:G	0.3549	5.94E-08	-0.007255	0.001337	0.986783	12:62995340
12:62868455:C:T	0.3549	5.77E-08	-0.007264	0.001338	0.986783	12:62995340
12:62868499:C:T	0.3549	1.11E-07	0.008098	0.001524	0.986783	12:62995340
12:62868500:A:C	0.3549	6.19E-08	-0.007249	0.001338	0.986783	12:62995340
12:62870574:C:G	0.3598	4.74E-08	-0.007274	0.001331	0.965221	12:62995340
12:62875839:C:T	0.3598	6.53E-08	-0.007197	0.001331	0.965221	12:62995340
12:62880276:C:G	0.3598	6.07E-08	-0.007214	0.001331	0.965221	12:62995340
12:62884714:A:ATTT	0.3598	NA	NA	NA	0.965221	12:62995340
12:62884972:A:G	0.3598	5.86E-08	-0.007222	0.001331	0.965221	12:62995340
12:62886001:A:T	0.3608	5.67E-08	-0.007231	0.001331	0.960943	12:62995340
12:62886372:C:G	0.3598	1.29E-07	0.007999	0.001514	0.965221	12:62995340
12:62886649:C:T	0.3598	9.77E-08	-0.00712	0.001335	0.965221	12:62995340
12:62888219:A:C	0.3608	5.91E-08	-0.00722	0.001331	0.960943	12:62995340
12:62889402:A:G	0.3598	8.05E-08	-0.007179	0.001337	0.965221	12:62995340
12:62889409:A:G	0.3598	7.62E-08	-0.007189	0.001336	0.965221	12:62995340
12:62890491:A:C	0.3598	6.78E-08	-0.007187	0.001331	0.965221	12:62995340
12:62891828:C:CT	0.3787	NA	NA	NA	0.861695	12:62995340
12:62894058:A:G	0.3598	6.59E-08	-0.007194	0.001331	0.965221	12:62995340
12:62897930:A:G	0.3598	6.28E-08	-0.007206	0.001331	0.965221	12:62995340
12:62898111:A:G	0.3598	5.09E-08	-0.007258	0.001331	0.965221	12:62995340
12:62898463:C:CT	0.3638	NA	NA	NA	0.94849	12:62995340
12:62898490:C:T	0.3598	6.25E-08	-0.007207	0.001331	0.965221	12:62995340
12:62902420:C:T	0.3598	5.74E-08	-0.007224	0.00133	0.965221	12:62995340
12:62903639:A:G	0.3598	6.25E-08	-0.007203	0.00133	0.965221	12:62995340
12:62903655:G:GT	0.3698	NA	NA	NA	0.915038	12:62995340
12:62903793:A:G	0.3598	6.33E-08	-0.0072	0.00133	0.965221	12:62995340
12:62904252:C:T	0.3598	5.75E-08	-0.007223	0.00133	0.965221	12:62995340
12:62905380:C:CA	0.3598	NA	NA	NA	0.965221	12:62995340
12:62905772:A:AC	0.3598	NA	NA	NA	0.965221	12:62995340
12:62906488:A:G	0.3598	7.62E-08	-0.007157	0.00133	0.965221	12:62995340
12:62910714:A:T	0.3598	7.50E-08	-0.00716	0.00133	0.965221	12:62995340
12:62914776:T:TA	0.3519	NA	NA	NA	1	12:62995340

uniqID	*MAF*	*gwasP*	*beta*	*se*	*r2*	*IndSigSNP*
12:62917704:A:G	0.3549	5.09E-08	-0.007282	0.001336	0.986783	12:62995340
12:62920860:C:G	0.3549	5.24E-08	-0.007275	0.001336	0.986783	12:62995340
12:62921257:C:T	0.3549	5.06E-08	-0.007284	0.001336	0.986783	12:62995340
12:62922143:A:G	0.3549	5.29E-08	-0.007273	0.001336	0.986783	12:62995340
12:62926398:A:G	0.3549	6.74E-08	-0.007232	0.001339	0.986783	12:62995340
12:62928006:T:TA	0.3529	NA	NA	NA	0.959846	12:62995340
12:62928633:A:G	0.3549	8.34E-08	-0.007167	0.001336	0.986783	12:62995340
12:62930621:A:G	0.3549	6,00E-08	-0.007243	0.001336	0.986783	12:62995340
12:62930798:C:T	0.3549	5.93E-08	-0.007246	0.001336	0.986783	12:62995340
12:62931846:A:ATTTGT	0.3549	NA	NA	NA	0.986783	12:62995340
12:62932816:A:G	0.3549	5.86E-08	-0.007249	0.001336	0.986783	12:62995340
12:62935154:C:CA	0.3897	NA	NA	NA	0.77463	12:62995340
12:62935705:C:T	0.3549	7.53E-08	-0.007206	0.001339	0.986783	12:62995340
12:62937348:G:T	0.3887	1.62E-06	-0.006345	0.001322	0.686439	12:62995340
12:62937532:C:T	0.3618	3.52E-08	-0.007354	0.001333	0.93003	12:62995340
12:62939055:C:T	0.3549	5.24E-08	-0.007282	0.001337	0.986689	12:62995340
12:62941426:C:T	0.3549	4.97E-08	-0.007294	0.001337	0.986689	12:62995340
12:62941444:A:G	0.3549	5.07E-08	-0.00729	0.001337	0.986689	12:62995340
12:62941837:A:G	0.3549	4.93E-08	-0.007297	0.001337	0.986689	12:62995340
12:62941929:A:T	0.3529	5.54E-08	-0.007269	0.001337	0.995524	12:62995340
12:62942258:C:T	0.3519	2.83E-08	-0.007438	0.001339	1	12:62995340
12:62945158:G:GGAAACAACCTGACGAT	0.3539	NA	NA	NA	0.991112	12:62995340
12:62945245:A:G	0.3539	3.73E-08	-0.007386	0.001341	0.991112	12:62995340
12:62945970:C:G	0.3549	2.38E-08	-0.007478	0.001339	0.986783	12:62995340
12:62948736:A:G	0.3519	2.58E-08	-0.007468	0.00134	1	12:62995340
12:62949110:A:C	0.3569	3.38E-08	-0.007376	0.001335	0.977907	12:62995340
12:62950556:C:G	0.3539	2.66E-08	-0.007452	0.001339	0.991112	12:62995340
12:62952696:C:T	0.3539	3.33E-08	-0.007403	0.001339	0.991112	12:62995340
12:62952910:C:T	0.3539	3.90E-08	-0.007379	0.001342	0.991112	12:62995340
12:62957906:C:G	0.3579	3.25E-08	-0.007389	0.001336	0.973673	12:62995340
12:62960880:A:G	0.3579	2.98E-08	-0.007405	0.001335	0.973673	12:62995340
12:62961299:C:T	0.3519	3.05E-08	-0.007425	0.00134	1	12:62995340

uniqID	*MAF*	*gwasP*	*beta*	*se*	*r2*	*IndSigSNP*
12:62961801:A:G	0.3559	3.42E-08	-0.007377	0.001336	0.982288	12:62995340
12:62964552:C:T	0.3539	3.57E-08	-0.007429	0.001347	0.991112	12:62995340
12:62968280:C:T	0.3539	2.50E-08	-0.007464	0.001338	0.991112	12:62995340
12:62968740:C:CA	0.3539	NA	NA	NA	0.991112	12:62995340
12:62970482:A:G	0.3579	3.63E-08	-0.007376	0.001338	0.973673	12:62995340
12:62972210:G:GT	0.3668	NA	NA	NA	0.66683	12:62995340
12:62975307:C:T	0.3539	3.48E-08	-0.007408	0.001342	0.991112	12:62995340
12:62977162:A:G	0.3549	1,00E-07	0.00813	0.001525	0.986783	12:62995340
12:62977163:A:G	0.3549	2.41E-08	-0.007489	0.001341	0.986783	12:62995340
12:62977175:A:AG	0.3549	NA	NA	NA	0.986783	12:62995340
12:62980580:A:T	0.3579	4.22E-08	-0.007328	0.001336	0.973673	12:62995340
12:62982152:C:CT	0.3141	NA	NA	NA	0.763537	12:62995340
12:62985871:A:C	0.3539	2.59E-08	-0.007461	0.001339	0.991112	12:62995340
12:62986620:A:G	0.3588	1.57E-08	-0.008176	0.001445	0.969344	12:62995340
12:62988288:A:G	0.3519	4.38E-08	-0.007354	0.001342	1	12:62995340
12:62989110:A:C	0.4761	1.50E-08	-0.00726	0.001281	1	12:62989110
12:62990403:A:G	0.4761	1.60E-08	-0.007246	0.001281	1	12:62989110
12:62990415:C:T	0.4761	1.61E-08	-0.007244	0.001281	1	12:62989110
12:62990871:A:C	0.3539	3.56E-08	-0.007389	0.00134	0.991112	12:62995340
12:62992896:G:GA	0.3539	NA	NA	NA	0.991112	12:62995340
12:62993793:A:G	0.3519	3.31E-08	-0.007428	0.001344	1	12:62995340
12:62995269:A:G	0.3539	2.41E-08	-0.007479	0.001339	0.991112	12:62995340
12:62995340:A:G	0.3519	2.23E-08	-0.007497	0.001339	1	12:62995340
12:62995984:C:T	0.3598	1.52E-08	-0.008214	0.00145	0.966673	12:62995340
12:62996061:A:G	0.3519	3.29E-08	-0.007427	0.001343	1	12:62995340
12:62997180:C:T	0.4016	7.64E-07	-0.006461	0.001306	0.798122	12:62995340
12:62999154:C:G	0.3936	2.57E-07	-0.006765	0.001312	0.767469	12:62995340
12:63001068:A:AT	0.3956	NA	NA	NA	0.769246	12:62995340
13:83298475:A:G	0.4235	7.82E-07	-0.006459	0.001307	0.947295	13:83312341
13:83299269:C:T	0.4274	3.93E-07	-0.006623	0.001305	0.962535	13:83312341
13:83300084:A:G	0.3767	NA	NA	NA	0.811883	13:83312341
13:83300138:A:AGAGAGC	0.3936	NA	NA	NA	0.609915	13:83312341

uniqID	*MAF*	*gwasP*	*beta*	*se*	*r2*	*IndSigSNP*
13:83300269:C:T	0.3757	1.94E-07	-0.006959	0.001336	0.837377	13:83312341
13:83300359:A:G	0.3728	9.13E-08	-0.007131	0.001334	0.828615	13:83312341
13:83302594:C:T	0.3728	1.08E-07	-0.007089	0.001333	0.828615	13:83312341
13:83302795:A:T	0.3757	1.12E-07	-0.007075	0.001333	0.837377	13:83312341
13:83304274:C:T	0.3757	1.01E-07	-0.007094	0.001331	0.837377	13:83312341
13:83304898:G:T	0.4205	4.87E-08	-0.007095	0.001299	0.9962	13:83312341
13:83306586:A:G	0.4205	5.07E-08	-0.007087	0.0013	0.9962	13:83312341
13:83311141:A:G	0.4205	3.52E-08	-0.007179	0.001301	0.9962	13:83312341
13:83312341:C:T	0.4215	3.23E-08	-0.007206	0.001303	1	13:83312341
13:83312717:C:G	0.4225	3.74E-08	-0.007177	0.001303	0.973731	13:83312341
16:13021889:C:T	0.2753	2.51E-07	0.0078	0.001511	0.866757	16:13040514
16:13022033:A:AT	0.2753	NA	NA	NA	0.85606	16:13040514
16:13023207:C:T	0.3668	1.05E-07	0.007209	0.001355	0.715225	16:13040514
16:13023388:A:G	0.3658	1.07E-07	0.007205	0.001355	0.710518	16:13040514
16:13023394:A:G	0.2704	2.45E-07	0.007774	0.001505	0.852787	16:13040514
16:13024150:A:G	0.3658	1.12E-07	0.007203	0.001356	0.710518	16:13040514
16:13025315:G:T	0.3658	1.37E-07	0.007146	0.001355	0.710518	16:13040514
16:13026502:A:G	0.2724	2.35E-07	0.007756	0.001499	0.87145	16:13040514
16:13029711:G:GA	0.3131	NA	NA	NA	0.68377	16:13040514
16:13030222:A:G	0.3658	7.24E-08	0.00728	0.001351	0.71673	16:13040514
16:13030875:A:G	0.3658	1.18E-07	0.007142	0.001348	0.71673	16:13040514
16:13031195:A:C	0.3658	1.98E-07	0.007048	0.001354	0.71673	16:13040514
16:13032351:C:G	0.3658	8.65E-08	0.007241	0.001352	0.71673	16:13040514
16:13032547:A:G	0.3658	1.51E-07	0.00708	0.001347	0.71673	16:13040514
16:13032863:A:T	0.3658	8.31E-08	0.007242	0.00135	0.71673	16:13040514
16:13035206:T:TA	0.3648	NA	NA	NA	0.718275	16:13040514
16:13036811:C:T	0.3608	2.30E-07	-0.007613	0.001471	0.731335	16:13040514
16:13037305:C:T	0.3608	3.88E-07	-0.006847	0.001348	0.731335	16:13040514
16:13038054:G:T	0.3608	2.68E-07	-0.006939	0.001348	0.731335	16:13040514
16:13038196:A:G	0.3628	2.36E-07	-0.006961	0.001346	0.726441	16:13040514
16:13038723:G:T	0.2942	8.66E-08	-0.007784	0.001453	0.99501	16:13040514
16:13039154:C:T	0.2942	3.79E-08	-0.008713	0.001583	0.99501	16:13040514

uniqID	*MAF*	*gwasP*	*beta*	*se*	*r2*	*IndSigSNP*
16:13039642:A:T	0.2932	5.41E-08	-0.007918	0.001455	1	16:13040514
16:13039646:C:T	0.2704	1.94E-07	-0.007794	0.001496	0.882397	16:13040514
16:13040514:C:T	0.2932	2.19E-08	-0.008169	0.001459	1	16:13040514
16:13040889:C:G	0.2942	3.86E-08	-0.008004	0.001455	0.99501	16:13040514
16:13041027:G:T	0.2942	3.66E-08	-0.008021	0.001456	0.99501	16:13040514
16:13041921:C:T	0.2714	2.03E-07	-0.007785	0.001497	0.877197	16:13040514
16:13041924:A:G	0.2942	4.44E-08	-0.007968	0.001455	0.99501	16:13040514
16:13041940:A:C	0.2684	1.87E-07	-0.007811	0.001498	0.87328	16:13040514
16:13042097:A:G	0.2942	3.66E-08	-0.00802	0.001456	0.99501	16:13040514
16:13043452:A:G	0.2942	4.41E-08	-0.007971	0.001455	0.99501	16:13040514
16:13044033:C:T	0.2942	5.82E-08	-0.007898	0.001455	0.99501	16:13040514
16:13044827:C:T	0.2833	3.03E-07	-0.007597	0.001482	0.941519	16:13040514
16:13045117:C:CA	0.2833	NA	NA	NA	0.941519	16:13040514
16:13047022:C:G	0.2823	3.86E-07	-0.007566	0.00149	0.93654	16:13040514
16:13047598:C:G	0.2833	3.63E-07	-0.007542	0.001482	0.941519	16:13040514
16:13047734:C:T	0.2833	3.58E-07	-0.007546	0.001481	0.941519	16:13040514
16:13048395:C:T	0.2833	4.42E-07	-0.00749	0.001482	0.941519	16:13040514
16:13048841:C:T	0.2833	4.18E-07	-0.007507	0.001482	0.941519	16:13040514
16:13048916:G:GT	0.2783	NA	NA	NA	0.896961	16:13040514
16:13049490:T:TA	0.2654	NA	NA	NA	0.70988	16:13040514
16:13049557:C:T	0.2803	5.71E-07	-0.007419	0.001483	0.927671	16:13040514
16:13049749:A:C	0.2843	4.18E-07	-0.007506	0.001482	0.936805	16:13040514
16:13049906:A:T	0.2803	6.22E-07	-0.007398	0.001483	0.927671	16:13040514
16:13050340:A:C	0.2833	4.45E-07	-0.007488	0.001482	0.941519	16:13040514
16:13051686:A:C	0.2803	6.66E-07	-0.007378	0.001483	0.927671	16:13040514
16:13052050:A:G	0.2664	4.87E-07	-0.007535	0.001497	0.874302	16:13040514
16:13052714:C:G	0.2664	5.76E-07	-0.007487	0.001497	0.874302	16:13040514
16:13052846:A:G	0.2813	8.16E-07	-0.007315	0.001482	0.932254	16:13040514
16:13053072:A:G	0.2664	5.82E-07	-0.007478	0.001495	0.874302	16:13040514
16:13053136:G:T	0.2803	9.22E-07	-0.007297	0.001486	0.927671	16:13040514
16:13053140:C:G	0.2803	9.15E-07	-0.007299	0.001486	0.927671	16:13040514
16:13053187:C:T	0.2803	5.91E-07	-0.007411	0.001483	0.927671	16:13040514

uniqID	*MAF*	*gwasP*	*beta*	*se*	*r2*	*IndSigSNP*
16:13053270:A:G	0.2803	6.34E-07	-0.007378	0.00148	0.927671	16:13040514
16:13053457:A:AC	0.2803	NA	NA	NA	0.927671	16:13040514
16:13053576:A:T	0.2813	7.04E-07	-0.007348	0.00148	0.922569	16:13040514
16:13053882:A:G	0.2803	3.60E-07	-0.007557	0.001484	0.927671	16:13040514
16:13053924:C:G	0.2803	7.03E-07	-0.007366	0.001484	0.927671	16:13040514
16:13053951:C:T	0.2803	2.76E-07	-0.007646	0.001487	0.927671	16:13040514
16:13054037:C:G	0.2803	7.67E-07	-0.007322	0.00148	0.927671	16:13040514
16:13054265:A:G	0.2803	7.36E-07	-0.007335	0.00148	0.927671	16:13040514
16:13054645:C:T	0.2803	7.46E-07	-0.00733	0.00148	0.927671	16:13040514
16:13054883:C:G	0.2664	4.78E-07	-0.007561	0.001501	0.874302	16:13040514
16:13054897:A:C	0.2803	7.69E-07	-0.007325	0.001481	0.927671	16:13040514
16:13055220:C:T	0.2803	7.58E-07	-0.007326	0.00148	0.927671	16:13040514
16:13055621:T:TCC	0.2664	NA	NA	NA	0.874302	16:13040514
16:13055766:C:T	0.2803	8.17E-07	-0.007303	0.00148	0.927671	16:13040514
16:13057200:A:G	0.2803	8.02E-07	-0.007313	0.001481	0.918013	16:13040514
16:13058241:G:GT	0.3012	NA	NA	NA	0.769003	16:13040514
16:13059539:C:T	0.2803	7.45E-07	-0.007329	0.00148	0.927671	16:13040514
16:13061109:A:G	0.2664	4.68E-07	0.008623	0.00171	0.874302	16:13040514
16:13062232:A:G	0.2803	8.93E-07	-0.007277	0.00148	0.927671	16:13040514
16:13066833:C:T	0.2793	1.98E-07	-0.007585	0.001457	0.91263	16:13040514
16:13070238:A:G	0.2674	1.31E-06	-0.007193	0.001485	0.840116	16:13040514
16:13070671:C:T	0.2674	1.34E-06	-0.007183	0.001485	0.840116	16:13040514
16:13070809:C:G	0.2674	1.35E-06	-0.007181	0.001485	0.840116	16:13040514
16:13075745:A:G	0.2674	9.78E-07	-0.00726	0.001482	0.840116	16:13040514
16:13077351:C:T	0.2674	9.35E-07	-0.007298	0.001487	0.840116	16:13040514
16:13077901:A:G	0.3419	8.17E-07	-0.006759	0.00137	0.775397	16:13040514
16:13077915:A:G	0.2674	2.83E-06	-0.006974	0.001488	0.840116	16:13040514
16:13078044:A:T	0.2674	2.14E-06	-0.007044	0.001485	0.840116	16:13040514
16:13078807:C:T	0.3161	4.07E-06	-0.006399	0.001388	0.681398	16:13040514
16:13079063:C:CTT	0.3419	NA	NA	NA	0.775397	16:13040514
16:13079082:A:C	0.2674	1.34E-06	-0.007232	0.001495	0.840116	16:13040514
16:13079214:A:G	0.3419	1.09E-06	-0.006646	0.001362	0.775397	16:13040514

uniqID	*MAF*	*gwasP*	*beta*	*se*	*r2*	*IndSigSNP*
16:13079389:G:T	0.3161	2.09E-06	-0.006608	0.001392	0.681398	16:13040514
16:13079463:C:T	0.3419	1.14E-06	-0.006635	0.001362	0.775397	16:13040514
16:13079698:A:T	0.3161	4.47E-06	-0.006375	0.001388	0.681398	16:13040514
16:13080252:A:G	0.3161	3.84E-06	-0.006421	0.001389	0.681398	16:13040514
16:13080515:C:T	0.2674	1.80E-06	-0.007092	0.001484	0.840116	16:13040514
16:13081923:C:G	0.2674	1.45E-06	-0.007147	0.001482	0.840116	16:13040514
16:13082445:A:G	0.2674	1.39E-06	-0.007167	0.001484	0.840116	16:13040514
16:13083055:G:T	0.2674	1.37E-06	-0.00717	0.001484	0.840116	16:13040514
16:13083068:G:GT	0.2674	NA	NA	NA	0.840116	16:13040514
16:13086810:C:T	0.2664	1.17E-06	-0.007218	0.001484	0.835952	16:13040514
16:13088905:C:T	0.2932	3.51E-07	-0.007378	0.001447	0.940309	16:13040514
16:13089059:A:G	0.2664	1.29E-06	-0.007187	0.001483	0.835952	16:13040514
16:13089854:G:T	0.2664	1.14E-06	-0.007213	0.001481	0.835952	16:13040514
16:13091332:C:T	0.2664	7.04E-07	-0.007426	0.001496	0.835952	16:13040514
16:13092100:A:G	0.2803	2.24E-06	-0.006954	0.001469	0.888781	16:13040514
16:13092220:C:T	0.2932	3.83E-07	-0.007327	0.001442	0.940309	16:13040514
16:13092663:C:T	0.2803	2.13E-06	-0.006969	0.001469	0.888781	16:13040514
16:13093774:G:T	0.2803	1.59E-06	-0.007073	0.001473	0.888781	16:13040514
16:13093778:C:T	0.2803	2.10E-06	-0.006984	0.001471	0.888781	16:13040514
16:13093858:C:T	0.2803	2.20E-06	-0.006971	0.001471	0.888781	16:13040514
16:13094769:G:T	0.2803	2.53E-06	-0.006931	0.001472	0.888781	16:13040514
16:13094897:C:G	0.2803	3.16E-06	-0.006868	0.001473	0.888781	16:13040514
16:13095142:C:G	0.2803	2.83E-06	-0.006904	0.001473	0.888781	16:13040514
16:13095171:C:T	0.2803	2.81E-06	-0.006906	0.001473	0.888781	16:13040514
16:13095296:A:T	0.2803	2.54E-06	-0.006961	0.001479	0.888781	16:13040514
16:13095739:A:C	0.2803	2.68E-06	-0.006907	0.001471	0.888781	16:13040514
16:13096300:C:T	0.2803	1.25E-06	-0.007156	0.001475	0.888781	16:13040514
16:13097084:G:T	0.2664	1.08E-06	-0.007252	0.001486	0.835952	16:13040514
16:13097125:A:G	0.2932	4.81E-07	-0.00728	0.001445	0.940309	16:13040514
16:13097206:C:G	0.2793	2.57E-07	-0.007516	0.001458	0.88326	16:13040514
16:13097746:G:T	0.3131	NA	NA	NA	0.603152	16:13040514
16:13097749:G:T	0.2326	NA	NA	NA	0.697535	16:13040514

uniqID	*MAF*	*gwasP*	*beta*	*se*	*r2*	*IndSigSNP*
16:13098440:C:G	0.328	4.03E-07	-0.006964	0.001373	0.706808	16:13040514
16:13098508:A:G	0.328	4.50E-07	-0.006936	0.001373	0.706808	16:13040514
16:13098762:A:C	0.3151	2.50E-06	-0.006587	0.001398	0.677243	16:13040514
16:13098934:A:G	0.328	4.91E-07	-0.006913	0.001373	0.706808	16:13040514
16:13099114:C:G	0.2664	1.32E-06	-0.007199	0.001487	0.835952	16:13040514
16:13099177:G:T	0.3419	4.24E-07	-0.006932	0.001369	0.766957	16:13040514
16:13099919:A:G	0.2664	1.25E-06	-0.007216	0.001487	0.835952	16:13040514
16:13099953:G:T	0.2664	1.18E-06	-0.007233	0.001487	0.835952	16:13040514
16:13100021:C:T	0.2664	1.24E-06	-0.007219	0.001487	0.835952	16:13040514
16:13101555:C:T	0.3161	9.22E-06	-0.006237	0.001405	0.852692	16:13040514
16:13101618:A:G	0.2883	3.65E-05	-0.00594	0.001438	0.740125	16:13040514
16:13102532:C:T	0.2793	NA	NA	NA	0.705918	16:13040514
16:13102906:A:T	0.2803	4.72E-05	-0.005879	0.001444	0.711217	16:13040514
16:13104924:C:T	0.2922	3.68E-06	-0.006547	0.001413	0.668497	16:13040514
16:13105091:A:C	0.2932	2.99E-06	-0.006607	0.001413	0.664273	16:13040514
16:13105863:C:T	0.2922	3.25E-06	-0.006583	0.001413	0.660348	16:13040514
16:13105998:C:T	0.2932	3.75E-06	-0.006564	0.001418	0.664273	16:13040514
16:13109594:G:T	0.2932	3.08E-06	-0.006593	0.001412	0.664273	16:13040514
16:13112335:C:T	0.2932	2.42E-06	-0.006661	0.001412	0.664273	16:13040514
16:13115702:C:G	0.2942	4.39E-06	-0.006481	0.00141	0.660073	16:13040514
16:13115755:A:G	0.2942	4.05E-06	-0.006505	0.00141	0.660073	16:13040514
16:13116871:T:TTTTA	0.2942	NA	NA	NA	0.660073	16:13040514
16:13118299:A:T	0.2942	3.70E-06	-0.00656	0.001417	0.660073	16:13040514

Abbreviations: chr = chromosome; pos = pos correlation coefficient; IndSigSNP = rsid of independent significant SNP; dist = distan
across 127 tissue/cell type; commonChrStat

Table S6: Gene mapping FUMA

uniqID	***Genomic Locus***	***nearestGene***	***dist***	***func***	***CADD***
2:98325330:G:T	1	ZAP70	4692	intergenic	2.612
2:98326867:A:G	1	ZAP70	3155	intergenic	10.46
2:98330052:A:C	1	ZAP70	0	UTR5	3.694
2:98334679:C:T	1	ZAP70	0	intronic	1.276
2:98336200:A:C	1	ZAP70	0	intronic	1.961
2:98339513:C:T	1	ZAP70	0	intronic	10.26
2:98342323:A:C	1	ZAP70	0	intronic	1.587
2:98343258:A:G	1	ZAP70	0	intronic	0.578
2:98345086:G:T	1	ZAP70	0	intronic	4.315
2:98346461:A:G	1	ZAP70	0	intronic	0.589
2:98351654:T:TG	1	ZAP70	0	intronic	6.648
2:98351986:C:T	1	ZAP70	0	intronic	2.517
2:98353847:C:T	1	ZAP70	0	intronic	0.322
2:98354139:G:T	1	ZAP70	0	intronic	2.278
2:98354511:A:G	1	ZAP70	0	exonic	10.58
2:98355990:A:G	1	ZAP70	0	UTR3	0.484
2:98356846:A:G	1	ZAP70	520	downstream	3.061
2:98357163:C:T	1	ZAP70	837	downstream	0.591
2:98360443:A:G	1	ZAP70	4117	intergenic	2.059
2:98361679:C:G	1	ZAP70	5353	intergenic	0.189
2:98363313:A:T	1	ZAP70	6987	intergenic	0.132
2:98365164:C:G	1	TMEM131	7634	intergenic	0.757
2:98368551:A:ACT	1	TMEM131	4247	intergenic	1.011
2:98370698:A:T	1	TMEM131	2100	intergenic	1.091
2:98374567:C:T	1	TMEM131	0	intronic	1.088
2:98377512:A:T	1	TMEM131	0	intronic	1.357
2:98379267:C:T	1	TMEM131	0	intronic	3.889
2:98379813:A:G	1	TMEM131	0	intronic	5.987
2:98382886:A:G	1	TMEM131	0	intronic	5.539
2:98384528:A:ATTTTC	1	TMEM131	0	intronic	1.9
2:98386731:C:T	1	TMEM131	0	intronic	0.083

uniqID	*Genomic Locus*	*nearestGene*	*dist*	*func*	*CADD*
2:98390117:G:GT	1	TMEM131	0	intronic	1.21
2:98393231:C:T	1	TMEM131	0	intronic	0.26
2:98393894:C:T	1	TMEM131	0	intronic	0.124
2:98395653:A:C	1	TMEM131	0	intronic	1.114
2:98397301:C:T	1	TMEM131	0	intronic	6.049
2:98402753:A:T	1	TMEM131	0	intronic	3.156
2:98402772:C:G	1	TMEM131	0	intronic	1.59
2:98405034:C:T	1	TMEM131	0	intronic	0.387
2:98405695:C:T	1	TMEM131	0	intronic	0.348
2:98405929:A:G	1	TMEM131	0	intronic	5.081
2:98407350:C:T	1	TMEM131	0	intronic	0.663
2:98409046:A:G	1	TMEM131	0	exonic	18.87
2:98409565:C:T	1	TMEM131	0	intronic	0.091
2:98410769:G:T	1	TMEM131	0	intronic	2.088
2:98413781:C:T	1	TMEM131	0	intronic	0.711
2:98416848:A:C	1	TMEM131	0	intronic	3.475
2:98416850:G:T	1	TMEM131	0	intronic	6.209
2:98417089:C:T	1	TMEM131	0	intronic	1.456
2:98419726:A:T	1	TMEM131	0	intronic	5.647
2:98420142:C:G	1	TMEM131	0	intronic	5.067
2:98420431:C:T	1	TMEM131	0	intronic	8.313
2:98421364:A:G	1	TMEM131	0	intronic	4.476
2:98424802:A:G	1	TMEM131	0	intronic	3.82
2:98427382:C:T	1	TMEM131	0	intronic	10.38
2:98434931:G:GGGGGC	1	TMEM131	0	intronic	0.409
2:98435982:C:T	1	TMEM131	0	intronic	7.27
2:98440234:C:T	1	TMEM131	0	intronic	2.827
2:98442202:A:G	1	TMEM131	0	intronic	3.931
2:98443039:C:G	1	TMEM131	0	intronic	1.206
2:98443081:G:GA	1	TMEM131	0	intronic	0.909
2:98443658:C:T	1	TMEM131	0	intronic	5.605
2:98447004:C:T	1	TMEM131	0	intronic	4.929

uniqID	Genomic Locus	nearestGene	dist	func	CADD
2:98448395:G:T	1	TMEM131	0	intronic	9.048
2:98449024:A:G	1	TMEM131	0	intronic	1.746
2:98454472:A:G	1	TMEM131	0	intronic	2.226
2:98454473:C:T	1	TMEM131	0	intronic	2.151
2:98454572:C:G	1	TMEM131	0	intronic	1.995
2:98454931:A:G	1	TMEM131	0	intronic	0.645
2:98455152:C:T	1	TMEM131	0	intronic	2.056
2:98455510:C:G	1	TMEM131	0	intronic	1.062
2:98461821:C:T	1	TMEM131	0	intronic	0.604
2:98461953:C:T	1	TMEM131	0	intronic	2.06
2:98466647:C:T	1	TMEM131	0	intronic	2.087
2:98468892:A:AC	1	TMEM131	0	intronic	1.118
2:98472610:C:T	1	TMEM131	0	intronic	6.196
2:98479581:A:C	1	TMEM131	0	intronic	3.39
2:98488949:C:G	1	TMEM131	0	intronic	0.16
2:98491750:C:T	1	TMEM131	0	intronic	0.197
2:98493359:A:G	1	TMEM131	0	intronic	2.663
2:98495661:G:T	1	TMEM131	0	intronic	6.015
2:98501884:C:T	1	TMEM131	0	intronic	0.77
2:98502987:C:T	1	TMEM131	0	intronic	4.338
2:98506011:T:TATA	1	TMEM131	0	intronic	7.701
2:98506910:C:T	1	TMEM131	0	intronic	0.597
2:98507972:C:CA	1	TMEM131	0	intronic	7.161
2:98510166:G:T	1	TMEM131	0	intronic	1.728
2:98517626:A:G	1	TMEM131	0	intronic	1.939
2:98519014:C:T	1	TMEM131	0	intronic	3.591
2:98520564:C:T	1	TMEM131	0	intronic	7.871
2:98521823:A:C	1	TMEM131	0	intronic	11.46
2:98526685:A:AAAAT	1	TMEM131	0	intronic	1.979
2:98528689:A:G	1	TMEM131	0	intronic	1.128
2:98534412:C:T	1	TMEM131	0	intronic	0.101
2:98534531:C:T	1	TMEM131	0	intronic	8.039

uniqID	Genomic Locus	nearestGene	dist	func	CADD
2:98539588:A:C	1	TMEM131	0	intronic	1.541
2:98541001:C:T	1	TMEM131	0	intronic	5.185
2:98547069:A:AT	1	TMEM131	0	intronic	0.185
2:98548604:T:TA	1	TMEM131	0	intronic	6.515
2:98551456:G:GA	1	TMEM131	0	intronic	0.585
2:98552271:C:G	1	TMEM131	0	intronic	11.3
2:98552299:C:G	1	TMEM131	0	intronic	0.448
2:98554946:A:C	1	TMEM131	0	intronic	1.708
2:98557575:G:T	1	TMEM131	0	intronic	1.301
2:98561153:A:C	1	TMEM131	0	intronic	7.356
2:98565115:C:CT	1	TMEM131	0	intronic	1.425
2:98565144:A:G	1	TMEM131	0	intronic	2.208
2:98565400:A:G	1	TMEM131	0	intronic	3.026
2:98567820:A:G	1	TMEM131	0	intronic	4.684
2:98570999:A:G	1	TMEM131	0	intronic	3.673
2:98571084:A:G	1	TMEM131	0	intronic	1.749
2:98580012:C:T	1	TMEM131	0	intronic	3.615
2:98580724:G:T	1	TMEM131	0	intronic	0.478
2:98587288:A:G	1	TMEM131	0	intronic	8.473
2:98588372:A:C	1	TMEM131	0	intronic	0.983
2:98602165:A:G	1	TMEM131	0	intronic	0.322
2:98604570:G:GACAC	1	TMEM131	0	intronic	7.818
2:98605982:A:G	1	TMEM131	0	intronic	5.225
2:98606770:C:T	1	TMEM131	0	intronic	1.129
2:98612260:C:T	1	TMEM131	0	UTR5	14.06
2:98616519:A:ATT	1	TMEM131	4130	intergenic	3.339
2:98621058:A:AG	1	TMEM131	8669	intergenic	0.525
2:98621060:A:T	1	TMEM131	8671	intergenic	0.848
2:98623227:A:G	1	TMEM131	10838	intergenic	0.256
2:98623406:A:G	1	TMEM131	11017	intergenic	0.347
8:8524474:C:G	2	AC087269.2	33415	intergenic	0.307
8:8543324:C:T	2	CLDN23	16123	intergenic	3.038

uniqID	*Genomic Locus*	*nearestGene*	*dist*	*func*	*CADD*
8:8544808:C:T	2	CLDN23	14639	intergenic	2.513
8:8544872:A:G	2	CLDN23	14575	intergenic	5.216
8:8545624:T:TTTG	2	CLDN23	13823	intergenic	1.643
8:8546283:A:AAATT	2	CLDN23	13164	intergenic	3.898
8:8547110:C:G	2	CLDN23	12337	intergenic	1.581
8:8547313:A:C	2	CLDN23	12134	intergenic	0.412
8:8547811:A:G	2	CLDN23	11636	intergenic	0.532
8:8548117:A:T	2	CLDN23	11330	intergenic	0.38
8:8548801:A:G	2	CLDN23	10646	intergenic	0.129
8:8549020:A:C	2	CLDN23	10427	intergenic	0.637
8:8549432:A:G	2	CLDN23	10015	intergenic	1.549
8:8578067:C:T	2	RP11-211C9.1	3135	intergenic	2.144
8:8578120:A:T	2	RP11-211C9.1	3082	intergenic	1.801
8:8578229:A:G	2	RP11-211C9.1	2973	intergenic	0.116
8:8578794:A:T	2	RP11-211C9.1	2408	intergenic	2.053
8:8578811:C:T	2	RP11-211C9.1	2391	intergenic	6.568
8:8581408:C:G	2	RP11-211C9.1	0	ncRNA_intronic	2.184
8:8582155:A:AAAAAGAAAAG	2	RP11-211C9.1	0	ncRNA_intronic	0.435
8:8582155:AAAAAG:AAAAAGAAAAG	2	RP11-211C9.1	0	NA	NA
8:8583872:C:T	2	RP11-211C9.1	0	ncRNA_intronic	2.972
8:8584344:A:G	2	RP11-211C9.1	0	ncRNA_intronic	2.425
8:8587571:G:T	2	RP11-211C9.1	0	ncRNA_intronic	0.377
8:8589117:C:T	2	RP11-211C9.1	0	ncRNA_intronic	5.356
8:8592845:C:G	2	RP11-211C9.1	0	ncRNA_intronic	3.765
8:8595104:A:T	2	RP11-211C9.1	0	ncRNA_intronic	3.341
8:8595838:G:T	2	RP11-211C9.1	0	ncRNA_intronic	3.232
8:8596731:G:T	2	RP11-211C9.1	0	ncRNA_intronic	1.585
8:8598388:A:T	2	RP11-211C9.1	0	ncRNA_intronic	4.009
8:8602344:C:T	2	RP11-211C9.1	0	ncRNA_intronic	6.657
8:8603160:A:G	2	RP11-211C9.1	0	ncRNA_intronic	7.021
8:8633548:G:T	2	RP11-211C9.1	0	ncRNA_intronic	5.665
8:8637429:A:G	2	RP11-211C9.1	0	ncRNA_intronic	1.071

uniqID	*Genomic Locus*	*nearestGene*	*dist*	*func*	*CADD*
8:8639740:A:G	2	RP11-211C9.1	0	ncRNA_exonic	0.189
8:8640172:A:C	2	RP11-211C9.1	182	upstream	0.231
8:8641145:C:T	2	MFHAS1	0	UTR3	16.03
8:8643938:C:T	2	MFHAS1	0	intronic	1.258
8:8644213:G:GT	2	MFHAS1	0	intronic	0.986
8:8644213:G:GTT	2	MFHAS1	0	NA	0.961
8:8644274:C:G	2	MFHAS1	0	intronic	0.288
8:8644595:A:AT	2	MFHAS1	0	intronic	0.622
8:8646246:C:T	2	MFHAS1	0	intronic	0.006
8:8649881:C:T	2	MFHAS1	0	intronic	3.127
8:8652889:A:G	2	MFHAS1	0	intronic	2.542
8:8654057:A:G	2	MFHAS1	0	intronic	2.046
8:8654527:C:T	2	MFHAS1	0	intronic	2.606
8:8658540:A:G	2	MFHAS1	0	intronic	4.468
8:8660538:A:C	2	MFHAS1	0	intronic	0.886
8:8661026:C:CA	2	MFHAS1	0	intronic	1.165
8:8661114:C:CA	2	MFHAS1	0	intronic	1.142
8:8661534:C:T	2	MFHAS1	0	intronic	5.035
8:8661681:C:G	2	MFHAS1	0	intronic	0.053
8:8663215:C:T	2	MFHAS1	0	intronic	1.424
8:8664622:A:G	2	MFHAS1	0	intronic	0.544
8:8664940:A:G	2	MFHAS1	0	intronic	6.776
8:8665147:A:G	2	MFHAS1	0	intronic	1.321
8:8665802:C:T	2	MFHAS1	0	intronic	3.787
8:8666916:C:T	2	MFHAS1	0	intronic	1.862
8:8667444:C:T	2	MFHAS1	0	intronic	3.913
8:8668486:A:G	2	MFHAS1	0	intronic	4.816
8:8668917:A:C	2	MFHAS1	0	intronic	1.227
8:8669681:C:CGTAA	2	MFHAS1	0	intronic	3.15
8:8669681:C:T	2	MFHAS1	0	NA	0.033
8:8670082:C:G	2	MFHAS1	0	intronic	7.687
8:8670177:A:T	2	MFHAS1	0	intronic	3.257

uniqID	Genomic Locus	nearestGene	dist	func	CADD
8:8670322:C:CT	2	MFHAS1	0	intronic	0.841
8:8670599:A:G	2	MFHAS1	0	intronic	4.763
8:8670736:A:C	2	MFHAS1	0	intronic	2.53
8:8671962:C:T	2	MFHAS1	0	intronic	2.474
8:8672429:C:G	2	MFHAS1	0	intronic	1.464
8:8672579:A:G	2	MFHAS1	0	intronic	3.217
8:8672952:A:C	2	MFHAS1	0	intronic	3.414
8:8673320:C:T	2	MFHAS1	0	intronic	2.843
8:8673601:A:C	2	MFHAS1	0	intronic	3.727
8:8673736:C:T	2	MFHAS1	0	intronic	2.268
8:8675176:A:G	2	MFHAS1	0	intronic	0.987
8:8675325:A:T	2	MFHAS1	0	intronic	6.869
8:8676626:G:GAATC	2	MFHAS1	0	intronic	2.03
8:8678530:A:G	2	MFHAS1	0	intronic	5.451
8:8679141:C:CTT	2	MFHAS1	0	intronic	1.974
8:8679176:A:G	2	MFHAS1	0	intronic	10.14
8:8679614:C:T	2	MFHAS1	0	intronic	0.034
8:8680477:A:G	2	MFHAS1	0	intronic	3.841
8:8682192:A:T	2	MFHAS1	0	intronic	5.001
8:8682878:C:T	2	MFHAS1	0	intronic	3.001
8:8684953:A:G	2	MFHAS1	0	intronic	1.584
8:8685190:A:G	2	MFHAS1	0	intronic	5.143
8:8685646:C:T	2	MFHAS1	0	intronic	1.281
8:8685854:A:G	2	MFHAS1	0	intronic	1.488
8:8687054:C:G	2	MFHAS1	0	intronic	0.078
8:8687325:A:G	2	MFHAS1	0	intronic	0.63
8:8690787:C:T	2	MFHAS1	0	intronic	2.52
8:8691622:A:T	2	MFHAS1	0	intronic	2.747
8:8692477:C:T	2	MFHAS1	0	intronic	4.006
8:8696449:G:T	2	MFHAS1	0	intronic	0.068
8:8698977:C:CA	2	MFHAS1	0	intronic	1.834
8:8699757:A:T	2	MFHAS1	0	intronic	0.057

uniqID	*Genomic Locus*	*nearestGene*	*dist*	*func*	*CADD*
8:8699761:C:T	2	MFHAS1	0	intronic	0.311
8:8700851:C:T	2	MFHAS1	0	intronic	1.177
8:8702607:C:G	2	MFHAS1	0	intronic	0.87
8:8702827:A:G	2	MFHAS1	0	intronic	2.907
8:8702875:C:T	2	MFHAS1	0	intronic	0.501
8:8703781:G:GGA	2	MFHAS1	0	intronic	0.094
8:8704330:C:G	2	MFHAS1	0	intronic	2.414
8:8706130:A:AT	2	MFHAS1	0	intronic	1.522
8:8706209:A:C	2	MFHAS1	0	intronic	5.242
8:8706332:A:C	2	MFHAS1	0	intronic	3.613
8:8707197:C:G	2	MFHAS1	0	intronic	11.1
8:8708974:C:G	2	MFHAS1	0	intronic	1.073
8:8709756:C:G	2	MFHAS1	0	intronic	1.515
8:8709971:C:T	2	MFHAS1	0	intronic	1.462
8:8713038:C:T	2	MFHAS1	0	intronic	0.806
8:8718850:A:G	2	MFHAS1	0	intronic	0.563
8:8719513:A:G	2	MFHAS1	0	intronic	0.455
8:8721473:A:G	2	MFHAS1	0	intronic	4.862
8:8722675:C:T	2	MFHAS1	0	intronic	0.77
8:8723651:C:G	2	MFHAS1	0	intronic	0.712
8:8724257:C:T	2	MFHAS1	0	intronic	0.004
8:8724276:C:T	2	MFHAS1	0	intronic	4.798
8:8724415:C:T	2	MFHAS1	0	intronic	0.266
8:8725126:G:T	2	MFHAS1	0	intronic	0.21
8:8725229:A:G	2	MFHAS1	0	intronic	0.663
8:8725319:A:G	2	MFHAS1	0	intronic	0.824
8:8726804:G:T	2	MFHAS1	0	intronic	6.512
8:8729761:C:G	2	MFHAS1	0	intronic	15.49
8:8730488:A:G	2	MFHAS1	0	intronic	1.773
8:8768895:A:G	2	RNU6-682P	15466	intergenic	1.316
8:8769708:C:T	2	RNU6-682P	16279	intergenic	1.818
8:8770512:C:T	2	RNU6-682P	17083	intergenic	0.105

uniqID	*Genomic Locus*	*nearestGene*	*dist*	*func*	*CADD*
8:8824858:G:T	2	RP11-62H7.2	0	ncRNA_intronic	2.74
8:9394053:G:T	2	RP11-375N15.2	18602	intergenic	1.673
8:9489417:A:G	2	TNKS	0	intronic	1.908
8:9492426:G:T	2	TNKS	0	intronic	6.813
8:9492453:C:T	2	TNKS	0	intronic	0.86
8:9494732:G:T	2	TNKS	0	intronic	1.74
8:9496118:A:T	2	TNKS	0	intronic	8.138
8:9525325:C:T	2	TNKS	0	intronic	2.067
8:9527707:A:G	2	TNKS	0	intronic	0.807
8:9527863:A:T	2	TNKS	0	intronic	5.511
8:9527869:A:G	2	TNKS	0	intronic	2.726
8:9568369:A:G	2	TNKS	0	intronic	2.31
8:9569104:C:T	2	TNKS	0	intronic	2.099
8:9569109:C:T	2	TNKS	0	intronic	2.722
8:9572099:A:G	2	TNKS	0	intronic	5.956
8:9574830:G:T	2	TNKS	0	intronic	6.472
8:9575445:A:G	2	TNKS	0	intronic	1.93
8:9579144:C:CT	2	TNKS	0	intronic	0.957
8:9579318:C:T	2	TNKS	0	intronic	3.74
8:9579377:C:CA	2	TNKS	0	intronic	2.513
8:9583872:C:T	2	TNKS	0	intronic	9.427
8:9584598:C:G	2	TNKS	0	intronic	0.539
8:9586062:A:T	2	TNKS	0	intronic	2.722
8:9593309:A:C	2	TNKS	0	intronic	2.031
8:10121635:A:G	3	MSRA	0	intronic	0.878
8:10122423:A:G	3	MSRA	0	intronic	0.238
8:10146490:C:CT	3	MSRA	0	intronic	1.365
8:10147398:C:T	3	MSRA	0	intronic	1.289
8:10148447:A:G	3	MSRA	0	intronic	6.046
8:10149212:A:C	3	MSRA	0	intronic	12.79
8:10150070:C:G	3	MSRA	0	intronic	1.293
8:10176506:A:T	3	MSRA	0	intronic	0.045

uniqID	Genomic Locus	nearestGene	dist	func	CADD
8:10758213:A:G	3	XKR6	0	intronic	0.556
8:10810451:A:G	3	XKR6	0	intronic	3.584
8:10811829:C:T	3	XKR6	0	intronic	0.783
8:10812333:G:GT	3	XKR6	0	intronic	0.761
8:10813197:C:T	3	XKR6	0	intronic	2.464
8:10813904:G:T	3	XKR6	0	intronic	3.109
8:10815754:C:G	3	XKR6	0	intronic	14.04
8:10816772:A:G	3	XKR6	0	intronic	3.992
8:10817197:C:T	3	XKR6	0	intronic	1.116
8:10818607:A:G	3	XKR6	0	intronic	4.565
8:10818657:A:G	3	XKR6	0	intronic	3.434
8:10819854:C:T	3	XKR6	0	intronic	2.275
8:10821056:C:CT	3	XKR6	0	intronic	1.424
8:10828909:C:T	3	XKR6	0	intronic	2.26
8:10831868:G:T	3	XKR6	0	intronic	0.088
8:10835480:C:T	3	XKR6	0	intronic	1.023
8:10835917:C:T	3	XKR6	0	intronic	5.889
8:10836024:C:T	3	XKR6	0	intronic	7.428
8:10836069:C:T	3	XKR6	0	intronic	1.915
8:10836359:C:T	3	XKR6	0	intronic	0.384
8:10836436:C:T	3	XKR6	0	intronic	2.966
8:10836463:C:G	3	XKR6	0	intronic	3.938
8:10836508:A:G	3	XKR6	0	intronic	4.494
8:10837019:A:G	3	XKR6	0	intronic	2.3
8:10837190:A:G	3	XKR6	0	intronic	8.094
8:10837414:A:C	3	XKR6	0	intronic	1.612
8:10837420:A:G	3	XKR6	0	intronic	5.108
8:10837568:C:T	3	XKR6	0	intronic	1.276
8:10837569:C:G	3	XKR6	0	intronic	0.182
8:10839803:C:T	3	XKR6	0	intronic	1.947
8:10841858:A:G	3	XKR6	0	intronic	3.723
8:10842659:G:T	3	XKR6	0	intronic	0.558

uniqID	*Genomic Locus*	*nearestGene*	*dist*	*func*	*CADD*
8:10903475:A:T	3	XKR6	0	intronic	4.33
8:10909193:A:C	3	XKR6	0	intronic	0.513
8:10909936:C:T	3	XKR6	0	intronic	0.715
8:10910066:C:T	3	XKR6	0	intronic	5.728
8:10910343:C:G	3	XKR6	0	intronic	0.481
8:10926892:A:C	3	XKR6	0	intronic	6.456
8:10927234:C:CAGTA	3	XKR6	0	intronic	0.504
8:10930069:A:C	3	XKR6	0	intronic	1.146
8:10932203:C:G	3	XKR6	0	intronic	1.779
8:10932695:C:G	3	XKR6	0	intronic	0.134
8:10932868:A:G	3	XKR6	0	intronic	0.127
8:10933699:A:T	3	XKR6	0	intronic	4.451
8:10935082:C:T	3	XKR6	0	intronic	0.716
8:10935366:C:T	3	XKR6	0	intronic	3.97
8:10935368:A:C	3	XKR6	0	intronic	1.477
8:10935898:A:G	3	XKR6	0	intronic	0.29
8:10936811:A:G	3	XKR6	0	intronic	7.974
8:10936891:C:T	3	XKR6	0	intronic	13.99
8:10938260:A:G	3	XKR6	0	intronic	4.101
8:10939273:G:T	3	XKR6	0	intronic	5.647
8:10939490:G:T	3	XKR6	0	intronic	1.783
8:10943276:C:T	3	XKR6	0	intronic	0.551
8:10944809:G:T	3	XKR6	0	intronic	1.283
8:10945439:A:G	3	XKR6	0	intronic	6.536
8:10945767:C:CA	3	XKR6	0	intronic	0.627
8:10948422:C:CA	3	XKR6	0	intronic	0.578
8:10948968:A:G	3	XKR6	0	intronic	0.514
8:10950396:C:G	3	XKR6	0	intronic	2.2
8:10950757:C:G	3	XKR6	0	intronic	0.015
8:10950866:C:T	3	XKR6	0	intronic	3.266
8:10951175:G:GT	3	XKR6	0	intronic	0.28
8:10952500:A:T	3	XKR6	0	intronic	0.494

uniqID	Genomic Locus	nearestGene	dist	func	CADD
8:10953092:C:T	3	XKR6	0	intronic	0.934
8:10953874:A:G	3	XKR6	0	intronic	3.045
8:10955225:C:G	3	XKR6	0	intronic	0.174
8:10955383:G:GTT	3	XKR6	0	intronic	0.948
8:10955383:GT:GTT	3	XKR6	0	NA	NA
8:10957243:A:G	3	XKR6	0	intronic	0.551
8:10958824:C:T	3	XKR6	0	intronic	5.397
8:10960572:C:T	3	XKR6	0	intronic	0.232
8:10961433:C:T	3	XKR6	0	intronic	1.648
8:10962099:A:T	3	XKR6	0	intronic	5.806
8:10962800:C:T	3	XKR6:AF131215.9	00:00	ncRNA_exonic	1.253
8:10962929:C:G	3	XKR6:AF131215.9	00:00	ncRNA_exonic	2.251
8:10963288:C:G	3	XKR6:AF131215.9	00:00	ncRNA_exonic	1.422
8:10964906:C:T	3	XKR6	0	intronic	1.332
8:10964969:A:T	3	XKR6	0	intronic	3.513
8:10968550:C:G	3	XKR6	0	intronic	0.6
8:10968926:A:G	3	XKR6	0	intronic	2.187
8:10969075:C:T	3	XKR6	0	intronic	0.076
8:10970773:A:T	3	XKR6	0	intronic	2.962
8:10973149:C:CAA	3	XKR6	0	intronic	3.379
8:10973149:C:CA	3	XKR6	0	NA	3.444
8:10974917:C:T	3	XKR6	0	intronic	0.214
8:10975081:A:T	3	XKR6	0	intronic	3.799
8:10975629:A:C	3	XKR6	0	intronic	0.511
8:10975682:C:G	3	XKR6	0	intronic	0.667
8:10975733:A:G	3	XKR6	0	intronic	0.367
8:10976494:C:G	3	XKR6	0	intronic	0.565
8:10976569:A:G	3	XKR6	0	intronic	4.532
8:10976571:G:T	3	XKR6	0	intronic	0.657
8:10978065:G:T	3	XKR6	0	intronic	1.49
8:10979561:C:G	3	XKR6	0	intronic	0.327
8:10979821:A:G	3	XKR6	0	intronic	0.52

uniqID	Genomic Locus	nearestGene	dist	func	CADD
8:10981003:A:C	3	XKR6:AF131215.3	00:00	ncRNA_exonic	0.53
8:10982051:A:G	3	XKR6:AF131215.3	00:00	ncRNA_exonic	4.462
8:10982410:C:G	3	XKR6:AF131215.3	00:00	ncRNA_intronic	0.649
8:10983534:A:G	3	XKR6:AF131215.3	00:00	ncRNA_exonic	0.551
8:10983579:G:T	3	XKR6	0	intronic	0.982
8:10983921:C:G	3	XKR6	0	intronic	0.089
8:10985140:A:G	3	XKR6:AF131215.5	00:00	intronic	1.52
8:10985432:C:T	3	XKR6:AF131215.5	00:00	intronic	0.937
8:10986837:G:T	3	XKR6:AF131215.5	00:00	UTR5	4.475
8:10986859:C:T	3	XKR6:AF131215.5	00:00	UTR5	0.648
8:10987199:C:T	3	XKR6:AF131215.5	00:00	UTR5	9.773
8:10987553:C:T	3	XKR6:AF131215.5	00:00	UTR5	9.295
8:10987651:G:T	3	XKR6:AF131215.5	00:00	UTR5	9.078
8:10987967:A:G	3	XKR6	0	intronic	0.223
8:10988138:A:C	3	XKR6	0	intronic	5.227
8:10988275:A:G	3	XKR6	0	intronic	0.395
8:10989057:A:G	3	XKR6	0	intronic	0.796
8:10989206:C:T	3	XKR6	0	intronic	0.034
8:10989521:A:G	3	XKR6	0	intronic	1.299
8:10990164:A:G	3	XKR6	0	intronic	0.827
8:10990371:G:GA	3	XKR6	0	intronic	1.821
8:10990672:C:T	3	XKR6	0	intronic	10.12
8:10992252:C:G	3	XKR6	0	intronic	0.791
8:10992544:A:AT	3	XKR6	0	intronic	0.275
8:10992605:A:C	3	XKR6	0	intronic	0.079
8:10992883:C:T	3	XKR6	0	intronic	2.877
8:10993904:C:T	3	XKR6	0	intronic	1.881
8:10993995:C:T	3	XKR6	0	intronic	1.127
8:10994743:C:G	3	XKR6:AF131215.4	00:00	ncRNA_exonic	3.883
8:10996089:A:G	3	XKR6:AF131215.4	00:00	ncRNA_exonic	1.477
8:11010974:G:T	3	XKR6	0	intronic	7.035
8:11012977:C:T	3	XKR6	0	intronic	0.68

uniqID	Genomic Locus	nearestGene	dist	func	CADD
8:11013025:A:C	3	XKR6	0	intronic	3.158
8:11014616:G:T	3	XKR6	0	intronic	0.896
8:11015338:C:T	3	XKR6	0	intronic	5.978
8:11016889:A:G	3	XKR6	0	intronic	9.357
8:11019578:C:G	3	XKR6	0	intronic	12.47
8:11020313:G:T	3	XKR6	0	intronic	2.826
8:11021682:A:G	3	XKR6	0	intronic	8.265
8:11022106:C:G	3	XKR6	0	intronic	4.349
8:11022185:C:T	3	XKR6	0	intronic	0.972
8:11023655:C:T	3	XKR6	0	intronic	0.225
8:11023997:C:G	3	XKR6	0	intronic	0.096
8:11024275:C:T	3	XKR6	0	intronic	3.118
8:11024326:A:C	3	XKR6	0	intronic	2.053
8:11024663:A:C	3	XKR6	0	intronic	3.013
8:11027491:T:TAA	3	XKR6	0	intronic	0.751
8:11029029:G:T	3	XKR6	0	intronic	1.178
8:11029039:G:T	3	XKR6	0	intronic	0.176
8:11030892:A:G	3	XKR6	0	intronic	1.376
8:11030935:C:T	3	XKR6	0	intronic	1.584
8:11031472:C:T	3	XKR6	0	intronic	0.606
8:11032228:G:T	3	XKR6	0	intronic	7.268
8:11032240:C:T	3	XKR6	0	intronic	1.397
8:11033517:C:G	3	XKR6	0	intronic	1.862
8:11033525:C:T	3	XKR6	0	intronic	6.262
8:11033737:A:G	3	XKR6	0	intronic	1.049
8:11033976:C:T	3	XKR6	0	intronic	8.343
8:11034028:A:G	3	XKR6	0	intronic	7.031
8:11034859:A:G	3	XKR6	0	intronic	2.613
8:11035071:G:GAGGTCATAATGGAAT	3	XKR6	0	intronic	6.345
8:11036052:T:TAA	3	XKR6	0	intronic	0.389
8:11036799:C:G	3	XKR6	0	intronic	1.041
8:11036843:C:T	3	XKR6	0	intronic	5.53

uniqID	*Genomic Locus*	*nearestGene*	*dist*	*func*	*CADD*
8:11036919:A:G	3	XKR6	0	intronic	11.72
8:11037034:A:G	3	XKR6	0	intronic	0.081
8:11037187:C:T	3	XKR6	0	intronic	1.406
8:11037903:G:T	3	XKR6	0	intronic	1.457
8:11038244:A:T	3	XKR6	0	intronic	1.995
8:11038885:A:T	3	XKR6	0	intronic	1.307
8:11039159:C:G	3	XKR6	0	intronic	3.977
8:11039816:G:T	3	XKR6	0	intronic	1.235
8:11040216:C:T	3	XKR6	0	intronic	0.027
8:11040647:A:G	3	XKR6	0	intronic	1.146
8:11041642:A:T	3	XKR6	0	intronic	10.77
8:11041661:C:T	3	XKR6	0	intronic	11.96
8:11041897:T:TACACACACACACAC	3	XKR6	0	intronic	0.421
8:11042974:C:G	3	XKR6	0	intronic	0.038
8:11042974:G:T	3	XKR6	0	NA	0.036
8:11043138:C:T	3	XKR6	0	intronic	3.122
8:11043236:C:T	3	XKR6	0	intronic	0.426
8:11043926:C:T	3	XKR6	0	intronic	2.035
8:11044689:A:G	3	XKR6	0	intronic	7.705
8:11045161:A:G	3	XKR6	0	intronic	1.653
8:11046209:C:T	3	XKR6	0	intronic	6.142
8:11046394:C:G	3	XKR6	0	intronic	3.587
8:11053922:A:G	3	XKR6	0	intronic	0.938
8:11054097:A:C	3	XKR6	0	intronic	1.367
8:11055597:A:C	3	XKR6	0	intronic	0.225
8:11056175:A:G	3	XKR6	0	intronic	5.555
8:11056388:C:T	3	XKR6	0	intronic	3.66
8:11060217:T:TA	3	AF131215.8	256	upstream	2.243
8:11060311:A:C	3	AF131215.8	162	upstream	6.184
8:11061792:C:T	3	AF131215.8	611	downstream	6.566
8:11062882:C:T	3	AF131215.8	1701	intergenic	4.088
8:11065003:A:C	3	AF131215.8	3822	intergenic	0.106

uniqID	*Genomic Locus*	*nearestGene*	*dist*	*func*	*CADD*
8:11069960:C:T	3	AF131215.8	8779	intergenic	5.453
8:11070360:C:G	3	AF131215.8	9179	intergenic	0.57
8:11071057:A:G	3	AF131215.8	9876	intergenic	0.822
8:11072020:A:G	3	AF131215.8	10839	intergenic	1.899
8:11073402:A:G	3	AF131215.8	12221	intergenic	1.258
8:11073578:C:T	3	AF131215.8	12397	intergenic	0.008
8:11074036:A:G	3	AF131215.8	12855	intergenic	1.458
8:11074365:C:CTA	3	AF131215.8	13184	intergenic	0.606
8:11074812:C:T	3	AF131215.8	13631	intergenic	1.156
8:11076635:G:T	3	AF131215.8	15454	intergenic	1.543
8:11078781:A:C	3	AF131215.8	17600	intergenic	1.136
8:11078949:A:G	3	AF131215.8	17768	intergenic	2.448
8:11079367:A:G	3	AF131215.8	18186	intergenic	7.258
8:11080014:C:T	3	AF131215.8	18833	intergenic	0.214
8:11080665:A:T	3	AF131215.8	19484	intergenic	1.242
8:11080675:C:T	3	AF131215.8	19494	intergenic	0.164
8:11086942:C:T	3	LINC00529	18192	intergenic	1.004
8:11087475:A:G	3	LINC00529	17659	intergenic	1.164
8:11098992:A:G	3	LINC00529	6142	intergenic	2.279
8:11098996:C:T	3	LINC00529	6138	intergenic	1.865
8:11109269:C:T	3	LINC00529	0	ncRNA_intronic	5.097
8:11109303:C:G	3	LINC00529	0	ncRNA_intronic	0.469
8:11111462:A:T	3	LINC00529	0	ncRNA_intronic	1.428
8:11113089:A:G	3	LINC00529	0	ncRNA_intronic	0.595
8:11174484:C:G	3	MTMR9:AF131216.6	00:00	ncRNA_exonic	0.323
8:11176403:A:G	3	MTMR9:AF131216.6	00:00	ncRNA_exonic	2.582
8:11177126:A:C	3	MTMR9:AF131216.6	00:00	ncRNA_exonic	0.057
8:11178093:A:C	3	MTMR9:AF131216.6	00:00	ncRNA_exonic	2.319
8:11179458:G:T	3	MTMR9:AF131216.6	00:00	ncRNA_intronic	2.907
8:11182148:C:G	3	MTMR9:AF131216.6	00:00	ncRNA_intronic	3.447
8:11182455:A:G	3	MTMR9:AF131216.6	00:00	ncRNA_intronic	0.238
8:11182704:A:G	3	MTMR9:AF131216.6	00:00	ncRNA_intronic	0.579

uniqID	Genomic Locus	nearestGene	dist	func	CADD
8:11183505:A:G	3	MTMR9	0	UTR3	0.564
8:11183765:C:CTTA	3	MTMR9	0	UTR3	8.068
8:11184390:A:C	3	MTMR9	0	UTR3	0.244
8:11184396:A:G	3	MTMR9	0	UTR3	0.277
8:11184478:C:CAA	3	MTMR9	0	UTR3	2.867
8:11184937:A:G	3	MTMR9	0	UTR3	7.477
8:11185096:C:G	3	MTMR9	0	UTR3	14.92
8:11185671:G:T	3	MTMR9	24	downstream	7.622
8:11185673:A:AT	3	MTMR9	26	downstream	7.945
8:11186215:A:C	3	MTMR9	568	downstream	1.755
8:11186453:C:T	3	MTMR9	806	downstream	0.35
8:11186639:C:G	3	MTMR9	992	downstream	0.869
8:11186674:A:G	3	MTMR9	1027	intergenic	1.684
8:11187078:G:T	3	SLC35G5	1318	intergenic	0.495
8:11187434:C:T	3	SLC35G5	962	upstream	1.617
8:11187651:A:G	3	SLC35G5	745	upstream	0.354
8:11187675:C:T	3	SLC35G5	721	upstream	0.354
8:11187770:C:G	3	SLC35G5	626	upstream	0.005
8:11188532:C:G	3	SLC35G5	0	UTR5	4.766
8:11188540:A:G	3	SLC35G5	0	UTR5	7.904
8:11188586:C:T	3	SLC35G5	0	UTR5	8.567
8:11188752:A:G	3	SLC35G5	0	exonic	22
8:11189488:C:T	3	SLC35G5	0	exonic	6.484
8:11189535:C:T	3	SLC35G5	0	exonic	4.662
8:11190647:C:G	3	SLC35G5	929	downstream	1.131
8:11191537:C:T	3	SLC35G5	1819	intergenic	3.346
8:11192551:C:G	3	SLC35G5	2833	intergenic	1.302
8:11192593:A:G	3	SLC35G5	2875	intergenic	1.855
8:11193530:C:T	3	TDH	3615	intergenic	1.777
8:11193736:G:T	3	TDH	3409	intergenic	4.232
8:11194457:A:G	3	TDH	2688	intergenic	0.36
8:11194911:A:G	3	TDH	2234	intergenic	2.87

uniqID	Genomic Locus	nearestGene	dist	func	CADD
8:11196295:C:G	3	TDH	850	upstream	0.718
8:11196970:C:T	3	TDH	175	upstream	2.749
8:11197301:C:G	3	TDH	0	ncRNA_intronic	5.741
8:11197323:G:T	3	TDH	0	ncRNA_intronic	3.534
8:11197598:C:T	3	TDH	0	ncRNA_intronic	3.494
8:11198579:C:CTTTT	3	TDH	0	ncRNA_intronic	0.47
8:11198792:A:G	3	TDH	0	ncRNA_intronic	2.13
8:11199584:A:G	3	TDH	0	ncRNA_intronic	1.598
8:11199938:A:C	3	TDH	0	ncRNA_intronic	1.801
8:11200454:A:AAT	3	TDH	0	ncRNA_intronic	6.214
8:11201504:G:T	3	TDH	0	ncRNA_intronic	1.86
8:11201605:A:ATT	3	TDH	0	ncRNA_intronic	0.02
8:11202154:A:G	3	TDH	0	ncRNA_intronic	3.985
8:11202960:A:G	3	TDH	0	ncRNA_intronic	1.895
8:11203107:A:T	3	TDH	0	ncRNA_intronic	2.258
8:11204165:C:T	3	TDH:AF131216.5	00:00	ncRNA_intronic	1.208
8:11204184:A:G	3	TDH:AF131216.5	00:00	ncRNA_intronic	4.89
8:11204503:C:G	3	TDH:AF131216.5	00:00	ncRNA_intronic	8.084
8:11204532:A:G	3	TDH:AF131216.5	00:00	ncRNA_intronic	5.608
8:11204755:C:T	3	TDH:AF131216.5	00:00	ncRNA_intronic	8.921
8:11205593:C:T	3	TDH	0	ncRNA_intronic	9.341
8:11205602:C:T	3	TDH	0	ncRNA_intronic	8.019
8:11205654:A:T	3	TDH	0	ncRNA_intronic	9.436
8:11205665:C:G	3	TDH	0	ncRNA_intronic	4.565
8:11205817:T:TA	3	TDH	0	ncRNA_intronic	8.083
8:11206220:C:G	3	TDH	0	ncRNA_intronic	4.87
8:11206262:C:T	3	TDH	0	ncRNA_intronic	7.808
8:11206363:C:G	3	TDH	0	ncRNA_intronic	2.289
8:11206543:A:G	3	TDH	0	ncRNA_intronic	0.484
8:11206627:C:G	3	TDH	0	ncRNA_intronic	6.061
8:11207326:A:C	3	TDH	0	ncRNA_intronic	1.448
8:11207367:A:C	3	TDH	0	ncRNA_intronic	0.318

uniqID	*Genomic Locus*	*nearestGene*	*dist*	*func*	*CADD*
8:11207431:C:T	3	TDH	0	ncRNA_intronic	1.138
8:11207508:C:T	3	TDH	0	ncRNA_intronic	6.239
8:11207672:C:G	3	TDH	0	ncRNA_intronic	0.255
8:11208903:A:G	3	TDH	0	ncRNA_intronic	0.953
8:11209499:C:T	3	TDH	0	ncRNA_intronic	9.636
8:11210823:G:T	3	TDH	0	ncRNA_intronic	3.326
8:11210824:G:T	3	TDH	0	ncRNA_intronic	2.595
8:11210828:C:T	3	TDH	0	ncRNA_intronic	2.072
8:11210983:A:G	3	TDH	0	ncRNA_intronic	2.152
8:11211068:C:T	3	TDH	0	ncRNA_intronic	8.026
8:11211302:G:T	3	TDH	0	ncRNA_intronic	2.824
8:11212081:A:G	3	TDH	0	ncRNA_intronic	2.264
8:11212599:C:T	3	TDH	0	ncRNA_exonic	1.786
8:11212650:A:AT	3	TDH	0	ncRNA_exonic	0.321
8:11212778:C:T	3	TDH	0	ncRNA_intronic	5.929
8:11212811:A:C	3	TDH	0	ncRNA_intronic	1.117
8:11212812:C:G	3	TDH	0	ncRNA_intronic	1.498
8:11212875:C:G	3	TDH	0	ncRNA_intronic	0.27
8:11213092:A:G	3	TDH	0	ncRNA_intronic	0.89
8:11213250:C:T	3	TDH	0	ncRNA_intronic	0.197
8:11213363:C:T	3	TDH	0	ncRNA_intronic	4.861
8:11213389:C:T	3	TDH	0	ncRNA_intronic	13.3
8:11213589:G:T	3	TDH	0	ncRNA_intronic	0.079
8:11213881:C:G	3	TDH	0	ncRNA_intronic	0.823
8:11214455:A:G	3	TDH	0	ncRNA_intronic	3.7
8:11214972:A:G	3	TDH	0	ncRNA_intronic	0.262
8:11215617:C:G	3	TDH	0	ncRNA_intronic	0.513
8:11215868:A:AT	3	TDH	0	ncRNA_intronic	0.479
8:11216761:G:T	3	TDH	0	ncRNA_exonic	13.95
8:11217284:C:T	3	TDH	0	ncRNA_intronic	1.709
8:11217441:A:G	3	TDH	0	ncRNA_intronic	3.963
8:11218893:A:G	3	TDH	0	ncRNA_splicing	14.74

uniqID	*Genomic Locus*	*nearestGene*	*dist*	*func*	*CADD*
8:11219334:A:G	3	TDH	0	ncRNA_intronic	0.398
8:11219386:G:T	3	TDH	0	ncRNA_intronic	1.856
8:11219781:C:T	3	TDH	0	ncRNA_intronic	4.25
8:11220846:C:CAA	3	TDH	0	ncRNA_intronic	1.568
8:11221313:C:T	3	TDH	0	ncRNA_intronic	0.445
8:11223022:A:C	3	TDH	0	ncRNA_intronic	3.295
8:11223793:G:T	3	TDH	0	ncRNA_intronic	2.87
8:11224313:C:T	3	TDH	0	ncRNA_intronic	0.17
8:11225168:A:G	3	TDH	0	ncRNA_intronic	0.418
8:11225480:C:T	3	TDH	0	ncRNA_intronic	0.398
8:11225910:A:G	3	TDH:C8orf12	00:00	ncRNA_exonic	0.293
8:11226071:A:G	3	C8orf12	0	intronic	2.691
8:11226456:A:C	3	C8orf12	0	intronic	0.53
8:11227104:A:G	3	C8orf12	0	intronic	1.426
8:11227406:A:G	3	C8orf12	0	intronic	3.042
8:11227885:C:T	3	C8orf12	0	intronic	0.248
8:11228006:A:G	3	C8orf12	0	intronic	2.856
8:11228100:A:G	3	C8orf12	0	intronic	1.674
8:11228254:A:G	3	C8orf12	0	intronic	0.561
8:11228672:A:G	3	C8orf12	0	intronic	2.822
8:11229319:C:G	3	C8orf12	0	intronic	0.337
8:11229638:C:T	3	C8orf12	0	intronic	2.917
8:11229889:A:G	3	C8orf12	0	intronic	0.115
8:11230206:G:T	3	C8orf12	0	intronic	2.811
8:11230259:A:T	3	C8orf12	0	intronic	2.428
8:11230574:A:G	3	C8orf12	0	intronic	0.19
8:11231249:C:T	3	C8orf12	0	intronic	0.646
8:11231354:G:T	3	C8orf12	0	intronic	4.995
8:11231886:A:G	3	C8orf12	0	intronic	8.262
8:11232343:C:G	3	C8orf12	0	intronic	14.38
8:11232788:A:G	3	C8orf12	0	intronic	5.899
8:11232860:A:G	3	C8orf12	0	intronic	1.562

uniqID	Genomic Locus	nearestGene	dist	func	CADD
8:11233318:A:C	3	C8orf12	0	intronic	6.373
8:11233419:G:T	3	C8orf12	0	intronic	1.64
8:11233582:A:G	3	C8orf12	0	intronic	3.772
8:11233659:A:G	3	C8orf12	0	intronic	4.043
8:11233917:C:T	3	C8orf12	0	intronic	0.215
8:11233958:A:G	3	C8orf12	0	intronic	2.323
8:11234298:C:T	3	C8orf12	0	intronic	0.087
8:11234367:G:T	3	C8orf12	0	intronic	0.956
8:11234500:A:T	3	C8orf12	0	intronic	3.108
8:11234520:A:G	3	C8orf12	0	intronic	1.936
8:11234613:A:T	3	C8orf12	0	intronic	5.325
8:11234626:C:T	3	C8orf12	0	intronic	2.092
8:11234780:C:G	3	C8orf12	0	intronic	0.478
8:11234844:A:G	3	C8orf12	0	intronic	0.451
8:11234885:C:G	3	C8orf12	0	intronic	2.009
8:11235136:C:G	3	C8orf12	0	intronic	1.53
8:11235150:A:C	3	C8orf12	0	intronic	0.082
8:11235360:A:C	3	C8orf12	0	intronic	1.486
8:11235393:A:G	3	C8orf12	0	intronic	2.024
8:11235497:A:G	3	C8orf12	0	intronic	0.33
8:11235579:G:T	3	C8orf12	0	intronic	4.501
8:11235605:G:T	3	C8orf12	0	intronic	1.698
8:11235614:A:G	3	C8orf12	0	intronic	2.104
8:11235735:C:T	3	C8orf12	0	intronic	0.148
8:11235910:A:G	3	C8orf12	0	intronic	0.645
8:11236392:C:T	3	C8orf12	0	intronic	4.437
8:11236413:C:T	3	C8orf12	0	intronic	2.361
8:11236419:C:T	3	C8orf12	0	intronic	0.261
8:11236572:A:T	3	C8orf12	0	intronic	10.93
8:11236681:G:T	3	C8orf12	0	intronic	1.926
8:11236685:C:T	3	C8orf12	0	intronic	5.579
8:11236809:C:T	3	C8orf12	0	intronic	5.18

uniqID	*Genomic Locus*	*nearestGene*	*dist*	*func*	*CADD*
8:11236850:C:T	3	C8orf12	0	intronic	0.043
8:11236964:C:T	3	C8orf12:RN7SL293P	00:00	ncRNA_exonic	3.381
8:11236975:A:G	3	C8orf12:RN7SL293P	00:00	ncRNA_exonic	4.398
8:11237330:C:G	3	C8orf12	0	intronic	4.354
8:11237477:A:G	3	C8orf12	0	intronic	3.855
8:11237480:A:G	3	C8orf12	0	intronic	0.548
8:11237587:C:CAAAA	3	C8orf12	0	intronic	0.904
8:11237591:A:C	3	C8orf12	0	intronic	3.028
8:11237756:A:C	3	C8orf12	0	intronic	1.401
8:11237773:C:T	3	C8orf12	0	intronic	1.65
8:11238029:C:T	3	C8orf12	0	intronic	4.941
8:11238315:C:T	3	C8orf12	0	intronic	4.801
8:11238316:G:T	3	C8orf12	0	intronic	2.607
8:11238332:G:T	3	C8orf12	0	intronic	5.788
8:11238587:A:G	3	C8orf12	0	intronic	4.59
8:11238597:C:T	3	C8orf12	0	intronic	2.289
8:11239017:A:T	3	C8orf12	0	intronic	0.286
8:11239054:C:T	3	C8orf12	0	intronic	0.009
8:11239078:G:T	3	C8orf12	0	intronic	0.718
8:11239137:A:G	3	C8orf12	0	intronic	1.824
8:11239297:G:GATAG	3	C8orf12	0	intronic	4.732
8:11239297:G:GATAT	3	C8orf12	0	NA	4.732
8:11239352:A:G	3	C8orf12	0	intronic	4.714
8:11239510:A:G	3	C8orf12	0	intronic	0.798
8:11239565:C:T	3	C8orf12	0	intronic	2.887
8:11239640:C:T	3	C8orf12	0	intronic	1.125
8:11239762:A:T	3	C8orf12	0	intronic	7.515
8:11239942:C:CT	3	C8orf12	0	intronic	0.696
8:11240571:C:T	3	C8orf12	0	intronic	1.086
8:11241935:G:GT	3	C8orf12	0	intronic	10.26
8:11242025:G:T	3	C8orf12	0	intronic	2.468
8:11242039:T:TA	3	C8orf12	0	intronic	1.535

uniqID	Genomic Locus	nearestGene	dist	func	CADD
8:11242632:A:G	3	C8orf12	0	intronic	1.013
8:11243126:C:G	3	C8orf12	0	intronic	0.907
8:11244841:A:G	3	C8orf12	0	intronic	0.042
8:11245064:C:T	3	C8orf12	0	intronic	2.829
8:11245303:C:G	3	C8orf12	0	intronic	4.095
8:11245562:A:T	3	C8orf12	0	intronic	0.919
8:11247298:A:G	3	C8orf12	0	intronic	0.401
8:11247814:A:G	3	C8orf12	0	intronic	0.572
8:11248500:C:T	3	C8orf12	0	intronic	1.825
8:11248956:C:T	3	C8orf12	0	intronic	3.157
8:11249010:G:T	3	C8orf12	0	intronic	1.583
8:11249261:C:T	3	C8orf12	0	intronic	1.008
8:11250848:A:C	3	C8orf12	0	intronic	1.532
8:11251175:A:G	3	C8orf12	0	intronic	0.986
8:11251705:A:G	3	C8orf12	0	intronic	0.901
8:11252170:C:T	3	C8orf12	0	intronic	0.099
8:11252425:A:C	3	C8orf12	0	intronic	0.718
8:11309192:C:T	3	FAM167A	0	intronic	10.02
8:11336781:A:G	3	FAM167A	4556	intergenic	0.231
8:11338146:A:G	3	FAM167A	5921	intergenic	2.425
8:11355602:C:G	3	BLK	0	intronic	0.254
8:11358156:C:T	3	BLK	0	intronic	3.056
8:11361261:C:G	3	BLK	0	intronic	0.43
8:11361850:G:GAGGA	3	BLK	0	intronic	0.638
8:11362275:A:C	3	BLK	0	intronic	0.265
8:11362277:G:T	3	BLK	0	intronic	1.093
8:11382367:A:G	3	BLK	0	intronic	0.042
8:11384556:C:T	3	BLK	0	intronic	2.083
8:11392093:A:C	3	BLK	0	intronic	3.651
8:11393764:A:G	3	BLK	0	intronic	1.294
8:11395079:A:G	3	BLK	0	intronic	0.367
8:11396856:A:C	3	BLK	0	intronic	1.911

uniqID	Genomic Locus	nearestGene	dist	func	CADD
8:11396874:A:G	3	BLK	0	intronic	0.553
8:11397073:A:C	3	BLK	0	intronic	6.644
8:11397086:A:T	3	BLK	0	intronic	6.509
8:11397457:C:G	3	BLK	0	intronic	0.433
8:11398865:A:G	3	BLK	0	intronic	0.855
8:11398953:C:T	3	BLK	0	intronic	3.613
8:11399484:A:T	3	BLK	0	intronic	0.514
8:11400628:C:G	3	BLK	0	intronic	1.923
8:11400680:A:G	3	BLK	0	intronic	2.637
8:11400944:G:T	3	BLK	0	intronic	8.189
8:11401116:A:G	3	BLK	0	intronic	9.069
8:11402347:A:G	3	BLK	0	intronic	0.031
8:11410513:A:C	3	BLK	0	intronic	2.599
8:11411005:C:G	3	BLK:RP11-148O21.3	00:00	ncRNA_exonic	0.662
8:11415184:C:G	3	BLK:RP11-148O21.4	00:00	ncRNA_intronic	1.476
8:11415572:A:G	3	BLK	0	intronic	7
8:11415597:C:T	3	BLK	0	intronic	1.31
8:11415794:A:T	3	BLK	0	intronic	12.6
8:11415812:C:T	3	BLK	0	intronic	3.952
8:11416171:C:T	3	BLK:RP11-148O21.2	00:00	ncRNA_exonic	0.797
8:11416428:C:T	3	BLK:RP11-148O21.2	00:00	ncRNA_exonic	2.832
8:11416635:A:G	3	BLK:RP11-148O21.2	00:00	ncRNA_intronic	3.356
8:11416885:C:T	3	BLK:RP11-148O21.2	00:00	ncRNA_intronic	1.527
8:11417016:A:ACT	3	BLK:RP11-148O21.2	00:00	ncRNA_intronic	2.756
8:11417144:C:T	3	BLK:RP11-148O21.2	00:00	ncRNA_intronic	6.135
8:11417150:G:T	3	BLK:RP11-148O21.2	00:00	ncRNA_intronic	3.414
8:11417257:A:G	3	BLK:RP11-148O21.2	00:00	ncRNA_intronic	1.129
8:11417493:C:T	3	BLK:RP11-148O21.2	00:00	ncRNA_exonic	4.147
8:11417582:C:T	3	BLK	0	intronic	6.773
8:11418385:A:G	3	BLK	0	intronic	3.446
8:11418773:C:T	3	BLK	0	intronic	5.889
8:11419335:A:AT	3	BLK	0	intronic	0.088

uniqID	*Genomic Locus*	*nearestGene*	*dist*	*func*	*CADD*
8:11419852:C:T	3	BLK	0	intronic	0.213
8:11419861:G:T	3	BLK	0	intronic	1.014
8:11420104:C:G	3	BLK	0	intronic	0.642
8:11420221:A:G	3	BLK	0	intronic	0.375
8:11420295:C:T	3	BLK	0	intronic	3.616
8:11421016:A:G	3	BLK	0	intronic	2.018
8:11421358:A:C	3	BLK	0	intronic	2.157
8:11421384:C:T	3	BLK	0	intronic	6.665
8:11421793:C:T	3	BLK	0	UTR3	10.12
8:11422045:A:G	3	BLK	0	UTR3	10.74
8:11422130:C:T	3	BLK	16	downstream	10.52
8:11422170:A:G	3	BLK	56	downstream	8.243
8:11422289:C:G	3	BLK	175	downstream	1.012
8:11422442:A:C	3	BLK	328	downstream	8.058
8:11422491:A:C	3	BLK	377	downstream	2.275
8:11422492:C:G	3	BLK	378	downstream	0.574
8:11422494:A:AT	3	BLK	380	downstream	0.572
8:11422521:G:GA	3	BLK	407	downstream	4.036
8:11422861:C:G	3	BLK	747	downstream	1.233
8:11422936:C:T	3	BLK	822	downstream	6.434
8:11423072:A:G	3	BLK	958	downstream	7.57
8:11423083:A:C	3	BLK	969	downstream	4.608
8:11423142:A:G	3	BLK	1028	intergenic	4.855
8:11423434:A:G	3	BLK	1320	intergenic	7.415
8:11423537:A:G	3	BLK	1423	intergenic	1.13
8:11423781:C:G	3	BLK	1667	intergenic	2.101
8:11425077:C:T	3	BLK	2963	intergenic	0.451
8:11425081:G:T	3	BLK	2967	intergenic	4.213
8:11425105:G:T	3	BLK	2991	intergenic	0.373
8:11425809:G:T	3	BLK	3695	intergenic	3.475
8:11426400:G:T	3	BLK	4286	intergenic	3.736
8:11426790:C:G	3	BLK	4676	intergenic	1.063

uniqID	*Genomic Locus*	*nearestGene*	*dist*	*func*	*CADD*
8:11427133:G:T	3	BLK	5019	intergenic	0.308
8:11427341:G:T	3	BLK	5227	intergenic	0.701
8:11427637:A:T	3	BLK	5523	intergenic	4.827
8:11428395:C:T	3	LINC00208	5426	intergenic	0.711
8:11430485:A:G	3	LINC00208	3336	intergenic	0.543
8:11430990:C:T	3	LINC00208	2831	intergenic	0.042
8:11431558:T:TAC	3	LINC00208	2263	intergenic	0.204
8:11431943:T:TAA	3	LINC00208	1878	intergenic	0.02
8:11432085:A:ACG	3	LINC00208	1736	intergenic	1.509
8:11432085:A:ACACG	3	LINC00208	1736	NA	1.587
8:11432438:C:G	3	LINC00208	1383	intergenic	4.309
8:11432453:C:T	3	LINC00208	1368	intergenic	3.286
8:11432946:A:C	3	LINC00208	875	upstream	2.867
8:11433780:C:T	3	LINC00208	41	upstream	0.025
8:11433909:C:T	3	LINC00208	0	ncRNA_exonic	3.539
8:11434176:A:T	3	LINC00208	0	ncRNA_exonic	2.916
8:11434232:C:T	3	LINC00208	0	ncRNA_exonic	0.306
8:11434415:C:CTCGGTTT	3	LINC00208	0	ncRNA_exonic	4.114
8:11434792:C:G	3	LINC00208	0	ncRNA_exonic	1.963
8:11434929:C:T	3	LINC00208	0	ncRNA_intronic	0.875
8:11435049:A:G	3	LINC00208	0	ncRNA_intronic	0.072
8:11435291:G:GTGGC	3	LINC00208	0	ncRNA_intronic	0.725
8:11435516:C:T	3	LINC00208	0	ncRNA_intronic	1.67
8:11435564:C:T	3	LINC00208	0	ncRNA_intronic	2.009
8:11435927:C:G	3	LINC00208	0	ncRNA_exonic	3.254
8:11438064:G:T	3	LINC00208	0	ncRNA_exonic	0.484
8:11439225:A:G	3	LINC00208	373	downstream	0.142
8:11440019:A:G	3	LINC00208	1167	intergenic	2.025
8:11444516:A:G	3	LINC00208	5664	intergenic	0.019
8:11444837:C:T	3	LINC00208	5985	intergenic	0.37
8:11446421:A:G	3	LINC00208	7569	intergenic	2.107
8:11446637:A:G	3	LINC00208	7785	intergenic	0.282

uniqID	Genomic Locus	nearestGene	dist	func	CADD
8:11446652:A:G	3	LINC00208	7800	intergenic	1.483
8:11446680:C:T	3	LINC00208	7828	intergenic	3.541
8:11446800:C:T	3	LINC00208	7948	intergenic	2.108
8:11446868:C:T	3	LINC00208	8016	intergenic	7.794
8:11446955:A:G	3	LINC00208	8103	intergenic	1.776
8:11447093:A:G	3	LINC00208	8241	intergenic	0.836
8:11447119:C:T	3	LINC00208	8267	intergenic	3.088
8:11447679:C:G	3	LINC00208	8827	intergenic	1.295
8:11448659:C:G	3	LINC00208	9807	intergenic	4.266
8:11450133:A:G	3	LINC00208	11281	intergenic	0.998
8:11450422:A:G	3	LINC00208	11570	intergenic	1.775
8:11450587:G:T	3	LINC00208	11735	intergenic	0.463
8:11460909:T:TA	3	LINC00208	22057	intergenic	0.944
8:11461111:A:G	3	LINC00208	22259	intergenic	0.311
8:11466745:A:T	3	LINC00208	27893	intergenic	2.164
8:11467557:C:G	3	LINC00208	28705	intergenic	8.517
12:62830952:C:T	4	USP15	19740	intergenic	0.366
12:62831343:C:T	4	USP15	20131	intergenic	3.851
12:62837767:A:G	4	MON2	22829	intergenic	8.124
12:62847085:C:G	4	MON2	13511	intergenic	0.205
12:62848152:A:G	4	MON2	12444	intergenic	5.294
12:62849418:A:G	4	MON2	11178	intergenic	5.096
12:62851080:A:AC	4	MON2	9516	intergenic	0.161
12:62852271:A:G	4	MON2	8325	intergenic	1.554
12:62852916:A:G	4	MON2	7680	intergenic	1.166
12:62855388:C:T	4	MON2	5208	intergenic	0.035
12:62858342:A:G	4	MON2	2254	intergenic	0.806
12:62858561:C:T	4	MON2	2035	intergenic	0.25
12:62859241:A:AC	4	MON2	1355	intergenic	0.209
12:62861935:T:TA	4	MON2	0	intronic	4.258
12:62862739:G:T	4	MON2	0	intronic	2.853
12:62865152:C:T	4	MON2	0	intronic	0.057

uniqID	Genomic Locus	nearestGene	dist	func	CADD
12:62865291:A:G	4	MON2	0	intronic	17.38
12:62868455:C:T	4	MON2	0	intronic	6.552
12:62868499:C:T	4	MON2	0	intronic	0.688
12:62868500:A:C	4	MON2	0	intronic	1.141
12:62870574:C:G	4	MON2	0	intronic	12.04
12:62875839:C:T	4	MON2	0	intronic	0.415
12:62880276:C:G	4	MON2	0	intronic	4.482
12:62884714:A:ATTT	4	MON2	0	intronic	5.955
12:62884972:A:G	4	MON2	0	intronic	2.49
12:62886001:A:T	4	MON2	0	intronic	6.743
12:62886372:C:G	4	MON2	0	intronic	1.624
12:62886649:C:T	4	MON2	0	intronic	0.64
12:62888219:A:C	4	MON2	0	intronic	8.628
12:62889402:A:G	4	MON2	0	intronic	0.547
12:62889409:A:G	4	MON2	0	intronic	2.917
12:62890491:A:C	4	MON2	0	intronic	0.608
12:62891828:C:CT	4	MON2	0	intronic	2.052
12:62894058:A:G	4	MON2	0	intronic	4.943
12:62897930:A:G	4	MON2	0	intronic	0.788
12:62898111:A:G	4	MON2	0	intronic	0.439
12:62898463:C:CT	4	MON2	0	intronic	4.501
12:62898490:C:T	4	MON2	0	intronic	13.47
12:62902420:C:T	4	MON2	0	intronic	5.76
12:62903639:A:G	4	MON2	0	intronic	2.129
12:62903655:G:GT	4	MON2	0	intronic	0.527
12:62903793:A:G	4	MON2	0	intronic	5.794
12:62904252:C:T	4	MON2	0	intronic	0.16
12:62905380:C:CA	4	MON2	0	intronic	11.44
12:62905772:A:AC	4	MON2	0	intronic	0.954
12:62906488:A:G	4	MON2	0	intronic	0.206
12:62910714:A:T	4	MON2	0	intronic	3.425
12:62914776:T:TA	4	MON2	0	intronic	0.035

uniqID	Genomic Locus	nearestGene	dist	func	CADD
12:62917704:A:G	4	MON2	0	intronic	7.458
12:62920860:C:G	4	MON2	0	intronic	0.331
12:62921257:C:T	4	MON2	0	intronic	0.604
12:62922143:A:G	4	MON2	0	intronic	1.086
12:62926398:A:G	4	MON2	0	exonic	4.094
12:62928006:T:TA	4	MON2	0	intronic	9.725
12:62928633:A:G	4	MON2	0	exonic	22.2
12:62930621:A:G	4	MON2	0	intronic	1.7
12:62930798:C:T	4	MON2	0	intronic	1.385
12:62931846:A:ATTTGT	4	MON2	0	intronic	5.01
12:62932816:A:G	4	MON2	0	intronic	0.594
12:62935154:C:CA	4	MON2	0	intronic	0.829
12:62935705:C:T	4	MON2	0	intronic	1.512
12:62937348:G:T	4	MON2	0	intronic	1.592
12:62937532:C:T	4	MON2	0	intronic	0.549
12:62939055:C:T	4	MON2	0	intronic	2.072
12:62941426:C:T	4	MON2	0	intronic	4.232
12:62941444:A:G	4	MON2	0	intronic	1.013
12:62941837:A:G	4	MON2	0	intronic	0.389
12:62941929:A:T	4	MON2	0	intronic	1.022
12:62942258:C:T	4	MON2	0	intronic	3.639
12:62945158:G:GGAAACAACCTGACGAT	4	MON2	0	intronic	0.824
12:62945245:A:G	4	MON2	0	intronic	2.468
12:62945970:C:G	4	MON2	0	intronic	0.022
12:62948736:A:G	4	MON2	0	intronic	1.539
12:62949110:A:C	4	MON2	0	intronic	3.79
12:62950556:C:G	4	MON2	0	intronic	0.964
12:62952696:C:T	4	MON2	0	intronic	0.665
12:62952910:C:T	4	MON2	0	intronic	0.387
12:62957906:C:G	4	MON2	0	intronic	1.425
12:62960880:A:G	4	MON2	0	intronic	0.317
12:62961299:C:T	4	MON2	0	intronic	0.418

uniqID	Genomic Locus	nearestGene	dist	func	CADD
12:62961801:A:G	4	MON2	0	intronic	14.42
12:62964552:C:T	4	MON2	0	intronic	0.725
12:62968280:C:T	4	MON2	0	intronic	3.801
12:62968740:C:CA	4	MON2	0	intronic	1.134
12:62970482:A:G	4	MON2	0	intronic	8.697
12:62972210:G:GT	4	MON2	0	intronic	6.131
12:62975307:C:T	4	MON2	0	intronic	8.418
12:62977162:A:G	4	MON2	0	intronic	4.379
12:62977163:A:G	4	MON2	0	intronic	2.991
12:62977175:A:AG	4	MON2	0	intronic	0.663
12:62980580:A:T	4	MON2	0	intronic	1.964
12:62982152:C:CT	4	MON2	0	intronic	0.434
12:62985871:A:C	4	MON2	0	intronic	1.573
12:62986620:A:G	4	MON2	0	UTR3	5.166
12:62988288:A:G	4	MON2	0	UTR3	3.65
12:62989110:A:C	4	MON2	0	UTR3	2.64
12:62990403:A:G	4	MON2	0	UTR3	1.187
12:62990415:C:T	4	MON2	0	UTR3	5.356
12:62990871:A:C	4	MON2	0	UTR3	0.274
12:62992896:G:GA	4	MON2	1532	intergenic	0.466
12:62993793:A:G	4	C12orf61	1737	intergenic	2.935
12:62995269:A:G	4	C12orf61	261	downstream	3.248
12:62995340:A:G	4	C12orf61	190	downstream	10.81
12:62995984:C:T	4	C12orf61	0	UTR3	4.366
12:62996061:A:G	4	C12orf61	0	UTR3	7.271
12:62997180:C:T	4	C12orf61:RP11-631N16.2	00:00	ncRNA_intronic	8.091
12:62999154:C:G	4	RP11-631N16.2	0	ncRNA_intronic	6.226
12:63001068:A:AT	4	RP11-631N16.2	0	ncRNA_intronic	0.137
13:83298475:A:G	5	GYG1P2	98790	intergenic	2.716
13:83299269:C:T	5	GYG1P2	97996	intergenic	0.528
13:83300084:A:G	5	GYG1P2	97181	intergenic	0.305
13:83300138:A:AGAGAGC	5	GYG1P2	97127	intergenic	0.646

uniqID	Genomic Locus	nearestGene	dist	func	CADD
13:83300269:C:T	5	GYG1P2	96996	intergenic	0.096
13:83300359:A:G	5	GYG1P2	96906	intergenic	0.331
13:83302594:C:T	5	GYG1P2	94671	intergenic	0.263
13:83302795:A:T	5	GYG1P2	94470	intergenic	2.481
13:83304274:C:T	5	GYG1P2	92991	intergenic	6.255
13:83304898:G:T	5	GYG1P2	92367	intergenic	0.832
13:83306586:A:G	5	GYG1P2	90679	intergenic	0.529
13:83311141:A:G	5	GYG1P2	86124	intergenic	1.455
13:83312341:C:T	5	GYG1P2	84924	intergenic	1.149
13:83312717:C:G	5	GYG1P2	84548	intergenic	0.67
16:13021889:C:T	6	SHISA9	0	intronic	5.19
16:13022033:A:AT	6	SHISA9	0	intronic	0.394
16:13023207:C:T	6	SHISA9	0	intronic	1.744
16:13023388:A:G	6	SHISA9	0	intronic	0.702
16:13023394:A:G	6	SHISA9	0	intronic	0.612
16:13024150:A:G	6	SHISA9	0	intronic	2.228
16:13025315:G:T	6	SHISA9	0	intronic	0.331
16:13026502:A:G	6	SHISA9	0	intronic	3.705
16:13029711:G:GA	6	SHISA9	0	intronic	1.02
16:13030222:A:G	6	SHISA9	0	intronic	0.303
16:13030875:A:G	6	SHISA9	0	intronic	7.135
16:13031195:A:C	6	SHISA9	0	intronic	1.656
16:13032351:C:G	6	SHISA9	0	intronic	1.596
16:13032547:A:G	6	SHISA9	0	intronic	0.354
16:13032863:A:T	6	SHISA9	0	intronic	6.471
16:13035206:T:TA	6	SHISA9	0	intronic	0.103
16:13036811:C:T	6	SHISA9	0	intronic	6.27
16:13037305:C:T	6	SHISA9	0	intronic	6.084
16:13038054:G:T	6	SHISA9	0	intronic	0.838
16:13038196:A:G	6	SHISA9	0	intronic	2.321
16:13038723:G:T	6	SHISA9	0	intronic	4.269
16:13039154:C:T	6	SHISA9	0	intronic	0.774

uniqID	Genomic Locus	nearestGene	dist	func	CADD
16:13039642:A:T	6	SHISA9	0	intronic	0.153
16:13039646:C:T	6	SHISA9	0	intronic	0.227
16:13040514:C:T	6	SHISA9	0	intronic	2.022
16:13040889:C:G	6	SHISA9	0	intronic	0.618
16:13041027:G:T	6	SHISA9	0	intronic	2.795
16:13041921:C:T	6	SHISA9	0	intronic	2.982
16:13041924:A:G	6	SHISA9	0	intronic	6.099
16:13041940:A:C	6	SHISA9	0	intronic	0.074
16:13042097:A:G	6	SHISA9	0	intronic	13.23
16:13043452:A:G	6	SHISA9	0	intronic	5.192
16:13044033:C:T	6	SHISA9	0	intronic	2.077
16:13044827:C:T	6	SHISA9	0	intronic	0.396
16:13045117:C:CA	6	SHISA9	0	intronic	0.646
16:13047022:C:G	6	SHISA9	0	intronic	1.271
16:13047598:C:G	6	SHISA9	0	intronic	5.302
16:13047734:C:T	6	SHISA9	0	intronic	0.025
16:13048395:C:T	6	SHISA9	0	intronic	6.099
16:13048841:C:T	6	SHISA9	0	intronic	2.929
16:13048916:G:GT	6	SHISA9	0	intronic	0.18
16:13049490:T:TA	6	SHISA9	0	intronic	3.55
16:13049557:C:T	6	SHISA9	0	intronic	2.982
16:13049749:A:C	6	SHISA9	0	intronic	2.541
16:13049906:A:T	6	SHISA9	0	intronic	0.557
16:13050340:A:C	6	SHISA9	0	intronic	8.215
16:13051686:A:C	6	SHISA9	0	intronic	10.84
16:13052050:A:G	6	SHISA9	0	intronic	0.245
16:13052714:C:G	6	SHISA9	0	intronic	0.427
16:13052846:A:G	6	SHISA9	0	intronic	5.403
16:13053072:A:G	6	SHISA9	0	intronic	11.53
16:13053136:G:T	6	SHISA9	0	intronic	0.977
16:13053140:C:G	6	SHISA9	0	intronic	7.368
16:13053187:C:T	6	SHISA9	0	intronic	8.439

uniqID	*Genomic Locus*	*nearestGene*	*dist*	*func*	*CADD*
16:13053270:A:G	6	SHISA9	0	intronic	0.116
16:13053457:A:AC	6	SHISA9	0	intronic	0.367
16:13053576:A:T	6	SHISA9	0	intronic	4.662
16:13053882:A:G	6	SHISA9	0	intronic	6.437
16:13053924:C:G	6	SHISA9	0	intronic	0.189
16:13053951:C:T	6	SHISA9	0	intronic	13.54
16:13054037:C:G	6	SHISA9	0	intronic	6.68
16:13054265:A:G	6	SHISA9	0	intronic	1.912
16:13054645:C:T	6	SHISA9	0	intronic	5.104
16:13054883:C:G	6	SHISA9	0	intronic	1.632
16:13054897:A:C	6	SHISA9	0	intronic	3.295
16:13055220:C:T	6	SHISA9	0	intronic	1.037
16:13055621:T:TCC	6	SHISA9	0	intronic	2.733
16:13055766:C:T	6	SHISA9	0	intronic	11.99
16:13057200:A:G	6	SHISA9	0	intronic	0.058
16:13058241:G:GT	6	SHISA9	0	intronic	1.705
16:13059539:C:T	6	SHISA9	0	intronic	1.061
16:13061109:A:G	6	SHISA9	0	intronic	2.338
16:13062232:A:G	6	SHISA9	0	intronic	0.804
16:13066833:C:T	6	SHISA9	0	intronic	0.149
16:13070238:A:G	6	SHISA9	0	intronic	0.72
16:13070671:C:T	6	SHISA9	0	intronic	5.193
16:13070809:C:G	6	SHISA9	0	intronic	0.578
16:13075745:A:G	6	SHISA9	0	intronic	5.049
16:13077351:C:T	6	SHISA9	0	intronic	6.158
16:13077901:A:G	6	SHISA9	0	intronic	5.813
16:13077915:A:G	6	SHISA9	0	intronic	6.365
16:13078044:A:T	6	SHISA9	0	intronic	11.07
16:13078807:C:T	6	SHISA9	0	intronic	0.588
16:13079063:C:CTT	6	SHISA9	0	intronic	2.194
16:13079082:A:C	6	SHISA9	0	intronic	3.279
16:13079214:A:G	6	SHISA9	0	intronic	1.107

uniqID	Genomic Locus	nearestGene	dist	func	CADD
16:13079389:G:T	6	SHISA9	0	intronic	3.309
16:13079463:C:T	6	SHISA9	0	intronic	4.718
16:13079698:A:T	6	SHISA9	0	intronic	0.363
16:13080252:A:G	6	SHISA9	0	intronic	0.757
16:13080515:C:T	6	SHISA9	0	intronic	3.566
16:13081923:C:G	6	SHISA9	0	intronic	2.457
16:13082445:A:G	6	SHISA9	0	intronic	2.422
16:13083055:G:T	6	SHISA9	0	intronic	7.401
16:13083068:G:GT	6	SHISA9	0	intronic	0.631
16:13086810:C:T	6	SHISA9	0	intronic	3.294
16:13088905:C:T	6	SHISA9	0	intronic	3.136
16:13089059:A:G	6	SHISA9	0	intronic	0.275
16:13089854:G:T	6	SHISA9	0	intronic	4.927
16:13091332:C:T	6	SHISA9	0	intronic	0.023
16:13092100:A:G	6	SHISA9	0	intronic	4.241
16:13092220:C:T	6	SHISA9	0	intronic	1.601
16:13092663:C:T	6	SHISA9	0	intronic	4.331
16:13093774:G:T	6	SHISA9	0	intronic	2.987
16:13093778:C:T	6	SHISA9	0	intronic	2.641
16:13093858:C:T	6	SHISA9	0	intronic	5.85
16:13094769:G:T	6	SHISA9	0	intronic	7.55
16:13094897:C:G	6	SHISA9	0	intronic	3.361
16:13095142:C:G	6	SHISA9	0	intronic	9.344
16:13095171:C:T	6	SHISA9	0	intronic	7.956
16:13095296:A:T	6	SHISA9	0	intronic	4.542
16:13095739:A:C	6	SHISA9	0	intronic	9.648
16:13096300:C:T	6	SHISA9	0	intronic	0.266
16:13097084:G:T	6	SHISA9	0	intronic	2.854
16:13097125:A:G	6	SHISA9	0	intronic	4.784
16:13097206:C:G	6	SHISA9	0	intronic	10.25
16:13097746:G:T	6	SHISA9	0	intronic	8.377
16:13097749:G:T	6	SHISA9	0	intronic	6.476

uniqID	Genomic Locus	nearestGene	dist	func	CADD
16:13098440:C:G	6	SHISA9	0	intronic	1.645
16:13098508:A:G	6	SHISA9	0	intronic	0.372
16:13098762:A:C	6	SHISA9	0	intronic	2.113
16:13098934:A:G	6	SHISA9	0	intronic	6.741
16:13099114:C:G	6	SHISA9	0	intronic	7.072
16:13099177:G:T	6	SHISA9	0	intronic	1.897
16:13099919:A:G	6	SHISA9	0	intronic	1.291
16:13099953:G:T	6	SHISA9	0	intronic	4.047
16:13100021:C:T	6	SHISA9	0	intronic	9.243
16:13101555:C:T	6	SHISA9	0	intronic	0.07
16:13101618:A:G	6	SHISA9	0	intronic	4.209
16:13102532:C:T	6	SHISA9	0	intronic	2.573
16:13102906:A:T	6	SHISA9	0	intronic	1.494
16:13104924:C:T	6	SHISA9	0	intronic	0.494
16:13105091:A:C	6	SHISA9	0	intronic	0.902
16:13105863:C:T	6	SHISA9	0	intronic	1.341
16:13105998:C:T	6	SHISA9	0	intronic	2.094
16:13109594:G:T	6	SHISA9	0	intronic	9.678
16:13112335:C:T	6	SHISA9	0	intronic	1.172
16:13115702:C:G	6	SHISA9	0	intronic	1.687
16:13115755:A:G	6	SHISA9	0	intronic	3.883
16:13116871:T:TTTTA	6	SHISA9	0	intronic	0.442
16:13118299:A:T	6	SHISA9	0	intronic	12.68

Abbreviations: chr = chromosome; pos = posce to nearest gene; func = function; CADD = Combined Annotation Dependent De
across 127 tissue/cell type; commonChrStat

Table S6: Gene mapping FUMA

uniqID	*RDB*	*minChrState*	*commonChrState*	*posMapFilt*	*eqtlMapFilt*	*ciMapFilt*
2:98325330:G:T	3a	5	15	1	1	0
2:98326867:A:G	7	5	15	1	1	0
2:98330052:A:C	NA	1	15	1	1	0
2:98334679:C:T	5	1	15	1	1	0
2:98336200:A:C	5	2	15	1	1	0
2:98339513:C:T	5	4	15	1	1	0
2:98342323:A:C	5	4	15	1	1	0
2:98343258:A:G	5	4	15	1	1	0
2:98345086:G:T	5	2	15	1	1	0
2:98346461:A:G	7	4	9	1	1	0
2:98351654:T:TG	NA	1	15	1	1	0
2:98351986:C:T	5	1	15	1	1	0
2:98353847:C:T	5	4	15	1	1	0
2:98354139:G:T	5	4	15	1	1	0
2:98354511:A:G	5	4	15	1	1	0
2:98355990:A:G	5	4	15	1	1	0
2:98356846:A:G	5	4	15	1	1	0
2:98357163:C:T	4	4	15	1	1	0
2:98360443:A:G	2b	4	5	1	1	0
2:98361679:C:G	7	4	5	1	1	0
2:98363313:A:T	5	4	5	1	1	0
2:98365164:C:G	4	2	5	1	1	0
2:98368551:A:ACT	NA	4	5	1	1	0
2:98370698:A:T	6	4	5	1	1	0
2:98374567:C:T	7	4	4	1	1	0
2:98377512:A:T	5	3	4	1	1	0
2:98379267:C:T	7	4	4	1	1	0
2:98379813:A:G	5	2	4	1	1	0
2:98382886:A:G	5	2	4	1	1	0
2:98384528:A:ATTTTC	NA	4	5	1	1	0
2:98386731:C:T	5	2	5	1	1	0

uniqID	*RDB*	*minChrState*	*commonChrState*	*posMapFilt*	*eqtlMapFilt*	*ciMapFilt*
2:98390117:G:GT	NA	4	4	1	1	0
2:98393231:C:T	5	4	5	1	1	0
2:98393894:C:T	7	4	5	1	1	0
2:98395653:A:C	3a	4	5	1	1	0
2:98397301:C:T	7	4	5	1	1	0
2:98402753:A:T	6	4	5	1	1	0
2:98402772:C:G	6	4	5	1	1	0
2:98405034:C:T	7	4	4	1	1	0
2:98405695:C:T	4	4	4	1	1	0
2:98405929:A:G	3a	4	4	1	1	0
2:98407350:C:T	7	4	5	1	1	0
2:98409046:A:G	5	4	4	1	1	0
2:98409565:C:T	6	4	4	1	1	0
2:98410769:G:T	6	4	5	1	1	0
2:98413781:C:T	7	4	5	1	1	0
2:98416848:A:C	NA	4	5	1	1	0
2:98416850:G:T	NA	4	5	1	1	0
2:98417089:C:T	6	4	5	1	1	0
2:98419726:A:T	7	4	5	1	1	0
2:98420142:C:G	7	4	4	1	1	0
2:98420431:C:T	7	4	5	1	1	0
2:98421364:A:G	6	4	4	1	1	0
2:98424802:A:G	4	4	5	1	1	0
2:98427382:C:T	5	4	4	1	1	0
2:98434931:G:GGGGGC	NA	4	5	1	1	0
2:98435982:C:T	5	4	5	1	1	0
2:98440234:C:T	5	4	5	1	1	0
2:98442202:A:G	7	4	5	1	1	0
2:98443039:C:G	4	4	5	1	1	0
2:98443081:G:GA	NA	4	5	1	1	0
2:98443658:C:T	5	4	5	1	1	0
2:98447004:C:T	NA	4	5	1	1	0

uniqID	*RDB*	*minChrState*	*commonChrState*	*posMapFilt*	*eqtlMapFilt*	*ciMapFilt*
2:98448395:G:T	7	4	5	1	1	0
2:98449024:A:G	6	4	5	1	1	0
2:98454472:A:G	5	4	5	1	1	0
2:98454473:C:T	5	4	5	1	1	0
2:98454572:C:G	7	4	5	1	1	0
2:98454931:A:G	7	4	5	1	1	0
2:98455152:C:T	7	4	5	1	1	0
2:98455510:C:G	7	4	5	1	1	0
2:98461821:C:T	5	4	5	1	1	0
2:98461953:C:T	7	4	5	1	1	0
2:98466647:C:T	4	4	5	1	1	0
2:98468892:A:AC	NA	4	5	1	1	0
2:98472610:C:T	6	4	5	1	1	0
2:98479581:A:C	6	4	5	1	1	0
2:98488949:C:G	6	4	5	1	1	0
2:98491750:C:T	6	5	5	1	1	0
2:98493359:A:G	6	5	5	1	1	0
2:98495661:G:T	6	4	5	1	1	0
2:98501884:C:T	6	4	5	1	1	0
2:98502987:C:T	7	4	8	1	1	0
2:98506011:T:TATA	NA	4	5	1	1	0
2:98506910:C:T	4	4	5	1	1	0
2:98507972:C:CA	NA	5	5	1	1	0
2:98510166:G:T	7	5	5	1	1	0
2:98517626:A:G	5	5	5	1	1	0
2:98519014:C:T	6	4	5	1	1	0
2:98520564:C:T	6	4	5	1	1	0
2:98521823:A:C	5	4	5	1	1	0
2:98526685:A:AAAAT	NA	2	5	1	1	0
2:98528689:A:G	2c	1	5	1	1	0
2:98534412:C:T	3a	4	5	1	1	0
2:98534531:C:T	6	4	5	1	1	0

uniqID	*RDB*	*minChrState*	*commonChrState*	*posMapFilt*	*eqtlMapFilt*	*ciMapFilt*
2:98539588:A:C	7	4	5	1	1	0
2:98541001:C:T	7	4	4	1	1	0
2:98547069:A:AT	NA	2	5	1	1	0
2:98548604:T:TA	NA	5	5	1	1	0
2:98551456:G:GA	NA	5	5	1	1	0
2:98552271:C:G	4	1	7	1	1	0
2:98552299:C:G	2b	1	7	1	1	0
2:98554946:A:C	7	5	15	1	1	0
2:98557575:G:T	6	5	15	1	1	0
2:98561153:A:C	7	5	15	1	1	0
2:98565115:C:CT	NA	4	15	1	1	0
2:98565144:A:G	5	4	15	1	1	0
2:98565400:A:G	7	2	15	1	1	0
2:98567820:A:G	3a	5	7	1	1	0
2:98570999:A:G	5	2	15	1	1	0
2:98571084:A:G	5	5	15	1	1	0
2:98580012:C:T	7	5	5	1	1	0
2:98580724:G:T	7	5	5	1	1	0
2:98587288:A:G	5	5	15	1	1	0
2:98588372:A:C	7	5	15	1	1	0
2:98602165:A:G	6	5	5	1	1	0
2:98604570:G:GACAC	NA	5	5	1	1	0
2:98605982:A:G	5	2	15	1	1	0
2:98606770:C:T	NA	2	15	1	1	0
2:98612260:C:T	NA	1	1	1	1	0
2:98616519:A:ATT	NA	5	15	1	1	0
2:98621058:A:AG	NA	5	15	1	1	0
2:98621060:A:T	7	5	15	1	1	0
2:98623227:A:G	7	5	15	0	1	0
2:98623406:A:G	6	5	15	0	1	0
8:8524474:C:G	6	5	15	0	1	0
8:8543324:C:T	5	2	15	0	1	0

uniqID	*RDB*	*minChrState*	*commonChrState*	*posMapFilt*	*eqtlMapFilt*	*ciMapFilt*
8:8544808:C:T	NA	1	15	0	1	0
8:8544872:A:G	6	1	15	0	1	0
8:8545624:T:TTTG	NA	5	15	0	1	0
8:8546283:A:AAATT	NA	5	15	0	1	0
8:8547110:C:G	1f	2	15	0	1	0
8:8547313:A:C	5	5	15	0	1	0
8:8547811:A:G	5	5	15	0	1	0
8:8548117:A:T	6	5	15	0	1	0
8:8548801:A:G	6	5	15	0	1	0
8:8549020:A:C	6	5	15	0	1	0
8:8549432:A:G	7	2	15	0	1	0
8:8578067:C:T	5	9	14	0	1	0
8:8578120:A:T	5	9	14	0	1	0
8:8578229:A:G	5	7	14	0	1	0
8:8578794:A:T	3a	7	14	0	1	0
8:8578811:C:T	NA	7	14	0	1	0
8:8581408:C:G	5	7	13	0	1	0
8:8582155:A:AAAAAGAAAAG	NA	13	13	0	1	0
8:8582155:AAAAAG:AAAAAGAAAAG	NA	13	13	0	0	0
8:8583872:C:T	6	13	14	0	1	0
8:8584344:A:G	NA	13	14	0	1	0
8:8587571:G:T	NA	13	14	0	1	0
8:8589117:C:T	NA	13	14	0	1	0
8:8592845:C:G	6	5	14	0	1	0
8:8595104:A:T	6	5	14	0	1	0
8:8595838:G:T	5	5	14	0	1	0
8:8596731:G:T	6	5	14	0	1	0
8:8598388:A:T	5	5	14	0	1	0
8:8602344:C:T	2b	5	15	0	1	0
8:8603160:A:G	5	5	15	0	1	0
8:8633548:G:T	NA	5	15	1	1	0
8:8637429:A:G	2b	2	7	1	1	0

uniqID	*RDB*	*minChrState*	*commonChrState*	*posMapFilt*	*eqtlMapFilt*	*ciMapFilt*
8:8639740:A:G	4	1	5	1	1	0
8:8640172:A:C	NA	1	5	1	1	0
8:8641145:C:T	1f	2	5	1	1	0
8:8643938:C:T	5	4	5	1	1	0
8:8644213:G:GT	NA	5	5	1	1	0
8:8644213:G:GTT	NA	5	5	1	1	0
8:8644274:C:G	5	5	5	1	1	0
8:8644595:A:AT	NA	5	5	1	1	0
8:8646246:C:T	1d	4	5	1	1	0
8:8649881:C:T	7	4	5	1	1	0
8:8652889:A:G	6	4	5	1	1	0
8:8654057:A:G	7	4	5	1	1	0
8:8654527:C:T	3a	2	5	1	1	0
8:8658540:A:G	7	5	5	1	1	0
8:8660538:A:C	7	4	5	1	1	0
8:8661026:C:CA	NA	5	5	1	1	0
8:8661114:C:CA	NA	5	5	1	1	0
8:8661534:C:T	1f	4	5	1	1	0
8:8661681:C:G	7	4	5	1	1	0
8:8663215:C:T	5	5	5	1	1	0
8:8664622:A:G	1f	5	5	1	1	0
8:8664940:A:G	1f	5	5	1	1	0
8:8665147:A:G	1f	5	5	1	1	0
8:8665802:C:T	3a	5	5	1	1	0
8:8666916:C:T	4	2	5	1	1	0
8:8667444:C:T	7	5	5	1	1	0
8:8668486:A:G	1f	2	5	1	1	0
8:8668917:A:C	5	4	5	1	1	0
8:8669681:C:CGTAA	NA	2	5	1	1	0
8:8669681:C:T	NA	2	5	1	0	0
8:8670082:C:G	1f	4	5	1	1	0
8:8670177:A:T	5	4	5	1	1	0

uniqID	*RDB*	*minChrState*	*commonChrState*	*posMapFilt*	*eqtlMapFilt*	*ciMapFilt*
8:8670322:C:CT	NA	4	5	1	1	0
8:8670599:A:G	7	4	5	1	1	0
8:8670736:A:C	7	5	5	1	1	0
8:8671962:C:T	1f	4	5	1	1	0
8:8672429:C:G	7	4	5	1	1	0
8:8672579:A:G	7	4	5	1	1	0
8:8672952:A:C	7	4	5	1	1	0
8:8673320:C:T	6	4	5	1	1	0
8:8673601:A:C	5	4	5	1	1	0
8:8673736:C:T	6	4	5	1	1	0
8:8675176:A:G	5	1	5	1	1	0
8:8675325:A:T	5	5	5	1	1	0
8:8676626:G:GAATC	NA	5	5	1	1	0
8:8678530:A:G	NA	2	5	1	1	0
8:8679141:C:CTT	NA	2	5	1	1	0
8:8679176:A:G	6	2	5	1	1	0
8:8679614:C:T	4	2	5	1	1	0
8:8680477:A:G	5	4	5	1	1	0
8:8682192:A:T	4	4	5	1	1	0
8:8682878:C:T	7	4	5	1	1	0
8:8684953:A:G	NA	5	5	1	1	0
8:8685190:A:G	NA	5	5	1	1	0
8:8685646:C:T	1f	2	5	1	1	0
8:8685854:A:G	NA	2	15	1	1	0
8:8687054:C:G	7	5	15	1	1	0
8:8687325:A:G	6	5	15	1	1	0
8:8690787:C:T	7	5	15	1	1	0
8:8691622:A:T	7	5	15	1	1	0
8:8692477:C:T	6	5	15	1	1	0
8:8696449:G:T	5	5	15	1	1	0
8:8698977:C:CA	NA	5	15	1	1	0
8:8699757:A:T	7	1	5	1	1	0

uniqID	*RDB*	*minChrState*	*commonChrState*	*posMapFilt*	*eqtlMapFilt*	*ciMapFilt*
8:8699761:C:T	7	1	5	1	1	0
8:8700851:C:T	5	5	5	1	1	0
8:8702607:C:G	1f	2	5	1	1	0
8:8702827:A:G	1b	2	7	1	1	0
8:8702875:C:T	2b	2	7	1	1	0
8:8703781:G:GGA	NA	5	5	1	1	0
8:8704330:C:G	5	2	7	1	1	0
8:8706130:A:AT	NA	1	5	1	1	0
8:8706209:A:C	5	1	5	1	1	0
8:8706332:A:C	5	1	5	1	1	0
8:8707197:C:G	1f	2	5	1	1	0
8:8708974:C:G	5	1	7	1	1	0
8:8709756:C:G	5	5	5	1	1	0
8:8709971:C:T	7	5	5	1	1	0
8:8713038:C:T	6	4	5	1	1	0
8:8718850:A:G	6	4	5	1	1	0
8:8719513:A:G	4	2	5	1	1	0
8:8721473:A:G	6	4	5	1	1	0
8:8722675:C:T	NA	4	5	1	1	0
8:8723651:C:G	1f	4	5	1	1	0
8:8724257:C:T	NA	4	5	1	1	0
8:8724276:C:T	NA	4	5	1	1	0
8:8724415:C:T	5	4	5	1	1	0
8:8725126:G:T	3b	4	5	1	1	0
8:8725229:A:G	5	4	5	1	1	0
8:8725319:A:G	6	4	5	1	1	0
8:8726804:G:T	1d	1	5	1	1	0
8:8729761:C:G	1b	2	7	1	1	0
8:8730488:A:G	NA	2	7	1	1	0
8:8768895:A:G	NA	5	15	0	1	0
8:8769708:C:T	NA	5	15	0	1	0
8:8770512:C:T	NA	5	15	0	1	0

uniqID	*RDB*	*minChrState*	*commonChrState*	*posMapFilt*	*eqtlMapFilt*	*ciMapFilt*
8:8824858:G:T	7	5	15	0	1	0
8:9394053:G:T	7	5	15	0	1	0
8:9489417:A:G	6	5	15	1	1	0
8:9492426:G:T	7	5	15	1	1	0
8:9492453:C:T	7	5	15	1	1	0
8:9494732:G:T	5	1	15	1	1	0
8:9496118:A:T	6	5	15	1	1	0
8:9525325:C:T	7	5	15	1	1	0
8:9527707:A:G	5	5	15	1	1	0
8:9527863:A:T	6	5	15	1	1	0
8:9527869:A:G	6	5	15	1	1	0
8:9568369:A:G	6	4	5	1	0	0
8:9569104:C:T	6	4	5	1	1	0
8:9569109:C:T	6	4	5	1	0	0
8:9572099:A:G	5	4	5	1	0	0
8:9574830:G:T	7	4	5	1	0	0
8:9575445:A:G	6	4	5	1	0	0
8:9579144:C:CT	NA	4	5	1	0	0
8:9579318:C:T	7	4	5	1	0	0
8:9579377:C:CA	NA	4	5	1	0	0
8:9583872:C:T	6	4	5	1	0	0
8:9584598:C:G	6	4	5	1	0	0
8:9586062:A:T	6	1	5	1	0	0
8:9593309:A:C	5	4	4	1	0	0
8:10121635:A:G	5	5	15	1	1	0
8:10122423:A:G	7	5	15	1	1	0
8:10146490:C:CT	NA	5	15	1	1	0
8:10147398:C:T	7	5	15	1	1	0
8:10148447:A:G	5	5	15	1	1	0
8:10149212:A:C	5	5	15	1	1	0
8:10150070:C:G	5	5	15	1	1	0
8:10176506:A:T	5	5	15	1	1	0

uniqID	*RDB*	*minChrState*	*commonChrState*	*posMapFilt*	*eqtlMapFilt*	*ciMapFilt*
8:10758213:A:G	7	5	15	1	1	0
8:10810451:A:G	7	5	15	1	1	0
8:10811829:C:T	5	5	15	1	1	0
8:10812333:G:GT	NA	5	15	1	1	0
8:10813197:C:T	6	5	15	1	1	0
8:10813904:G:T	5	5	15	1	1	0
8:10815754:C:G	5	5	15	1	1	0
8:10816772:A:G	5	5	15	1	1	0
8:10817197:C:T	5	5	15	1	1	0
8:10818607:A:G	5	5	15	1	1	0
8:10818657:A:G	1f	5	15	1	1	0
8:10819854:C:T	5	5	15	1	1	0
8:10821056:C:CT	NA	5	15	1	1	0
8:10828909:C:T	7	5	15	1	1	0
8:10831868:G:T	2b	5	14	1	1	0
8:10835480:C:T	7	5	14	1	1	0
8:10835917:C:T	6	5	14	1	1	0
8:10836024:C:T	6	5	14	1	1	0
8:10836069:C:T	7	5	14	1	1	0
8:10836359:C:T	5	5	14	1	1	0
8:10836436:C:T	5	7	14	1	1	0
8:10836463:C:G	5	7	14	1	1	0
8:10836508:A:G	5	7	14	1	1	0
8:10837019:A:G	7	7	14	1	1	0
8:10837190:A:G	5	7	14	1	1	0
8:10837414:A:C	5	9	14	1	1	0
8:10837420:A:G	5	9	14	1	1	0
8:10837568:C:T	7	9	14	1	1	0
8:10837569:C:G	7	9	14	1	1	0
8:10839803:C:T	7	9	14	1	1	0
8:10841858:A:G	7	14	14	1	1	0
8:10842659:G:T	7	14	14	1	1	0

uniqID	*RDB*	*minChrState*	*commonChrState*	*posMapFilt*	*eqtlMapFilt*	*ciMapFilt*
8:10903475:A:T	7	5	15	1	1	0
8:10909193:A:C	5	5	14	1	1	0
8:10909936:C:T	5	5	14	1	1	0
8:10910066:C:T	5	5	14	1	1	0
8:10910343:C:G	2b	5	14	1	1	0
8:10926892:A:C	5	5	14	1	1	0
8:10927234:C:CAGTA	NA	5	14	1	1	0
8:10930069:A:C	5	5	15	1	1	0
8:10932203:C:G	6	5	15	1	1	0
8:10932695:C:G	5	5	15	1	1	0
8:10932868:A:G	1f	5	15	1	1	0
8:10933699:A:T	7	5	15	1	1	0
8:10935082:C:T	5	5	15	1	1	0
8:10935366:C:T	5	5	15	1	1	0
8:10935368:A:C	5	5	15	1	1	0
8:10935898:A:G	5	5	15	1	1	0
8:10936811:A:G	5	5	15	1	1	0
8:10936891:C:T	6	5	15	1	1	0
8:10938260:A:G	1f	5	15	1	1	0
8:10939273:G:T	1f	2	15	1	1	0
8:10939490:G:T	5	2	15	1	1	0
8:10943276:C:T	1f	5	15	1	1	0
8:10944809:G:T	6	5	15	1	1	0
8:10945439:A:G	5	5	15	1	1	0
8:10945767:C:CA	NA	5	15	1	1	0
8:10948422:C:CA	NA	7	15	1	1	0
8:10948968:A:G	6	7	15	1	1	0
8:10950396:C:G	4	1	15	1	1	0
8:10950757:C:G	7	5	15	1	1	0
8:10950866:C:T	5	5	15	1	1	0
8:10951175:G:GT	NA	5	15	1	1	0
8:10952500:A:T	7	5	15	1	1	0

uniqID	*RDB*	*minChrState*	*commonChrState*	*posMapFilt*	*eqtlMapFilt*	*ciMapFilt*
8:10953092:C:T	1f	5	15	1	1	0
8:10953874:A:G	5	5	15	1	1	0
8:10955225:C:G	6	5	15	1	1	0
8:10955383:G:GTT	NA	5	15	1	0	0
8:10955383:GT:GTT	NA	5	15	1	1	0
8:10957243:A:G	6	5	15	1	1	0
8:10958824:C:T	6	5	15	1	1	0
8:10960572:C:T	6	5	15	1	1	0
8:10961433:C:T	7	5	15	1	1	0
8:10962099:A:T	6	5	15	1	1	0
8:10962800:C:T	NA	5	15	1	1	0
8:10962929:C:G	2b	4	15	1	1	0
8:10963288:C:G	5	4	15	1	1	0
8:10964906:C:T	7	4	15	1	1	0
8:10964969:A:T	7	4	15	1	1	0
8:10968550:C:G	NA	5	15	1	1	0
8:10968926:A:G	6	4	15	1	1	0
8:10969075:C:T	6	4	15	1	1	0
8:10970773:A:T	7	5	15	1	1	0
8:10973149:C:CAA	NA	5	15	1	0	0
8:10973149:C:CA	NA	5	15	1	1	0
8:10974917:C:T	6	2	15	1	1	0
8:10975081:A:T	7	2	15	1	1	0
8:10975629:A:C	7	5	15	1	1	0
8:10975682:C:G	7	5	15	1	1	0
8:10975733:A:G	7	5	15	1	1	0
8:10976494:C:G	6	5	15	1	1	0
8:10976569:A:G	7	5	15	1	1	0
8:10976571:G:T	7	5	15	1	1	0
8:10978065:G:T	6	5	15	1	1	0
8:10979561:C:G	6	5	15	1	1	0
8:10979821:A:G	6	5	15	1	1	0

uniqID	*RDB*	*minChrState*	*commonChrState*	*posMapFilt*	*eqtlMapFilt*	*ciMapFilt*
8:10981003:A:C	5	2	15	1	1	0
8:10982051:A:G	4	1	15	1	1	0
8:10982410:C:G	5	1	15	1	1	0
8:10983534:A:G	5	5	15	1	1	0
8:10983579:G:T	6	5	15	1	1	0
8:10983921:C:G	6	5	15	1	1	0
8:10985140:A:G	5	5	15	1	1	0
8:10985432:C:T	6	5	15	1	1	0
8:10986837:G:T	1f	5	15	1	1	0
8:10986859:C:T	NA	5	15	1	1	0
8:10987199:C:T	NA	5	15	1	1	0
8:10987553:C:T	1b	5	15	1	1	0
8:10987651:G:T	5	5	15	1	1	0
8:10987967:A:G	6	5	15	1	1	0
8:10988138:A:C	6	5	15	1	1	0
8:10988275:A:G	5	5	15	1	1	0
8:10989057:A:G	6	5	15	1	1	0
8:10989206:C:T	7	5	15	1	1	0
8:10989521:A:G	5	5	15	1	1	0
8:10990164:A:G	7	5	15	1	1	0
8:10990371:G:GA	NA	5	15	1	1	0
8:10990672:C:T	4	5	15	1	1	0
8:10992252:C:G	7	5	15	1	1	0
8:10992544:A:AT	NA	5	15	1	1	0
8:10992605:A:C	7	5	15	1	1	0
8:10992883:C:T	7	5	15	1	1	0
8:10993904:C:T	7	5	15	1	1	0
8:10993995:C:T	6	5	15	1	1	0
8:10994743:C:G	4	5	15	1	1	0
8:10996089:A:G	7	5	15	1	1	0
8:11010974:G:T	7	5	15	1	1	0
8:11012977:C:T	7	5	15	1	1	0

uniqID	*RDB*	*minChrState*	*commonChrState*	*posMapFilt*	*eqtlMapFilt*	*ciMapFilt*
8:11013025:A:C	7	5	15	1	1	0
8:11014616:G:T	5	5	15	1	1	0
8:11015338:C:T	6	5	15	1	1	0
8:11016889:A:G	3a	2	15	1	1	0
8:11019578:C:G	5	1	15	1	1	0
8:11020313:G:T	5	2	15	1	1	0
8:11021682:A:G	4	2	15	1	1	0
8:11022106:C:G	6	2	15	1	1	0
8:11022185:C:T	6	2	15	1	1	0
8:11023655:C:T	7	5	15	1	1	0
8:11023997:C:G	7	5	15	1	1	0
8:11024275:C:T	7	5	15	1	1	0
8:11024326:A:C	6	5	15	1	1	0
8:11024663:A:C	7	5	15	1	1	0
8:11027491:T:TAA	NA	5	15	1	1	0
8:11029029:G:T	5	4	15	1	1	0
8:11029039:G:T	5	4	15	1	1	0
8:11030892:A:G	6	5	15	1	1	0
8:11030935:C:T	6	5	15	1	1	0
8:11031472:C:T	7	5	15	1	1	0
8:11032228:G:T	6	5	15	1	1	0
8:11032240:C:T	6	5	15	1	1	0
8:11033517:C:G	5	5	15	1	1	0
8:11033525:C:T	5	5	15	1	1	0
8:11033737:A:G	6	5	15	1	1	0
8:11033976:C:T	6	5	15	1	1	0
8:11034028:A:G	6	5	15	1	1	0
8:11034859:A:G	1d	5	15	1	1	0
8:11035071:G:GAGGTCATAATGGAAT	NA	5	15	1	1	0
8:11036052:T:TAA	NA	5	15	1	1	0
8:11036799:C:G	7	5	15	1	1	0
8:11036843:C:T	6	5	15	1	1	0

uniqID	*RDB*	*minChrState*	*commonChrState*	*posMapFilt*	*eqtlMapFilt*	*ciMapFilt*
8:11036919:A:G	5	5	15	1	1	0
8:11037034:A:G	1f	5	15	1	1	0
8:11037187:C:T	7	5	15	1	1	0
8:11037903:G:T	1f	5	15	1	1	0
8:11038244:A:T	7	5	15	1	1	0
8:11038885:A:T	7	5	15	1	1	0
8:11039159:C:G	7	5	15	1	1	0
8:11039816:G:T	6	4	15	1	1	0
8:11040216:C:T	6	4	15	1	1	0
8:11040647:A:G	6	4	15	1	1	0
8:11041642:A:T	7	5	15	1	1	0
8:11041661:C:T	7	5	15	1	1	0
8:11041897:T:TACACACACACACAC	NA	5	15	1	1	0
8:11042974:C:G	7	5	15	1	0	0
8:11042974:G:T	NA	5	15	1	1	0
8:11043138:C:T	5	5	15	1	1	0
8:11043236:C:T	5	5	15	1	1	0
8:11043926:C:T	5	5	15	1	1	0
8:11044689:A:G	5	5	15	1	1	0
8:11045161:A:G	6	2	15	1	1	0
8:11046209:C:T	5	5	15	1	1	0
8:11046394:C:G	6	5	15	1	1	0
8:11053922:A:G	6	2	15	1	1	0
8:11054097:A:C	5	2	15	1	1	0
8:11055597:A:C	7	1	15	1	1	0
8:11056175:A:G	1f	1	15	1	1	0
8:11056388:C:T	5	1	15	1	1	0
8:11060217:T:TA	NA	5	14	1	1	0
8:11060311:A:C	5	5	14	1	1	0
8:11061792:C:T	6	5	14	1	1	0
8:11062882:C:T	1f	5	14	1	1	0
8:11065003:A:C	7	5	13	1	1	0

uniqID	*RDB*	*minChrState*	*commonChrState*	*posMapFilt*	*eqtlMapFilt*	*ciMapFilt*
8:11069960:C:T	6	9	14	0	1	0
8:11070360:C:G	7	9	13	0	1	0
8:11071057:A:G	7	9	14	0	1	0
8:11072020:A:G	6	7	13	0	1	0
8:11073402:A:G	7	9	13	0	1	0
8:11073578:C:T	6	9	13	0	1	0
8:11074036:A:G	7	9	14	0	1	0
8:11074365:C:CTA	NA	13	14	0	1	0
8:11074812:C:T	7	13	14	0	1	0
8:11076635:G:T	6	13	14	0	1	0
8:11078781:A:C	6	13	14	0	1	0
8:11078949:A:G	6	13	14	0	1	0
8:11079367:A:G	7	12	14	0	1	0
8:11080014:C:T	6	13	14	0	1	0
8:11080665:A:T	5	13	14	0	1	0
8:11080675:C:T	5	13	14	0	1	0
8:11086942:C:T	1f	5	14	0	1	0
8:11087475:A:G	NA	6	13	0	1	0
8:11098992:A:G	4	9	14	0	1	0
8:11098996:C:T	4	9	14	0	1	0
8:11109269:C:T	6	2	14	0	1	0
8:11109303:C:G	6	2	14	0	1	0
8:11111462:A:T	6	5	14	0	1	0
8:11113089:A:G	7	5	14	0	1	0
8:11174484:C:G	6	4	4	1	1	0
8:11176403:A:G	6	4	5	1	1	0
8:11177126:A:C	7	4	4	1	1	0
8:11178093:A:C	7	4	4	1	1	0
8:11179458:G:T	NA	4	4	1	1	0
8:11182148:C:G	1f	4	5	1	1	0
8:11182455:A:G	7	4	5	1	1	0
8:11182704:A:G	5	4	5	1	1	0

uniqID	*RDB*	*minChrState*	*commonChrState*	*posMapFilt*	*eqtlMapFilt*	*ciMapFilt*
8:11183505:A:G	NA	4	5	1	1	0
8:11183765:C:CTTA	NA	4	5	1	1	0
8:11184390:A:C	6	4	5	1	1	0
8:11184396:A:G	6	4	5	1	1	0
8:11184478:C:CAA	NA	4	5	1	1	0
8:11184937:A:G	5	4	5	1	1	0
8:11185096:C:G	7	4	5	1	1	0
8:11185671:G:T	5	5	15	1	1	0
8:11185673:A:AT	NA	5	15	1	1	0
8:11186215:A:C	5	5	15	1	1	0
8:11186453:C:T	5	5	15	1	1	0
8:11186639:C:G	5	5	15	1	1	0
8:11186674:A:G	5	5	15	1	1	0
8:11187078:G:T	3a	4	15	1	1	0
8:11187434:C:T	6	4	15	1	1	0
8:11187651:A:G	5	5	15	1	1	0
8:11187675:C:T	5	5	15	1	1	0
8:11187770:C:G	5	5	15	1	1	0
8:11188532:C:G	5	5	15	1	1	0
8:11188540:A:G	5	5	15	1	1	0
8:11188586:C:T	5	5	15	1	1	0
8:11188752:A:G	5	5	15	1	1	0
8:11189488:C:T	1f	5	9	1	1	0
8:11189535:C:T	5	5	9	1	1	0
8:11190647:C:G	4	5	9	1	1	0
8:11191537:C:T	7	5	9	1	1	0
8:11192551:C:G	7	5	15	1	1	0
8:11192593:A:G	6	5	15	1	1	0
8:11193530:C:T	7	5	15	1	1	0
8:11193736:G:T	5	7	15	1	1	0
8:11194457:A:G	4	2	15	1	1	0
8:11194911:A:G	7	2	15	1	1	0

uniqID	*RDB*	*minChrState*	*commonChrState*	*posMapFilt*	*eqtlMapFilt*	*ciMapFilt*
8:11196295:C:G	6	5	15	1	1	0
8:11196970:C:T	7	5	15	1	1	0
8:11197301:C:G	7	5	15	1	1	0
8:11197323:G:T	7	5	15	1	1	0
8:11197598:C:T	7	5	15	1	1	0
8:11198579:C:CTTTT	NA	5	14	1	1	0
8:11198792:A:G	7	5	14	1	1	0
8:11199584:A:G	5	5	14	1	1	0
8:11199938:A:C	5	5	14	0	1	0
8:11200454:A:AAT	NA	5	14	0	1	0
8:11201504:G:T	7	5	14	0	1	0
8:11201605:A:ATT	NA	5	14	0	1	0
8:11202154:A:G	4	5	13	0	1	0
8:11202960:A:G	4	1	13	0	1	0
8:11203107:A:T	4	1	13	0	1	0
8:11204165:C:T	NA	1	13	0	1	0
8:11204184:A:G	5	1	13	0	1	0
8:11204503:C:G	4	1	13	0	1	0
8:11204532:A:G	4	1	13	0	1	0
8:11204755:C:T	4	1	11	0	1	0
8:11205593:C:T	2b	1	10	0	1	0
8:11205602:C:T	2a	1	11	0	1	0
8:11205654:A:T	2b	1	11	0	1	0
8:11205665:C:G	2b	1	11	0	1	0
8:11205817:T:TA	NA	1	11	0	1	0
8:11206220:C:G	3a	1	13	0	1	0
8:11206262:C:T	4	1	13	0	1	0
8:11206363:C:G	2b	1	13	0	1	0
8:11206543:A:G	2b	1	13	0	1	0
8:11206627:C:G	2b	1	13	0	1	0
8:11207326:A:C	5	2	13	0	1	0
8:11207367:A:C	5	2	13	0	1	0

uniqID	*RDB*	*minChrState*	*commonChrState*	*posMapFilt*	*eqtlMapFilt*	*ciMapFilt*
8:11207431:C:T	5	2	13	0	1	0
8:11207508:C:T	5	2	13	0	1	0
8:11207672:C:G	4	2	13	0	1	0
8:11208903:A:G	NA	2	14	0	1	0
8:11209499:C:T	NA	5	14	0	1	0
8:11210823:G:T	NA	5	14	0	1	0
8:11210824:G:T	7	5	14	0	1	0
8:11210828:C:T	7	5	14	0	1	0
8:11210983:A:G	6	5	14	0	1	0
8:11211068:C:T	6	5	14	0	1	0
8:11211302:G:T	NA	5	14	0	1	0
8:11212081:A:G	5	5	14	0	1	0
8:11212599:C:T	NA	5	14	0	1	0
8:11212650:A:AT	NA	5	14	0	1	0
8:11212778:C:T	5	5	14	0	1	0
8:11212811:A:C	NA	5	14	0	1	0
8:11212812:C:G	5	5	14	0	1	0
8:11212875:C:G	5	5	14	0	1	0
8:11213092:A:G	4	5	14	0	1	0
8:11213250:C:T	4	5	14	0	1	0
8:11213363:C:T	4	5	14	0	1	0
8:11213389:C:T	4	5	14	0	1	0
8:11213589:G:T	7	5	14	0	1	0
8:11213881:C:G	6	4	14	0	1	0
8:11214455:A:G	1f	4	14	0	1	0
8:11214972:A:G	1f	4	14	0	1	0
8:11215617:C:G	6	4	14	0	1	0
8:11215868:A:AT	NA	4	14	0	1	0
8:11216761:G:T	6	4	14	1	1	0
8:11217284:C:T	7	4	14	1	1	0
8:11217441:A:G	NA	4	14	1	1	0
8:11218893:A:G	5	2	14	1	1	0

uniqID	*RDB*	*minChrState*	*commonChrState*	*posMapFilt*	*eqtlMapFilt*	*ciMapFilt*
8:11219334:A:G	2b	2	14	1	1	0
8:11219386:G:T	4	2	14	1	1	0
8:11219781:C:T	6	2	14	1	1	0
8:11220846:C:CAA	NA	4	14	1	1	0
8:11221313:C:T	7	4	14	1	1	0
8:11223022:A:C	6	4	14	1	1	0
8:11223793:G:T	NA	4	14	1	1	0
8:11224313:C:T	NA	4	14	1	1	0
8:11225168:A:G	NA	4	14	1	1	0
8:11225480:C:T	1f	5	14	1	1	0
8:11225910:A:G	1f	5	14	1	1	0
8:11226071:A:G	5	5	14	1	1	0
8:11226456:A:C	6	5	14	1	1	0
8:11227104:A:G	5	5	14	1	1	0
8:11227406:A:G	NA	5	14	1	1	0
8:11227885:C:T	5	5	14	1	1	0
8:11228006:A:G	5	5	14	1	1	0
8:11228100:A:G	7	5	14	1	1	0
8:11228254:A:G	NA	5	14	1	1	0
8:11228672:A:G	6	5	14	1	1	0
8:11229319:C:G	5	5	14	1	1	0
8:11229638:C:T	NA	5	14	1	1	0
8:11229889:A:G	6	5	14	1	1	0
8:11230206:G:T	7	5	14	1	1	0
8:11230259:A:T	6	5	14	1	1	0
8:11230574:A:G	6	5	14	1	1	0
8:11231249:C:T	NA	5	14	1	1	0
8:11231354:G:T	7	5	14	1	1	0
8:11231886:A:G	6	5	14	1	1	0
8:11232343:C:G	7	5	14	1	1	0
8:11232788:A:G	7	5	14	1	1	0
8:11232860:A:G	6	5	14	1	1	0

uniqID	*RDB*	*minChrState*	*commonChrState*	*posMapFilt*	*eqtlMapFilt*	*ciMapFilt*
8:11233318:A:C	6	5	14	1	1	0
8:11233419:G:T	7	5	14	1	1	0
8:11233582:A:G	7	5	14	1	1	0
8:11233659:A:G	6	5	14	1	1	0
8:11233917:C:T	6	5	14	1	1	0
8:11233958:A:G	7	5	14	1	1	0
8:11234298:C:T	5	5	14	1	1	0
8:11234367:G:T	NA	5	14	1	1	0
8:11234500:A:T	1f	5	14	1	1	0
8:11234520:A:G	NA	5	14	1	1	0
8:11234613:A:T	7	5	14	1	1	0
8:11234626:C:T	6	5	14	1	1	0
8:11234780:C:G	6	5	14	1	1	0
8:11234844:A:G	6	5	14	1	1	0
8:11234885:C:G	6	5	14	1	1	0
8:11235136:C:G	7	5	14	1	1	0
8:11235150:A:C	6	5	14	1	1	0
8:11235360:A:C	NA	5	14	1	1	0
8:11235393:A:G	NA	5	14	1	1	0
8:11235497:A:G	NA	5	14	1	1	0
8:11235579:G:T	7	5	14	1	1	0
8:11235605:G:T	NA	5	14	1	1	0
8:11235614:A:G	NA	5	14	1	1	0
8:11235735:C:T	NA	5	14	1	1	0
8:11235910:A:G	NA	5	14	1	1	0
8:11236392:C:T	NA	5	14	1	1	0
8:11236413:C:T	7	5	14	1	1	0
8:11236419:C:T	7	5	14	1	1	0
8:11236572:A:T	6	5	14	1	1	0
8:11236681:G:T	7	5	14	1	1	0
8:11236685:C:T	7	5	14	1	1	0
8:11236809:C:T	6	5	14	1	1	0

uniqID	*RDB*	*minChrState*	*commonChrState*	*posMapFilt*	*eqtlMapFilt*	*ciMapFilt*
8:11236850:C:T	7	5	14	1	1	0
8:11236964:C:T	7	5	14	1	1	0
8:11236975:A:G	NA	5	14	1	1	0
8:11237330:C:G	6	5	14	1	1	0
8:11237477:A:G	NA	5	14	1	1	0
8:11237480:A:G	NA	5	14	1	1	0
8:11237587:C:CAAAA	NA	5	14	1	1	0
8:11237591:A:C	NA	5	14	1	1	0
8:11237756:A:C	NA	9	14	1	1	0
8:11237773:C:T	5	9	14	1	1	0
8:11238029:C:T	5	9	14	1	1	0
8:11238315:C:T	5	9	14	1	1	0
8:11238316:G:T	5	9	14	1	1	0
8:11238332:G:T	NA	9	14	1	1	0
8:11238587:A:G	NA	9	14	1	1	0
8:11238597:C:T	NA	9	14	1	1	0
8:11239017:A:T	5	13	14	1	1	0
8:11239054:C:T	5	13	14	1	1	0
8:11239078:G:T	5	13	14	1	1	0
8:11239137:A:G	NA	13	14	1	1	0
8:11239297:G:GATAG	NA	13	14	1	0	0
8:11239297:G:GATAT	NA	13	14	1	1	0
8:11239352:A:G	NA	13	14	1	1	0
8:11239510:A:G	NA	13	14	1	1	0
8:11239565:C:T	NA	13	14	1	1	0
8:11239640:C:T	3a	13	14	1	1	0
8:11239762:A:T	1f	13	14	1	1	0
8:11239942:C:CT	NA	13	14	1	1	0
8:11240571:C:T	7	13	14	1	1	0
8:11241935:G:GT	NA	13	14	1	1	0
8:11242025:G:T	6	13	14	1	1	0
8:11242039:T:TA	NA	13	14	1	1	0

uniqID	*RDB*	*minChrState*	*commonChrState*	*posMapFilt*	*eqtlMapFilt*	*ciMapFilt*
8:11242632:A:G	7	13	14	1	1	0
8:11243126:C:G	6	13	14	1	1	0
8:11244841:A:G	5	7	14	1	1	0
8:11245064:C:T	5	7	14	1	1	0
8:11245303:C:G	5	5	14	1	1	0
8:11245562:A:T	5	5	14	1	1	0
8:11247298:A:G	5	5	14	1	1	0
8:11247814:A:G	5	5	14	1	1	0
8:11248500:C:T	5	5	14	1	1	0
8:11248956:C:T	5	2	14	1	1	0
8:11249010:G:T	NA	5	14	1	1	0
8:11249261:C:T	5	5	14	1	1	0
8:11250848:A:C	4	7	14	1	1	0
8:11251175:A:G	6	7	14	1	1	0
8:11251705:A:G	NA	7	14	1	1	0
8:11252170:C:T	1f	5	14	1	1	0
8:11252425:A:C	7	5	14	1	1	0
8:11309192:C:T	3a	5	15	1	1	0
8:11336781:A:G	3a	5	15	1	1	0
8:11338146:A:G	4	2	15	1	1	0
8:11355602:C:G	1f	2	15	1	1	0
8:11358156:C:T	7	5	15	1	1	0
8:11361261:C:G	6	3	15	1	1	0
8:11361850:G:GAGGA	NA	5	15	1	1	0
8:11362275:A:C	5	5	15	1	1	0
8:11362277:G:T	5	5	15	1	1	0
8:11382367:A:G	NA	1	14	1	1	0
8:11384556:C:T	NA	5	14	1	1	0
8:11392093:A:C	NA	2	14	1	1	0
8:11393764:A:G	NA	5	14	1	1	0
8:11395079:A:G	NA	2	15	1	1	0
8:11396856:A:C	1d	2	15	1	1	0

uniqID	*RDB*	*minChrState*	*commonChrState*	*posMapFilt*	*eqtlMapFilt*	*ciMapFilt*
8:11396874:A:G	4	2	15	1	1	0
8:11397073:A:C	NA	2	15	1	1	0
8:11397086:A:T	5	2	15	1	1	0
8:11397457:C:G	5	2	15	1	1	0
8:11398865:A:G	6	5	15	1	1	0
8:11398953:C:T	NA	5	15	1	1	0
8:11399484:A:T	7	5	15	1	1	0
8:11400628:C:G	5	5	15	1	1	0
8:11400680:A:G	NA	5	15	1	1	0
8:11400944:G:T	NA	5	15	1	1	0
8:11401116:A:G	5	5	15	1	1	0
8:11402347:A:G	6	5	15	1	1	0
8:11410513:A:C	3a	4	14	1	1	0
8:11411005:C:G	2b	5	14	1	1	0
8:11415184:C:G	3a	2	14	1	1	0
8:11415572:A:G	3a	2	14	1	1	0
8:11415597:C:T	2a	2	14	1	1	0
8:11415794:A:T	1f	2	14	1	1	0
8:11415812:C:T	1f	2	14	1	1	0
8:11416171:C:T	1d	2	13	1	1	0
8:11416428:C:T	4	2	14	1	1	0
8:11416635:A:G	2b	2	14	1	1	0
8:11416885:C:T	4	2	14	1	1	0
8:11417016:A:ACT	NA	2	14	1	1	0
8:11417144:C:T	2b	2	14	1	1	0
8:11417150:G:T	2b	2	14	1	1	0
8:11417257:A:G	4	4	14	1	1	0
8:11417493:C:T	7	4	14	1	1	0
8:11417582:C:T	5	4	14	1	1	0
8:11418385:A:G	4	4	13	1	1	0
8:11418773:C:T	NA	4	13	1	1	0
8:11419335:A:AT	NA	4	14	1	1	0

uniqID	*RDB*	*minChrState*	*commonChrState*	*posMapFilt*	*eqtlMapFilt*	*ciMapFilt*
8:11419852:C:T	2b	4	13	1	1	0
8:11419861:G:T	2b	4	13	1	1	0
8:11420104:C:G	4	4	13	1	1	0
8:11420221:A:G	4	4	13	1	1	0
8:11420295:C:T	4	4	13	1	1	0
8:11421016:A:G	4	5	13	1	1	0
8:11421358:A:C	2b	1	13	1	1	0
8:11421384:C:T	2b	1	13	1	1	0
8:11421793:C:T	NA	1	11	1	1	0
8:11422045:A:G	2c	1	11	1	1	0
8:11422130:C:T	4	1	11	1	1	0
8:11422170:A:G	4	1	11	1	1	0
8:11422289:C:G	2b	1	10	1	1	0
8:11422442:A:C	4	1	11	1	1	0
8:11422491:A:C	3a	1	11	1	1	0
8:11422492:C:G	3a	1	11	1	1	0
8:11422494:A:AT	NA	1	11	1	1	0
8:11422521:G:GA	NA	1	11	1	1	0
8:11422861:C:G	1b	2	13	1	1	0
8:11422936:C:T	3a	2	13	1	1	0
8:11423072:A:G	4	5	13	1	1	0
8:11423083:A:C	4	5	13	1	1	0
8:11423142:A:G	4	5	13	1	1	0
8:11423434:A:G	3a	5	13	1	1	0
8:11423537:A:G	2b	5	13	1	1	0
8:11423781:C:G	4	5	13	1	1	0
8:11425077:C:T	5	2	14	1	1	0
8:11425081:G:T	5	2	14	1	1	0
8:11425105:G:T	5	2	14	1	1	0
8:11425809:G:T	6	5	14	1	1	0
8:11426400:G:T	3a	5	14	1	1	0
8:11426790:C:G	1f	5	14	1	1	0

uniqID	*RDB*	*minChrState*	*commonChrState*	*posMapFilt*	*eqtlMapFilt*	*ciMapFilt*
8:11427133:G:T	3a	5	14	1	1	0
8:11427341:G:T	1f	5	14	1	1	0
8:11427637:A:T	1f	5	14	1	1	0
8:11428395:C:T	5	5	14	1	1	0
8:11430485:A:G	4	2	14	1	1	0
8:11430990:C:T	1d	2	14	1	1	0
8:11431558:T:TAC	NA	5	14	1	1	0
8:11431943:T:TAA	NA	5	14	1	1	0
8:11432085:A:ACG	NA	5	14	1	1	0
8:11432085:A:ACACG	NA	5	14	1	0	0
8:11432438:C:G	5	5	14	0	1	0
8:11432453:C:T	5	5	14	0	1	0
8:11432946:A:C	7	5	14	0	1	0
8:11433780:C:T	5	5	14	0	1	0
8:11433909:C:T	5	5	14	0	1	0
8:11434176:A:T	5	5	14	0	1	0
8:11434232:C:T	5	5	14	0	1	0
8:11434415:C:CTCGGTTT	NA	5	14	0	1	0
8:11434792:C:G	5	5	14	0	1	0
8:11434929:C:T	5	5	14	0	1	0
8:11435049:A:G	5	5	14	0	1	0
8:11435291:G:GTGGC	NA	5	14	0	1	0
8:11435516:C:T	5	5	14	0	1	0
8:11435564:C:T	5	5	14	0	1	0
8:11435927:C:G	5	5	14	0	1	0
8:11438064:G:T	5	5	14	0	1	0
8:11439225:A:G	5	5	14	0	1	0
8:11440019:A:G	7	5	14	0	1	0
8:11444516:A:G	5	5	14	0	1	0
8:11444837:C:T	5	5	14	0	1	0
8:11446421:A:G	5	5	14	0	1	0
8:11446637:A:G	5	5	14	0	1	0

uniqID	*RDB*	*minChrState*	*commonChrState*	*posMapFilt*	*eqtlMapFilt*	*ciMapFilt*
8:11446652:A:G	5	5	14	0	1	0
8:11446680:C:T	5	5	14	0	1	0
8:11446800:C:T	5	5	14	0	1	0
8:11446868:C:T	2b	2	14	0	1	0
8:11446955:A:G	4	2	14	0	1	0
8:11447093:A:G	1b	1	14	0	1	0
8:11447119:C:T	4	1	14	0	1	0
8:11447679:C:G	4	2	14	0	1	0
8:11448659:C:G	5	5	14	0	1	0
8:11450133:A:G	5	5	14	0	1	0
8:11450422:A:G	NA	5	14	0	1	0
8:11450587:G:T	5	5	14	0	1	0
8:11460909:T:TA	NA	5	14	0	1	0
8:11461111:A:G	6	5	14	0	1	0
8:11466745:A:T	6	5	14	0	1	0
8:11467557:C:G	6	5	14	0	1	0
12:62830952:C:T	NA	5	15	0	1	0
12:62831343:C:T	6	5	15	0	1	0
12:62837767:A:G	7	9	15	0	1	0
12:62847085:C:G	5	14	15	0	1	0
12:62848152:A:G	6	9	15	0	1	0
12:62849418:A:G	6	9	15	0	1	0
12:62851080:A:AC	NA	14	15	1	1	0
12:62852271:A:G	7	14	15	1	1	0
12:62852916:A:G	4	9	15	1	1	0
12:62855388:C:T	NA	5	15	1	1	0
12:62858342:A:G	6	5	15	1	1	0
12:62858561:C:T	6	5	15	1	1	0
12:62859241:A:AC	NA	1	15	1	1	0
12:62861935:T:TA	NA	1	1	1	1	0
12:62862739:G:T	5	1	7	1	1	0
12:62865152:C:T	7	4	5	1	1	0

uniqID	*RDB*	*minChrState*	*commonChrState*	*posMapFilt*	*eqtlMapFilt*	*ciMapFilt*
12:62865291:A:G	6	4	5	1	1	0
12:62868455:C:T	6	4	5	1	1	0
12:62868499:C:T	6	4	5	1	1	0
12:62868500:A:C	6	4	5	1	1	0
12:62870574:C:G	6	4	5	1	1	0
12:62875839:C:T	4	1	5	1	1	0
12:62880276:C:G	7	4	5	1	1	0
12:62884714:A:ATTT	NA	4	5	1	1	0
12:62884972:A:G	7	4	5	1	1	0
12:62886001:A:T	7	5	5	1	1	0
12:62886372:C:G	7	5	5	1	1	0
12:62886649:C:T	6	5	5	1	1	0
12:62888219:A:C	7	4	5	1	1	0
12:62889402:A:G	7	4	5	1	1	0
12:62889409:A:G	7	4	5	1	1	0
12:62890491:A:C	6	4	5	1	1	0
12:62891828:C:CT	NA	4	5	1	1	0
12:62894058:A:G	5	4	5	1	1	0
12:62897930:A:G	6	4	5	1	1	0
12:62898111:A:G	6	4	5	1	1	0
12:62898463:C:CT	NA	4	5	1	1	0
12:62898490:C:T	5	4	5	1	1	0
12:62902420:C:T	6	4	4	1	1	0
12:62903639:A:G	5	4	5	1	1	0
12:62903655:G:GT	NA	4	5	1	1	0
12:62903793:A:G	6	4	5	1	1	0
12:62904252:C:T	6	4	5	1	1	0
12:62905380:C:CA	NA	4	5	1	1	0
12:62905772:A:AC	NA	4	5	1	1	0
12:62906488:A:G	6	4	5	1	1	0
12:62910714:A:T	6	4	5	1	1	0
12:62914776:T:TA	NA	4	5	1	1	0

uniqID	*RDB*	*minChrState*	*commonChrState*	*posMapFilt*	*eqtlMapFilt*	*ciMapFilt*
12:62917704:A:G	5	4	5	1	1	0
12:62920860:C:G	6	4	5	1	1	0
12:62921257:C:T	7	4	5	1	1	0
12:62922143:A:G	7	4	5	1	1	0
12:62926398:A:G	5	4	5	1	1	0
12:62928006:T:TA	NA	4	5	1	1	0
12:62928633:A:G	7	4	5	1	1	0
12:62930621:A:G	7	4	5	1	1	0
12:62930798:C:T	5	4	5	1	1	0
12:62931846:A:ATTTGT	NA	4	4	1	1	0
12:62932816:A:G	6	4	5	1	1	0
12:62935154:C:CA	NA	4	5	1	1	0
12:62935705:C:T	7	4	5	1	1	0
12:62937348:G:T	5	4	5	1	1	0
12:62937532:C:T	7	4	4	1	0	0
12:62939055:C:T	7	4	5	1	1	0
12:62941426:C:T	6	4	4	1	1	0
12:62941444:A:G	7	4	4	1	1	0
12:62941837:A:G	NA	4	4	1	1	0
12:62941929:A:T	7	4	4	1	1	0
12:62942258:C:T	7	4	5	1	1	0
12:62945158:G:GGAAACAACCTGACGAT	NA	4	5	1	1	0
12:62945245:A:G	6	4	5	1	1	0
12:62945970:C:G	7	4	5	1	1	0
12:62948736:A:G	7	4	4	1	1	0
12:62949110:A:C	6	4	4	1	1	0
12:62950556:C:G	5	4	4	1	1	0
12:62952696:C:T	7	4	5	1	1	0
12:62952910:C:T	7	4	5	1	1	0
12:62957906:C:G	7	4	5	1	1	0
12:62960880:A:G	7	4	4	1	1	0
12:62961299:C:T	7	4	4	1	1	0

uniqID	*RDB*	*minChrState*	*commonChrState*	*posMapFilt*	*eqtlMapFilt*	*ciMapFilt*
12:62961801:A:G	7	4	5	1	1	0
12:62964552:C:T	7	4	5	1	1	0
12:62968280:C:T	7	4	5	1	1	0
12:62968740:C:CA	NA	4	5	1	1	0
12:62970482:A:G	3b	4	5	1	1	0
12:62972210:G:GT	NA	4	5	1	1	0
12:62975307:C:T	7	4	5	1	1	0
12:62977162:A:G	7	4	5	1	1	0
12:62977163:A:G	7	4	5	1	1	0
12:62977175:A:AG	NA	4	5	1	1	0
12:62980580:A:T	7	4	5	1	1	0
12:62982152:C:CT	NA	4	5	1	1	0
12:62985871:A:C	6	4	5	1	1	0
12:62986620:A:G	7	4	5	1	1	0
12:62988288:A:G	6	4	5	1	1	0
12:62989110:A:C	7	4	5	1	0	0
12:62990403:A:G	6	5	5	1	0	0
12:62990415:C:T	6	5	5	1	0	0
12:62990871:A:C	6	5	5	1	1	0
12:62992896:G:GA	NA	5	5	1	1	0
12:62993793:A:G	4	5	5	1	1	0
12:62995269:A:G	3a	1	5	1	1	0
12:62995340:A:G	3a	1	5	1	1	0
12:62995984:C:T	4	1	2	1	1	0
12:62996061:A:G	4	1	1	1	1	0
12:62997180:C:T	4	1	1	1	1	0
12:62999154:C:G	6	1	1	1	1	0
12:63001068:A:AT	NA	1	15	1	1	0
13:83298475:A:G	6	5	15	0	0	0
13:83299269:C:T	6	5	15	0	0	0
13:83300084:A:G	6	5	15	0	0	0
13:83300138:A:AGAGAGC	NA	5	15	0	0	0

uniqID	*RDB*	*minChrState*	*commonChrState*	*posMapFilt*	*eqtlMapFilt*	*ciMapFilt*
13:83300269:C:T	7	5	15	0	0	0
13:83300359:A:G	7	5	15	0	0	0
13:83302594:C:T	7	5	15	0	0	0
13:83302795:A:T	5	5	15	0	0	0
13:83304274:C:T	7	5	15	0	0	0
13:83304898:G:T	7	5	15	0	0	0
13:83306586:A:G	7	5	15	0	0	0
13:83311141:A:G	7	9	15	0	0	0
13:83312341:C:T	6	9	15	0	0	0
13:83312717:C:G	7	9	15	0	0	0
16:13021889:C:T	NA	5	15	1	0	0
16:13022033:A:AT	NA	5	15	1	0	0
16:13023207:C:T	7	5	15	1	0	0
16:13023388:A:G	NA	5	15	1	0	0
16:13023394:A:G	6	5	15	1	0	0
16:13024150:A:G	NA	5	15	1	0	0
16:13025315:G:T	7	5	15	1	0	0
16:13026502:A:G	NA	5	15	1	0	0
16:13029711:G:GA	NA	5	15	1	0	0
16:13030222:A:G	NA	5	15	1	0	0
16:13030875:A:G	NA	5	15	1	0	0
16:13031195:A:C	NA	5	15	1	0	0
16:13032351:C:G	7	5	15	1	0	0
16:13032547:A:G	NA	5	15	1	0	0
16:13032863:A:T	5	5	15	1	0	0
16:13035206:T:TA	NA	5	15	1	0	0
16:13036811:C:T	5	5	15	1	0	0
16:13037305:C:T	7	5	15	1	0	0
16:13038054:G:T	7	5	15	1	0	0
16:13038196:A:G	7	5	15	1	0	0
16:13038723:G:T	7	5	15	1	0	0
16:13039154:C:T	7	5	15	1	0	0

uniqID	*RDB*	*minChrState*	*commonChrState*	*posMapFilt*	*eqtlMapFilt*	*ciMapFilt*
16:13039642:A:T	6	5	15	1	0	0
16:13039646:C:T	6	5	15	1	0	0
16:13040514:C:T	5	5	15	1	0	0
16:13040889:C:G	6	5	15	1	0	0
16:13041027:G:T	7	5	15	1	0	0
16:13041921:C:T	5	5	15	1	0	0
16:13041924:A:G	5	5	15	1	0	0
16:13041940:A:C	5	5	15	1	0	0
16:13042097:A:G	7	5	15	1	0	0
16:13043452:A:G	6	5	15	1	0	0
16:13044033:C:T	7	5	15	1	0	0
16:13044827:C:T	6	5	15	1	0	0
16:13045117:C:CA	NA	5	15	1	0	0
16:13047022:C:G	7	5	15	1	0	0
16:13047598:C:G	7	5	15	1	0	0
16:13047734:C:T	7	5	15	1	0	0
16:13048395:C:T	6	5	15	1	0	0
16:13048841:C:T	7	5	15	1	0	0
16:13048916:G:GT	NA	5	15	1	0	0
16:13049490:T:TA	NA	5	15	1	0	0
16:13049557:C:T	7	5	15	1	0	0
16:13049749:A:C	6	5	15	1	0	0
16:13049906:A:T	6	5	15	1	0	0
16:13050340:A:C	6	5	15	1	0	0
16:13051686:A:C	5	5	15	1	0	0
16:13052050:A:G	6	5	15	1	0	0
16:13052714:C:G	6	5	15	1	0	0
16:13052846:A:G	7	5	15	1	0	0
16:13053072:A:G	5	5	15	1	0	0
16:13053136:G:T	5	5	15	1	0	0
16:13053140:C:G	5	5	15	1	0	0
16:13053187:C:T	6	5	15	1	0	0

uniqID	*RDB*	*minChrState*	*commonChrState*	*posMapFilt*	*eqtlMapFilt*	*ciMapFilt*
16:13053270:A:G	7	5	15	1	0	0
16:13053457:A:AC	NA	5	15	1	0	0
16:13053576:A:T	7	5	15	1	0	0
16:13053882:A:G	7	5	15	1	0	0
16:13053924:C:G	7	5	15	1	0	0
16:13053951:C:T	6	5	15	1	0	0
16:13054037:C:G	6	5	15	1	0	0
16:13054265:A:G	5	5	15	1	0	0
16:13054645:C:T	7	5	15	1	0	0
16:13054883:C:G	6	5	15	1	0	0
16:13054897:A:C	7	5	15	1	0	0
16:13055220:C:T	7	5	15	1	0	0
16:13055621:T:TCC	NA	5	15	1	0	0
16:13055766:C:T	7	5	15	1	0	0
16:13057200:A:G	7	5	15	1	0	0
16:13058241:G:GT	NA	5	15	1	0	0
16:13059539:C:T	5	5	15	1	0	0
16:13061109:A:G	7	5	15	1	0	0
16:13062232:A:G	7	5	15	1	0	0
16:13066833:C:T	7	5	15	1	0	0
16:13070238:A:G	7	5	15	1	0	0
16:13070671:C:T	5	5	15	1	0	0
16:13070809:C:G	5	5	15	1	0	0
16:13075745:A:G	7	5	15	1	0	0
16:13077351:C:T	7	5	15	1	0	0
16:13077901:A:G	7	5	15	1	0	0
16:13077915:A:G	6	5	15	1	0	0
16:13078044:A:T	5	5	15	1	0	0
16:13078807:C:T	NA	5	15	1	0	0
16:13079063:C:CTT	NA	5	15	1	0	0
16:13079082:A:C	NA	5	15	1	0	0
16:13079214:A:G	NA	5	15	1	0	0

uniqID	*RDB*	*minChrState*	*commonChrState*	*posMapFilt*	*eqtlMapFilt*	*ciMapFilt*
16:13079389:G:T	7	5	15	1	0	0
16:13079463:C:T	NA	5	15	1	0	0
16:13079698:A:T	7	5	15	1	0	0
16:13080252:A:G	6	5	15	1	0	0
16:13080515:C:T	7	5	15	1	0	0
16:13081923:C:G	7	5	15	1	0	0
16:13082445:A:G	6	5	15	1	0	0
16:13083055:G:T	6	5	15	1	0	0
16:13083068:G:GT	NA	5	15	1	0	0
16:13086810:C:T	7	5	15	1	0	0
16:13088905:C:T	6	5	15	1	0	0
16:13089059:A:G	7	5	15	1	0	0
16:13089854:G:T	5	5	15	1	0	0
16:13091332:C:T	6	5	15	1	0	0
16:13092100:A:G	6	7	15	1	0	0
16:13092220:C:T	5	7	15	1	0	0
16:13092663:C:T	5	7	15	1	0	0
16:13093774:G:T	3a	7	15	1	0	0
16:13093778:C:T	3a	7	15	1	0	0
16:13093858:C:T	5	7	15	1	0	0
16:13094769:G:T	5	7	15	1	0	0
16:13094897:C:G	6	7	15	1	0	0
16:13095142:C:G	7	7	15	1	0	0
16:13095171:C:T	7	7	15	1	0	0
16:13095296:A:T	5	5	15	1	0	0
16:13095739:A:C	6	5	15	1	0	0
16:13096300:C:T	5	5	15	1	0	0
16:13097084:G:T	7	5	15	1	0	0
16:13097125:A:G	7	5	15	1	0	0
16:13097206:C:G	7	5	15	1	0	0
16:13097746:G:T	6	5	15	1	0	0
16:13097749:G:T	6	5	15	1	0	0

uniqID	*RDB*	*minChrState*	*commonChrState*	*posMapFilt*	*eqtlMapFilt*	*ciMapFilt*
16:13098440:C:G	7	5	15	1	0	0
16:13098508:A:G	7	5	15	1	0	0
16:13098762:A:C	7	5	15	1	0	0
16:13098934:A:G	6	5	15	1	0	0
16:13099114:C:G	7	5	15	1	0	0
16:13099177:G:T	7	5	15	1	0	0
16:13099919:A:G	3a	5	15	1	0	0
16:13099953:G:T	5	5	15	1	0	0
16:13100021:C:T	5	5	15	1	0	0
16:13101555:C:T	NA	5	15	1	0	0
16:13101618:A:G	6	5	15	1	0	0
16:13102532:C:T	6	5	15	1	0	0
16:13102906:A:T	6	5	15	1	0	0
16:13104924:C:T	6	5	15	1	0	0
16:13105091:A:C	6	5	15	1	0	0
16:13105863:C:T	6	5	15	1	0	0
16:13105998:C:T	6	5	15	1	0	0
16:13109594:G:T	5	5	15	1	0	0
16:13112335:C:T	5	5	15	1	0	0
16:13115702:C:G	7	2	15	1	0	0
16:13115755:A:G	7	2	15	1	0	0
16:13116871:T:TTTTA	NA	5	15	1	0	0
16:13118299:A:T	5	7	15	1	0	0

Abbreviations: chr = chromosome; pos = posoletion score; RDB = RegulomeDB score; minChrState = minimum 15-core chromatin state across 127 tissue/cell type; commonChrStat[illegible]

Table S7: MAGMA gene mapping

GENE	*CHR*	*START*	*STOP*	*NSNPS*	*NPARAM*	*N*	*ZSTAT*	*P*	*SYMBOL*	*P_bonferroni*
ENSG00000175806	8	9911778	10286401	2323	113	875864	66.651	1.32E-07	*MSRA*	2.51E-07
ENSG00000171044	8	10753555	11058875	1537	60	869954	61.473	3.94E-06	*XKR6*	7.49E-06
ENSG00000147324	8	8640864	8751155	729	52	877684	60.589	6.85E-06	*MFHAS1*	1.30E-05
ENSG00000184608	8	11225911	11296167	484	55	850654	59.693	1.19E-05	*C8orf12*	2.26E-05
ENSG00000061987	12	62860597	62991363	455	43	865430	55.472	1.45E-04	*MON2*	2.76E-04
ENSG00000221949	12	62995531	62997214	9	4	900877	53.823	3.68E-05	*C12orf61*	6.98E-04
ENSG00000215346	8	10983980	10987745	16	5	874968	53.664	4.02E-04	*AF131215.5*	7.63E-04
ENSG00000237515	16	12995477	13334272	1900	140	872998	53.093	5.50E-04	*SHISA9*	1.05E-03
ENSG00000177710	8	11188397	11189717	12	3	799029	51.892	1.06E-04	*SLC35G5*	2.01E-03
ENSG00000070778	14	88932122	89021077	304	23	870589	51.008	1.69E-03	*PTPN21*	3.21E-03
ENSG00000136573	8	11351510	11422113	543	49	852722	49.869	3.07E-03	*BLK*	5.83E-03
ENSG00000075568	2	98372799	98612388	726	37	867258	48.755	5.43E-03	*TMEM131*	1.03E-02
ENSG00000042317	14	88851268	88936694	202	47	859970	48.013	7.88E-07	*SPATA7*	1.50E-02
ENSG00000108465	17	46045176	46059140	52	12	896719	46.487	1.67E-02	*CDK5RAP3*	3.17E-02
ENSG00000168959	11	88237744	88799113	2445	66	873387	46.475	1.68E-02	*GRM5*	3.19E-02
ENSG00000142599	1	8412457	8877702	1077	50	864581	46.276	1.85E-02	*RERE*	3.51E-02
ENSG00000104626	8	8859657	8974256	734	60	850658	4.556	2.61E-02	*ERI1*	4.95E-02

Ordered by P_bonferroni significance. Abbreviations: CHR = chromosome; NSNPS = number of SNPs; NPARAM = number of relevant parameters used in the model; P_bonferroni = Bonferroni corrected P-value

Table S8: Single-nucleus enrichment analysis for tinnitus.

Trait	*Annotation*	*Prop._SNPs*	*Prop._h2*	*Prop._h2_std_error*	*Enrichment*	*Enrichment_std_error*	*Enrichment_p*	*Coefficient*	*Coefficient_std_error*	*Coefficient_z-score*	*P*	*P.fdr*
tinnitus2025	Amygdala_excitatory	0.152876982	0.226933756	0.018628355	1.484420689	0.12185193	7.86194763606336e-05	8.73159351300842e-10	3.06456350272491e-10	2.849212785	0.002191378	0.067359432
tinnitus2025	MGE_interneuron	0.133352424	0.20643354	0.019022266	1.54802991	0.142646569	8.60740760156949e-05	8.50648951986393e-10	3.42623889705163e-10	2.482748511	0.006518655	0.067359432
tinnitus2025	Midbrain_derived_inhibitory	0.13006464	0.201374314	0.016355034	1.548263343	0.125745429	8.62941460440933e-05	8.34221873439008e-10	3.23035282643954e-10	2.582448167	0.004905105	0.067359432
tinnitus2025	Eccentric_medium_spiny_neuron	0.139940572	0.20515623	0.01815926	1.466023947	0.129764085	0.000213199	6.42559821359025e-10	3.06740308519855e-10	2.094800727	0.018094355	0.112185002
tinnitus2025	Hippocampal_dentate_gyrus	0.1334826	0.189416125	0.016196102	1.419032334	0.121334933	0.000510472	6.07295698735022e-10	2.88720309253778e-10	2.10340485	0.017715197	0.112185002
tinnitus2025	Miscellaneous	0.145742967	0.201388924	0.017041337	1.381808867	0.116927338	0.001436709	5.84280155246167e-10	2.94682588074343e-10	1.982744074	0.023698012	0.122439728
tinnitus2025	LAMP5_LHX6_and_Chandelier	0.133661592	0.196115253	0.018213756	1.467252117	0.136267688	0.00057977	6.2314564516445e-10	3.34911167625412e-10	1.86062964	0.031398248	0.139049384
tinnitus2025	Astrocyte	0.119646196	0.183241491	0.015927672	1.531527923	0.133123094	9.16051423005103e-05	5.34353848750774e-10	3.15158660020065e-10	1.695507427	0.044989601	0.154964182
tinnitus2025	Medium_spiny_neuron	0.131058574	0.182163183	0.015746081	1.389937161	0.12014537	0.002132079	5.06548064909016e-10	2.97349587433754e-10	1.703543863	0.044233168	0.154964182
tinnitus2025	Upper_layer_intratelencephalic	0.126680231	0.175263693	0.016602202	1.38351258	0.131055982	0.003676245	4.32739207340791e-10	3.22394858784372e-10	1.342264604	0.089755108	0.278240835
tinnitus2025	Hippocampal_CA4	0.138661123	0.183798225	0.014645432	1.325520965	0.105620316	0.00203606	3.28624632300328e-10	2.6449295689142e-10	1.242470258	0.107031554	0.301634379
tinnitus2025	Cerebellar_inhibitory	0.115840896	0.160505287	0.016690673	1.385566695	0.144082734	0.009614818	3.34375568417634e-10	3.44752709403102e-10	0.969899755	0.166048231	0.372101437
tinnitus2025	Deep_layer_corticothalamic_and_6b	0.141697445	0.181523622	0.016447884	1.281064891	0.116077489	0.013701036	2.75609061663093e-10	2.86028426859115e-10	0.963572274	0.167630207	0.372101437
tinnitus2025	Hippocampal_CA1_3	0.139934868	0.178039223	0.016510035	1.27230064	0.11798371	0.021309584	2.80044257454926e-10	2.9113160923844e-10	0.961916359	0.16804581	0.372101437
tinnitus2025	Mammillary_body	0.141411091	0.182643502	0.017893079	1.291578332	0.126532358	0.023329793	2.82458754035369e-10	3.18370430276857e-10	0.887201597	0.187485187	0.387469385
tinnitus2025	Deep_layer_intratelencephalic	0.135613729	0.17685359	0.016823364	1.304097978	0.124053544	0.014261492	2.29448498299912e-10	3.09360213046942e-10	0.741687162	0.22913845	0.417840703
tinnitus2025	Upper_rhombic_lip	0.104736009	0.149273135	0.015620991	1.425232231	0.149146326	0.006187972	2.83112648041351e-10	3.65240199761581e-10	0.775140985	0.219128178	0.417840703
tinnitus2025	Deep_layer_near_projecting	0.131748373	0.168179081	0.015591548	1.276517327	0.118343379	0.021039361	1.96576858016448e-10	2.87037193790924e-10	0.684848035	0.24671991	0.424906512
tinnitus2025	Oligodendrocyte_precursor	0.143097341	0.172339174	0.014307525	1.204349243	0.099984559	0.043498078	7.64804405948756e-11	2.61690145704117e-10	0.292255715	0.385045558	0.628232226
tinnitus2025	Lower_rhombic_lip	0.120869784	0.149469194	0.015820239	1.236613394	0.130886632	0.079018281	1.59630439044502e-11	3.12780272316336e-10	0.051035968	0.47964843	0.743455066
tinnitus2025	Bergmann_glia	0.116902602	0.153482412	0.016296613	1.312908428	0.139403335	0.024575051	-2.71E-11	3.37494132912577e-10	-0.080412318	0.532045335	0.785400256
tinnitus2025	Thalamic_excitatory	0.133378761	0.156492148	0.017858811	1.173291365	0.133895463	0.189909026	-5.40E-11	3.28699487996094e-10	-0.164231775	0.565225656	0.796454334
tinnitus2025	CGE_interneuron	0.123059123	0.145215638	0.016633898	1.180047724	0.13516997	0.172606268	-1.23E-10	3.29454379671213e-10	-0.372618951	0.645283967	0.869730564
tinnitus2025	Choroid_plexus	0.121924445	0.130736593	0.015152612	1.07227549	0.124278703	0.562623669	-4.93E-10	3.08999315606108e-10	-1.593859947	0.944516296	0.999589736
tinnitus2025	Committed_oligodendrocyte_precursor	0.117432533	0.122365846	0.014506513	1.042009766	0.123530611	0.734815925	-6.79E-10	3.16893016589491e-10	-2.141600599	0.983887182	0.999589736
tinnitus2025	Ependymal	0.112796689	0.12145964	0.014470329	1.07680146	0.128286828	0.542067433	-5.19E-10	3.12780106795032e-10	-1.658742045	0.951416108	0.999589736
tinnitus2025	Fibroblast	0.120058868	0.144637461	0.015377403	1.204721185	0.12808219	0.112802896	-2.78E-10	3.27464391651808e-10	-0.848327127	0.801872093	0.999589736
tinnitus2025	Microglia	0.103532551	0.109572369	0.015050966	1.058337375	0.145374244	0.689845312	-5.16E-10	3.54025771725018e-10	-1.45777074	0.927548127	0.999589736
tinnitus2025	Oligodendrocyte	0.110614899	0.096718483	0.014372231	0.874371213	0.12993034	0.335209172	-1.08E-09	3.2277791926713e-10	-3.34577556	0.999589736	0.999589736
tinnitus2025	Splatter	0.121207973	0.13225441	0.014221623	1.09113622	0.117332407	0.437488372	-3.29E-10	2.76407686696471e-10	-1.191680124	0.883306653	0.999589736
tinnitus2025	Vascular	0.108495177	0.104928711	0.015499894	0.967127884	0.142862515	0.81796989	-9.64E-10	3.71333541520922e-10	-2.597051178	0.995298604	0.999589736

Abbreviation: Prop = probability; h2 = heritability coefficient

Table S9: LD score regression genetic correlation analysis

Trait1	*Trait2*	*PMID*	*Rg*	*Rg_se*	*Pval*	*Pval_FDR*	*is_sig*
Tinnitus	Hearing loss	35580588	0.5008	0.0296	2.64E-64	3.43E-63	yes
Tinnitus	Major Depressive Disorder	30718901	0.3249	0.0281	5.70E-31	3.71E-30	yes
Tinnitus	Neuroticism	29942085	0.2624	0.0239	4.93E-28	2.14E-27	yes
Tinnitus	Anxiety Disorder	31748690	0.3671	0.044	6.87E-17	2.23E-16	yes
Tinnitus	Insomnia	30804565	0.1908	0.0339	1.80E-08	4.68E-08	yes
Tinnitus	Vertigo	34620984	0.2196	0.0513	1.85E-05	4.01E-05	yes
Tinnitus	Chronic Pain	31194737	0.2962	0.0931	0.0015	2.79E-03	yes
Tinnitus	Post-traumatic stress syndrome	31594949	0.2359	0.0778	0.0024	3.90E-03	yes
Tinnitus	Schizophrenia	35396580	0.0676	0.0294	0.0215	3.11E-02	yes
Tinnitus	Bipolar disorder	34002096	0.0798	0.0367	0.0297	3.86E-02	yes
Tinnitus	Alzheimer's disease	35379992	0.0485	0.0527	0.3577	4.23E-01	no
Tinnitus	Type 2 Diabetes	35551307	0.0242	0.0329	0.4616	5.00E-01	no
Tinnitus	Cerebellar volume	35842455	0.0272	0.051	0.5942	5.94E-01	no

Abbreviations: PMID = pubmed ID; Rg = correlation coefficient; se = standard error; Pval = p-value; FDR = false discovery rate

Table S10: Single nucleus enrichment analysis on hearing loss, major depressive disorder, insomnia, neuroticism, sc

Trait	*Supercluster*	*dataset_name*	*Supercluster_ID*
bip2021	Amygdala excitatory	bip2021	Amygdala_excitatory
bip2021	Astrocyte	bip2021	Astrocyte
bip2021	Bergmann glia	bip2021	Bergmann_glia
bip2021	Cerebellar inhibitory	bip2021	Cerebellar_inhibitory
bip2021	CGE interneuron	bip2021	CGE_interneuron
bip2021	Choroid plexus	bip2021	Choroid_plexus
bip2021	Committed oligodendrocyte precursor	bip2021	Committed_oligodendrocyte_precursor
bip2021	Deep-layer corticothalamic and 6b	bip2021	Deep_layer_corticothalamic_and_6b
bip2021	Deep-layer intratelencephalic	bip2021	Deep_layer_intratelencephalic
bip2021	Deep-layer near-projecting	bip2021	Deep_layer_near_projecting
bip2021	Eccentric medium spiny neuron	bip2021	Eccentric_medium_spiny_neuron
bip2021	Ependymal	bip2021	Ependymal
bip2021	Fibroblast	bip2021	Fibroblast
bip2021	Hippocampal CA1-3	bip2021	Hippocampal_CA1_3
bip2021	Hippocampal CA4	bip2021	Hippocampal_CA4
bip2021	Hippocampal dentate gyrus	bip2021	Hippocampal_dentate_gyrus
bip2021	LAMP5-LHX6 and Chandelier	bip2021	LAMP5_LHX6_and_Chandelier
bip2021	Lower rhombic lip	bip2021	Lower_rhombic_lip
bip2021	Mammillary body	bip2021	Mammillary_body
bip2021	Medium spiny neuron	bip2021	Medium_spiny_neuron
bip2021	MGE interneuron	bip2021	MGE_interneuron
bip2021	Microglia	bip2021	Microglia
bip2021	Midbrain-derived inhibitory	bip2021	Midbrain_derived_inhibitory
bip2021	Miscellaneous	bip2021	Miscellaneous
bip2021	Oligodendrocyte precursor	bip2021	Oligodendrocyte_precursor
bip2021	Oligodendrocyte	bip2021	Oligodendrocyte
bip2021	Splatter	bip2021	Splatter
bip2021	Thalamic excitatory	bip2021	Thalamic_excitatory
bip2021	Upper-layer intratelencephalic	bip2021	Upper_layer_intratelencephalic
bip2021	Upper rhombic lip	bip2021	Upper_rhombic_lip

Trait	***Supercluster***	***dataset_name***	***Supercluster_ID***
bip2021	Vascular	bip2021	Vascular
hearing_loss	Amygdala excitatory	hearing_loss	Amygdala_excitatory
hearing_loss	Astrocyte	hearing_loss	Astrocyte
hearing_loss	Bergmann glia	hearing_loss	Bergmann_glia
hearing_loss	Cerebellar inhibitory	hearing_loss	Cerebellar_inhibitory
hearing_loss	CGE interneuron	hearing_loss	CGE_interneuron
hearing_loss	Choroid plexus	hearing_loss	Choroid_plexus
hearing_loss	Committed oligodendrocyte precursor	hearing_loss	Committed_oligodendrocyte_precursor
hearing_loss	Deep-layer corticothalamic and 6b	hearing_loss	Deep_layer_corticothalamic_and_6b
hearing_loss	Deep-layer intratelencephalic	hearing_loss	Deep_layer_intratelencephalic
hearing_loss	Deep-layer near-projecting	hearing_loss	Deep_layer_near_projecting
hearing_loss	Eccentric medium spiny neuron	hearing_loss	Eccentric_medium_spiny_neuron
hearing_loss	Ependymal	hearing_loss	Ependymal
hearing_loss	Fibroblast	hearing_loss	Fibroblast
hearing_loss	Hippocampal CA1-3	hearing_loss	Hippocampal_CA1_3
hearing_loss	Hippocampal CA4	hearing_loss	Hippocampal_CA4
hearing_loss	Hippocampal dentate gyrus	hearing_loss	Hippocampal_dentate_gyrus
hearing_loss	LAMP5-LHX6 and Chandelier	hearing_loss	LAMP5_LHX6_and_Chandelier
hearing_loss	Lower rhombic lip	hearing_loss	Lower_rhombic_lip
hearing_loss	Mammillary body	hearing_loss	Mammillary_body
hearing_loss	Medium spiny neuron	hearing_loss	Medium_spiny_neuron
hearing_loss	MGE interneuron	hearing_loss	MGE_interneuron
hearing_loss	Microglia	hearing_loss	Microglia
hearing_loss	Midbrain-derived inhibitory	hearing_loss	Midbrain_derived_inhibitory
hearing_loss	Miscellaneous	hearing_loss	Miscellaneous
hearing_loss	Oligodendrocyte precursor	hearing_loss	Oligodendrocyte_precursor
hearing_loss	Oligodendrocyte	hearing_loss	Oligodendrocyte
hearing_loss	Splatter	hearing_loss	Splatter
hearing_loss	Thalamic excitatory	hearing_loss	Thalamic_excitatory
hearing_loss	Upper-layer intratelencephalic	hearing_loss	Upper_layer_intratelencephalic
hearing_loss	Upper rhombic lip	hearing_loss	Upper_rhombic_lip

Trait	*Supercluster*	*dataset_name*	*Supercluster_ID*
hearing_loss	Vascular	hearing_loss	Vascular
insomnia	Amygdala excitatory	insomnia	Amygdala_excitatory
insomnia	Astrocyte	insomnia	Astrocyte
insomnia	Bergmann glia	insomnia	Bergmann_glia
insomnia	Cerebellar inhibitory	insomnia	Cerebellar_inhibitory
insomnia	CGE interneuron	insomnia	CGE_interneuron
insomnia	Choroid plexus	insomnia	Choroid_plexus
insomnia	Committed oligodendrocyte precursor	insomnia	Committed_oligodendrocyte_precursor
insomnia	Deep-layer corticothalamic and 6b	insomnia	Deep_layer_corticothalamic_and_6b
insomnia	Deep-layer intratelencephalic	insomnia	Deep_layer_intratelencephalic
insomnia	Deep-layer near-projecting	insomnia	Deep_layer_near_projecting
insomnia	Eccentric medium spiny neuron	insomnia	Eccentric_medium_spiny_neuron
insomnia	Ependymal	insomnia	Ependymal
insomnia	Fibroblast	insomnia	Fibroblast
insomnia	Hippocampal CA1-3	insomnia	Hippocampal_CA1_3
insomnia	Hippocampal CA4	insomnia	Hippocampal_CA4
insomnia	Hippocampal dentate gyrus	insomnia	Hippocampal_dentate_gyrus
insomnia	LAMP5-LHX6 and Chandelier	insomnia	LAMP5_LHX6_and_Chandelier
insomnia	Lower rhombic lip	insomnia	Lower_rhombic_lip
insomnia	Mammillary body	insomnia	Mammillary_body
insomnia	Medium spiny neuron	insomnia	Medium_spiny_neuron
insomnia	MGE interneuron	insomnia	MGE_interneuron
insomnia	Microglia	insomnia	Microglia
insomnia	Midbrain-derived inhibitory	insomnia	Midbrain_derived_inhibitory
insomnia	Miscellaneous	insomnia	Miscellaneous
insomnia	Oligodendrocyte precursor	insomnia	Oligodendrocyte_precursor
insomnia	Oligodendrocyte	insomnia	Oligodendrocyte
insomnia	Splatter	insomnia	Splatter
insomnia	Thalamic excitatory	insomnia	Thalamic_excitatory
insomnia	Upper-layer intratelencephalic	insomnia	Upper_layer_intratelencephalic
insomnia	Upper rhombic lip	insomnia	Upper_rhombic_lip

Trait	*Supercluster*	*dataset_name*	*Supercluster_ID*
insomnia	Vascular	insomnia	Vascular
mdd2019	Amygdala excitatory	mdd2019	Amygdala_excitatory
mdd2019	Astrocyte	mdd2019	Astrocyte
mdd2019	Bergmann glia	mdd2019	Bergmann_glia
mdd2019	Cerebellar inhibitory	mdd2019	Cerebellar_inhibitory
mdd2019	CGE interneuron	mdd2019	CGE_interneuron
mdd2019	Choroid plexus	mdd2019	Choroid_plexus
mdd2019	Committed oligodendrocyte precursor	mdd2019	Committed_oligodendrocyte_precursor
mdd2019	Deep-layer corticothalamic and 6b	mdd2019	Deep_layer_corticothalamic_and_6b
mdd2019	Deep-layer intratelencephalic	mdd2019	Deep_layer_intratelencephalic
mdd2019	Deep-layer near-projecting	mdd2019	Deep_layer_near_projecting
mdd2019	Eccentric medium spiny neuron	mdd2019	Eccentric_medium_spiny_neuron
mdd2019	Ependymal	mdd2019	Ependymal
mdd2019	Fibroblast	mdd2019	Fibroblast
mdd2019	Hippocampal CA1-3	mdd2019	Hippocampal_CA1_3
mdd2019	Hippocampal CA4	mdd2019	Hippocampal_CA4
mdd2019	Hippocampal dentate gyrus	mdd2019	Hippocampal_dentate_gyrus
mdd2019	LAMP5-LHX6 and Chandelier	mdd2019	LAMP5_LHX6_and_Chandelier
mdd2019	Lower rhombic lip	mdd2019	Lower_rhombic_lip
mdd2019	Mammillary body	mdd2019	Mammillary_body
mdd2019	Medium spiny neuron	mdd2019	Medium_spiny_neuron
mdd2019	MGE interneuron	mdd2019	MGE_interneuron
mdd2019	Microglia	mdd2019	Microglia
mdd2019	Midbrain-derived inhibitory	mdd2019	Midbrain_derived_inhibitory
mdd2019	Miscellaneous	mdd2019	Miscellaneous
mdd2019	Oligodendrocyte precursor	mdd2019	Oligodendrocyte_precursor
mdd2019	Oligodendrocyte	mdd2019	Oligodendrocyte
mdd2019	Splatter	mdd2019	Splatter
mdd2019	Thalamic excitatory	mdd2019	Thalamic_excitatory
mdd2019	Upper-layer intratelencephalic	mdd2019	Upper_layer_intratelencephalic
mdd2019	Upper rhombic lip	mdd2019	Upper_rhombic_lip

Trait	*Supercluster*	*dataset_name*	*Supercluster_ID*
mdd2019	Vascular	mdd2019	Vascular
neuroticism	Amygdala excitatory	neuroticism	Amygdala_excitatory
neuroticism	Astrocyte	neuroticism	Astrocyte
neuroticism	Bergmann glia	neuroticism	Bergmann_glia
neuroticism	Cerebellar inhibitory	neuroticism	Cerebellar_inhibitory
neuroticism	CGE interneuron	neuroticism	CGE_interneuron
neuroticism	Choroid plexus	neuroticism	Choroid_plexus
neuroticism	Committed oligodendrocyte precursor	neuroticism	Committed_oligodendrocyte_precursor
neuroticism	Deep-layer corticothalamic and 6b	neuroticism	Deep_layer_corticothalamic_and_6b
neuroticism	Deep-layer intratelencephalic	neuroticism	Deep_layer_intratelencephalic
neuroticism	Deep-layer near-projecting	neuroticism	Deep_layer_near_projecting
neuroticism	Eccentric medium spiny neuron	neuroticism	Eccentric_medium_spiny_neuron
neuroticism	Ependymal	neuroticism	Ependymal
neuroticism	Fibroblast	neuroticism	Fibroblast
neuroticism	Hippocampal CA1-3	neuroticism	Hippocampal_CA1_3
neuroticism	Hippocampal CA4	neuroticism	Hippocampal_CA4
neuroticism	Hippocampal dentate gyrus	neuroticism	Hippocampal_dentate_gyrus
neuroticism	LAMP5-LHX6 and Chandelier	neuroticism	LAMP5_LHX6_and_Chandelier
neuroticism	Lower rhombic lip	neuroticism	Lower_rhombic_lip
neuroticism	Mammillary body	neuroticism	Mammillary_body
neuroticism	Medium spiny neuron	neuroticism	Medium_spiny_neuron
neuroticism	MGE interneuron	neuroticism	MGE_interneuron
neuroticism	Microglia	neuroticism	Microglia
neuroticism	Midbrain-derived inhibitory	neuroticism	Midbrain_derived_inhibitory
neuroticism	Miscellaneous	neuroticism	Miscellaneous
neuroticism	Oligodendrocyte precursor	neuroticism	Oligodendrocyte_precursor
neuroticism	Oligodendrocyte	neuroticism	Oligodendrocyte
neuroticism	Splatter	neuroticism	Splatter
neuroticism	Thalamic excitatory	neuroticism	Thalamic_excitatory
neuroticism	Upper-layer intratelencephalic	neuroticism	Upper_layer_intratelencephalic
neuroticism	Upper rhombic lip	neuroticism	Upper_rhombic_lip

Trait	*Supercluster*	*dataset_name*	*Supercluster_ID*
neuroticism	Vascular	neuroticism	Vascular
scz2022	Amygdala excitatory	scz2022_eur	Amygdala_excitatory
scz2022	Astrocyte	scz2022_eur	Astrocyte
scz2022	Bergmann glia	scz2022_eur	Bergmann_glia
scz2022	Cerebellar inhibitory	scz2022_eur	Cerebellar_inhibitory
scz2022	CGE interneuron	scz2022_eur	CGE_interneuron
scz2022	Choroid plexus	scz2022_eur	Choroid_plexus
scz2022	Committed oligodendrocyte precursor	scz2022_eur	Committed_oligodendrocyte_precursor
scz2022	Deep-layer corticothalamic and 6b	scz2022_eur	Deep_layer_corticothalamic_and_6b
scz2022	Deep-layer intratelencephalic	scz2022_eur	Deep_layer_intratelencephalic
scz2022	Deep-layer near-projecting	scz2022_eur	Deep_layer_near_projecting
scz2022	Eccentric medium spiny neuron	scz2022_eur	Eccentric_medium_spiny_neuron
scz2022	Ependymal	scz2022_eur	Ependymal
scz2022	Fibroblast	scz2022_eur	Fibroblast
scz2022	Hippocampal CA1-3	scz2022_eur	Hippocampal_CA1_3
scz2022	Hippocampal CA4	scz2022_eur	Hippocampal_CA4
scz2022	Hippocampal dentate gyrus	scz2022_eur	Hippocampal_dentate_gyrus
scz2022	LAMP5-LHX6 and Chandelier	scz2022_eur	LAMP5_LHX6_and_Chandelier
scz2022	Lower rhombic lip	scz2022_eur	Lower_rhombic_lip
scz2022	Mammillary body	scz2022_eur	Mammillary_body
scz2022	Medium spiny neuron	scz2022_eur	Medium_spiny_neuron
scz2022	MGE interneuron	scz2022_eur	MGE_interneuron
scz2022	Microglia	scz2022_eur	Microglia
scz2022	Midbrain-derived inhibitory	scz2022_eur	Midbrain_derived_inhibitory
scz2022	Miscellaneous	scz2022_eur	Miscellaneous
scz2022	Oligodendrocyte precursor	scz2022_eur	Oligodendrocyte_precursor
scz2022	Oligodendrocyte	scz2022_eur	Oligodendrocyte
scz2022	Splatter	scz2022_eur	Splatter
scz2022	Thalamic excitatory	scz2022_eur	Thalamic_excitatory
scz2022	Upper-layer intratelencephalic	scz2022_eur	Upper_layer_intratelencephalic
scz2022	Upper rhombic lip	scz2022_eur	Upper_rhombic_lip

Trait	***Supercluster***	***dataset_name***	***Supercluster_ID***
scz2022	Vascular	scz2022_eur	Vascular

Classified by disease and alphabetically sorted by cluster region. Abbreviation: P = p value; P.fdr = FDR adjusted P value Y

Table S10: Single nucleus enrichment analysis on hearchizophrenia and bipolar disorder

Trait	*Supercluster*	*disorder_type*	*P*	*P.fdr*	*BETA*	*SE*	*Enrichment*
bip2021	Amygdala excitatory	psychiatric	1.51E-04	1.32E-03	4.41E-09	1.22E-09	1.477
bip2021	Astrocyte	psychiatric	9.87E-01	1.00E+00	-2.44E-09	1.09E-09	1.095
bip2021	Bergmann glia	psychiatric	7.88E-01	1.00E+00	-9.02E-10	1.13E-09	1.210
bip2021	Cerebellar inhibitory	psychiatric	4.48E-03	1.39E-02	2.81E-09	1.07E-09	1.441
bip2021	CGE interneuron	psychiatric	6.33E-04	2.80E-03	3.81E-09	1.18E-09	1.498
bip2021	Choroid plexus	psychiatric	9.92E-01	1.00E+00	-2.48E-09	1.03E-09	1.036
bip2021	Committed oligodendrocyte precursor	psychiatric	9.42E-01	1.00E+00	-1.86E-09	1.18E-09	1.137
bip2021	Deep-layer corticothalamic and 6b	psychiatric	2.11E-02	4.37E-02	2.18E-09	1.08E-09	1.326
bip2021	Deep-layer intratelencephalic	psychiatric	2.33E-06	7.23E-05	5.57E-09	1.22E-09	1.618
bip2021	Deep-layer near-projecting	psychiatric	1.63E-02	3.89E-02	2.33E-09	1.09E-09	1.356
bip2021	Eccentric medium spiny neuron	psychiatric	1.26E-04	1.32E-03	3.96E-09	1.08E-09	1.495
bip2021	Ependymal	psychiatric	1.00E+00	1.00E+00	-3.44E-09	1.03E-09	0.971
bip2021	Fibroblast	psychiatric	1.00E+00	1.00E+00	-3.58E-09	1.08E-09	0.994
bip2021	Hippocampal CA1-3	psychiatric	5.02E-04	2.59E-03	3.87E-09	1.18E-09	1.452
bip2021	Hippocampal CA4	psychiatric	8.16E-04	3.16E-03	3.42E-09	1.09E-09	1.438
bip2021	Hippocampal dentate gyrus	psychiatric	2.77E-04	1.72E-03	4.02E-09	1.16E-09	1.475
bip2021	LAMP5-LHX6 and Chandelier	psychiatric	1.39E-02	3.60E-02	2.22E-09	1.01E-09	1.369
bip2021	Lower rhombic lip	psychiatric	9.38E-01	1.00E+00	-1.57E-09	1.02E-09	1.067
bip2021	Mammillary body	psychiatric	6.81E-01	1.00E+00	-4.51E-10	9.62E-10	1.119
bip2021	Medium spiny neuron	psychiatric	1.77E-02	3.91E-02	2.30E-09	1.09E-09	1.350
bip2021	MGE interneuron	psychiatric	1.21E-03	4.18E-03	3.39E-09	1.12E-09	1.455
bip2021	Microglia	psychiatric	9.43E-01	1.00E+00	-2.03E-09	1.29E-09	1.087
bip2021	Midbrain-derived inhibitory	psychiatric	1.30E-01	2.36E-01	1.27E-09	1.12E-09	1.280
bip2021	Miscellaneous	psychiatric	6.95E-03	1.96E-02	3.05E-09	1.24E-09	1.385
bip2021	Oligodendrocyte precursor	psychiatric	9.84E-01	1.00E+00	-2.36E-09	1.10E-09	1.074
bip2021	Oligodendrocyte	psychiatric	8.96E-01	1.00E+00	-1.19E-09	9.41E-10	1.065
bip2021	Splatter	psychiatric	9.79E-01	1.00E+00	-2.05E-09	1.01E-09	1.021
bip2021	Thalamic excitatory	psychiatric	7.12E-02	1.38E-01	1.67E-09	1.14E-09	1.294
bip2021	Upper-layer intratelencephalic	psychiatric	1.92E-01	3.30E-01	1.07E-09	1.23E-09	1.345
bip2021	Upper rhombic lip	psychiatric	1.71E-04	1.32E-03	4.46E-09	1.24E-09	1.541

Trait	*Supercluster*	*disorder_type*	*P*	*P.fdr*	*BETA*	*SE*	*Enrichment*
bip2021	Vascular	psychiatric	1.00E+00	1.00E+00	-4.54E-09	1.21E-09	0.934
hearing_loss	Amygdala excitatory	neurological	1.33E-02	2.53E-01	1.38E-09	6.23E-10	1.363
hearing_loss	Astrocyte	neurological	8.42E-01	9.00E-01	-5.53E-10	5.52E-10	1.250
hearing_loss	Bergmann glia	neurological	4.16E-01	6.30E-01	1.42E-10	6.67E-10	1.383
hearing_loss	Cerebellar inhibitory	neurological	9.03E-01	9.03E-01	-6.63E-10	5.11E-10	1.106
hearing_loss	CGE interneuron	neurological	1.52E-01	4.02E-01	5.30E-10	5.15E-10	1.317
hearing_loss	Choroid plexus	neurological	8.05E-01	9.00E-01	-5.17E-10	6.02E-10	1.203
hearing_loss	Committed oligodendrocyte precursor	neurological	4.29E-01	6.30E-01	1.03E-10	5.77E-10	1.392
hearing_loss	Deep-layer corticothalamic and 6b	neurological	1.17E-01	4.02E-01	6.43E-10	5.41E-10	1.261
hearing_loss	Deep-layer intratelencephalic	neurological	2.56E-02	2.53E-01	1.36E-09	6.97E-10	1.447
hearing_loss	Deep-layer near-projecting	neurological	3.27E-02	2.53E-01	1.09E-09	5.92E-10	1.372
hearing_loss	Eccentric medium spiny neuron	neurological	2.00E-01	4.14E-01	4.18E-10	4.97E-10	1.249
hearing_loss	Ependymal	neurological	4.47E-01	6.30E-01	7.33E-11	5.55E-10	1.310
hearing_loss	Fibroblast	neurological	1.46E-01	4.02E-01	7.97E-10	7.57E-10	1.548
hearing_loss	Hippocampal CA1-3	neurological	1.89E-02	2.53E-01	1.23E-09	5.94E-10	1.359
hearing_loss	Hippocampal CA4	neurological	5.27E-01	7.10E-01	-3.11E-11	4.67E-10	1.150
hearing_loss	Hippocampal dentate gyrus	neurological	3.42E-01	6.24E-01	2.55E-10	6.25E-10	1.196
hearing_loss	LAMP5-LHX6 and Chandelier	neurological	4.79E-02	2.54E-01	8.76E-10	5.26E-10	1.360
hearing_loss	Lower rhombic lip	neurological	4.19E-01	6.30E-01	9.68E-11	4.75E-10	1.217
hearing_loss	Mammillary body	neurological	1.45E-01	4.02E-01	5.77E-10	5.46E-10	1.240
hearing_loss	Medium spiny neuron	neurological	7.41E-02	3.28E-01	7.54E-10	5.21E-10	1.311
hearing_loss	MGE interneuron	neurological	1.56E-01	4.02E-01	5.87E-10	5.79E-10	1.305
hearing_loss	Microglia	neurological	8.75E-01	9.03E-01	-6.71E-10	5.82E-10	1.247
hearing_loss	Midbrain-derived inhibitory	neurological	1.77E-01	4.14E-01	5.19E-10	5.59E-10	1.279
hearing_loss	Miscellaneous	neurological	2.45E-01	4.74E-01	3.56E-10	5.15E-10	1.199
hearing_loss	Oligodendrocyte precursor	neurological	8.23E-01	9.00E-01	-5.63E-10	6.08E-10	1.224
hearing_loss	Oligodendrocyte	neurological	7.07E-01	8.77E-01	-2.43E-10	4.45E-10	1.123
hearing_loss	Splatter	neurological	1.91E-01	4.14E-01	4.89E-10	5.60E-10	1.282
hearing_loss	Thalamic excitatory	neurological	6.65E-01	8.59E-01	-2.18E-10	5.10E-10	1.101
hearing_loss	Upper-layer intratelencephalic	neurological	3.97E-01	6.30E-01	1.53E-10	5.85E-10	1.345
hearing_loss	Upper rhombic lip	neurological	4.92E-02	2.54E-01	8.84E-10	5.35E-10	1.367

Trait	*Supercluster*	*disorder_type*	*P*	*P.fdr*	*BETA*	*SE*	*Enrichment*
hearing_loss	Vascular	neurological	7.57E-01	9.00E-01	-5.13E-10	7.36E-10	1.353
insomnia	Amygdala excitatory	brain traits	1.82E-03	4.23E-02	2.15E-09	7.40E-10	1.330
insomnia	Astrocyte	brain traits	9.58E-01	9.88E-01	-1.60E-09	9.27E-10	0.979
insomnia	Bergmann glia	brain traits	6.31E-01	9.12E-01	-2.81E-10	8.41E-10	1.150
insomnia	Cerebellar inhibitory	brain traits	2.39E-01	4.89E-01	6.20E-10	8.74E-10	1.208
insomnia	CGE interneuron	brain traits	2.73E-03	4.23E-02	2.45E-09	8.82E-10	1.425
insomnia	Choroid plexus	brain traits	9.31E-01	9.88E-01	-1.41E-09	9.46E-10	0.937
insomnia	Committed oligodendrocyte precursor	brain traits	6.83E-01	9.21E-01	-3.96E-10	8.31E-10	1.097
insomnia	Deep-layer corticothalamic and 6b	brain traits	1.44E-01	4.89E-01	8.60E-10	8.11E-10	1.189
insomnia	Deep-layer intratelencephalic	brain traits	8.40E-03	6.51E-02	2.03E-09	8.49E-10	1.355
insomnia	Deep-layer near-projecting	brain traits	2.78E-02	1.23E-01	1.61E-09	8.39E-10	1.299
insomnia	Eccentric medium spiny neuron	brain traits	2.52E-01	4.89E-01	5.65E-10	8.46E-10	1.183
insomnia	Ependymal	brain traits	6.47E-01	9.12E-01	-3.09E-10	8.18E-10	1.076
insomnia	Fibroblast	brain traits	9.88E-01	9.88E-01	-1.83E-09	8.13E-10	0.908
insomnia	Hippocampal CA1-3	brain traits	1.66E-01	4.89E-01	7.26E-10	7.48E-10	1.161
insomnia	Hippocampal CA4	brain traits	1.90E-01	4.89E-01	6.70E-10	7.63E-10	1.194
insomnia	Hippocampal dentate gyrus	brain traits	4.32E-01	7.04E-01	1.21E-10	7.02E-10	1.100
insomnia	LAMP5-LHX6 and Chandelier	brain traits	2.20E-02	1.13E-01	1.65E-09	8.19E-10	1.335
insomnia	Lower rhombic lip	brain traits	2.31E-01	4.89E-01	6.33E-10	8.60E-10	1.183
insomnia	Mammillary body	brain traits	8.38E-01	9.88E-01	-7.38E-10	7.50E-10	1.010
insomnia	Medium spiny neuron	brain traits	3.84E-01	6.61E-01	2.25E-10	7.65E-10	1.108
insomnia	MGE interneuron	brain traits	1.25E-02	7.73E-02	1.78E-09	7.92E-10	1.354
insomnia	Microglia	brain traits	8.27E-01	9.88E-01	-8.75E-10	9.27E-10	0.965
insomnia	Midbrain-derived inhibitory	brain traits	1.21E-01	4.67E-01	9.73E-10	8.30E-10	1.250
insomnia	Miscellaneous	brain traits	5.35E-01	8.29E-01	-6.56E-11	7.52E-10	1.067
insomnia	Oligodendrocyte precursor	brain traits	9.77E-01	9.88E-01	-1.67E-09	8.38E-10	0.940
insomnia	Oligodendrocyte	brain traits	8.08E-01	9.88E-01	-6.80E-10	7.82E-10	1.022
insomnia	Splatter	brain traits	3.29E-01	6.00E-01	3.64E-10	8.24E-10	1.154
insomnia	Thalamic excitatory	brain traits	1.86E-01	4.89E-01	7.74E-10	8.67E-10	1.192
insomnia	Upper-layer intratelencephalic	brain traits	2.23E-01	4.89E-01	6.88E-10	9.01E-10	1.225
insomnia	Upper rhombic lip	brain traits	7.03E-03	6.51E-02	1.99E-09	8.12E-10	1.349

Trait	*Supercluster*	*disorder_type*	*P*	*P.fdr*	*BETA*	*SE*	*Enrichment*
insomnia	Vascular	brain traits	9.81E-01	9.88E-01	-2.00E-09	9.62E-10	0.875
mdd2019	Amygdala excitatory	psychiatric	6.17E-05	6.38E-04	3.34E-09	8.69E-10	1.357
mdd2019	Astrocyte	psychiatric	8.52E-01	1.00E+00	-1.07E-09	1.02E-09	0.926
mdd2019	Bergmann glia	psychiatric	6.96E-01	9.44E-01	-4.39E-10	8.56E-10	1.017
mdd2019	Cerebellar inhibitory	psychiatric	4.49E-03	1.55E-02	2.67E-09	1.02E-09	1.280
mdd2019	CGE interneuron	psychiatric	1.42E-05	2.20E-04	4.63E-09	1.11E-09	1.474
mdd2019	Choroid plexus	psychiatric	1.00E+00	1.00E+00	-3.26E-09	7.45E-10	0.683
mdd2019	Committed oligodendrocyte precursor	psychiatric	9.96E-01	1.00E+00	-2.22E-09	8.33E-10	0.764
mdd2019	Deep-layer corticothalamic and 6b	psychiatric	3.63E-02	8.65E-02	1.74E-09	9.71E-10	1.206
mdd2019	Deep-layer intratelencephalic	psychiatric	1.01E-02	3.13E-02	2.32E-09	9.99E-10	1.276
mdd2019	Deep-layer near-projecting	psychiatric	7.18E-02	1.32E-01	1.40E-09	9.54E-10	1.179
mdd2019	Eccentric medium spiny neuron	psychiatric	2.40E-03	9.30E-03	2.80E-09	9.94E-10	1.333
mdd2019	Ependymal	psychiatric	1.00E+00	1.00E+00	-2.96E-09	8.57E-10	0.701
mdd2019	Fibroblast	psychiatric	9.99E-01	1.00E+00	-2.57E-09	8.01E-10	0.719
mdd2019	Hippocampal CA1-3	psychiatric	1.76E-03	7.79E-03	2.95E-09	1.01E-09	1.311
mdd2019	Hippocampal CA4	psychiatric	1.93E-02	4.98E-02	2.12E-09	1.02E-09	1.266
mdd2019	Hippocampal dentate gyrus	psychiatric	7.19E-02	1.32E-01	1.26E-09	8.60E-10	1.161
mdd2019	LAMP5-LHX6 and Chandelier	psychiatric	1.77E-04	1.37E-03	3.47E-09	9.70E-10	1.405
mdd2019	Lower rhombic lip	psychiatric	7.01E-01	9.44E-01	-5.17E-10	9.81E-10	0.972
mdd2019	Mammillary body	psychiatric	1.47E-01	2.27E-01	9.20E-10	8.76E-10	1.161
mdd2019	Medium spiny neuron	psychiatric	8.55E-02	1.47E-01	1.25E-09	9.12E-10	1.134
mdd2019	MGE interneuron	psychiatric	3.65E-06	1.13E-04	4.71E-09	1.05E-09	1.518
mdd2019	Microglia	psychiatric	9.94E-01	1.00E+00	-2.01E-09	7.98E-10	0.680
mdd2019	Midbrain-derived inhibitory	psychiatric	7.24E-02	1.32E-01	1.48E-09	1.01E-09	1.231
mdd2019	Miscellaneous	psychiatric	1.74E-03	7.79E-03	2.65E-09	9.08E-10	1.268
mdd2019	Oligodendrocyte precursor	psychiatric	9.59E-01	1.00E+00	-1.59E-09	9.14E-10	0.847
mdd2019	Oligodendrocyte	psychiatric	4.24E-02	9.39E-02	1.46E-09	8.46E-10	1.202
mdd2019	Splatter	psychiatric	1.07E-01	1.74E-01	1.22E-09	9.78E-10	1.137
mdd2019	Thalamic excitatory	psychiatric	2.46E-04	1.53E-03	3.24E-09	9.31E-10	1.355
mdd2019	Upper-layer intratelencephalic	psychiatric	6.83E-01	9.44E-01	-4.29E-10	8.99E-10	0.955
mdd2019	Upper rhombic lip	psychiatric	1.66E-02	4.67E-02	2.15E-09	1.01E-09	1.235

Trait	Supercluster	disorder_type	P	P.fdr	BETA	SE	Enrichment
mdd2019	Vascular	psychiatric	9.96E-01	1.00E+00	-2.38E-09	9.08E-10	0.674
neuroticism	Amygdala excitatory	brain traits	3.37E-04	1.31E-03	4.55E-09	1.34E-09	1.301
neuroticism	Astrocyte	brain traits	9.45E-01	1.00E+00	-2.12E-09	1.32E-09	0.998
neuroticism	Bergmann glia	brain traits	7.13E-01	9.62E-01	-8.22E-10	1.46E-09	1.086
neuroticism	Cerebellar inhibitory	brain traits	7.18E-02	1.24E-01	2.02E-09	1.38E-09	1.189
neuroticism	CGE interneuron	brain traits	2.08E-03	5.37E-03	4.20E-09	1.47E-09	1.302
neuroticism	Choroid plexus	brain traits	7.60E-01	9.81E-01	-9.74E-10	1.38E-09	0.993
neuroticism	Committed oligodendrocyte precursor	brain traits	9.74E-01	1.00E+00	-2.34E-09	1.20E-09	0.929
neuroticism	Deep-layer corticothalamic and 6b	brain traits	1.44E-04	8.91E-04	5.26E-09	1.45E-09	1.338
neuroticism	Deep-layer intratelencephalic	brain traits	1.19E-06	3.69E-05	6.97E-09	1.48E-09	1.448
neuroticism	Deep-layer near-projecting	brain traits	5.57E-03	1.15E-02	3.73E-09	1.47E-09	1.274
neuroticism	Eccentric medium spiny neuron	brain traits	2.30E-04	1.09E-03	4.54E-09	1.30E-09	1.336
neuroticism	Ependymal	brain traits	9.57E-01	1.00E+00	-2.26E-09	1.31E-09	0.926
neuroticism	Fibroblast	brain traits	1.00E+00	1.00E+00	-5.02E-09	1.28E-09	0.783
neuroticism	Hippocampal CA1-3	brain traits	1.08E-03	3.72E-03	4.04E-09	1.32E-09	1.269
neuroticism	Hippocampal CA4	brain traits	1.01E-02	1.95E-02	3.12E-09	1.34E-09	1.256
neuroticism	Hippocampal dentate gyrus	brain traits	1.69E-03	4.90E-03	3.57E-09	1.22E-09	1.256
neuroticism	LAMP5-LHX6 and Chandelier	brain traits	2.47E-04	1.09E-03	4.71E-09	1.35E-09	1.357
neuroticism	Lower rhombic lip	brain traits	1.34E-01	2.07E-01	1.53E-09	1.38E-09	1.145
neuroticism	Mammillary body	brain traits	2.25E-03	5.37E-03	3.48E-09	1.22E-09	1.269
neuroticism	Medium spiny neuron	brain traits	3.76E-05	3.89E-04	5.29E-09	1.34E-09	1.327
neuroticism	MGE interneuron	brain traits	1.74E-03	4.90E-03	4.49E-09	1.54E-09	1.347
neuroticism	Microglia	brain traits	9.91E-01	1.00E+00	-2.97E-09	1.25E-09	0.798
neuroticism	Midbrain-derived inhibitory	brain traits	7.23E-02	1.24E-01	2.15E-09	1.48E-09	1.228
neuroticism	Miscellaneous	brain traits	2.37E-05	3.68E-04	5.01E-09	1.23E-09	1.309
neuroticism	Oligodendrocyte precursor	brain traits	9.93E-01	1.00E+00	-3.19E-09	1.30E-09	0.906
neuroticism	Oligodendrocyte	brain traits	1.13E-01	1.85E-01	1.68E-09	1.39E-09	1.179
neuroticism	Splatter	brain traits	2.01E-01	2.97E-01	1.26E-09	1.50E-09	1.133
neuroticism	Thalamic excitatory	brain traits	2.62E-03	5.80E-03	3.93E-09	1.41E-09	1.293
neuroticism	Upper-layer intratelencephalic	brain traits	4.83E-01	6.81E-01	5.91E-11	1.40E-09	1.068
neuroticism	Upper rhombic lip	brain traits	9.19E-05	7.12E-04	4.87E-09	1.30E-09	1.328

Trait	***Supercluster***	***disorder_type***	***P***	***P.fdr***	***BETA***	***SE***	***Enrichment***
neuroticism	Vascular	brain traits	9.99E-01	1.00E+00	-4.45E-09	1.36E-09	0.764
scz2022	Amygdala excitatory	psychiatric	3.91E-05	2.88E-04	1.87E-08	4.74E-09	1.395
scz2022	Astrocyte	psychiatric	9.53E-01	1.00E+00	-8.93E-09	5.35E-09	1.093
scz2022	Bergmann glia	psychiatric	9.10E-01	1.00E+00	-7.47E-09	5.58E-09	1.125
scz2022	Cerebellar inhibitory	psychiatric	1.42E-02	2.94E-02	1.08E-08	4.95E-09	1.335
scz2022	CGE interneuron	psychiatric	2.28E-06	3.53E-05	2.14E-08	4.68E-09	1.485
scz2022	Choroid plexus	psychiatric	9.86E-01	1.00E+00	-1.06E-08	4.83E-09	1.014
scz2022	Committed oligodendrocyte precursor	psychiatric	8.25E-01	1.00E+00	-4.12E-09	4.41E-09	1.129
scz2022	Deep-layer corticothalamic and 6b	psychiatric	6.12E-04	2.37E-03	1.48E-08	4.57E-09	1.349
scz2022	Deep-layer intratelencephalic	psychiatric	1.27E-05	1.31E-04	2.30E-08	5.46E-09	1.502
scz2022	Deep-layer near-projecting	psychiatric	4.60E-02	8.90E-02	7.64E-09	4.53E-09	1.261
scz2022	Eccentric medium spiny neuron	psychiatric	4.64E-05	2.88E-04	1.80E-08	4.61E-09	1.428
scz2022	Ependymal	psychiatric	9.98E-01	1.00E+00	-1.39E-08	4.86E-09	0.972
scz2022	Fibroblast	psychiatric	1.00E+00	1.00E+00	-2.51E-08	5.10E-09	0.813
scz2022	Hippocampal CA1-3	psychiatric	8.12E-07	2.52E-05	2.10E-08	4.37E-09	1.435
scz2022	Hippocampal CA4	psychiatric	6.75E-05	3.49E-04	1.60E-08	4.19E-09	1.393
scz2022	Hippocampal dentate gyrus	psychiatric	5.30E-03	1.49E-02	1.25E-08	4.90E-09	1.323
scz2022	LAMP5-LHX6 and Chandelier	psychiatric	2.41E-03	8.32E-03	1.36E-08	4.83E-09	1.377
scz2022	Lower rhombic lip	psychiatric	3.16E-01	4.67E-01	2.07E-09	4.34E-09	1.178
scz2022	Mammillary body	psychiatric	1.47E-01	2.41E-01	4.71E-09	4.50E-09	1.215
scz2022	Medium spiny neuron	psychiatric	5.99E-03	1.55E-02	1.21E-08	4.83E-09	1.311
scz2022	MGE interneuron	psychiatric	6.72E-03	1.60E-02	1.21E-08	4.88E-09	1.356
scz2022	Microglia	psychiatric	1.00E+00	1.00E+00	-1.64E-08	4.28E-09	0.892
scz2022	Midbrain-derived inhibitory	psychiatric	5.67E-02	9.76E-02	6.90E-09	4.36E-09	1.268
scz2022	Miscellaneous	psychiatric	3.09E-03	9.59E-03	1.28E-08	4.69E-09	1.310
scz2022	Oligodendrocyte precursor	psychiatric	5.83E-01	8.21E-01	-1.14E-09	5.45E-09	1.182
scz2022	Oligodendrocyte	psychiatric	5.51E-02	9.76E-02	6.96E-09	4.36E-09	1.253
scz2022	Splatter	psychiatric	8.33E-01	1.00E+00	-4.21E-09	4.36E-09	1.075
scz2022	Thalamic excitatory	psychiatric	1.06E-02	2.36E-02	9.54E-09	4.14E-09	1.289
scz2022	Upper-layer intratelencephalic	psychiatric	2.68E-01	4.16E-01	3.13E-09	5.06E-09	1.228
scz2022	Upper rhombic lip	psychiatric	5.40E-04	2.37E-03	1.78E-08	5.45E-09	1.424

Trait	*Supercluster*	*disorder_type*	*P*	*P.fdr*	*BETA*	*SE*	*Enrichment*
scz2022	Vascular	psychiatric	9.99E-01	1.00E+00	-1.55E-08	4.91E-09	0.930

Classified by disease and alphabetically sorted by cluster/ao et al.,; BETA = beta coefficient; SE = standard error; P.adj = adjusted p value

Table S10: Single nucleus enrichment analysis on hear

Trait	*Supercluster*	*Coefficient_z-score*	*if.sig.fdr*	*P.adj*
bip2021	Amygdala excitatory	3.614	yes	0.001647971
bip2021	Astrocyte	-2.238	no	0.999999576
bip2021	Bergmann glia	-0.798	no	0.999999576
bip2021	Cerebellar inhibitory	2.614	yes	0.018556757
bip2021	CGE interneuron	3.224	yes	0.004201661
bip2021	Choroid plexus	-2.396	no	0.999999576
bip2021	Committed oligodendrocyte precursor	-1.569	no	0.999999576
bip2021	Deep-layer corticothalamic and 6b	2.031	yes	0.060466055
bip2021	Deep-layer intratelencephalic	4.579	yes	1,08E+12
bip2021	Deep-layer near-projecting	2.136	yes	0.050539943
bip2021	Eccentric medium spiny neuron	3.661	yes	0.001558106
bip2021	Ependymal	-3.338	no	0.999999576
bip2021	Fibroblast	-3.305	no	0.999999576
bip2021	Hippocampal CA1-3	3.290	yes	0.003733175
bip2021	Hippocampal CA4	3.150	yes	0.005236121
bip2021	Hippocampal dentate gyrus	3.453	yes	0.002240254
bip2021	LAMP5-LHX6 and Chandelier	2.199	yes	0.044689886
bip2021	Lower rhombic lip	-1.535	no	0.999999576
bip2021	Mammillary body	-0.469	no	0.970464278
bip2021	Medium spiny neuron	2.105	yes	0.052962387
bip2021	MGE interneuron	3.032	yes	0.007287292
bip2021	Microglia	-1.580	no	0.999999576
bip2021	Midbrain-derived inhibitory	1.128	no	0.270790252
bip2021	Miscellaneous	2.460	yes	0.025634174
bip2021	Oligodendrocyte precursor	-2.154	no	0.999999576
bip2021	Oligodendrocyte	-1.261	no	0.999999576
bip2021	Splatter	-2.037	no	0.999999576
bip2021	Thalamic excitatory	1.467	no	0.164226151
bip2021	Upper-layer intratelencephalic	0.871	yes	0.001728208
bip2021	Upper rhombic lip	3.582	no	0.346498985

Trait	*Supercluster*	*Coefficient_z-score*	*if.sig.fdr*	*P.adj*
bip2021	Vascular	-3.742	no	0.999999576
hearing_loss	Amygdala excitatory	2.216	yes	0.043497782
hearing_loss	Astrocyte	-1.002	no	0.999999576
hearing_loss	Bergmann glia	0.213	no	0.660921704
hearing_loss	Cerebellar inhibitory	-1.296	no	0.999999576
hearing_loss	CGE interneuron	1.028	no	0.294355006
hearing_loss	Choroid plexus	-0.858	no	0.999999576
hearing_loss	Committed oligodendrocyte precursor	0.179	no	0.669061456
hearing_loss	Deep-layer corticothalamic and 6b	1.190	no	0.250163558
hearing_loss	Deep-layer intratelencephalic	1.950	yes	0.071062896
hearing_loss	Deep-layer near-projecting	1.843	yes	0.088094495
hearing_loss	Eccentric medium spiny neuron	0.840	no	0.356092532
hearing_loss	Ependymal	0.132	no	0.687787789
hearing_loss	Fibroblast	1.052	no	0.288637478
hearing_loss	Hippocampal CA1-3	2.077	yes	0.05581955
hearing_loss	Hippocampal CA4	-0.067	no	0.796236714
hearing_loss	Hippocampal dentate gyrus	0.407	no	0.55793081
hearing_loss	LAMP5-LHX6 and Chandelier	1.666	no	0.121930379
hearing_loss	Lower rhombic lip	0.204	no	0.660921704
hearing_loss	Mammillary body	1.057	no	0.288637478
hearing_loss	Medium spiny neuron	1.446	no	0.166145716
hearing_loss	MGE interneuron	1.013	no	0.298285674
hearing_loss	Microglia	-1.152	no	0.999999576
hearing_loss	Midbrain-derived inhibitory	0.929	no	0.331662625
hearing_loss	Miscellaneous	0.692	no	0.417377752
hearing_loss	Oligodendrocyte precursor	-0.925	no	0.981862301
hearing_loss	Oligodendrocyte	-0.546	no	0.999999576
hearing_loss	Splatter	0.874	no	0.346498985
hearing_loss	Thalamic excitatory	-0.427	no	0.966544645
hearing_loss	Upper-layer intratelencephalic	0.261	no	0.123576739
hearing_loss	Upper rhombic lip	1.653	no	0.636794375

Trait	*Supercluster*	*Coefficient_z-score*	*if.sig.fdr*	*P.adj*
hearing_loss	Vascular	-0.696	no	0.999999576
insomnia	Amygdala excitatory	2.907	yes	0.00942258
insomnia	Astrocyte	-1.723	no	0.999999576
insomnia	Bergmann glia	-0.334	no	0.931124972
insomnia	Cerebellar inhibitory	0.709	no	0.411967634
insomnia	CGE interneuron	2.779	yes	0.012085021
insomnia	Choroid plexus	-1.485	no	0.999999576
insomnia	Committed oligodendrocyte precursor	-0.477	no	0.970464278
insomnia	Deep-layer corticothalamic and 6b	1.061	no	0.288637478
insomnia	Deep-layer intratelencephalic	2.391	yes	0.030063241
insomnia	Deep-layer near-projecting	1.914	yes	0.076080034
insomnia	Eccentric medium spiny neuron	0.667	no	0.426588002
insomnia	Ependymal	-0.378	no	0.948056573
insomnia	Fibroblast	-2.246	no	0.999999576
insomnia	Hippocampal CA1-3	0.970	no	0.315008097
insomnia	Hippocampal CA4	0.878	no	0.346498985
insomnia	Hippocampal dentate gyrus	0.172	no	0.669061456
insomnia	LAMP5-LHX6 and Chandelier	2.015	yes	0.06187812
insomnia	Lower rhombic lip	0.736	no	0.401159856
insomnia	Mammillary body	-0.984	no	0.999999576
insomnia	Medium spiny neuron	0.295	no	0.62121173
insomnia	MGE interneuron	2.243	yes	0.041394362
insomnia	Microglia	-0.943	no	0.999999576
insomnia	Midbrain-derived inhibitory	1.172	no	0.25474205
insomnia	Miscellaneous	-0.087	no	0.80218329
insomnia	Oligodendrocyte precursor	-1.994	no	0.999999576
insomnia	Oligodendrocyte	-0.869	no	0.999999576
insomnia	Splatter	0.442	no	0.541823182
insomnia	Thalamic excitatory	0.893	no	0.345905112
insomnia	Upper-layer intratelencephalic	0.764	yes	0.025634174
insomnia	Upper rhombic lip	2.456	no	0.390522603

Trait	Supercluster	Coefficient_z-score	if.sig.fdr	P.adj
insomnia	Vascular	-2.078	no	0.999999576
mdd2019	Amygdala excitatory	3.839	yes	9,57E+11
mdd2019	Astrocyte	-1.046	no	0.999999576
mdd2019	Bergmann glia	-0.512	no	0.979992891
mdd2019	Cerebellar inhibitory	2.613	yes	0.018556757
mdd2019	CGE interneuron	4.186	yes	3,77E+12
mdd2019	Choroid plexus	-4.379	no	0.999999576
mdd2019	Committed oligodendrocyte precursor	-2.662	no	0.999999576
mdd2019	Deep-layer corticothalamic and 6b	1.796	yes	0.096341778
mdd2019	Deep-layer intratelencephalic	2.323	yes	0.034759647
mdd2019	Deep-layer near-projecting	1.462	no	0.164226151
mdd2019	Eccentric medium spiny neuron	2.820	yes	0.011227221
mdd2019	Ependymal	-3.451	no	0.999999576
mdd2019	Fibroblast	-3.211	no	0.999999576
mdd2019	Hippocampal CA1-3	2.918	yes	0.009346929
mdd2019	Hippocampal CA4	2.069	yes	0.056027172
mdd2019	Hippocampal dentate gyrus	1.462	no	0.164226151
mdd2019	LAMP5-LHX6 and Chandelier	3.573	yes	0.001728208
mdd2019	Lower rhombic lip	-0.527	no	0.979992891
mdd2019	Mammillary body	1.051	no	0.288637478
mdd2019	Medium spiny neuron	1.369	no	0.189254026
mdd2019	MGE interneuron	4.485	yes	1,36E+12
mdd2019	Microglia	-2.522	no	0.999999576
mdd2019	Midbrain-derived inhibitory	1.458	no	0.164226151
mdd2019	Miscellaneous	2.921	yes	0.009346929
mdd2019	Oligodendrocyte precursor	-1.736	no	0.111041993
mdd2019	Oligodendrocyte	1.724	no	0.999999576
mdd2019	Splatter	1.244	no	0.233727894
mdd2019	Thalamic excitatory	3.485	yes	0.002084962
mdd2019	Upper-layer intratelencephalic	-0.478	yes	0.050539943
mdd2019	Upper rhombic lip	2.130	no	0.970464278

Trait	*Supercluster*	*Coefficient_z-score*	*if.sig.fdr*	*P.adj*
mdd2019	Vascular	-2.624	no	0.999999576
neuroticism	Amygdala excitatory	3.400	yes	0.002614463
neuroticism	Astrocyte	-1.602	no	0.999999576
neuroticism	Bergmann glia	-0.564	no	0.983032489
neuroticism	Cerebellar inhibitory	1.462	no	0.164226151
neuroticism	CGE interneuron	2.865	yes	0.01046776
neuroticism	Choroid plexus	-0.705	no	0.999999576
neuroticism	Committed oligodendrocyte precursor	-1.946	no	0.999999576
neuroticism	Deep-layer corticothalamic and 6b	3.626	yes	0.001647971
neuroticism	Deep-layer intratelencephalic	4.718	yes	1,08E+12
neuroticism	Deep-layer near-projecting	2.538	yes	0.022028991
neuroticism	Eccentric medium spiny neuron	3.503	yes	0.002084962
neuroticism	Ependymal	-1.720	no	0.999999576
neuroticism	Fibroblast	-3.920	no	0.999999576
neuroticism	Hippocampal CA1-3	3.067	yes	0.006692759
neuroticism	Hippocampal CA4	2.324	yes	0.034759647
neuroticism	Hippocampal dentate gyrus	2.932	yes	0.009346929
neuroticism	LAMP5-LHX6 and Chandelier	3.484	yes	0.002084962
neuroticism	Lower rhombic lip	1.109	no	0.276377621
neuroticism	Mammillary body	2.840	yes	0.011031051
neuroticism	Medium spiny neuron	3.959	yes	7,27E+11
neuroticism	MGE interneuron	2.922	yes	0.009346929
neuroticism	Microglia	-2.381	no	0.999999576
neuroticism	Midbrain-derived inhibitory	1.459	no	0.164226151
neuroticism	Miscellaneous	4.068	yes	5,51E+11
neuroticism	Oligodendrocyte precursor	-2.461	no	0.244716818
neuroticism	Oligodendrocyte	1.210	no	0.999999576
neuroticism	Splatter	0.838	no	0.356092532
neuroticism	Thalamic excitatory	2.792	yes	0.011874837
neuroticism	Upper-layer intratelencephalic	0.042	yes	0.001220881
neuroticism	Upper rhombic lip	3.740	no	0.736677639

Trait	*Supercluster*	*Coefficient_z-score*	*if.sig.fdr*	*P.adj*
neuroticism	Vascular	-3.263	no	0.999999576
scz2022	Amygdala excitatory	3.950	yes	7,27E+11
scz2022	Astrocyte	-1.670	no	0.999999576
scz2022	Bergmann glia	-1.339	no	0.999999576
scz2022	Cerebellar inhibitory	2.191	yes	0.044850486
scz2022	CGE interneuron	4.584	yes	1,08E+12
scz2022	Choroid plexus	-2.190	no	0.999999576
scz2022	Committed oligodendrocyte precursor	-0.935	no	0.999999576
scz2022	Deep-layer corticothalamic and 6b	3.233	yes	0.004201661
scz2022	Deep-layer intratelencephalic	4.211	yes	3,77E+12
scz2022	Deep-layer near-projecting	1.685	no	0.118706049
scz2022	Eccentric medium spiny neuron	3.909	yes	7,85E+11
scz2022	Ependymal	-2.851	no	0.999999576
scz2022	Fibroblast	-4.924	no	0.999999576
scz2022	Hippocampal CA1-3	4.795	yes	1,08E+12
scz2022	Hippocampal CA4	3.817	yes	9,66E+11
scz2022	Hippocampal dentate gyrus	2.555	yes	0.021443306
scz2022	LAMP5-LHX6 and Chandelier	2.818	yes	0.011227221
scz2022	Lower rhombic lip	0.478	no	0.525610614
scz2022	Mammillary body	1.048	no	0.288637478
scz2022	Medium spiny neuron	2.513	yes	0.023195824
scz2022	MGE interneuron	2.472	yes	0.025526232
scz2022	Microglia	-3.836	no	0.999999576
scz2022	Midbrain-derived inhibitory	1.583	no	0.1387516
scz2022	Miscellaneous	2.738	yes	0.013374532
scz2022	Oligodendrocyte precursor	-0.208	no	0.136737077
scz2022	Oligodendrocyte	1.597	no	0.866810961
scz2022	Splatter	-0.967	no	0.999999576
scz2022	Thalamic excitatory	2.303	yes	0.036001552
scz2022	Upper-layer intratelencephalic	0.618	yes	0.003865074
scz2022	Upper rhombic lip	3.269	no	0.449780819

Trait	***Supercluster***	***Coefficient_z-score***	***if.sig.fdr***	***P.adj***
scz2022	Vascular	-3.146	no	0.999999576

Classified by disease and alphabetically sorted by cluster for included studies in the current analysis

4. References

1. Harris, T.B., Launer, L.J., Eiriksdottir, G., Kjartansson, O., Jonsson, P.V., Sigurdsson, G., Thorgeirsson, G., Aspelund, T., Garcia, M.E., Cotch, M.F., et al. (2007). Age, Gene/Environment Susceptibility-Reykjavik Study: multidisciplinary applied phenomics. Am J Epidemiol *165*, 1076–1087. https://doi.org/10.1093/aje/kwk115.

2. Pedersen, D.A., Larsen, L.A., Nygaard, M., Mengel-From, J., McGue, M., Dalgård, C., Hvidberg, L., Hjelmborg, J., Skytthe, A., Holm, N.V., et al. (2019). The Danish Twin Registry: An Updated Overview. Twin Res Hum Genet *22*, 499–507. https://doi.org/10.1017/thg.2019.72.

3. Milani, L., Alver, M., Laur, S., Reisberg, S., Haller, T., Aasmets, O., Abner, E., Alavere, H., Allik, A., Annilo, T., et al. (2025). The Estonian Biobank’s journey from biobanking to personalized medicine. Nat Commun *16*, 3270. https://doi.org/10.1038/s41467-025-58465-3.

4. Mbatchou, J., Barnard, L., Backman, J., Marcketta, A., Kosmicki, J.A., Ziyatdinov, A., Benner, C., O’Dushlaine, C., Barber, M., Boutkov, B., et al. (2021). Computationally efficient whole-genome regression for quantitative and binary traits. Nat Genet *53*, 1097–1103. https://doi.org/10.1038/s41588-021-00870-7.

5. Dawber, T.R., Kannel, W.B., and Lyell, L.P. (1963). An approach to longitudinal studies in a community: the Framingham Study. Ann N Y Acad Sci *107*, 539–556. https://doi.org/10.1111/j.1749-6632.1963.tb13299.x.

6. Feinleib, M., Kannel, W.B., Garrison, R.J., McNamara, P.M., and Castelli, W.P. (1975). The Framingham Offspring Study. Design and preliminary data. Prev Med *4*, 518–525. https://doi.org/10.1016/0091-7435(75)90037-7.

7. Splansky, G.L., Corey, D., Yang, Q., Atwood, L.D., Cupples, L.A., Benjamin, E.J., D’Agostino, R.B., Fox, C.S., Larson, M.G., Murabito, J.M., et al. (2007). The Third Generation Cohort of the National Heart, Lung, and Blood Institute’s Framingham Heart Study: design, recruitment, and initial examination. Am J Epidemiol *165*, 1328–1335. https://doi.org/10.1093/aje/kwm021.

8. Sardone, R., Battista, P., Donghia, R., Lozupone, M., Tortelli, R., Guerra, V., Grasso, A., Griseta, C., Castellana, F., Zupo, R., et al. (2020). Age-Related Central Auditory Processing Disorder, MCI, and Dementia in an Older Population of Southern Italy. Otolaryngol Head Neck Surg *163*, 348–355. https://doi.org/10.1177/0194599820913635.

9. Stolk, R.P., Rosmalen, J.G.M., Postma, D.S., de Boer, R.A., Navis, G., Slaets, J.P.J., Ormel, J., and Wolffenbuttel, B.H.R. (2008). Universal risk factors for multifactorial diseases: LifeLines: a three-generation population-based study. Eur J Epidemiol *23*, 67–74. https://doi.org/10.1007/s10654-007-9204-4.

10. Scholtens, S., Smidt, N., Swertz, M.A., Bakker, S.J.L., Dotinga, A., Vonk, J.M., van Dijk, F., van Zon, S.K.R., Wijmenga, C., Wolffenbuttel, B.H.R., et al. (2015). Cohort Profile: LifeLines, a three-generation cohort study and biobank. Int J Epidemiol *44*, 1172–1180. https://doi.org/10.1093/ije/dyu229.

11. Carss, K.J., Arno, G., Erwood, M., Stephens, J., Sanchis-Juan, A., Hull, S., Megy, K., Grozeva, D., Dewhurst, E., Malka, S., et al. (2017). Comprehensive Rare Variant Analysis via Whole-Genome Sequencing to Determine the Molecular Pathology of Inherited Retinal Disease. The American Journal of Human Genetics *100*, 75–90. https://doi.org/10/f9ktdw.

12. Ikram, M.A., Brusselle, G., Ghanbari, M., Goedegebure, A., Ikram, M.K., Kavousi, M., Kieboom, B.C.T., Klaver, C.C.W., de Knegt, R.J., Luik, A.I., et al. (2020). Objectives, design and main findings until 2020 from the Rotterdam Study. Eur J Epidemiol *35*, 483–517. https://doi.org/10.1007/s10654-020-00640-5.

13. Zagai, U., Lichtenstein, P., Pedersen, N.L., and Magnusson, P.K.E. (2019). The Swedish Twin Registry: Content and Management as a Research Infrastructure. Twin Res Hum Genet *22*, 672–680. https://doi.org/10.1017/thg.2019.99.

14. Moayyeri, A., Hammond, C.J., Valdes, A.M., and Spector, T.D. (2013). Cohort Profile: TwinsUK and healthy ageing twin study. Int J Epidemiol *42*, 76–85. https://doi.org/10.1093/ije/dyr207.

15. Sudlow, C., Gallacher, J., Allen, N., Beral, V., Burton, P., Danesh, J., Downey, P., Elliott, P., Green, J., Landray, M., et al. (2015). UK biobank: an open access resource for identifying the causes of a wide range of complex diseases of middle and old age. PLoS Med *12*, e1001779. https://doi.org/10.1371/journal.pmed.1001779.

5. **Study specific acknowledgements**

The **AGES-Reykjavik** study has been funded by NIH contract N01-AG012100, the NIA Intramural Research Program, an Intramural Research Program Award (ZIAEY000401) from the National Eye Institute, an award from the National Institute on Deafness and Other Communication Disorders (NIDCD) Division of Scientific Programs (IAA Y2-DC_1004-02), Hjartavernd (the Icelandic Heart Association), and the Althingi (the Icelandic Parliament). The researchers are indebted to the participants for their willingness to participate in the study.

DTR has been supported by grants from The National Program for Research Infrastructure 2007 from the Danish Agency for Science, Technology and Innovation (09-063256) and the US National Institutes of Health (P01 AG08761). Genotyping was supported by NIH R01 AG037985 (Pedersen).

The **FinnGen** project is funded by two grants from Business Finland (HUS 4685/31/2016 and UH 4386/31/2016) and the following industry partners: AbbVie Inc., AstraZeneca UK Ltd, Biogen MA Inc., Bristol Myers Squibb (and Celgene Corporation & Celgene International II Sàrl), Genentech Inc., Merck Sharp & Dohme Corp, Pfizer Inc., GlaxoSmithKline Intellectual Property Development Ltd., Sanofi US Services Inc., Maze Therapeutics Inc., Janssen Biotech Inc, Novartis AG, and Boehringer Ingelheim. The following biobanks are acknowledged for delivering biobank samples to FinnGen: Auria Biobank (www.auria.fi/biopankki), THL Biobank (www.thl.fi/biobank), Helsinki Biobank (www.helsinginbiopankki.fi), Biobank Borealis of Northern Finland (https://www.ppshp.fi/Tutkimus-ja-opetus/Biopankki/Pages/Biobank-Borealis-briefly-in-English.aspx), Finnish Clinical Biobank Tampere (www.tays.fi/en-US/Research_and_development/Finnish_Clinical_Biobank_Tampere), Biobank of Eastern Finland (www.ita-suomenbiopankki.fi/en), Central Finland Biobank (www.ksshp.fi/fi-FI/Potilaalle/Biopankki), Finnish Red Cross Blood Service Biobank (www.veripalvelu.fi/verenluovutus/biopankkitoiminta) and Terveystalo Biobank (www.terveystalo.com/fi/Yritystietoa/Terveystalo-Biopankki/Biopankki/). All Finnish Biobanks are members of BBMRI.fi infrastructure (www.bbmri.fi). Finnish Biobank Cooperative -FINBB (https://finbb.fi/) is the coordinator of BBMRI-ERIC operations in Finland. The Finnish biobank data can be accessed through the Fingenious® services (https://site.fingenious.fi/en/) managed by FINBB.The researchers would like to acknowledge all participants and investigators of the FinnGen study.

The **Framingham Heart Study** is conducted and supported by the National Heart, Lung, and Blood Institute (NHLBI) in collaboration with Boston University (Contract No. N01-HC-25195 and HHSN268201500001). This manuscript was not prepared in collaboration with investigators of the

Framingham Heart Study and does not necessarily reflect the opinions or views of the Framingham Heart Study, Boston University, or NHLBI. Funding for SHARe Affymetrix genotyping was provided by NHLBI Contract N02-HL- 64278. Nancy L. Heard-Costa is supported by NIH/NHLBI HHSN268201500001 (Ramachandran). Further support was provided by P30AG066546, funded by the National Institute on Aging (HHS - NIH), awarded to University of Texas Health Science Center of San Antonio.

The Great Age study (Salus in Apulia study) were funded by Apulia Government and Italian Ministry of Health, under the Studies on Aging Network, at Italian Research Hospitals (IRCCS). The authors thank the MICOL Study group, the Salus in Apulia Research Team, and the General Practitioners of Castellana Grotte for the fundamental role in recruiting participants to this study.

The **Health, Aging and Body Composition Study** was supported by the Intramural Research Program, National Institute on Aging, National Institutes of Health, Department of Health and Human Services, project ZO1 AG000949. This work utilized the computational resources of the NIH STRIDES Initiative (https://cloud.nih.gov) through the Other Transaction agreement - Azure: OT2OD032100, Google Cloud Platform: OT2OD027060, Amazon Web Services: OT2OD027852. This work utilized the computational resources of the NIH HPC Biowulf cluster (https://hpc.nih.gov). The Health, Aging, and Body Composition Study (Health ABC) was supported by National Institute on Aging (NIA) Contracts N01-AG-6-2101; N01-AG-6-2103; N01-AG-6-2106; NIA grant R01-AG028050, and NINR grant R01-NR012459. This research was funded in part by the Intramural Research Program of the NIH, National Institute on Aging.

The **Lifelines** initiative has been made possible by subsidy from the Dutch Ministry of Health, Welfare and Sport, the Dutch Ministry of Economic Affairs, the University Medical Center Groningen (UMCG), Groningen University and the Provinces in the North of the Netherlands (Drenthe, Friesland, Groningen). The generation and management of GWAS genotype data for the Lifelines Cohort Study is supported by the UMCG Genetics Lifelines Initiative (UGLI). UGLI is partly supported by a Spinoza Grant from NWO, awarded to Cisca Wijmenga. The authors wish to acknowledge the services of the Lifelines Cohort Study, the contributing research centers delivering data to Lifelines, and all the study participants.

The **Rotterdam Study** is funded by Erasmus Medical Center and Erasmus University, Rotterdam, Netherlands Organisation for the Health Research and Development (ZonMw), the Research Institute for Diseases in the Elderly (RIDE), the Ministry of Education, Culture and Science, the Ministry for Health, Welfare and Sports, the European Commission (DG XII), and the Municipality of Rotterdam. The authors are grateful to the study participants, the staff from the Rotterdam Study and the participating general practitioners and pharmacists. The generation and management of GWAS

genotype data for the Rotterdam Study (RS I, RS II, RS III) was executed by the Human Genotyping Facility of the Genetic Laboratory of the Department of Internal Medicine, Erasmus MC, Rotterdam, The Netherlands. The GWAS datasets are supported by the Netherlands Organization of Scientific Research NWO Investments (nr. 175.010.2005.011, 911-03-012), the Genetic Laboratory of the Department of Internal Medicine, Erasmus MC, the Research Institute for Diseases in the Elderly (014-93-015; RIDE2), the Netherlands Genomics Initiative (NGI)/Netherlands Organization for Scientific Research (NWO) Netherlands Consortium for Healthy Aging (NCHA), project nr. 050-060-810. We thank Pascal Arp, Mila Jhamai, Marijn Verkerk, Lizbeth Herrera and Marjolein Peters, MSc, and Carolina Medina-Gomez, MSc, for their help in creating the GWAS database, and Karol Estrada, PhD, Yurii Aulchenko, PhD, and Carolina Medina-Gomez, MSc, for the creation and analysis of imputed data.

For the **TwinsUK** study, the authors of this paper wish to express their appreciation to all study participants.. TwinsUK is funded by the Wellcome Trust, Medical Research Council, European Union, the National Institute for Health Research (NIHR)-funded BioResource, Clinical Research Facility and Biomedical Research Centre based at Guy’s and St Thomas’ NHS Foundation Trust in partnership with King’s College London. HRRW is funded by Action on Hearing Loss, CJS is funded under a grant from the Chronic Disease Research Foundation (CDRF). FMKW is supported by Arthritis Research UK grant number 20682.
